# Palladium-Catalyzed Regioselective B(3,5)-Dialkenylation and B(4)-Alkenylation of *o*-Carboranes

**DOI:** 10.1021/acs.joc.3c02496

**Published:** 2024-02-02

**Authors:** Shasha Yuan, Huifang Zhang, Zaozao Qiu, Zuowei Xie

**Affiliations:** † School of Materials and Chemistry, University of Shanghai for Science and Technology, Shanghai 200093, China; ‡ Shanghai-Hong Kong Joint Laboratory in Chemical Synthesis, Shanghai Institute of Organic Chemistry, University of Chinese Academy of Sciences, Chinese Academy of Sciences, Shanghai 200032, China; § Innovation Institute of Carbon Neutrality and International Joint Laboratory of Catalytic Chemistry, Department of Chemistry, College of Sciences, Shanghai University, Shanghai 200444, China; || Department of Chemistry, The Chinese University of Hong Kong, Shatin, N. T., Hong Kong, China; ⊥ Shenzhen Grubbs Institute and Department of Chemistry, Southern University of Science and Technology, Shenzhen 518055, China

## Abstract

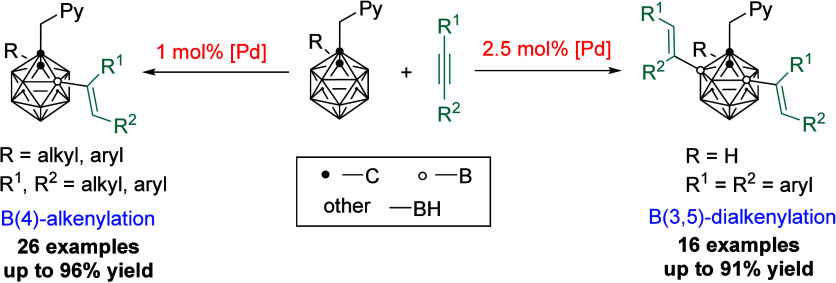

Picolyl group directed B(3,5)-dialkenylation and B(4)-monoalkenylation of *o*-carboranes has been developed with a very low palladium catalyst loading. The degree of substitution is determined by the cage C(2)-substituents due to steric reasons. On the basis of experimental results, a plausible mechanism is proposed including electrophilic palladation and alkyne insertion followed by protonation.

## Introduction

**Scheme 1:**
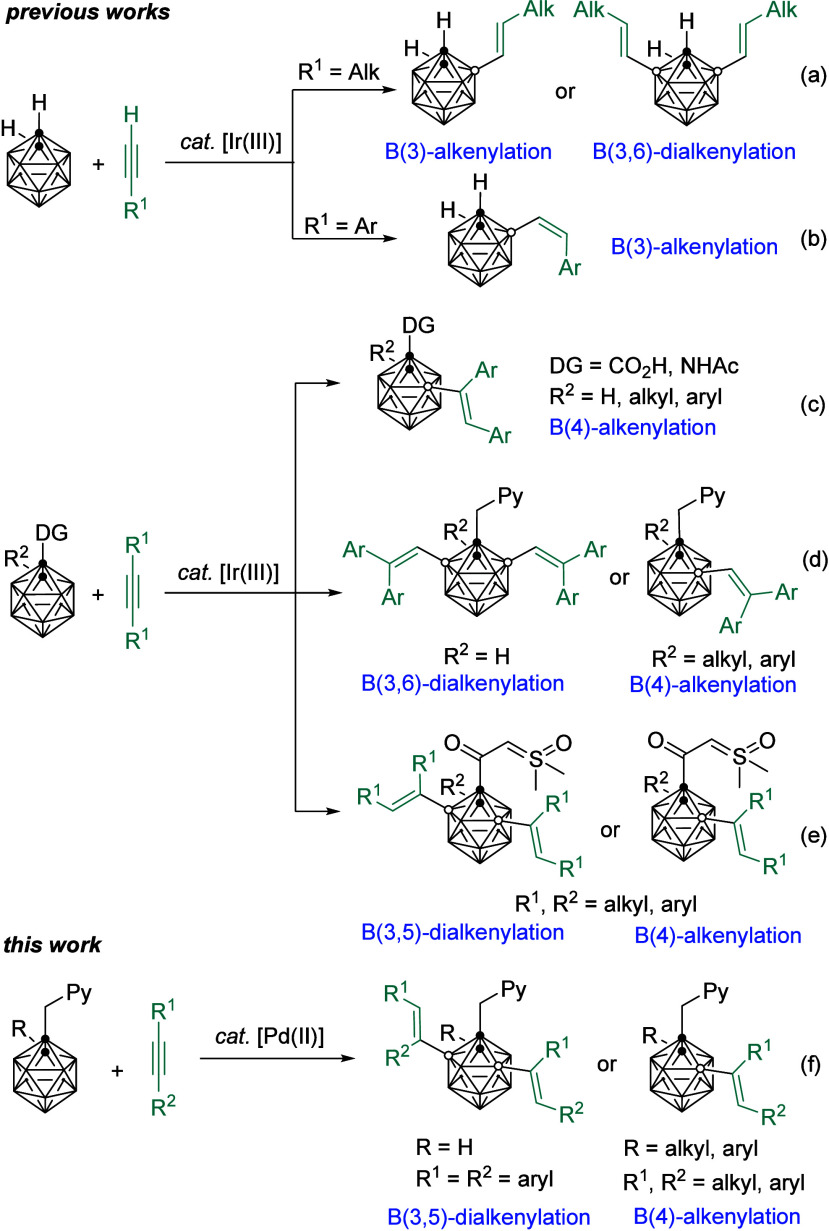
Transition Metal Catalyzed B–H Alkenylation of *o*-Carboranes with Alkynes

Carboranes are a class of polyhedral boron hydride molecular clusters in which one or more of the BH vertices are replaced by CH units.^1^ Their structural features, such as spherical geometry and 3-D electron delocalization,^1c^ make them valuable functional units for a variety of applications ranging from functional materials to pharmaceuticals.^2,3^ Recently, transition metal catalyzed *o*-carborane B–H activation has been rapidly developed,^4^ offering a series of methodologies for cage boron functionalization.^5^ However, different regioselectivity among ten very similar B–H bonds is still challenging for the construction of new B–C and B–heteroatom bonds in functional carborane molecules. The B(3,6)- and B(4,5)-substitution patterns are more common in difunctionalization processes via C(1)-directed B–H bond activation,^6,7^ while there are also a few reports of B(3,4)- and B(3,5)-disubstitution modes.^8,9^ For transition metal catalyzed cage B–H alkenylation with alkynes, Ir catalysis has been broadly used to generate different types of products under various reaction conditions. Sneddon described the first example of [Cp*IrCl_2_]_2_ catalyzed hydroboration of propyne with *o*-carborane B(3)–H to give 3-(*trans*-propenyl)-*o*-carborane in 40% GC yield (Scheme 1a).^10^ A series of terminal alkynes were later found to be compatible with a [Cp*IrCl_2_]_2_ catalyzed alkenylation process at the most electron-deficient B(3/6)–H vertices. The configurations of the resultant olefins were controlled by the nature of the alkyne substituents (Scheme 1a and 1b).^11^ A carboxy or acetylamino directing group assisted Ir(III) catalyzed B(4)-alkenylation was reported to perform in a *cis*-addition manner (Scheme 1c).^12−14^ On the other hand, B(3,6)–H or B(4)–H geminal addition via 1,2-carbon migration of alkynes was developed under the assistance of the picolyl directing group at cage C(1).^15^ The degree of substitution and regioselectivity were determined by the cage C(2)-substituents due to steric reasons (Scheme 1d). Replacement of the Ir by Pd catalyst in the aforementioned reaction offered unprecedentedly a B(3,5)–H regioselectivity,^9^ which was very different from that of Ir catalysis. Herein, we report these results (Scheme 1f). Noted that during the preparation of our manuscript, the Lee group reported a similar work on a sulfoxonium ylide directing group guided Ir catalyzed B(3,5)-dialkenylation and B(4)-monoalkenylation of *o*-carborane (Scheme 1e).^16^

## Results and Discussion

We initially examined different Pd catalysts for regioselective B(3,5)–H activation using 1-(2′-picolyl)-*o*-carborane (**1a**) and PhC≡CPh (**2a**) as model substrates in the presence of 10 mol % AgNTf_2_ and 0.5 equiv of HOAc in toluene at 130 °C. The Pd(0) catalyst Pd(PPh_3_)_4_ was inactive (Table 1, entry 1), whereas 5 mol % Pd(OAc)_2_ afforded the B(3,5)-dialkenylation species **3aa** in 66% yield, in addition to a small amount of B(4)-alkenylation byproduct **4aa** (Table 1, entry 2). Subsequent evaluation found that PdCl_2_ without any ligand demonstrated the best reactivity (Table 1, entries 3–6). Control experiments showed that a mixture of products was observed in the absence of silver salt additive. Silver salts, such as AgTFA and AgOTf, showed lower reaction efficiencies, whereas AgBF_4_ led to the formation of **3aa** in 90% yield (Table 1, entries 7–10). The absence of acid additive resulted in a poor reaction efficiency (Table 1, entry 11). Reducing the PdCl_2_ catalyst loading to 2.5 mol % and the amount of AgNTf_2_ additive to 5 mol % did not affect the yield of **3aa** (Table 1, entries 12 and 13). Lowering the **2a** amount to 1 equiv and catalyst loading to 1 mol % led to the formation of **3aa** and **4aa** in 13% and 68% yields, respectively (Table 1, entry 14). On the other hand, cage C(2)-Me substrate, 1-(2′-picolyl)-2-methyl-*o*-carborane (**1b**), offered a mixture of dialkenylated/monoalkenylated *o*-carboranes in a ratio of 67/24 in the presence of 2.5 mol % Pd catalyst and an excess amount of **2a** (Table 1, entries 15 and 16). However, lowering the amount of **2a** to 1.0 equiv afforded the B(4)-monoalkenylation product **4ba** in 94% yield (Table 1, entry 17). In this case, PdCl_2_ can be further reduced to 1 mol % loading, representing the lowest catalyst loading in the transition metal catalyzed carborane B–H functionalization to the best of our knowledge (Table 1, entry 18). The molecular structures of **3ba** and **4ba** were further confirmed by single-crystal X-ray analyses.

**Table 1:**
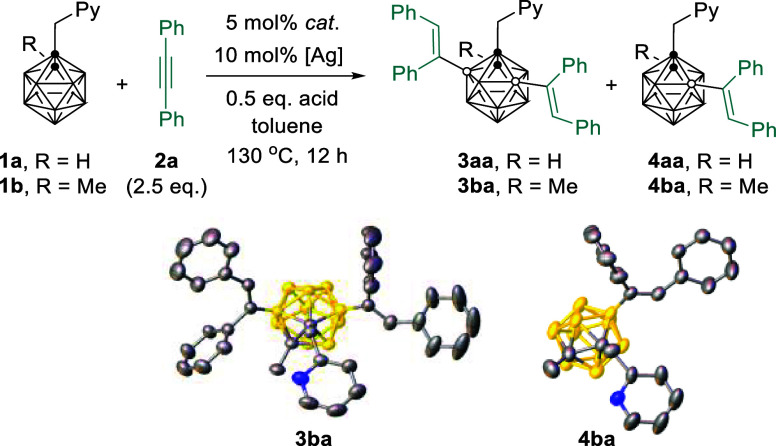
Optimization of Reaction Conditions^a^

				yield (%)^b^	
entry	R	cat.	[Ag]	**3**	**4**
1	H	Pd(PPh_3_)_4_	AgNTf_2_	N.R.	
2	H	Pd(OAc)_2_	AgNTf_2_	66	4
3	H	PdCl_2_(cod)	AgNTf_2_	68	2
4	H	PdCl_2_(PPh_3_)_2_	AgNTf_2_	56	2
5	H	PdCl_2_(PCy_3_)_2_	AgNTf_2_	80	2
6	H	PdCl_2_	AgNTf_2_	93	2
7	H	PdCl_2_	–	messy	
8	H	PdCl_2_	AgTFA	27	7
9	H	PdCl_2_	AgOTf	67	5
10	H	PdCl_2_	AgBF_4_	90	4
11^c^	H	PdCl_2_	AgNTf_2_	72	4
12^d^	H	PdCl_2_	AgNTf_2_	92	2
13^e^	H	PdCl_2_	AgNTf_2_	83	10
14^f^^,^^g^	H	PdCl_2_	AgNTf_2_	13	68
15^d^	Me	PdCl_2_	AgNTf_2_	67	24
16^d^^,^^h^	Me	PdCl_2_	AgNTf_2_	67	23
17^d^^,^^g^	Me	PdCl_2_	AgNTf_2_	-	94
18^f^^,^^g^	Me	PdCl_2_	AgNTf_2_	-	92

a All reactions were carried out on 0.2 mmol scale in 3 mL of toluene; crystal structures drawn at the 50% probability level.

b ^1^H NMR yield using 1,1,2,2-tetrachloroethane as the internal standard.

c Without HOAc.

d 2.5 mmol % PdCl_2_, 5 mol % AgNTf_2_.

e 1.5 mol % PdCl_2_, 3 mol % AgNTf_2._

f 1.0 mol % PdCl_2_, 2 mol % AgNTf_2_, 16 h.

g 1.0 equiv of **2a**.

h 4 equiv of **2a**.

With the optimal reaction conditions in hand (Table 1, entry 12), the substrate generality for the B(3,5)–H dialkenylation of *o*-carborane was evaluated, and the results were compiled in Table 2. Treatment of **1a** with 2.5 equiv of diphenylacetylene **2a** gave the desired product **3aa** in 87% isolated yield. Electronic effects on the reactions were not obvious. Diphenyl acetylenes with a variety of *para*-electron-donating units worked very well, affording **3ab**–**3af** in 88–91% yields. Those with *para*-electron-withdrawing groups such as −Ph, −F, and −CF_3_ gave **3ag**, **3ah**, and **3aj** in 80–89% yields. It was noted that *p*-Cl was a poor substrate since it might be reactive toward Pd. However, *meta*-substituted diphenyl acetylenes afforded the corresponding products **3ak**–**3ao** in lower yields of 52–87%. Di-*o*-tolylacetylene was not compatible due to steric factors. 3-Thienyl acetylenes offered **3aq** in 54% yields, whereas 2-thienyl was not compatible with this reaction. These results suggested that steric factors played a role in the reactions. Unsymmetrical alkyne MeC≡CPh and dialkyl acetylene ^*n*^BuC≡CBu^*n*^ gave a mixture of inseparable products.

**Table 2:**
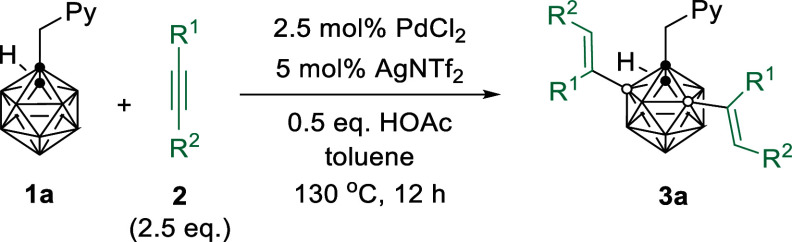

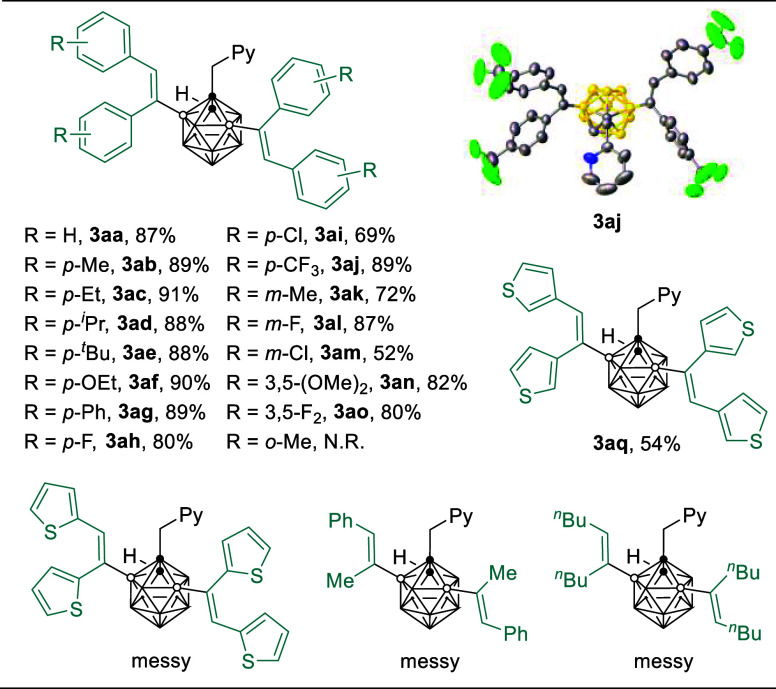
B(3,5)-Dialkenylation of 1-(2′-Picolyl)-*o*-carborane^a^

a All reactions were carried out on 0.2 mmol scale in 3 mL of toluene; crystal structure drawn at the 50% probability level.

As the cage C(2) substituent has a significant impact on the degree of functionalization and regioselectivity (Table 1), the mono B(4)-alkenylation of cage C(2) substituted *o*-carboranes was also examined as illustrated in Table 3. Similar to the B(3,5)-dialkenylation of **1a**, a series diphenylacetylenes with different *para*- or *meta*-substituents reacted with **1b** to afford **4ba**–**4bo** in 67–93% isolated yields with only 1 mol % PdCl_2_ catalyst loading. Both 2- and 3-thienyl acetylenes were compatible with monoalkenylation reaction, giving **4bq** and **4br** in 69% and 68% yields, respectively. Unsymmetrical alkynes such as MeC≡CPh and ^*n*^BuC≡CPh gave only one alkyne insertion regioisomer. Dialkyl acetylene ^*n*^BuC≡CBu^*n*^ was also tolerated with an 85% yield (**4bu**). Alkyl and aryl substituents R on cage C(2) had no impact on the reaction efficiency, giving **4ca**–**4ha** in very high yields. These results indicated that steric effects in monoalkenylation reactions were not obvious.

**Table 3:**
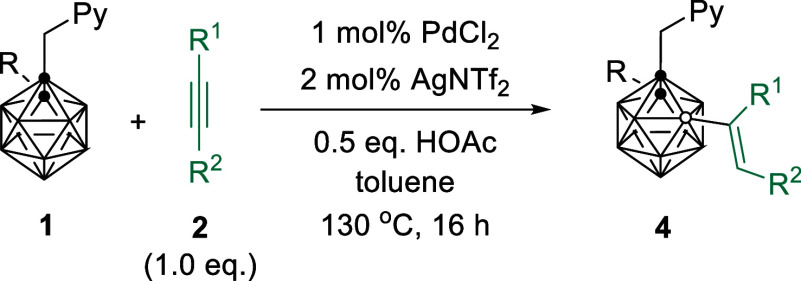

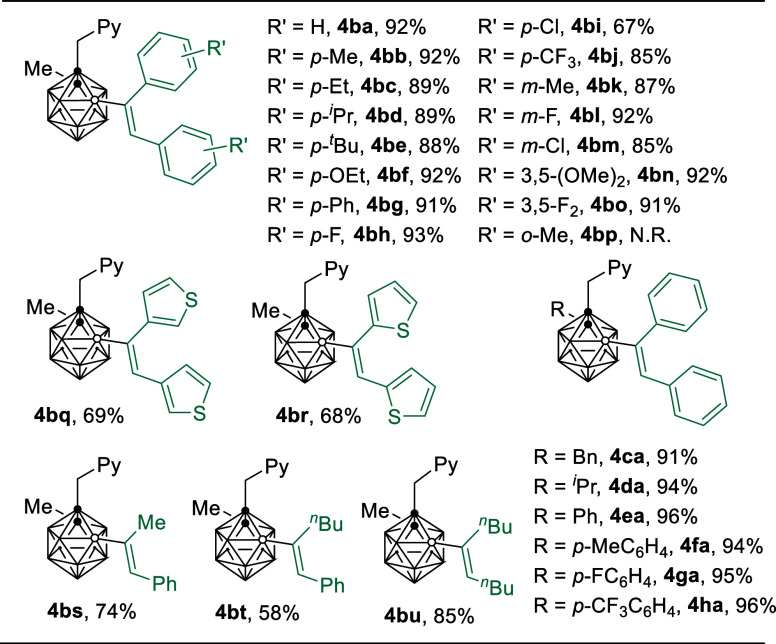
B(4)-Alkenylation of 1-(2′-Picolyl)-*o*-carboranes^a^

a All reactions were carried out on 0.5 mmol scale in 7.5 mL of toluene.

**Scheme 2:**
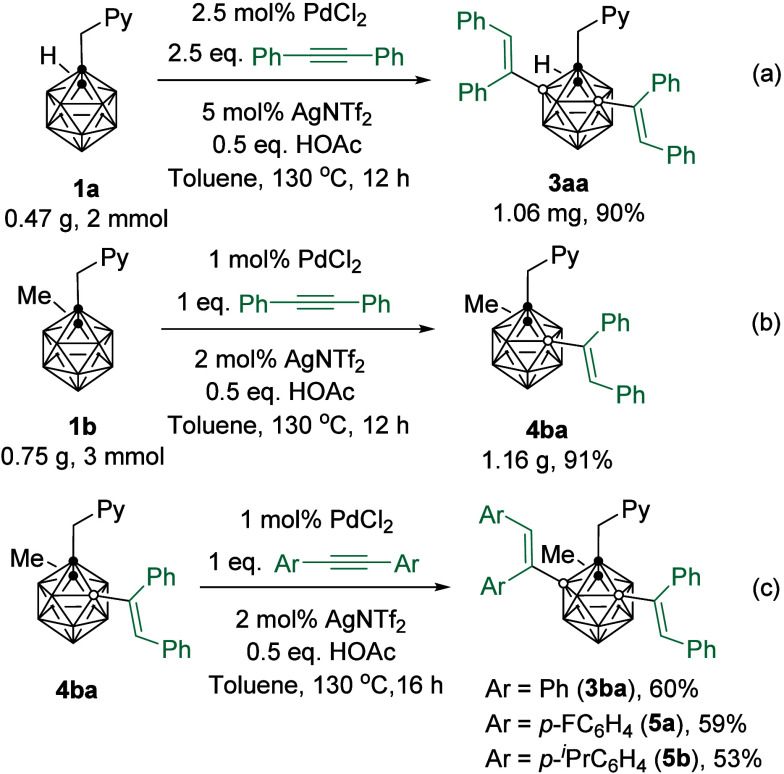
Scale-up Synthesis of 3aa/4ba and B(3,5)-Dialkenylation with Different Alkynes

Scaled-up syntheses of **3aa** and **4ba** were subsequently carried out under standard reaction conditions to afford the B(3,5)-dialkenylation and B(4)-monoalkenylation products in about 90% isolated yields, respectively (Scheme 2a and 2b). Compound **4ba** could be further alkenylated with a second equivalent of diarylacetylenes to give B(3,5)-dialkenylation products **3ba**, **5a** and **5b** in 53–60% isolated yields (Scheme 2c).

**Scheme 3:**
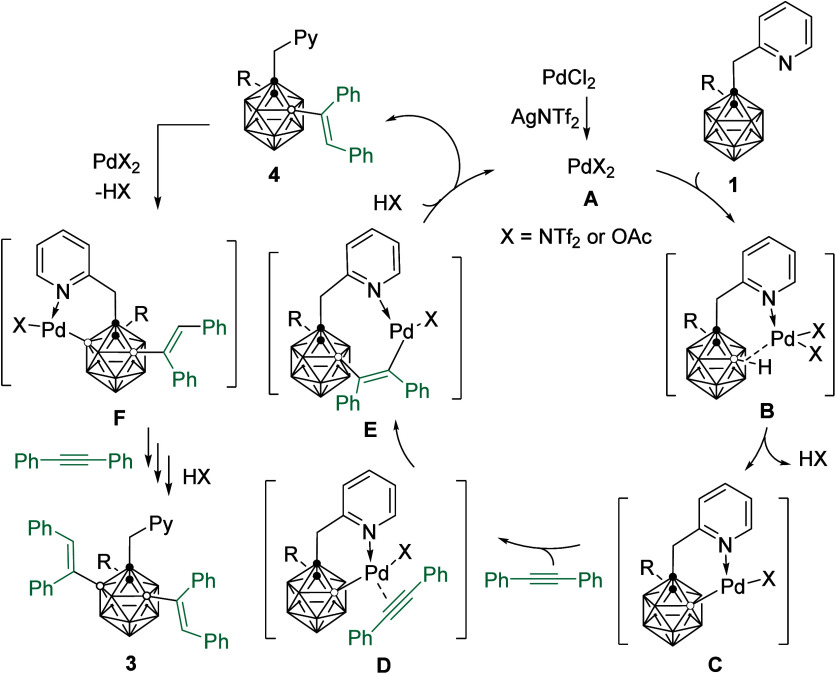
Proposed Mechanism

According to literature reports and our previous publications,^7,16^ a plausible catalytic cycle is proposed to account for the formation of the products (Scheme 3). The reaction is initiated by the salt metathesis reaction of PdCl_2_ with AgNTf_2_, giving an active Pd(II) species **A**. The picolyl-directed regioselective electrophilic palladation on the B(4)–H [= B(5)-H] in *o*-carborane affords a Pd–B intermediate **C**. Coordination and subsequent insertion of alkyne lead to the formation of an intermediate **E** that undergoes protonation to give the monoalkenylated product **4**. The presence of the cage B(4)- or B(5)-alkenyl group blocks both B(3,5)- or B(4,6)-H sites, and the remaining B(6) or B(3)–H is then activated by Pd catalyst to afford B(4,6)- or B(3,5)-dialkenylated product **3**.

## Conclusions

In summary, a picolyl directing group assisted palladium catalyzed efficient B(3,5)-dialkenylation or B(4)-monoalkenylation of 1-(2′-picolyl)-*o*-carboranes with internal alkynes has been developed with a very low catalyst loading of 2.5 mol % or 1 mol %. The results suggested that cage C(2) substituent has a significant impact on the degree of functionalization. For the unsubstituted C(2)–H vertex, B(3,5)-dialkenylation was observed, whereas the cage C(2) substituted *o*-carboranes led to B(4)-monoalkenylation due to steric reasons. No obvious electronic effects were found in these reactions. The aforementioned alkenylation process was compatible with a broad substrate scope in high efficiency. This work provides valuable references on the control of different regioselectivity for the functionalization of *o*-carboranes.

## Data Availability

The data underlying this study are available in the published article and its Supporting Information.

## Supporting Information Available

Detailed experimental procedures, characterization data, X-ray crystallographic data, and copies of NMR spectra (PDF)
