# Supplementary material for: Palladium-Catalyzed Regioselective B(3,5)-Dialkenylation and B(4)-Alkenylation of o-Carboranes
Source: J Org Chem. 2024 Feb 2;89(4):2474–9. doi: 10.1021/acs.joc.3c02496 (PMC11526369; doi:10.1021/acs.joc.3c02496)
Supplement: Supplementary file 1 — jo3c02496_si_001.pdf [file jo3c02496_si_001.pdf]

Supporting Information

Palladium-Catalyzed Regioselective B(3,5)-Dialkenylation and B(4)-Alkenylation of *o*-Carboranes

Shasha Yuan,[†,‡] Huifang Zhang,[‡] Zaozao Qiu*,[‡,§] and Zuowei Xie*,[‡,||,⊥]

[†]*School of Materials and Chemistry, University of Shanghai for Science and Technology, Shanghai, China.*

[‡]*Shanghai-Hong Kong Joint Laboratory in Chemical Synthesis, Shanghai Institute of Organic Chemistry, University of Chinese Academy of Sciences, Chinese Academy of Sciences, 345 Lingling Rd, Shanghai 200032, China.*

[§]*Innovation Institute of Carbon Neutrality and International Joint Laboratory of Catalytic Chemistry, Department of Chemistry, College of Sciences, Shanghai University, Shanghai 200444, China.*

[||]*Department of Chemistry, The Chinese University of Hong Kong, Shatin, N. T., Hong Kong, China.*

[⊥]*Shenzhen Grubbs Institute and Department of Chemistry, Southern University of Science and Technology, Shenzhen 518055, China.*

E-mail: qiuzz@sioc.ac.cn; zxie@cuhk.edu.hk

Table of Contents

General Information S2

Experimental Procedures S2

Characterization Data S4

Crystal Data and Summary of Data Collection and Refinements S37

References S39

NMR Spectra S40

General Information

All reactions were carried out in oven-dried glassware under an atmosphere of dry N_2 with the rigid exclusion of air and moisture using standard Schlenk techniques or in a glovebox. Diethyl ether and toluene were purified by solvent purification system prior to use. 1-(2-Picolyl)-*o*-carboranes (**1**)[1,2] and alkynes (**2**)[3] were prepared according to literature procedures. All other chemicals were purchased from either Aldrich or J&K Chemical Co. and used as received unless otherwise specified. ^{1}H NMR spectra were recorded on a Bruker/Agilent/Varian 400 spectrometer at 400 MHz. ^{11}B NMR spectra were recorded on a Bruker 400 spectrometer at 128 MHz. $^{13}C\{^{1}H\}$ and ^{19}F NMR spectra were recorded on a Bruker/Varian 400/600 spectrometer at 101/150 and 376/565 MHz, respectively. All signals were reported in ppm unit with references to the residual solvent resonances of the deuterated solvents for proton and carbon chemical shifts, to external $BF_3·OEt_2$ (0.00) for boron chemical shifts and to external $CFCl_3$ (0.00) for fluorine chemical shifts. Mass spectra were obtained on a Thermo Fisher Scientific LTQ FTICR-MS/Thermo Scientific Q Exactive HF Orbitrap-FTMS/JEOL AccuTOF-MS spectrometer. The melting points of the solid compounds were determined by the melting point apparatus (Shanghai INESA Physico-Optical Instrument Co., LTD).

Experimental Procedures

General procedure for the synthesis of starting materials 1a-1h (GP-1). *o*-Carborane/1-substituted-*o*-carborane (10.0 mmol) was dissolved in Et_2O (20 mL) and cooled to 0 °C, to which was slowly added ^{n}BuLi (11.0 mmol, 2.5 M in hexane, 4.4

mL). The resulting solution was stirred for 2 h at 0 ºC. Then 2-picolyl chloride (1.91 g, 15.0 mmol) was added at 0 ºC. The resulting solution was stirred at 40 ºC (oil bath) overnight. After hydrolysis with water (20 mL) and extraction with diethyl ether (20 mL x 3), the ether solutions were combined and concentrated to dryness in vacuo. The residue was subjected to flash column chromatography on silica gel (200-300 mesh) using *n*-hexane and ethyl acetate (4/1 in v/v) as eluent to give the products **1**.

General procedure for the synthesis of 3 (GP-2).

An oven-dried Schlenk flask equipped with a stir bar was charged with 1-(2-picolyl)-*o*-carborane (**1**) (0.2 mmol), alkyne (**2**) (0.5 mmol), $PdCl_2$ (0.9 mg, 0.005 mmol), $AgNTf_2$ (3.9 mg, 0.01 mmol) and HOAc (6.0 mg, 0.1 mmol), followed by dry toluene (3 mL). The flask was closed under an atmosphere of nitrogen and stirred at 130 ^{o}C (oil bath) for 12 h. After the addition of water (5 mL) and extraction with diethyl ether (5 mL x 3), the ether solutions were combined and concentrated to dryness in vacuo. The residue was subjected to flash column chromatography on silica gel (200-300 mesh) using *n*-hexane and ethyl acetate (10/1 in v/v) as eluent to give product **3**.

General procedure for the synthesis of 4 (GP-3).

An oven-dried Schlenk flask equipped with a stir bar was charged with 1-(2-picolyl)-*o*-carborane (**1**) (0.5 mmol), alkyne (**2**) (0.5 mmol), $PdCl_2$ (0.9 mg, 0.005 mmol), $AgNTf_2$ (3.9 mg, 0.01 mmol) and HOAc (15.0 mg, 0.25 mmol), followed by dry toluene (7.5 mL). The flask was closed under an atmosphere of nitrogen and stirred at 130 ^{o}C (oil bath) for 16 h. After the addition of water (5 mL) and extraction with diethyl ether (5 mL x 3), the ether solutions were combined and concentrated to dryness in vacuo.

The residue was subjected to flash column chromatography on silica gel (200-300 mesh) using *n*-hexane and ethyl acetate (10/1 in v/v) as eluent to give product **4**.

General procedure for the synthesis of 3ba and 5 (GP-4).

An oven-dried Schlenk flask equipped with a stir bar was charged with **4ba** (214.7 mg, 0.5 mmol), alkyne (**2**) (0.5 mmol), $PdCl_2$ (0.9 mg, 0.005 mmol), $AgNTf_2$ (3.9 mg, 0.01 mmol) and HOAc (15.0 mg, 0.1 mmol), followed by dry toluene (7.5 mL). The flask was closed under an atmosphere of nitrogen and stirred at 130 °C (oil bath) for 16 h. After the addition of water (5 mL) and extraction with diethyl ether (5 mL x 3), the ether solutions were combined and concentrated to dryness in vacuo. The residue was subjected to flash column chromatography on silica gel (200-300 mesh) using *n*-hexane and ethyl acetate (10/1 in v/v) as eluent to give product **3ba** or **5**.

Characterization Data

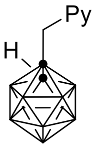

1-(2-Picolyl)-*o*-carborane (1a) was prepared as a white solid, following the general procedure (GP-1) and purified by column chromatography on silica gel (200-300 mesh) with an eluent (*n*-hexane/ethyl acetate = 4/1) (1.79 g, 76%). ^1H NMR (400 MHz, $CDCl_3$): δ 8.55 (d, J = 4.0 Hz, 1H), 7.70 (m, 1H), 7.26 (m, 1H), 7.17 (d, J = 7.6 Hz, 1H), 4.09 (s, 1H), 3.66 (s, 2H). These data were the same as those reported in literature.[2]

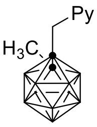

1-(2-Picolyl)-2-methyl-*o*-carborane (1b) was prepared as a white solid, following the general procedure (GP-1) and purified by column chromatography on silica gel (200-300 mesh) with an eluent (*n*-hexane/ethyl acetate =

4/1) (2.04 g, 82%). ^{1}H NMR (400 MHz, $CDCl_3$): δ 8.56 (d, J = 4.4 Hz, 1H) , 7.70 (td, J = 7.6, 1.6 Hz, 1H), 7.25 (m, 2H), 3.69 (s, 2H), 2.26 (s, 3H). These data were the same as those reported in literature.[2]

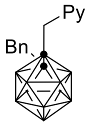

1-(2-Picolyl)-2-benzyl-*o*-carborane (1c) was prepared as a white solid, following the general procedure (GP-1) and purified by column chromatography on silica gel (200-300 mesh) with an eluent (*n*-hexane/ethyl acetate = 4/1) (2.38 g, 73%). ^{1}H NMR (400 MHz, $CDCl_3$): δ 8.60 (d, J = 4.4 Hz, 1H), 7.72 (td, J = 7.6, 1.6 Hz, 1H), 7.29 (m, 7H), 3.90 (s, 2H), 3.86 (s, 2H). These data were the same as those reported in literature.[2]

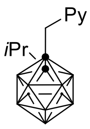

1-(2-Picolyl)-2-*iso*-propyl-*o*-carborane (1d) was prepared as a white solid, following the general procedure (GP-1) and purified by column chromatography on silica gel (200-300 mesh) with an eluent (*n*-hexane/ethyl acetate = 4/1) (2.28 g, 82%). ^{1}H NMR (400 MHz, $CDCl_3$): δ 8.54 (d, J = 4.4 Hz, 1H), 7.67 (td, J = 7.6, 1.6 Hz, 1H), 7.23 (m, 2H), 3.66 (s, 2H), 2.92 (m, 1H), 1.24 (d, J = 6.8 Hz, 6H). These data were the same as those reported in literature.[2]

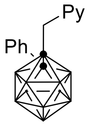

1-(2-Picolyl)-2-phenyl-*o*-carborane (1e) was prepared as a white solid, following the general procedure (GP-1) and purified by column chromatography on silica gel (200-300 mesh) with an eluent (*n*-hexane/ethyl acetate = 4/1) (2.81 g, 90%). ^{1}H NMR (400 MHz, $CDCl_3$): δ 8.53 (d, J = 4.8 Hz, 1H), 7.75 (m, 2H) , 7.57 (td, J = 7.6, 2.0 Hz, 1H), 7.50 (m, 1H), 7.44 (m, 2H), 7.18 (m, 1H), 6.87 (d, J = 7.6 Hz, 1H), 3.23 (s, 2H). These data were the same as those reported in literature.[2]

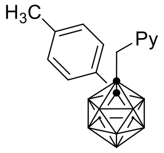

1-(2-Picolyl)-2-(4-methylphenyl)-*o*-carborane (1f) was prepared as a white solid, following the general procedure (GP-1) and purified by column chromatography on silica gel (200-300 mesh) with an eluent (*n*-hexane/ethyl acetate = 4/1) (2.48 g, 76%). Mp: 119.3-119.8 °C. ^{1}H NMR (400 MHz, $CDCl_3$): δ 8.55 (d, J = 2.8 Hz, 1H), 7.63 (d, J = 7.6 Hz, 2H), 7.58 (t, J = 7.2 Hz, 1H), 7.25 (d, J = 7.6 Hz, 2H), 7.18 (t, J = 6.0 Hz, 1H), 6.90 (d, J = 7.6 Hz, 1H), 3.25 (s, 2H), 2.40 (s, 3H). ^{13}C{^{1}H} NMR ($CDCl_3$, 101 MHz): δ 155.0, 149.5, 141.3, 136.4, 131.3, 129.7, 124.8, 124.7, 122.6, 84.0, 80.7, 43.0, 21.1. ^{11}B NMR ($CDCl_3$, 128 MHz): δ -3.5 (d, J = 128.0 Hz, 2B), -10.1 (d, J = 125.4 Hz, 8B). HRMS (DART) Calcd for $C_{15}H_{24}{}^{10}B_2{}^{11}B_8N^+$ [M+H$^+$]: 326.2906, Found: 326.2904.

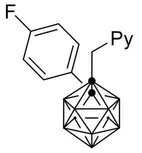

1-(2-Picolyl)-2-(4-fluorophenyl)-*o*-carborane (1g) was prepared as a white solid, following the general procedure (GP-1) and purified by column chromatography on silica gel (200-300 mesh) with an eluent (*n*-hexane/ethyl acetate = 4/1) (1.65 g, 50%). Mp: 97.0-97.8 °C. ^{1}H NMR (400 MHz, $CDCl_3$): δ 8.48 (d, J = 4.4 Hz, 1H), 7.62 (m, 2H), 7.53 (t, J = 7.6 Hz, 1H), 7.12 (m, 1H), 7.06 (t, J = 8.4 Hz, 2H), 6.88 (d, J = 7.6 Hz, 1H), 3.20 (s, 2H). ^{13}C{^{1}H} NMR ($CDCl_3$, 101 MHz): δ 164.2 (d, $^1J_{CF}$ = 253.5 Hz), 154.9, 149.7, 136.6, 133.7 (d, $^3J_{CF}$ = 9.1 Hz), 126.9 (d, $^4J_{CF}$ = 4.0 Hz), 124.8, 122.9, 116.2 (d, $^2J_{CF}$ = 22.2 Hz), 82.9, 80.8, 43.2. ^{11}B NMR ($CDCl_3$, 128 MHz): δ -3.4 (d, J = 145.9 Hz, 2B), -9.9 (m, 8B). ^{19}F NMR ($CDCl_3$, 376 MHz): δ -109.2 (m, 1F). HRMS (DART) Calcd for $C_{14}H_{21}{}^{10}B_2{}^{11}B_8FN^+$ [M+H$^+$]: 330.2656, Found: 330.2654.

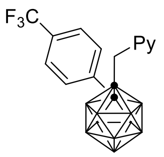

1-(2-Picolyl)-2-(4-(trifluoromethyl)phenyl)-*o*-carborane (1h) was prepared as a yellow solid following the general procedure (GP-1) and purified by column chromatography on silica gel (200-300 mesh) with an eluent (*n*-hexane/ethyl acetate = 4/1) (2.81 g, 74%). Mp: 81.3-81.9 °C. ^{1}H NMR (400 MHz, $CDCl_3$): δ 8.52 (d, J = 4.8 Hz, 1H), 7.90 (d, J = 8.4 Hz, 2H), 7.71 (d, J = 8.0 Hz, 2H), 7.60 (td, J = 3.6, 1.6 Hz, 1H), 7.20 (m, 1H), 6.97 (d, J = 7.6 Hz, 1H), 3.23 (s, 2H). ^{13}C{^{1}H} NMR ($CDCl_3$, 101 MHz): δ 153.7, 148.8, 135.7, 133.6, 131.9 (q, $^{2}J_{CF}$ = 32.9 Hz), 131.2, 125.1 (q, $^{3}J_{CF}$ = 3.7 Hz), 123.8, 122.6 (q, $^{1}J_{CF}$ = 271.8 Hz), 122.0, 121.2. ^{11}B NMR ($CDCl_3$, 128 MHz): δ -3.5 (d, J = 128.0 Hz, 2B), -10.1 (m, 8B). ^{19}F NMR ($CDCl_3$, 376 MHz): δ -63.1 (s, 3F). HRMS (DART) Calcd for $C_{15}H_{21}{}^{10}B_2{}^{11}B_8F_3N^+$ [M+H$^+$]: 380.2624, Found: 380.2621.

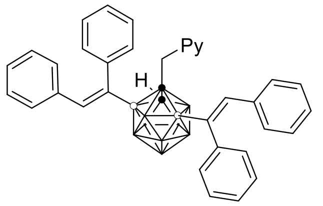

1-(2-Picolyl)-3,5-bis(*cis*-1,2-diphenylvinyl)-*o*-carborane (3aa) was prepared as a white solid, following the general procedure (GP-2) and purified by column chromatography on silica gel (200-300 mesh) with an eluent (*n*-hexane/ethyl acetate = 10/1) (103.1 mg, 87%). Mp: 136.6-137.2 °C. ^{1}H NMR (400 MHz, $CDCl_3$): δ 8.51 (d, J = 4.8 Hz, 1H), 7.57 (td, J = 7.6, 1.6 Hz, 1H), 7.37 (m, 4H), 7.30 (m, 7H), 7.21 (t, J = 5.6 Hz, 1H), 7.15 (m, 6H), 7.06 (s, 1H), 7.00 (m, 4H), 6.90 (d, J = 8.0 Hz, 1H), 4.90 (s, 1H), 4.05 (d, J = 15.2 Hz, 1H), 3.72 (d, J = 15.2 Hz, 1H). ^{13}C{^{1}H} NMR ($CDCl_3$, 101 MHz): δ 156.0, 149.4, 143.3, 142.2, 141.7, 137.6, 137.0, 136.8, 129.9, 129.7, 128.9, 128.9, 128.8, 128.7, 128.1, 127.7, 127.2, 126.7, 126.4, 124.9, 122.6, 74.7, 57.7, 38.1. ^{11}B NMR ($CDCl_3$, 128

MHz): δ -0.1 (s, 1B), -2.9 (m, 3B), -9.6 (m, 1B), -12.2 (m, 3B), -14.2 (m, 2B). HRMS (ESI) Calcd for $C_{36}H_{38}{}^{10}B_2{}^{11}B_8N^+$ [M+H$^+$]:592.4002, Found: 592.4015.

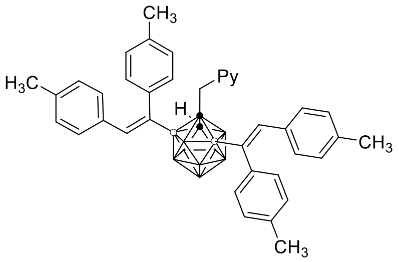

1-(2-Picolyl)-3,5-bis(*cis*-1,2-di(4-methylphenyl)vinyl)-*o*-carborane (3ab) was prepared as a white solid, following the general procedure (GP-2) and purified by column chromatography on silica gel (200-300 mesh) with an eluent (*n*-hexane/ethyl acetate = 10/1) (115.6 mg, 89%). Mp: 105.1-105.8 °C. ^{1}H NMR (400 MHz, $CDCl_3$): δ 8.48 (d, *J* = 3.6 Hz, 1H), 7.53 (t, *J* = 7.6 Hz, 1H), 7.18 (m, 10H), 6.92 (m, 10H), 4.81(s, 1H), 3.99 (d, *J* = 15.2 Hz, 1H), 3.67 (d, *J* = 15.2 Hz, 1H), 2.41(s, 6H), 2.29 (s, 3H), 2.26 (s, 3H). $^{13}C\{^1H\}$ NMR ($CDCl_3$, 101 MHz): δ 156.3, 149.3, 141.9, 141.3, 140.5, 139.0, 137.5, 137.0, 136.6, 136.1, 135.8, 135.0, 134.3, 129.8, 129.64, 129.6, 129.4, 128.8, 128.75, 128.71, 124.9, 122.5, 74.7, 57.7, 38.2, 21.4, 21.3, 21.3. ^{11}B NMR ($CDCl_3$, 128 MHz): δ -0.3 (s, 1B), -2.9 (m, 3B), -9.9 (m, 1B), -11.6 (m, 2B), -14.2 (m, 3B). HRMS (ESI) Calcd for $C_{40}H_{45}{}^{10}B_2{}^{11}B_8N^+$ [M$^+$]: 649.4620, Found: 649.4619.

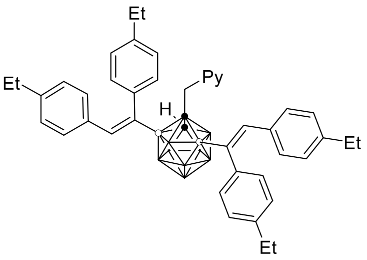

1-(2-Picolyl)-3,5-bis(*cis*-1,2-di(4-ethylphenyl)vinyl)-*o*-carborane (3ac) was prepared as a white solid, following the general procedure (GP-2) and purified by column chromatography on silica gel (200-300 mesh) with an eluent (*n*-hexane/ethyl acetate = 10/1) (128.4 mg, 91%). Mp: 84.3-85.3 °C. ^{1}H NMR (400 MHz, $CDCl_3$): δ 8.48 (d, *J* = 4.4 Hz, 1H), 7.53 (t, *J* = 7.2 Hz, 1H), 7.23 (m, 10H), 6.98 (m, 7H), 6.88 (m, 3H), 4.83 (s, 1H), 4.01 (d, *J* = 14.8

Hz, 1H), 3.69 (d, J = 15.2 Hz, 1H), 2.72 (q, J = 7.6 Hz, 4H), 2.58 (m, 4H), 1.32 (t, J = 7.6 Hz, 6H), 1.26 (m, 6H). $^{13}C\{^1H\}$ NMR ($CDCl_3$, 101 MHz): δ 156.2, 149.2, 143.9, 143.3, 142.5, 142.2, 141.9, 141.4, 140.7, 139.2, 136.6, 135.2, 134.6, 129.9, 129.7, 128.8, 128.7, 128.4, 128.2, 127.6, 127.5, 124.9, 122.5, 74.6, 57.6, 38.1, 28.7, 28.6, 15.8, 15.7, 15.5, 15.4. ^{11}B NMR ($CDCl_3$, 128 MHz): δ -0.8 (s, 1H), -3.1 (m, 3B), -11.5 (m, 6B). HRMS (ESI) Calcd for $C_{44}H_{53}{}^{10}B_2{}^{11}B_8N^+$ [M^+]: 705.5249, Found: 705.5238.

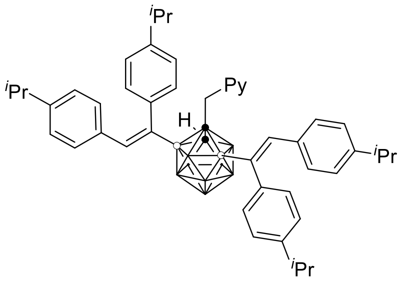

1-(2-Picolyl)-3,5-bis(*cis*-1,2-di(4-*iso*-propylphenyl)vinyl)-*o*-carborane (3ad) was prepared as a white solid, following the general procedure (GP-2) and purified by column chromatography on silica gel (200-300 mesh) with an eluent (*n*-hexane/ethyl acetate = 10/1) (134.0 mg, 88%). Mp: 109.6–110.4 °C. ^1H NMR (400 MHz, $CDCl_3$): δ 8.48 (d, J = 4.0 Hz, 1H), 7.50 (td, J = 7.6, 1.6 Hz, 1H), 7.17 (m, 10H), 6.98 (m, 5H), 6.89 (m, 4H), 6.82 (d, J = 7.6 Hz, 1H), 4.80 (s, 1H), 3.99 (d, J = 15.2 Hz, 1H), 3.68 (d, J = 15.2 Hz, 1H), 2.96 (m, 2H), 2.82 (m, 2H), 1.32 (d, J = 6.8 Hz, 12H), 1.21 (d, J = 6.8, 6H), 1.18 (d, J = 6.8, 6H). $^{13}C\{^1H\}$ NMR ($CDCl_3$, 101 MHz): δ 156.3, 149.2, 148.5, 147.9, 147.2, 146.9, 141.9, 141.4, 140.9, 139.4, 136.6, 135.3, 134.8, 130.0, 129.8, 128.7, 128.6, 126.9, 126.7, 126.2, 126.1, 124.9, 122.4, 74.6, 57.6, 38.0, 33.9, 33.8, 24.3, 24.2, 24.3, 24.0, 23.9, 23.9. ^{11}B NMR ($CDCl_3$, 128 MHz): δ -3.3 (m, 4B), -11.4 (m, 6B). HRMS (ESI) Calcd for $C_{48}H_{61}{}^{10}B_2{}^{11}B_8N^+$ [M^+]: 761.5877, Found: 761.5874.

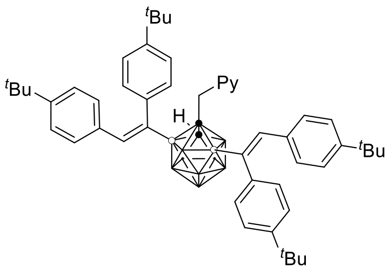

1-(2-Picolyl)-3,5-bis(*cis*-1,2-di(4-*tert*-butylphenyl)vinyl)-*o*-carborane (3ae) was prepared as a white solid, following the general procedure (GP-2) and purified by column chromatography on silica gel (200-300 mesh) with an eluent (*n*-hexane/ethyl acetate = 10/1) (143.9 mg, 88%). Mp: 114.2-114.9 °C. ^{1}H NMR (400 MHz, $CDCl_3$): δ 8.46 (d, J = 4.8 Hz, 1H), 7.51 (td, J = 7.6, 1.6 Hz, 1H), 7.38 (m, 4H), 7.19 (m, 10H), 6.92 (m, 5H), 6.83 (d, J = 8.0 Hz, 1H), 4.81 (s, 1H), 4.00 (d, J = 15.2 Hz, 1H), 3.69 (d, J = 15.2 Hz, 1H), 1.40 (s, 18H), 1.29 (s, 9H), 1.26 (s, 9H). $^{13}C\{^1H\}$ NMR ($CDCl_3$, 101 MHz): δ 156.3, 150.8, 150.2, 149.5, 149.2, 141.8, 141.3, 140.5, 139.0, 136.6, 134.9, 134.4, 129.7, 129.5, 128.4, 128.3, 125.8, 125.5, 125.0, 124.9, 122.4, 74.6, 57.6, 38.0, 34.7, 34.6, 31.7, 31.65, 31.4, 31.3. ^{11}B NMR ($CDCl_3$, 128 MHz): δ -3.4 (m, 4B), -11.5 (m, 6B). HRMS (ESI) Calcd for $C_{52}H_{69}{}^{10}B_2{}^{11}B_8N^+$ [M^+]: 817.6506, Found: 817.6513.

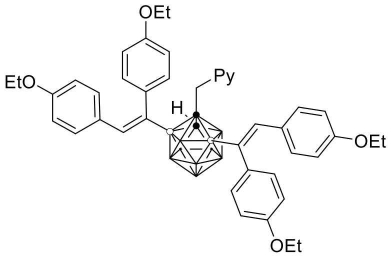

1-(2-Picolyl)-3,5-bis(*cis*-1,2-di(4-ethoxylphenyl)vinyl)-*o*-carborane (3af) was prepared as a white solid, following the general procedure (GP-2) and purified by column chromatography on silica gel (200-300 mesh) with an eluent (*n*-hexane/ethyl acetate = 10/1) (138.3 mg, 90%). Mp: 101.7-102.2 °C. ^{1}H NMR (400 MHz, $CDCl_3$): δ 8.46 (d, J = 4.0 Hz, 1H), 7.52 (t, J = 7.6 Hz, 1H), 7.16 (m, 6H), 6.90 (m, 10H), 6.65 (m, 4H), 4.75 (s, 1H), 4.07 (q, J = 7.2 Hz, 4H), 3.96 (m, 5H), 3.63 (d, J = 15.2 Hz, 1H), 1.46 (t, J = 6.8 Hz, 6H), 1.37 (m, 6H). $^{13}C\{^1H\}$ NMR ($CDCl_3$, 101 MHz): δ 158.3, 158.0, 157.7,

157.5, 156.3, 149.3, 141.7, 136.6, 135.7, 134.2, 131.3, 131.0, 130.4, 130.0, 129.9, 124.9, 122.5, 115.0, 114.9, 114.7, 114.0, 74.6, 63.4, 63.3, 57.7, 38.1, 15.1, 15.0, 14.9. ^{11}B NMR ($CDCl_3$, 128 MHz): δ -3.0 (m, 4B), -11.5 (m, 6B). HRMS (ESI) Calcd for $C_{44}H_{54}O_4{}^{10}B_2{}^{11}B_8N^+$ [M+H$^+$]: 768.5051, Found: 768.5073.

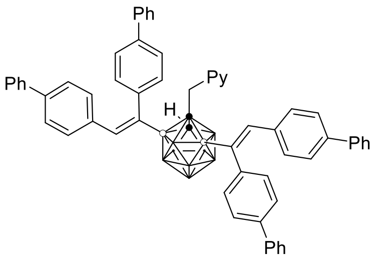

1-(2-Picolyl)-3,5-bis(*cis*-1,2-di(4-biphenyl)vinyl)-*o*-carborane (3ag) was prepared as a white solid, following the general procedure (GP-2) and purified by column chromatography on silica gel (200-300 mesh) with an eluent (*n*-hexane/ethyl acetate = 10/1) (159.6 mg, 89%). Mp: 144.3-144.9 °C. ^{1}H NMR (400 MHz, $CDCl_3$): δ 8.44 (d, J = 5.2 Hz, 1H), 7.67 (m, 3H), 7.61 (m, 4H), 7.54 (m, 2H), 7.50 (m, 4H), 7.45 (m, 3H), 7.37 (m, 15H), 7.29 (m, 2H), 7.25 (s, 1H), 7.14 (m, 1H), 7.08 (m, 5H), 6.84 (d, J = 8.0 Hz, 1H), 4.89 (s, 1H), 4.01 (d, J = 15.2 Hz, 1H), 3.69 (d, J = 15.6 Hz, 1H). ^{13}C{^{1}H} NMR ($CDCl_3$, 101 MHz): δ 155.6, 149.4, 142.5, 142.2, 141.6, 141.0, 140.9, 140.6, 140.5, 140.3, 139.9, 139.4, 139.1, 136.8, 136.7, 136.1, 130.4, 130.3, 129.5, 129.4, 129.0, 128.9, 127.6, 127.5, 127.5, 127.4, 127.2, 127.1, 127.0, 126.9, 126.8, 125.6, 124.9, 122.6, 74.5, 57.7, 38.1. ^{11}B NMR ($CDCl_3$, 128 MHz): δ -3.1 (m, 4B), -11.2 (m, 6B). HRMS (ESI) Calcd for $C_{60}H_{54}{}^{10}B_2{}^{11}B_8N^+$ [M+H$^+$]: 896.5254, Found: 896.5279.

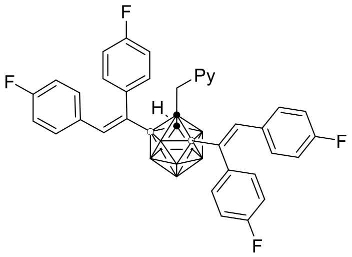

1-(2-Picolyl)-3,5-bis(*cis*-1,2-di(4-fluorophenyl)vinyl)-*o*-carborane (3ah) was prepared as a white solid, following the general procedure (GP-2) and purified by column chromatography on silica gel (200-300 mesh)

with an eluent (n-hexane/ethyl acetate = 10/1) (106.5 mg, 80%). Mp: 84.8–85.5 °C. ^{1}H NMR (400 MHz, $CDCl_3$): δ 8.46 (d, J = 4.0 Hz, 1H), 7.57 (t, J = 7.6 Hz, 1H), 7.18 (m, 5H), 7.04 (t, J = 8.8 Hz, 5H), 6.87 (m, 10H), 4.82 (s, 1H), 3.90 (d, J = 15.6 Hz, 1H), 3.58 (d, J =15.2 Hz, 1H). $^{13}C\{^1H\}$ NMR ($CDCl_3$, 101 MHz): δ 162.1 (d, $^1J_{CF}$ = 250.5 Hz), 162.0 (d, $^1J_{CF}$ = 246.4 Hz), 161.8 (d, $^1J_{CF}$ = 248.5 Hz), 161.7 (d, $^1J_{CF}$ = 246.4 Hz), 155.8, 149.5, 141.6, 141.0, 138.7 (d, $^4J_{CF}$ = 4.0 Hz), 137.2 (d, $^4J_{CF}$ = 3.0 Hz), 136.9, 133.5 (d, $^4J_{CF}$ = 3.0 Hz), 133.0 (d, $^4J_{CF}$ = 3.0 Hz), 131.6 (d, $^3J_{CF}$ = 7.1 Hz), 131.3 (d, $^3J_{CF}$ = 8.1 Hz), 130.5 (d, $^3J_{CF}$ = 8.1 Hz), 130.4 (d, $^3J_{CF}$ = 8.1 Hz), 124.7, 122.8, 116.1 (d, $^2J_{CF}$ = 20.2 Hz), 115.8 (d, $^2J_{CF}$ = 19.2 Hz), 115.3, 115.0, 74.5, 57.7, 38.1. ^{11}B NMR ($CDCl_3$, 128 MHz): δ -2.7 (m, 4B), -11.6 (m, 6B). ^{19}F NMR ($CDCl_3$, 376 MHz): δ -113.0 (m, 1F), -113.7 (m, 1F), -115.6 (m, 1F), -115.9 (m, 1F). HRMS (ESI) Calcd for $C_{36}H_{33}{}^{10}B_2{}^{11}B_8F_4N^+$ [M^+]: 665.3615, Found: 665.3605.

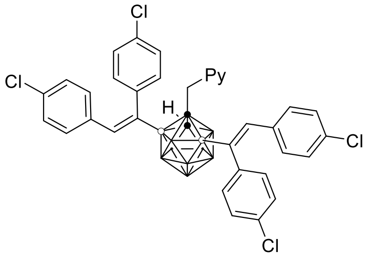

1-(2-Picolyl)-3,5-bis(*cis*-1,2-di(4-chlorophenyl)vinyl)-*o*-carborane (3ai) was prepared as a white solid, following the general procedure (GP-2) and purified by column chromatography on silica gel (200-300 mesh) with an eluent (n-hexane/ethyl acetate = 10/1) (100.9 mg, 69%). Mp: 103.9-104.7 °C. ^{1}H NMR (400 MHz, $CDCl_3$): δ 8.44 (d, J = 5.2 Hz, 1H), 7.55 (t, J = 8.0 Hz, 1H), 7.30 (d, J = 7.6 Hz, 4H), 7.19 (m, 3H), 7.12 (m, 7H), 6.94 (s, 1H), 6.86 (m, 4H), 6.77 (d, J = 8.0 Hz, 1H), 4.81 (s, 1H), 3.85 (d, J = 5.2 Hz, 1H), 3.52 (d, J =15.2 Hz, 1H). $^{13}C\{^1H\}$ NMR ($CDCl_3$, 101 MHz): δ 155.6, 149.5, 141.6, 141.2, 141.1, 139.7, 137.0, 135.6, 135.1, 133.7, 133.3, 133.0, 132.6, 131.0, 130.9, 130.33, 130.29, 129.3,

129.1, 128.5, 128.4, 124.6, 122.9, 74.5, 57.7, 38.1. ^{11}B NMR ($CDCl_3$, 128 MHz): *δ* -2.7 (m, 4B), -11.5 (m, 6B). HRMS (ESI) Calcd for $C_{36}H_{33}{}^{10}B_2{}^{11}B_8Cl_4N^+$ [M^+]: 731.2412, Found: 731.2413.

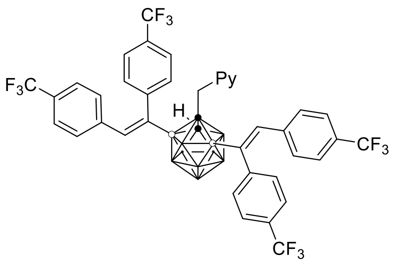

1-(2-Picolyl)-3,5-bis(*cis*-1,2-bis(4-(trifluoromethyl)phenyl)vinyl)-*o*-carborane (3aj) was prepared as a white solid, following the general procedure (GP-2) and purified by column chromatography on silica gel (200-300 mesh) with an eluent (*n*-hexane/ethyl acetate = 10/1) (153.7mg, 89%). Single crystals suitable for X-ray analyses were obtained by slow evaporation of a benzene solution over a period of 3 days at ambient conditions. Mp: 194.6–195.0 °C. ^{1}H NMR (400 MHz, $CDCl_3$): *δ* 8.41 (d, *J* = 3.6 Hz, 1H), 7.60 (d, *J* = 7.6 Hz, 4H), 7.53 (t, *J* = 8.0 Hz, 1H), 7.37 (m, 9H), 7.20 (t, *J* = 7.2 Hz, 1H), 7.09 (s, 1H), 7.02 (m, 4H), 6.68 (d, *J* = 7.6 Hz, 1H), 4.90 (s, 1H), 3.87 (d, *J* = 15.2 Hz, 1H), 3.55 (d, *J* = 15.2 Hz, 1H). $^{13}C\{^1H\}$ NMR ($CDCl_3$, 150 MHz): *δ* 155.3, 149.6, 146.3, 144.9, 141.9, 141.5, 140.4, 139.7, 137.1, 130.1-129.2 (m), 129.9, 129.8, 129.3, 129.2, 129.0 (q, $^2J_{CF}$ = 20.1 Hz), 126.1 (q, $^4J_{CF}$ = 3.0 Hz), 125.9 (q, $^4J_{CF}$ = 3.5 Hz), 125.3 (m), 124.5, 124.3 (q, $^1J_{CF}$ = 271.2 Hz), 124.09 (q, $^1J_{CF}$ = 270.2 Hz), 124.0 (q, $^1J_{CF}$ = 271.2 Hz), 123.1, 74.5, 57.7, 38.0. ^{11}B NMR ($CDCl_3$, 128 MHz): *δ* -2.4 (m, 4B), -11.2 (m, 6B). ^{19}F NMR ($CDCl_3$, 565 MHz): *δ* -62.2 (s, 3F), -62.3 (s, 3F), -62.6 (s, 3F), -62.7 (s, 3F). HRMS (ESI) Calcd for $C_{40}H_{32}{}^{10}B_2{}^{11}B_8F_{12}N^-$ [$M-H^-$]: 863.3315, Found: 863.3335.

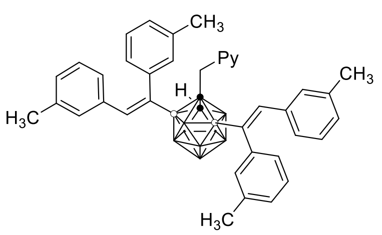

1-(2-Picolyl)-3,5-bis(*cis*-1,2-di(3-methylphenyl)vinyl)-*o*-carborane (3ak) was prepared as a white solid,, following the general procedure (GP-2) and purified by column chromatography on silica gel (200-300 mesh) with an eluent (*n*-hexane/ethyl acetate = 10/1) (93.5 mg, 72%). Mp: 79.5-81.3 °C. ^{1}H NMR (400 MHz, $CDCl_3$): δ 8.49 (d, J = 4.4 Hz, 1H), 7.55 (td, J = 8.0, 2.0 Hz, 1H), 7.21 (m， 4H), 7.11 (m, 5H), 7.01 (m, 3H), 6.94 (m, 4H), 6.85 (s, 1H), 6.80 (s, 1H), 6.73 (t, J = 9.6 Hz, 2H), 4.81 (s, 1H), 4.05 (d, J = 15.6 Hz, 1H), 3.72 (d, J = 15.2 Hz, 1H), 2.34 (s, 6H), 2.21 (s, 3H), 2.18 (s, 3H). ^{13}C{^{1}H} NMR ($CDCl_3$, 101 MHz): δ 156.2, 149.3, 143.4, 142.1, 141.9, 141.5, 138.3, 138.1, 137.5, 137.4, 137.4, 137.0, 136.6, 131.0, 130.8, 129.4, 129.3, 128.7, 128.5, 128.4, 128.0, 127.9, 127.8, 127.3, 127.0, 126.8, 126.7, 125.9, 125.8, 125.0, 122.6, 74.7, 57.7, 38.2, 21.71, 21.67, 21.5, 21.4. ^{11}B NMR ($CDCl_3$, 128 MHz): δ -2.4 (m, 4B), -11.5 (m, 6B). HRMS (ESI) Calcd for $C_{40}H_{45}{}^{10}B_2{}^{11}B_8N^+$ [M^+]: 649.4620, Found: 649.4625.

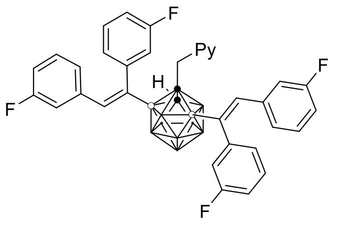

1-(2-Picolyl)-3,5-bis(*cis*-1,2-di(3-fluorophenyl)vinyl)-*o*-carborane (3al) was prepared as a white solid,, following the general procedure (GP-2) and purified by column chromatography on silica gel (200-300 mesh) with an eluent (*n*-hexane/ethyl acetate = 10/1) (115.6 mg, 87%). Mp: 76.9-77.8 °C. ^{1}H NMR (400 MHz, $CDCl_3$): δ 8.46 (d, J = 4.0 Hz, 1H), 7.58 (t, J = 7.6 Hz, 1H), 7.32 (m, 2H), 7.20 (m, 2H), 7.08 (m, 3H), 7.00 (m, 5H), 6.93 (d, J = 9.2 Hz, 1H), 6.85 (m, 3H), 6.77 (d, J = 7.6 Hz, 1H), 6.73 (d, J = 7.6 Hz, 1H), 6.61 (d, J = 8.8 Hz, 2H), 4.85 (s, 1H), 3.93

(d, J = 15.6 Hz, 1H), 3.61 (d, J =15.2 Hz, 1H). $^{13}C\{^1H\}$ NMR ($CDCl_3$, 101 MHz): δ 163.3 (d, $^1J_{CF}$ = 247.5 Hz), 163.2 (d, $^1J_{CF}$ = 247.5 Hz), 162.6 (d, $^1J_{CF}$ = 246.4 Hz), 162.5 (d, $^1J_{CF}$ = 246.4 Hz), 155.6, 149.6, 145.0 (d, $^3J_{CF}$ = 8.1 Hz), 143.4 (d, $^3J_{CF}$ = 8.1 Hz), 141.5 (d, $^4J_{CF}$ = 2.0 Hz), 141.1 (d, $^4J_{CF}$ = 2.0 Hz), 139.3 (d, $^3J_{CF}$ = 8.1 Hz), 138.7 (d, $^3J_{CF}$ = 8.1 Hz), 137.0, 130.6 (d, $^3J_{CF}$ = 8.1 Hz), 130.5 (d, $^3J_{CF}$ = 9.1 Hz), 129.7 (d, $^3J_{CF}$ = 8.1 Hz), 129.6 (d, $^3J_{CF}$ = 8.1 Hz), 125.7 (d, $^4J_{CF}$ = 3.0 Hz), 125.5 (d, $^4J_{CF}$ = 3.0 Hz), 124.7, 124.6, 124.57, 124.5, 122.9, 116.2 (d, $^2J_{CF}$ = 22.2 Hz), 116.1 (d, $^2J_{CF}$ = 22.2 Hz), 115.7 (d, $^2J_{CF}$ = 22.2 Hz), 115.6 (d, $^2J_{CF}$ = 21.2 Hz), 114.9 (d, $^2J_{CF}$ = 21.2 Hz), 114.5 (d, $^2J_{CF}$ = 21.2 Hz), 114.0 (d, $^2J_{CF}$ = 21.2 Hz), 113.7 (d, $^2J_{CF}$ = 21.2 Hz), 74.6, 57.8, 38.1. ^{11}B NMR ($CDCl_3$, 128 MHz): δ -2.5 (m, 4B), -11.6 (m, 6B). ^{19}F NMR ($CDCl_3$, 376 MHz): δ -112.2 (m, 1F), -112.4 (m, 1F), -113.1 (m, 1F), -113.2 (m, 1F). HRMS (ESI) Calcd for $C_{36}H_{33}{}^{10}B_2{}^{11}B_8F_4N^+$ [M^+]: 664.3631, Found: 664.3621.

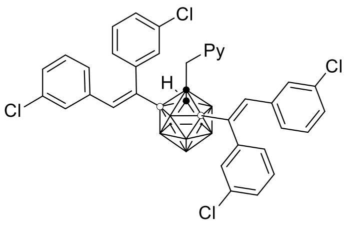

1-(2-Picolyl)-3,5-bis(*cis*-1,2-di(3-chlorophenyl)vinyl)-*o*-carborane (3am) was prepared as a white solid,, following the general procedure (GP-2) and purified by column chromatography on silica gel (200-300 mesh) with an eluent (*n*-hexane/ethyl acetate = 10/1) (75.9 mg, 52%). Mp: 73.6-74.1 °C. ^1H NMR (400 MHz, $CDCl_3$): δ 8.54 (d, J = 4.0 Hz, 1H), 7.62 (t, J = 7.6 Hz, 1H), 7.44 (s, 1H), 7.30 (m, 4H), 7.22 (m, 3H), 7.11 (m, 4H), 7.03 (m, 5H), 6.89 (d, J = 8.4 Hz, 1H), 6.80 (m, 2H), 4.87 (s, 1H), 3.96 (d, J = 15.2 Hz, 1H), 3.63 (d, J = 15.6 Hz, 1H). $^{13}C\{^1H\}$ NMR ($CDCl_3$, 101 MHz): δ 155.4, 149.6, 144.5, 143.0, 141.6, 141.1, 138.8, 138.3, 137.1, 134.8, 134.6, 134.2, 134.1, 130.3, 130.2, 130.0, 129.8, 129.5, 129.4, 128.7, 128.0,

127.7, 127.6, 127.5, 127.3, 127.2, 127.1, 127.0, 124.7, 123.0, 74.7, 57.7, 38.1. ^{11}B NMR (CDCl$_3$, 128 MHz): δ -2.4 (m, 4B), -11.6 (m, 6B). HRMS (ESI) Calcd for $C_{36}H_{33}{}^{10}B_2{}^{11}B_8Cl_4N^+$ [M$^+$]: 730.2429, Found: 730.2411.

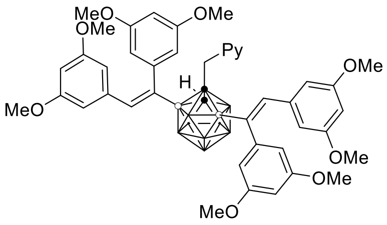

1-(2-Picolyl)-3,5-bis(*cis*-1,2-di(3,5-dimethoxylphenyl)vinyl)-*o*-carborane (3an) was prepared as a colorless oil, following the general procedure (GP-2) and purified by column chromatography on silica gel (200-300 mesh) with an eluent (*n*-hexane/ethyl acetate = 10/1) (136.7 mg, 82%). ^{1}H NMR (400 MHz, CDCl$_3$): δ 8.49 (d, J = 4.4 Hz, 1H), 7.60 (t, J = 7.6 Hz, 1H), 7.21 (m, 1H), 7.13 (s, 1H), 7.04 (d, J = 7.6 Hz, 1H), 6.87 (s, 1H), 6.50 (s, 2H), 6.40 (m, 4H), 6.26 (m, 6H), 4.84 (s, 1H), 4.04 (d, J = 15.6 Hz, 1H), 3.78 (m, 13H), 3.59 (s, 6H), 3.56 (s, 6H). ^{13}C{^{1}H} NMR (CDCl$_3$, 101 MHz): δ 161.4, 161.2, 160.3, 160.2, 156.1, 149.4, 145.6, 143.9, 141.9, 139.0, 138.4, 136.8, 125.0, 122.7, 107.5, 107.4, 106.7, 106.5 101.2, 100.6, 99.2, 98.4, 74.8, 57.9, 55.6, 55.5, 55.1, 38.4. ^{11}B NMR (CDCl$_3$, 128 MHz): δ -2.7 (m, 4B), -11.3 (m, 6B). HRMS (ESI) Calcd for $C_{44}H_{53}{}^{10}B_2{}^{11}B_8O_8N^+$ [M$^+$]: 833.4843, Found: 833.4827.

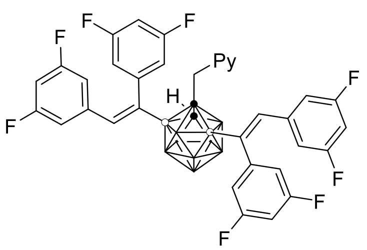

1-(2-Picolyl)-3,5-bis(*cis*-1,2-di(3,5-difluorophenyl)vinyl)-*o*-carborane (3ao) was prepared as a white solid, following the general procedure (GP-2) and purified by column chromatography on silica gel (200-300 mesh) with an eluent (*n*-hexane/ethyl acetate = 10/1) (117.8 mg, 80%). Mp: 83.6-84.2 °C. ^{1}H NMR (400 MHz, CDCl$_3$): δ 8.50 (d, J = 4.0 Hz, 1H), 7.63 (t, J = 7.2

Hz, 1H), 7.25 (s, 1H), 7.16 (s, 1H), 6.90 (s, 2H), 6.82 (m, 2H), 6.72 (m, 4H), 6.61 (m, 2H), 6.45 (m, 4H), 4.86 (s, 1H), 3.86 (d, J = 15.2 Hz, 1H), 3.55 (d, J =15.6 Hz, 1H). $^{13}C\{^1H\}$ NMR ($CDCl_3$, 101 MHz): δ 163.7 (d, $^1J_{CF}$ = 248.7 Hz), 163.5 (d, $^1J_{CF}$ = 248.8 Hz), 163.4 (d, $^1J_{CF}$ = 248.6 Hz), 162.9 (d, $^1J_{CF}$ = 246.7 Hz), 162.8 (d, $^1J_{CF}$ = 246.4 Hz), 162.6 (d, $^1J_{CF}$ = 246.4 Hz), 155.1, 149.7, 145.6 (d, $^3J_{CF}$ = 10.0 Hz), 145.4 (d, $^3J_{CF}$ = 9.0 Hz), 141.1,140.5, 139.7 139.7 (d, $^3J_{CF}$ = 6.0 Hz), 139.6 (d, $^3J_{CF}$ = 6.0 Hz), 139.1 (d, $^3J_{CF}$ = 9.0 Hz), 139.0 (d, $^3J_{CF}$ = 9.0 Hz), 137.3, 124.6, 123.3, 112.5-111.4 (m), 103.9 (d, $^2J_{CF}$ = 25.4 Hz), 103.6 (d, $^2J_{CF}$ = 25.2 Hz), 103.5 (d, $^2J_{CF}$ = 25.0 Hz), 103.9 (d, $^2J_{CF}$ = 25.4 Hz), 103.2 (d, $^2J_{CF}$ = 25.3 Hz), 103.9 (d, $^2J_{CF}$ = 25.4 Hz), 103.1 (d, $^2J_{CF}$ = 25.3 Hz), 102.8 (d, $^2J_{CF}$ = 24.9 Hz), 102.5 (d, $^2J_{CF}$ = 24.9 Hz), 74.6, 57.9, 38.2. ^{11}B NMR ($CDCl_3$, 128 MHz): δ -2.4 (m, 4B), -11.5 (m, 6B). ^{19}F NMR ($CDCl_3$, 376 MHz): δ -108.3 (m, 2F), -108.5 (m, 2F), -109.6 (m, 2F), -109.7 (m, 2F). HRMS (ESI) Calcd for $C_{36}H_{29}{}^{10}B_2{}^{11}B_8F_8N^+$ [M^+]: 736.3265, Found: 736.3273.

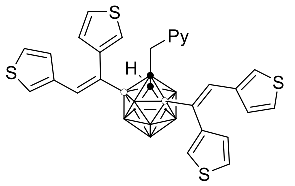

1-(2-Picolyl)-3,5-bis(*cis*-1,2-di(3-thienyl)vinyl)-*o*-carborane (3aq) was prepared as a white solid, following the general procedure (GP-2) and purified by column chromatography on silica gel (200-300 mesh) with an eluent (*n*-hexane/ethyl acetate = 10/1) (66.7 mg, 54%). Mp: 199.4-199.8 °C. ^1H NMR (400 MHz, $CDCl_3$): δ 8.45 (d, J = 4.0 Hz, 1H), 7.54 (t, J = 7.6 Hz, 1H), 7.36 (m, 2H), 7.23 (m, 2H), 7.17 (t, J = 6.0 Hz, 1H), 7.06 (m, 3H), 6.97 (m, 3H), 6.91 (m, 3H), 6.51 (d, J = 4.4 Hz, 2H), 4.68 (s, 1H), 3.91 (d, J = 15.6 Hz, 1H), 3.62 (d, J =15.6 Hz, 1H). $^{13}C\{^1H\}$ NMR ($CDCl_3$, 101 MHz): δ 156.0, 149.4, 143.7, 141.8, 139.5, 139.1, 137.5, 136.8, 136.4, 128.8, 128.7, 128.2,

128.1, 126.4, 126.1, 125.7, 125.4, 125.0, 124.9, 124.8, 122.6, 121.8, 121.4, 74.6, 58.0, 38.5. ^{11}B NMR ($CDCl_3$, 128 MHz): δ -2.7 (m, 4B), -11.9 (m, 6B). HRMS (ESI) Calcd for $C_{28}H_{29}{}^{10}B_2{}^{11}B_8S_4N^+$ [M^+]: 617.2246, Found: 617.2220.

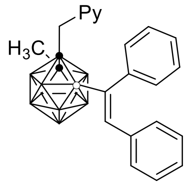

1-(2-Picolyl)-2-methyl-4-(*cis*-1,2-diphenylvinyl)-*o*-carborane (4ba) was prepared as a white solid, following the general procedure (GP-3) and purified by column chromatography on silica gel (200-300 mesh) with an eluent (*n*-hexane/ethyl acetate = 10/1) (197.5 mg, 92%). Single crystals suitable for X-ray analyses were obtained by slow evaporation of an ether acetate solution over a period of 3 days at ambient conditions. Mp: 138.1-138.9 °C. ^{1}H NMR (400 MHz, $CDCl_3$): δ 8.47 (d, J = 3.6 Hz, 1H), 7.55 (t, J = 6.8 Hz, 1H), 7.35 (t, J = 7.2 Hz, 2H), 7.27 (m, 3H), 7.14 (m, 5H), 6.99 (m, 2H), 6.76 (d, J = 7.6 Hz, 1H), 3.99 (d, J = 15.2 Hz, 1H), 3.48 (d, J = 15.6 Hz, 1H), 2.29 (s, 1H). ^{13}C{^{1}H} NMR ($CDCl_3$, 101 MHz): δ 156.1, 149.2, 143.4, 142.4, 137.6, 136.7, 130.0, 129.1, 128.8, 128.1, 127.3, 126.5, 125.1, 122.8, 77.6, 40.6, 24.0. ^{11}B NMR ($CDCl_3$, 128 MHz): δ 0.8 (s, 1B), -3.1 (d, J = 147.2 Hz, 1B), -4.5 (d, J = 163.8 Hz, 1B), -11.1 (m, 7B). HRMS (DART) Calcd for $C_{23}H_{31}{}^{10}B_2{}^{11}B_8N^+$ [$M+H^+$]: 429.3454, Found: 429.3338.

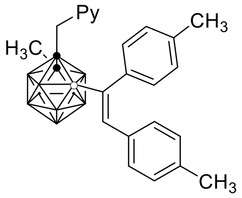

1-(2-Picolyl)-2-methyl-4-(*cis*-1,2-di(4-methylphenyl)vinyl)-*o*-carborane (4bb) was prepared as a white solid, following the general procedure (GP-3) and purified by column chromatography on silica gel (200-300 mesh) with an eluent (*n*-hexane/ethyl acetate = 10/1) (209.9 mg, 92%). Mp: 70.7-71.4 °C. ^{1}H NMR (400 MHz, $CDCl_3$): δ 8.50 (d, J =

3.6 Hz, 1H), 7.58 (t, J = 7.2 Hz, 1H), 7.16 (m, 6H), 6.95 (m, 4H), 6.85 (d, J = 7.6 Hz, 1H), , 4.03 (d, J = 15.2 Hz, 1H), 3.49 (d, J = 15.2 Hz, 1H), 2.41 (s, 6H), 2.32 (s, 3H), 2.28 (s, 3H). $^{13}C\{^1H\}$ NMR ($CDCl_3$, 101 MHz): δ 156.2, 149.2, 142.1, 140.5, 137.1, 136.6, 135.9, 134.8, 129.7, 129.5, 128.82, 128.75, 125.1, 122.8, 77.6, 76.6, 40.5, 24.0, 21.4, 21.3. ^{11}B NMR ($CDCl_3$, 128 MHz): δ 1.2 (s, 1B), -3.2 (d, J = 151.0 Hz, 1B), -4.5 (d, J = 153.6 Hz, 1B), -11.6 (m, 7B). HRMS (DART) Calcd for $C_{25}H_{34}{}^{10}B_2{}^{11}B_8N^+$ [M+H$^+$]: 456.3689, Found: 456.3684.

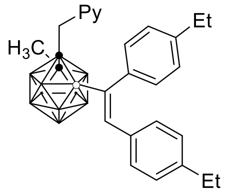

1-(2-Picolyl)-2-methyl-4-(*cis*-1,2-di(4-ethylphenyl)vinyl)-*o*-carborane (4bc) was prepared as a white solid, following the general procedure (GP-3) and purified by column chromatography on silica gel (200-300 mesh) with an eluent (*n*-hexane/ethyl acetate = 10/1) (215.6 mg, 89%). Mp: 131.5-132.2 °C. ^1H NMR (400 MHz, $CDCl_3$): δ 8.45 (d, J = 4.0 Hz, 1H), 7.50 (td, J = 7.2, 1.6 Hz, 1H), 7.18 (m, 4H), 7.12 (m, 2H), 6.94 (m, 4H), 6.69 (d, J = 7.6 Hz, 1H), 4.00 (d, J = 15.2 Hz, 1H), 3.45 (d, J = 15.2 Hz, 1H), 2.69 (q, J = 7.2 Hz, 2H), 2.53 (q, J = 7.6 Hz, 2H), 2.28 (s, 3H), 1.28 (t, J = 7.6 Hz, 3H), 1.15 (t, J = 7.6 Hz, 3H). $^{13}C\{^1H\}$ NMR ($CDCl_3$, 101 MHz): δ 156.2, 149.1, 143.4, 142.4, 142.1, 140.7, 136.6, 135.0, 129.8, 129.0, 128.2, 127.5, 125.1, 122.7, 77.6, 76.8, 40.4, 28.7, 28.6, 23.2, 15.9, 15.4. ^{11}B NMR ($CDCl_3$, 128 MHz): δ 1.2 (s, 1B), -3.2 (d, J = 140.8 Hz, 1B), -4.3 (d, J = 152.3 Hz, 1B), -11.4 (m, 7B). HRMS (DART) Calcd for $C_{27}H_{38}{}^{10}B_2{}^{11}B_8N^+$ [M+H$^+$]: 484.4002, Found: 484.4002.

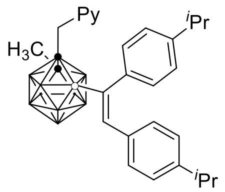

1-(2-Picolyl)-2-methyl-4-(*cis*-1,2-di(4-*iso*-propylphenyl)vinyl)-*o*-carborane (4bd) was prepared as a white solid, following the general procedure (GP-3) and purified by column chromatography on silica gel (200-300 mesh) with an eluent (*n*-hexane/ethyl acetate = 10/1) (228.0 mg, 89%). Mp: 135.4-136.3 °C. ^{1}H NMR (400 MHz, $CDCl_3$): δ 8.45 (d, J = 4.0 Hz, 1H), 7.49 (t, J = 7.2 Hz, 1H), 7.20 (m, 4H), 7.13 (m, 2H), 6.96 (m, 4H), 6.62 (d, J = 7.6 Hz, 1H), 4.01 (d, J = 15.2 Hz, 1H), 3.45 (d, J = 15.2 Hz, 1H), 2.95 (m, 1H), 2.86 (m, 1H), 2.29 (s, 3H), 1.29 (d, J = 6.4 Hz, 6H), 1.18 (d, J = 6.0 Hz, 6H). ^{13}C{^{1}H} NMR ($CDCl_3$, 101 MHz): δ 149.1, 148.1, 142.2, 140.8, 136.6, 135.2, 129.8, 129.0, 126.8, 126.1, 125.1, 122.7, 77.5, 77.0, 40.3, 34.0, 33.9, 24.3, 24.2, 24.0, 23.9, 23.8. ^{11}B NMR ($CDCl_3$, 128 MHz): δ 1.1 (s, 1B), -3.3 (d, J = 153.6 Hz, 1B), -4.4 (d, J = 140.8 Hz, 1B), -11.4 (m, 7B). HRMS (DART) Calcd for $C_{29}H_{42}{}^{10}B_2{}^{11}B_8N^+$ [M+H$^+$]: 512.4315, Found: 512.4310.

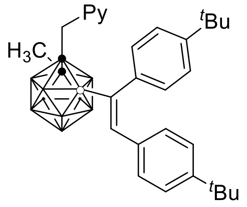

1-(2-Picolyl)-2-methyl-4-(*cis*-1,2-di(4-*tert*-butylphenyl)vinyl)-*o*-carborane (4be) was prepared as a white solid, following the general procedure (GP-3) and purified by column chromatography on silica gel (200-300 mesh) with an eluent (*n*-hexane/ethyl acetate = 10/1) (237.8 mg, 88%). Mp: 123.4-124.2 °C. ^{1}H NMR (400 MHz, $CDCl_3$): δ 8.51 (d, J = 3.2 Hz, 1H), 7.53 (t, J = 7.2 Hz, 1H), 7.44 (m, 2H), 7.22 (m, 6H), 7.02 (d, J = 8.4 Hz, 2H), 6.64 (d, J = 7.6 Hz, 1H), 4.07 (d, J = 15.2 Hz, 1H), 3.52 (d, J = 14.8 Hz, 1H), 2.35 (s, 3H), 1.44 (s, 9H), 1.31 (s, 9H). ^{13}C{^{1}H} NMR ($CDCl_3$, 101 MHz): δ 156.3, 150.4, 149.4, 149.1, 142.1, 140.4, 136.6, 134.8, 129.6, 128.7, 125.6, 125.1, 125.0, 122.7, 77.0,

40.3, 34.7, 34.6, 31.7, 31.3, 23.9. ^{11}B NMR ($CDCl_3$, 128 MHz): δ 1.4 (s, 1B), -3.1 (d, *J* = 145.9 Hz, 1B), -4.3 (d, *J* = 151.0 Hz, 1B), -11.3 (m, 7B). HRMS (DART) Calcd for $C_{31}H_{46}{}^{10}B_2{}^{11}B_8N^+$ [M+H$^+$]: 540.4628, Found: 540.4622.

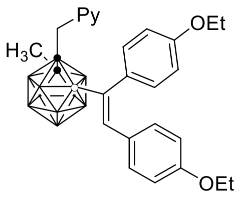

1-(2-Picolyl)-2-methyl-4-(*cis*-1,2-di(4-ethoxylphenyl)vinyl)-*o*-carborane (4bf) was prepared as a white solid, following the general procedure (GP-3) and purified by column chromatography on silica gel (200-300 mesh) with an eluent (*n*-hexane/ethyl acetate = 10/1) (237.5 mg, 92%). Mp: 68.9-69.5 °C. ^{1}H NMR (400 MHz, $CDCl_3$): δ 8.49 (d, *J* = 4.8 Hz, 1H), 7.58 (td, *J* = 7.6, 1.2 Hz, 1H), 7.18 (m, 3H), 7.09 (s, 1H), 6.95 (d, *J* = 8.8 Hz, 2H), 6.92 (d, *J* = 8.0 Hz, 2H), 6.84 (d, *J* = 7.6 Hz, 1H), 6.68 (d, *J* = 8.8 Hz, 2H), 4.08 (q, *J* = 6.8 Hz, 2H), 3.98 (m, 3H), 3.47 (d, *J* = 15.2 Hz, 1H), 2.30 (s, 3H), 1.47 (t, *J* = 6.8 Hz, 3H), 1.38 (t, *J* = 6.8 Hz, 3H). ^{13}C{^{1}H} NMR ($CDCl_3$, 101 MHz): δ 158.1, 157.5, 156.2, 149.1, 141.7, 136.6, 135.7, 131.1, 130.3, 130.1, 125.1, 122.7, 114.8, 114.0, 77.6, 76.6, 63.5, 63.4, 40.5, 23.9, 15.0, 14.9. ^{11}B NMR ($CDCl_3$, 128 MHz): δ 1.4 (s, 1B), -3.3 (d, *J* = 160.0 Hz, 1B), -4.4 (d, *J* = 139.5 Hz, 1B), -11.4 (m, 7B). HRMS (DART) Calcd for $C_{27}H_{38}{}^{10}B_2{}^{11}B_8O_2N^+$ [M+H$^+$]: 516.3900, Found: 516.3895.

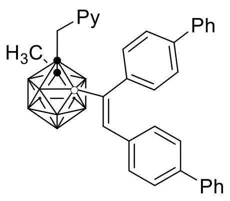

1-(2-Picolyl)-2-methyl-4-(*cis*-1,2-di(4-biphenyl)vinyl)-*o*-carborane (4bg) was prepared as a white solid, following the general procedure (GP-3) and purified by column chromatography on silica gel (200-300 mesh) with an eluent (*n*-hexane/ethyl acetate = 10/1) (264.1 mg, 91%). Mp: 171.9-172.4 °C. ^{1}H NMR (400 MHz, $CDCl_3$): δ 8.48 (d, *J* = 4.0 Hz, 1H), 7.65 (m, 4H), 7.52 (m, 3H), 7.45 (m, 2H), 7.37 (m, 7H), 7.29 (m, 1H), 7.23 (m, 1H),

7.14 (m, 3H), 6.78 (d, J = 8.0 Hz, 1H), 4.02 (d, J = 15.2 Hz, 1H), 3.51 (d, J = 15.2 Hz, 1H), 2.30 (s, 3H). $^{13}C\{^1H\}$ NMR ($CDCl_3$, 101 MHz): δ 156.1, 149.2, 142.5, 142.1, 140.8, 140.6, 140.0, 139.2, 136.7, 136.6, 130.3, 129.6, 129.0, 128.8, 127.4, 127.0, 126.9, 126.7, 125.1, 122.8, 77.7, 76.9, 40.6, 24.0. ^{11}B NMR ($CDCl_3$, 128 MHz): δ 0.9 (s, 1B), -3.1 (d, J = 145.9 Hz, 1B), -4.4 (d, J = 169.0 Hz, 1B), -11.3 (m, 7B). HRMS (ESI) Calcd for $C_{35}H_{38}{}^{10}B_2{}^{11}B_8N^+$ [M+H$^+$]: 580.4002, Found: 580.4016.

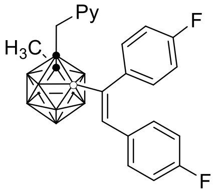

1-(2-Picolyl)-2-methyl-4-(*cis*-1,2-di(4-fluorophenyl)vinyl)-*o*-carborane (4bh) was prepared as a white solid, following the general procedure (GP-3) and purified by column chromatography on silica gel (200-300 mesh) with an eluent (*n*-hexane/ethyl acetate = 10/1) (215.9 mg, 93%). Mp: 126.4-127.3 °C. ^1H NMR (400 MHz, $CDCl_3$): δ 8.50 (d, J = 4.0 Hz, 1H), 7.60 (td, J = 7.6, 1.6 Hz, 1H), 7.21 (m, 3H), 7.15 (s, 1H), 7.07 (t, J = 8.4 Hz, 2H), 6.96 (m, 2H), 6.83 (m, 3H), 3.94 (d, J = 15.2 Hz, 1H), 3.51 (d, J = 15.2 Hz, 1H), 2.31 (s, 3H). $^{13}C\{^1H\}$ NMR ($CDCl_3$, 101 MHz): δ 161.8 (d, $^1J_{CF}$ = 249.5 Hz), 161.7 (d, $^1J_{CF}$ = 246.4 Hz), 155.9, 149.3, 141.5, 138.9 (d, $^4J_{CF}$ = 3.0 Hz), 136.8, 133.5 (d, $^4J_{CF}$ = 4.0 Hz), 131.4 (d, $^3J_{CF}$ = 8.1 Hz), 130.6 (d, $^3J_{CF}$ = 8.1 Hz), 124.9, 122.9, 115.9 (d, $^2J_{CF}$ = 21.2 Hz), 115.1 (d, $^2J_{CF}$ = 21.2 Hz), 77.6, 77.4, 40.7, 24.0. ^{11}B NMR ($CDCl_3$, 128 MHz): δ 1.2 (s, 1B), -3.2 (d, J = 153.6 Hz, 1B), -4.5 (d, J = 162.6 Hz, 1B), -11.4 (m, 7B). ^{19}F NMR ($CDCl_3$, 376 MHz): δ -113.0 (m, 1F), -113.7 (m, 1F), -115.6 (m, 1F), -115.9 (m, 1F). HRMS (DART) Calcd for $C_{23}H_{28}{}^{10}B_2{}^{11}B_8F_2N^+$ [M+H$^+$]: 464.3188, Found: 464.3183.

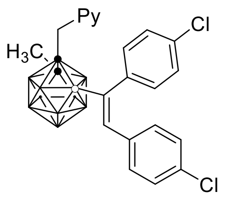

1-(2-Picolyl)-2-methyl-4-(*cis*-1,2-di(4-chlorophenyl)vinyl)-*o*-carborane (4bi) was prepared as a white solid, following the general procedure (GP-3) and purified by column chromatography on silica gel (200-300 mesh) with an eluent (*n*-hexane/ethyl acetate = 10/1) (166.2 mg, 67%). Mp: 54.1-54.8 °C. ^{1}H NMR (400 MHz, CDCl$_3$): δ 8.50 (d, J = 4.4 Hz, 1H), 7.61 (t, J = 8.0 Hz, 1H), 7.34 (m, 2H), 7.17 (m, 6H), 6.91 (d, J = 8.0 Hz, 2H), 6.81 (d, J = 8.0 Hz, 1H), 3.89 (d, J = 15.2 Hz, 1H), 3.50 (d, J = 15.2 Hz, 1H), 2.29 (s, 3H). ^{13}C{^{1}H} NMR (CDCl$_3$, 101 MHz): δ 155.8, 149.3, 141.5, 136.9, 135.7, 133.2, 132.6, 130.9, 130.4, 129.2, 128.4, 124.9, 123.0, 40.7, 24.0. ^{11}B NMR (CDCl$_3$, 128 MHz): δ 0.4 (s, 1B), -3.1 (d, J = 165.1 Hz, 1B), -4.7 (d, J = 163.8 Hz, 1B), -11.4 (m, 7B). HRMS (DART) Calcd for $C_{23}H_{28}{}^{10}B_2{}^{11}B_8Cl_2N^+$ [M+H$^+$]: 496.2596, Found: 496.2595.

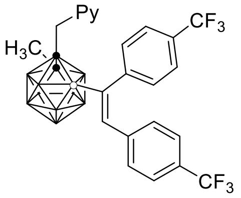

1-(2-Picolyl)-2-methyl-4-(*cis*-1,2-bis(4-(trifluoromethyl)phenyl)vinyl)-*o*-carborane (4bj) was prepared as a colorless oil, following the general procedure (GP-3) and purified by column chromatography on silica gel (200-300 mesh) with an eluent (*n*-hexane/ethyl acetate = 10/1) (240.3 mg, 85%). ^{1}H NMR (400 MHz, CDCl$_3$): δ 8.50 (d, J = 4.0 Hz, 1H), 7.60 (m, 3H), 7.38 (m, 4H), 7.25 (m, 1H), 7.19 (m, 1H), 7.06 (d, J = 8.0 Hz, 2H), 6.75 (d, J = 7.6 Hz, 1H), 3.89 (d, J = 15.6 Hz, 1H), 3.53 (d, J = 15.2 Hz, 1H), 2.30 (s, 3H). ^{13}C{^{1}H} NMR (CDCl$_3$, 101 MHz): δ 155.6, 149.4, 146.7, 141.5, 140.5, 136.8, 129.8, 129.4, 129.3 (q, $^{2}J_{CF}$ = 24.0 Hz), 129.0 (q, $^{2}J_{CF}$ = 24.0 Hz), 125.8 (q, $^{3}J_{CF}$ = 4.0 Hz), 125.1 (q, $^{3}J_{CF}$ = 4.0 Hz), 124.8, 124.3 (q, $^{1}J_{CF}$ = 270.1 Hz), 124.1 (q, $^{1}J_{CF}$ = 270.0 Hz), 123.0, 77.7, 77.1, 40.7, 24.0. ^{11}B NMR (CDCl$_3$, 128 MHz): δ 0.11 (s,

1B), -3.1 (d, J = 151.0 Hz, 1B), -4.5 (d, J = 137.0 Hz, 1B), -11.3 (m, 7B). ^{19}F NMR ($CDCl_3$, 376 MHz): δ -62.3 (s, 3F), -62.6 (s, 3F). HRMS (DART) Calcd for $C_{25}H_{28}{}^{10}B_2{}^{11}B_8F_6N^+$ [M+H$^+$]: 565.3087, Found: 565.3085.

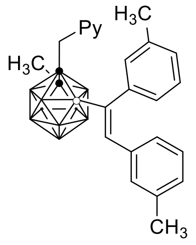

1-(2-Picolyl)-2-methyl-4-(*cis*-1,2-di(3-methylphenyl)vinyl)-*o*-carborane (4bk) was prepared as a white solid, following the general procedure (GP-3) and purified by column chromatography on silica gel (200-300 mesh) with an eluent (*n*-hexane/ethyl acetate = 10/1) (198.5 mg, 87%). Mp: 57.3-57.9 °C. ^{1}H NMR (400 MHz, $CDCl_3$): δ 8.50 (d, J = 3.2 Hz, 1H), 7.58 (t, J = 7.2 Hz, 1H), 7.25 (m, 1H), 7.16 (m, 4H), 7.04 (m, 2H), 6.95 (d, J = 7.8 Hz, 1H), 6.90 (s, 1H), 6.82 (m, 2H), 4.07 (d, J = 15.2 Hz, 1H), 3.52 (d, J = 15.2 Hz, 1H), 2.34 (d, J = 15.2 Hz, 6H), 2.22 (s, 3H). ^{13}C{^{1}H} NMR ($CDCl_3$, 101 MHz): δ 156.2, 149.2, 143.5, 142.2, 138.2, 137.4, 136.7, 130.8, 129.4, 128.6, 128.1, 127.9, 127.1, 126.6, 125.9, 125.0, 122.8, 77.7, 76.7, 40.5, 23.9, 21.7, 21.5. ^{11}B NMR ($CDCl_3$, 128 MHz): δ 1.1 (s, 1B), -3.2 (d, J = 134.4 Hz, 1B), -4.4 (d, J = 156.2 Hz, 1B), -11.4 (m, 7B). HRMS (DART) Calcd for $C_{25}H_{34}{}^{10}B_2{}^{11}B_8N^+$ [M+H$^+$]: 456.3689, Found: 456.3684.

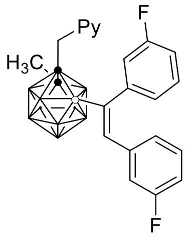

1-(2-Picolyl)-2-methyl-4-(*cis*-1,2-di(3-fluorophenyl)vinyl)-*o*-carborane (4bl) was prepared as a white solid, following the general procedure (GP-3) and purified by column chromatography on silica gel (200-300 mesh) with an eluent (*n*-hexane/ethyl acetate = 10/1) (213.6 mg, 92%). Mp: 133.9-134.6 °C. ^{1}H NMR (400 MHz, $CDCl_3$): δ 8.51 (d, J = 4.4 Hz, 1H), 7.61 (t, J = 7.6 Hz, 1H), 7.33 (m, 1H), 7.21 (m, 1H), 7.12 (m, 2H), 7.0 (m, 3H),

6.84 (m, 3H), 6.65 (d, J = 10.4 Hz, 1H), 3.95 (d, J = 15.2 Hz, 1H), 3.53 (d, J = 15.2 Hz, 1H), 2.31 (s, 3H). $^{13}C\{^1H\}$ NMR ($CDCl_3$, 101 MHz): δ 163.2 (d, $^1J_{CF}$ = 247.5 Hz), 162.5 (d, $^1J_{CF}$ = 245.4 Hz), 155.8, 149.3, 145.2 (d, $^3J_{CF}$ = 8.1 Hz), 141.4 (d, $^4J_{CF}$ = 2.0 Hz), 139.3 (d, $^3J_{CF}$ = 8.1 Hz), 136.8, 130.4 (d, $^3J_{CF}$ = 8.1 Hz), 129.5 (d, $^3J_{CF}$ = 9.1 Hz), 125.6 (d, $^4J_{CF}$ = 2.0 Hz), 124.9, 124.5 (d, $^4J_{CF}$ = 3.0 Hz), 122.9, 116.1 (d, $^2J_{CF}$ = 22.2 Hz), 115.7 (d, $^2J_{CF}$ = 22.2 Hz), 114.5 (d, $^2J_{CF}$ = 21.2 Hz), 113.7 (d, $^2J_{CF}$ = 21.2 Hz), 77.6, 77.3, 40.7, 24.0. ^{11}B NMR ($CDCl_3$, 128 MHz): δ 0.3 (s, 1B), -3.0 (d, J = 151.0 Hz, 1B), -4.5 (d, J = 158.7 Hz, 1B), -11.4 (m, 7B). ^{19}F NMR ($CDCl_3$, 376 MHz): δ -112.2 (m, 1F), -113.4 (m, 1F). HRMS (DART) Calcd for $C_{23}H_{28}{}^{10}B_2{}^{11}B_8F_2N^+$ [M+H$^+$]: 464.3188, Found: 464.3186.

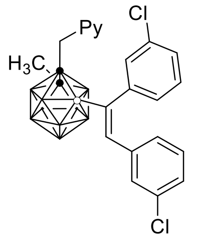

1-(2-Picolyl)-2-methyl-4-(*cis*-1,2-di(3-chlorophenyl)vinyl)-*o*-carborane (4bm) was prepared as a white solid, following the general procedure (GP-3) and purified by column chromatography on silica gel (200-300 mesh) with an eluent (*n*-hexane/ethyl acetate = 10/1) (210.9 mg, 85%). Mp: 110.2-110.9 °C. ^1H NMR (400 MHz, $CDCl_3$): δ 8.51 (d, J = 4.0 Hz, 1H), 7.61 (t, J = 7.2 Hz, 1H), 7.28 (m, 3H), 7.20 (m, 1H), 7.12 (m, 3H), 7.05 (m, 2H), 6.84 (m, 2H), 3.93 (d, J = 15.2 Hz, 1H), 3.53 (d, J = 15.2 Hz, 1H), 2.32 (s, 3H). $^{13}C\{^1H\}$ NMR ($CDCl_3$, 101 MHz): δ 155.8, 149.3, 144.8, 141.2, 138.8, 136.8, 134.6, 134.0, 130.1, 129.8, 129.3, 128.6, 127.6, 127.1, 126.9, 124.8, 122.9, 77.6, 77.4, 40.7, 24.0. ^{11}B NMR ($CDCl_3$, 128 MHz): δ 0.2 (s, 1B), -3.1 (d, J = 161.3 Hz, 1B), -4.5 (d, J = 143.4 Hz, 1B), -11.3 (m, 7B). HRMS (DART) Calcd for $C_{23}H_{28}{}^{10}B_2{}^{11}B_8Cl_2N^+$ [M+H$^+$]: 496.2596, Found: 496.2593.

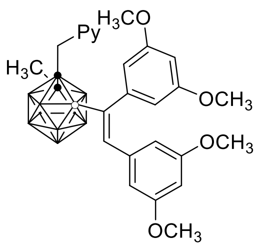

1-(2-Picolyl)-2-methyl-4-(*cis*-1,2-di(3,5-dimethoxylphenyl)vinyl)-*o*-carborane (4bn) was prepared as a colorless oil, following the general procedure (GP-3) and purified by column chromatography on silica gel (200-300 mesh) with an eluent (*n*-hexane/ethyl acetate = 10/1) (252.3 mg, 92%). ^{1}H NMR (400 MHz, $CDCl_3$): δ 8.50 (s, 1H), 7.61 (t, J = 7.6 Hz, 1H), 7.20 (t, J = 5.6 Hz, 1H), 7.01 (m, 2H), 6.45 (s, 2H), 6.39 (s, 1H), 6.29 (d, J = 7.6 Hz, 3H), 4.07 (d, J = 16.0 Hz, 1H), 3.86 (s, 6H), 3.54 (m, 7H), 2.30 (s, 3H). $^{13}C\{^1H\}$ NMR ($CDCl_3$, 101 MHz): δ 161.2, 160.2, 156.0, 149.2, 145.7, 141.8, 139.0, 136.8, 125.0, 122.9, 107.4, 106.6, 100.7, 98.7, 77.7, 76.6, 55.5, 55.1, 40.6, 24.0. ^{11}B NMR ($CDCl_3$, 128 MHz): δ 0.5 (s, 1B), -3.3 (d, J = 163.8 Hz, 1B), -4.4 (d, J = 128.0 Hz, 1B), -11.5 (m, 7B). HRMS (DART) Calcd for $C_{27}H_{38}{}^{10}B_2{}^{11}B_8O_4N^+$ [M+H$^+$]: 548.3799, Found: 548.3792.

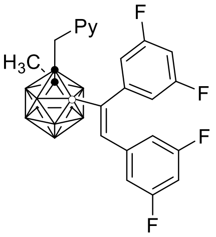

1-(2-Picolyl)-2-methyl-4-(*cis*-1,2-di(3,5-difluorophenyl)vinyl)-*o*-carborane (4bo) was prepared as a white solid, following the general procedure (GP-3) and purified by column chromatography on silica gel (200-300 mesh) with an eluent (*n*-hexane/ethyl acetate = 10/1) (227.6 mg, 91%). Mp: 151.1-151.8 °C. ^{1}H NMR (400 MHz, $CDCl_3$): δ 8.52 (d, J = 4.4 Hz, 1H), 7.65 (t, J = 7.2 Hz, 1H), 7.24 (m, 1H), 7.07 (s, 1H), 6.95 (d, J = 7.6 Hz, 1H), 6.76 (d, J = 7.2 Hz, 3H), 6.62 (t, J = 8.8 Hz, 1H) , 6.50 (d, J = 7.2 Hz, 2H), 3.89 (d, J = 15.2 Hz, 1H), 3.55 (d, J = 15.2 Hz, 1H), 2.31 (s, 3H). $^{13}C\{^1H\}$ NMR ($CDCl_3$, 101 MHz): δ 163.5 (d, $^1J_{CF}$ = 248.2 Hz), 163.4 (d, $^1J_{CF}$ = 248.4 Hz), 162.8 (d, $^1J_{CF}$ = 246.4 Hz), 162.7 (d, $^1J_{CF}$ = 246.5 Hz), 155.5, 149.5, 146.0 (d, $^3J_{CF}$ =

9.3 Hz), 145.9 (d, $^3J_{CF}$ = 9.3 Hz), 140.5 (d, $^4J_{CF}$ = 2.2 Hz), 140.4 (d, $^4J_{CF}$ = 2.2 Hz), 139.9 (d, $^3J_{CF}$ = 9.3 Hz), 139.8 (d, $^3J_{CF}$ = 9.6 Hz), 136.9, 124.8, 123.1, 112.4-111.4 (m), 103.4 (d, $^2J_{CF}$ = 25.3 Hz), 103.1 (d, $^2J_{CF}$ = 25.4 Hz), 102.6 (d, $^2J_{CF}$ = 25.1 Hz), 102.4 (d, $^2J_{CF}$ = 25.1 Hz), 77.6, 77.3, 40.7, 24.0. ^{11}B NMR ($CDCl_3$, 128 MHz): *δ* -0.5 (s, 1B), -3.0 (d, *J* = 176.6 Hz, 1B), -4.6 (d, *J* = 169.0 Hz, 1B), -11.3 (m, 7B). ^{19}F NMR ($CDCl_3$, 376 MHz): *δ* -108.7 (m, 2F), -110.0 (m, 2F). HRMS (DART) Calcd for $C_{23}H_{26}{}^{10}B_2{}^{11}B_8F_4N^+$ [M+H$^+$]: 500.2999, Found: 500.2991.

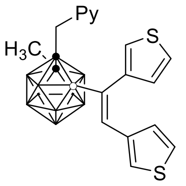

1-(2-Picolyl)-2-methyl-4-(*cis*-1,2-di(3-thienyl)vinyl)-*o*-carborane (4bq) was prepared as a white solid, following the general procedure (GP-3) and purified by column chromatography on silica gel (200-300 mesh) with an eluent (*n*-hexane/ethyl acetate = 10/1) (151.9 mg, 69%). Mp: 167.4-168.3 °C. ^{1}H NMR (400 MHz, $CDCl_3$): *δ* 8.50 (d, *J* = 4.0 Hz, 1H), 7.60 (td, *J* = 7.6, 1.6 Hz, 1H), 7.42 (dd, *J* = 4.8, 2.8 Hz, 1H), 7.19 (m, 2H), 7.08 (m, 2H), 7.01 (dd, *J* = 5.2, 1.2 Hz, 1H), 6.97 (m, 1H), 6.89 (d, *J* = 8.0 Hz, 1H), 6.57 (dd, *J* = 5.2, 1.2 Hz, 1H), 3.99 (d, *J* = 15.6 Hz, 1H), 3.51 (d, *J* = 15.2 Hz, 1H), 2.31 (s, 3H). ^{13}C{^{1}H} NMR ($CDCl_3$, 101 MHz): *δ* 156.1, 149.2, 143.8, 139.5, 137.0, 136.7, 128.7, 128.2, 126.0, 125.7, 125.1, 124.8, 122.9, 121.3, 77.4, 76.6, 40.7, 24.0. ^{11}B NMR ($CDCl_3$, 128 MHz): *δ* 0.6 (s, 1B), -3.2 (d, *J* = 162.6 Hz, 1B), -4.6 (d, *J* = 153.6 Hz, 1B), -11.6 (m, 7B). HRMS (DART) Calcd for $C_{19}H_{26}{}^{10}B_2{}^{11}B_8S_2N^+$ [M+H$^+$]: 440.2504, Found: 440.2501.

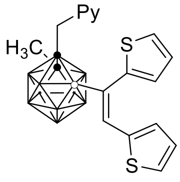

1-(2-Picolyl)-2-methyl-4-(*cis*-1,2-di(2-thienyl)vinyl)-*o*-carborane (4br) was prepared as a white solid, following the general procedure (GP-3) and purified by column chromatography on silica gel (200-300

mesh) with an eluent (*n*-hexane/ethyl acetate = 10/1) (149.7 mg, 68%). Mp: 181.2-181.9 °C. ^{1}H NMR (400 MHz, $CDCl_3$): δ 8.51 (d, J = 4.8 Hz, 1H), 7.63 (td, J = 8.0, 1.6 Hz, 1H), 7.45 (d, J = 5.2 Hz, 1H), 7.41 (s, 1H), 7.16 (m, 4H), 7.06 (d, J = 3.6 Hz, 1H), 6.92 (m, 1H), 6.89 (m, 1H), 4.08 (d, J = 15.2 Hz, 1H), 3.58 (d, J = 15.2 Hz, 1H), 2.30 (s, 3H). $^{13}C\{^1H\}$ NMR ($CDCl_3$, 101 MHz): δ 156.0, 149.3, 143.6, 140.8, 138.5, 136.8, 130.7, 128.3, 127.9, 126.1, 126.0, 125.2, 122.9, 77.6, 76.3, 40.9, 24.0. ^{11}B NMR ($CDCl_3$, 128 MHz): δ 0.3 (s, 1B), -3.2 (d, J = 147.6Hz, 1B), -4.4 (d, J = 148.5 Hz, 1B), -11.7 (m, 7B). HRMS (DART) Calcd for $C_{19}H_{26}{}^{10}B_2{}^{11}B_8S_2N^+$ [M+H$^+$]: 440.2504, Found: 440.2500.

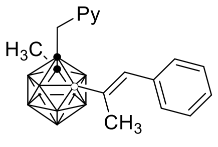

1-(2-Picolyl)-2-methyl-4-(*cis*-1-methyl-2-phenylvinyl)-*o*-carborane (4bs) was prepared as a white solid, following the general procedure (GP-3) and purified by column chromatography on silica gel (200-300 mesh) with an eluent (*n*-hexane/ethyl acetate = 10/1) (135.5 mg, 74%). Mp: 87.3-87.9 °C. ^{1}H NMR (400 MHz, $CDCl_3$): δ 8.52 (d, J = 4.4 Hz, 1H), 7.61 (t, J = 7.6 Hz, 1H), 7.37 (m, 4H), 7.24 (m, 1H), 7.17 (m, 2H), 7.0 (s, 1H), 3.83 (d, J = 15.2 Hz, 1H), 3.56 (d, J = 15.2 Hz, 1H), 2.34 (s, 3H), 2.09 (s, 3H). $^{13}C\{^1H\}$ NMR ($CDCl_3$, 101 MHz): δ 155.8, 149.3, 140.2, 138.7, 136.7, 129.2, 128.2, 126.8, 125.2, 122.9, 77.7, 76.1, 40.9, 24.1, 20.6. ^{11}B NMR ($CDCl_3$, 128 MHz): δ 1.2 (s, 1B), -3.4 (d, J = 154.9 Hz, 1B), -4.8 (d, J = 156.2 Hz, 1B), -11.5 (m, 7B). HRMS (DART) Calcd for $C_{18}H_{28}{}^{10}B_2{}^{11}B_8N^+$ [M+H$^+$]: 366.3219, Found: 366.3215.

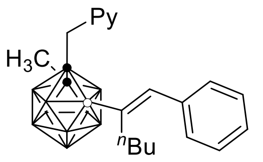

1-(2-Picolyl)-2-methyl-4-(*cis*-1-*tert*-butyl-2-phenylvinyl)-*o*-carborane (4bt) was prepared as a white solid, following the general procedure (GP-3) and purified by column chromatography on silica gel (200-300 mesh) with an eluent (*n*-hexane/ethyl acetate = 10/1) (118.4 mg, 58%). Mp: 85.3-84.2 °C. ^{1}H NMR (400 MHz, $CDCl_3$): δ 8.51 (d, J = 3.2 Hz, 1H), 7.62 (t, J = 6.8 Hz, 1H), 7.34 (m, 4H), 7.24 (m, 1H), 7.18 (m, 2H), 7.0 (s, 1H), 3.89 (d, J = 15.2 Hz, 1H), 3.55 (d, J = 15.2 Hz, 1H), 2.46 (m, 2H), 2.34 (s, 3H), 1.62 (m, 2H), 1.37 (m, 2H), 0.91 (t, J = 7.2 Hz, 3H). ^{13}C{^{1}H} NMR ($CDCl_3$, 101 MHz): δ 156.0, 149.3, 141.3, 138.7, 136.7, 128.8, 128.3, 126.9, 125.1, 122.9, 77.6, 76.1, 40.9, 33.2, 31.6, 24.1, 23.2, 14.1. ^{11}B NMR ($CDCl_3$, 128 MHz): δ 1.0 (s, 1B), -3.2 (d, J = 170.2 Hz, 1B), -4.8 (d, J = 154.9 Hz, 1B), -11.6 (m, 7B). HRMS (DART) Calcd for $C_{21}H_{34}{}^{10}B_2{}^{11}B_8N^+$ [M+H$^+$]: 408.3689, Found: 408.3685.

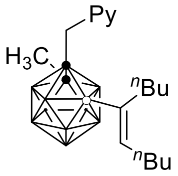

1-(2-Picolyl)-2-methyl-4-(*cis*-1,2-di-*tert*-butylvinyl)-*o*-carborane (4bu) was prepared as a colorless oil, following the general procedure (GP-3) and purified by column chromatography on silica gel (200-300 mesh) with an eluent (*n*-hexane/ethyl acetate = 10/1) (165.1 mg, 85%). ^{1}H NMR (400 MHz, $CDCl_3$): δ 8.51 (d, J = 3.2 Hz, 1H), 7.66 (t, J = 6.8 Hz, 1H), 7.18 (m, 2H), 5.97 (t, J = 6.4 Hz, 1H), 3.84 (d, J = 15.6 Hz, 1H), 3.48 (d, J = 14.8 Hz, 1H), 2.32 (s, 3H), 2.26 (m, 2H), 2.20 (dd, J = m, 2H), 1.41 (m, 8H), 0.94 (m, 6H). ^{13}C{^{1}H} NMR ($CDCl_3$, 101 MHz): δ 156.3, 149.3, 143.7, 136.6, 125.0, 122.8, 77.5, 75.8, 40.7, 32.6, 32.0, 31.8, 29.0, 24.1, 23.3, 22.7, 14.21, 14.20. ^{11}B NMR ($CDCl_3$, 128 MHz): δ 1.3 (s, 1B), -3.4 (d, J = 151.0 Hz, 1B), -4.8 (d, J = 169.0 Hz, 1B), -11.8 (m, 7B). HRMS (DART) Calcd for

$C_{19}H_{38}{}^{10}B_2{}^{11}B_8N^+$ [M+H$^+$]: 388.4002, Found: 388.3998.

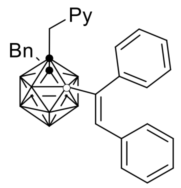

1-(2-Picolyl)-2-benzyl-4-(*cis*-1,2-diphenylvinyl)-*o*-carborane (4ca) was prepared as a white solid, following the general procedure (GP-3) and purified by column chromatography on silica gel (200-300 mesh) with an eluent (*n*-hexane/ethyl acetate = 10/1) (229.5 mg, 91%). Mp: 154.0-154.8 °C. ^{1}H NMR (400 MHz, $CDCl_3$): δ 8.54 (d, J = 4.0 Hz, 1H), 7.56 (td, J = 7.6, 1.6 Hz, 1H), 7.35 (m, 2H), 7.27 (m, 6H), 7.21 (m, 4H), 7.11 (m, 3H), 7.0 (m, 2H), 6.74 (d, J = 7.6 Hz, 1H), 4.23 (d, J = 14.8 Hz, 1H), 4.06 (d, J = 15.6 Hz, 1H), 3.77 (m, 2H). ^{13}C{^{1}H} NMR ($CDCl_3$, 101 MHz): δ 156.2, 149.2, 143.3, 142.4, 137.5, 136.9, 136.2, 130.5, 129.8, 129.1, 128.8, 128.6, 128.0, 127.8, 127.4, 126.5, 125.1, 123.0, 81.6, 79.7, 40.7, 40.5. ^{11}B NMR ($CDCl_3$, 128 MHz): δ 0.7 (s, 1B), -3.4 (d, J = 90.9 Hz, 2B), -11.4 (m, 7B). HRMS (DART) Calcd for $C_{29}H_{34}{}^{10}B_2{}^{11}B_8N^+$ [M+H$^+$]: 504.3689, Found: 504.3691.

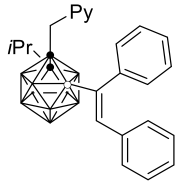

1-(2-Picolyl)-2-*iso*-propyl-4-(*cis*-1,2-diphenylvinyl)-*o*-carborane (4da) was prepared as a white solid, following the general procedure (GP-3) and purified by column chromatography on silica gel (200-300 mesh) with an eluent (*n*-hexane/ethyl acetate = 10/1) (214.5 mg, 94%). Mp: 124.8-125.6 °C. ^{1}H NMR (400 MHz, $CDCl_3$): δ 8.46 (d, J = 4 Hz, 1H), 7.53 (td, J = 7.6, 2.0 Hz, 1H), 7.34 (m, 2H), 7.26 (m, 3H), 7.16 (m, 2H), 7.11 (m, 3H), 6.98 (m, 2H), 6.73 (d, J = 8.0 Hz, 1H), 3.95 (d, J = 15.6 Hz, 1H), 3.51 (d, J = 15.6 Hz, 1H), 3.21 (m, 1H), 1.23 (d, J = 6.8 Hz, 3H), 1.12 (d, J = 6.8 Hz, 3H). ^{13}C{^{1}H} NMR ($CDCl_3$, 101 MHz): δ 156.0, 149.1, 143.3, 142.3, 137.6, 136.6, 129.7, 129.1, 128.8, 128.0, 127.3, 126.5, 125.2, 122.8, 88.6, 82.0, 40.3, 30.9, 24.5, 24.4. ^{11}B NMR ($CDCl_3$, 128 MHz): δ 0.3 (s, 1B), -2.8 (d, J

= 160. Hz, 1B), -4.2 (d, *J* = 160 Hz, 1B), -10.8 (m, 7B). HRMS (DART) Calcd for $C_{25}H_{34}{}^{10}B_2{}^{11}B_8N^+$ [M+H$^+$]: 456.3689, Found: 456.3685.

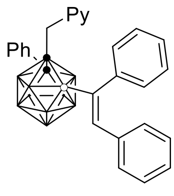

1-(2-Picolyl)-2-phenyl-4-(*cis*-1,2-diphenylvinyl)-*o*-carborane (4ea) was prepared as a white solid, following the general procedure (GP-3) and purified by column chromatography on silica gel (200-300 mesh) with an eluent (*n*-hexane/ethyl acetate = 10/1) (235.4mg, 96%). Mp: 166.5-167.3 °C. ^{1}H NMR (400 MHz, $CDCl_3$): δ 8.49 (d, *J* = 3.6 Hz, 1H), 7.67 (d, *J* = 7.6 Hz, 2H), 7.42 (m, 9H), 7.23 (s, 1H), 7.14 (s, 4H), 7.03 (m, 2H), 6.57 (d, *J* = 7.6 Hz, 1H), 3.83 (d, *J* = 15.2 Hz, 1H), 2.97 (d, *J* = 15.2 Hz, 1H). $^{13}C\{^1H\}$ NMR ($CDCl_3$, 101 MHz): δ 155.4, 149.4, 143.4, 142.2, 137.6, 135.9, 131.9, 130.8, 129.8, 129.0, 128.8, 128.76, 128.0, 127.2, 126.4, 125.0, 122.5, 84.7, 81.5, 39.9. ^{11}B NMR ($CDCl_3$, 128 MHz): δ 0.7 (s, 1B), -2.3 (d, *J* = 115.2 Hz, 2B), -11.0 (m, 7B). HRMS (DART) Calcd for $C_{28}H_{32}{}^{10}B_2{}^{11}B_8N^+$ [M+H$^+$]: 490.3532, Found: 490.3528.

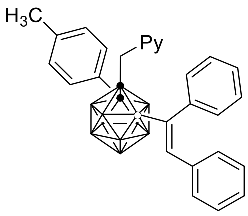

1-(2-Picolyl)-2-(4-methylphenyl)-4-(*cis*-1,2-diphenylvinyl)-*o*-carborane (4fa) was prepared as a white solid, following the general procedure (GP-3) and purified by column chromatography on silica gel (200-300 mesh) with an eluent (*n*-hexane/ethyl acetate = 10/1) (237.1 mg, 94%). Mp: 196.0-196.8 °C. ^{1}H NMR (400 MHz, $CDCl_3$): δ 8.48 (d, *J* = 4.4 Hz, 1H), 7.54 (d, *J* = 8.4 Hz, 2H), 7.45 (td, *J* = 8.0, 1.6 Hz, 1H), 7.37 (m, 2H), 7.30 (m, 3H), 7.20 (d, *J* = 6.8 Hz, 3H), 7.13 (m, 4H), 7.0 (m, 2H), 6.61 (d, *J* = 7.6 Hz, 1H), 3.79 (d, *J* = 15.2 Hz, 1H), 2.94 (d, *J* = 14.8 Hz, 1H), 2.40 (s, 3H). $^{13}C\{^1H\}$ NMR ($CDCl_3$, 101 MHz): δ 155.5, 149.3, 143.5, 142.1, 141.2, 137.7,

135.9, 131.7, 129.7, 129.5, 128.9, 128.7, 128.0, 127.2, 126.4, 125.0, 122.4, 85.1, 81.6, 39.8, 21.2. ^{11}B NMR ($CDCl_3$, 128 MHz): δ 0.5 (s, 1B), -2.5 (d, J = 105.0 Hz, 2B), -11.0 (m, 7B). HRMS (DART) Calcd for $C_{29}H_{33}{}^{10}B_2{}^{11}B_8N^+$ $C_{29}H_{34}{}^{10}B_2{}^{11}B_8N^+$ [M+H$^+$]: 504.3689, Found: 504.3687.

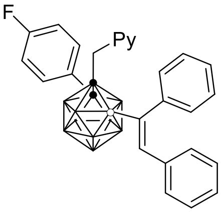

1-(2-Picolyl)-2-(4-fluorophenyl)-4-(*cis*-1,2-diphenylvinyl)-*o*-carborane (4ga) was prepared as a white solid, following the general procedure (GP-3) and purified by column chromatography on silica gel (200-300 mesh) with an eluent (*n*-hexane/ethyl acetate = 10/1) (241.5 mg, 95%). Mp: 174.1-175.0 °C. ^{1}H NMR (400 MHz, $CDCl_3$): δ 8.45 (d, J = 4.4 Hz, 1H), 7.65 (dd, J = 8.4, 5.2 Hz, 2H), 7.47 (t, J = 7.6 Hz, 1H), 7.37 (m, 2H), 7.31 (d, J = 6.8 Hz, 3H), 7.20 (s, 1H), 7.09 (m, 6H), 7.03 (m, 2H), 6.63 (d, J = 7.6 Hz, 1H), 3.80 (d, J = 14.8 Hz, 1H), 2.93 (d, J = 15.2 Hz, 1H). $^{13}C\{^1H\}$ NMR ($CDCl_3$, 101 MHz): δ 164.2 (d, $^1J_{CF}$ = 253.5 Hz), 155.4, 149.4, 143.3, 142.4, 137.6, 136.0, 134.0 (d, $^3J_{CF}$ = 8.1 Hz), 129.8, 129.0, 128.8, 128.0, 127.3, 126.9 (d, $^4J_{CF}$ = 3.0 Hz), 126.5, 125.0, 122.6, 115.8 (d, $^2J_{CF}$ =21.2 Hz), 84.0, 81.6, 39.9. ^{11}B NMR ($CDCl_3$, 128 MHz): δ 0.8 (s, 1B), -2.4 (d, J = 116.5 Hz, 2B), -11.1 (m, 7B). ^{19}F NMR ($CDCl_3$, 376 MHz): δ -109.5 (m, 1F). HRMS (DART) Calcd for $C_{28}H_{31}{}^{10}B_2{}^{11}B_8FN^+$ [M+H$^+$]: 508.3438, Found: 508.3433.

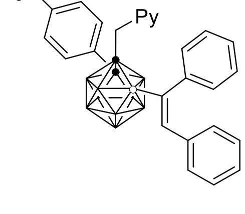

1-(2-Picolyl)-2-(4-trifluoromethylphenyl)-4-(*cis*-1,2-diphenylvinyl)-*o*-carborane (4ha) was prepared as a white solid, following the general procedure (GP-3) and purified by column chromatography on silica gel (200-300 mesh) with an eluent (*n*-hexane/ethyl acetate =

10/1) (268.0 mg, 96%). Mp: 163.2-163.9 °C. ^{1}H NMR (400 MHz, $CDCl_3$): δ 8.28 (d, J = 3.6 Hz, 1H), 7.66 (d, J = 8.4 Hz, 2H), 7.51 (d, J = 8.2 Hz, 2H), 7.32 (m, 1H), 7.22 (m, 5H), 7.09 (s, 1H), 7.00 (m, 4H), 6.88 (m, 2H), 6.53 (d, J = 7.6, 1H), 3.68 (d, J = 14.8 Hz, 1H), 2.83 (d, J = 15.2 Hz, 1H). ^{13}C{^{1}H} NMR ($CDCl_3$, 101 MHz): δ 155.0, 149.3, 143.2, 142.6, 137.5, 136.1, 134.5, 132.7 (q, $^{2}J_{CF}$ = 32.7 Hz), 132.4, 129.8, 129.0, 128.9, 128.1, 127.4, 126.6, 125.7 (q, $^{3}J_{CF}$ = 4.0 Hz), 125.0, 123.6 (q, $^{1}J_{CF}$ = 271.3 Hz), 122.7, 84.0, 81.6, 39.9. ^{11}B NMR ($CDCl_3$, 128 MHz): δ 1.0 (s, 1B), -2.0 (m, 2B), -10.7 (m, 7B). ^{19}F NMR ($CDCl_3$, 376 MHz): δ -62.9 (s, 1F). HRMS (DART) Calcd for $C_{29}H_{31}{}^{10}B_2{}^{11}B_8F_3N^+$ [M+H$^+$]: 558.3406, Found: 558.3398.

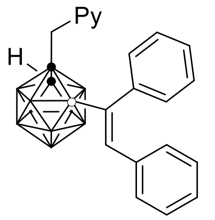

1-(2-Picolyl)-4-(*cis*-1,2-diphenylvinyl)-*o*-carborane (4aa) was prepared as a colorless oil, following the general procedure (GP-3) and purified by column chromatography on silica gel (200-300 mesh) with an eluent (*n*-hexane/ethyl acetate = 10/1) (134.2 mg, 65%). ^{1}H NMR (400 MHz, $CDCl_3$): δ 8.51 (d, J = 4.0 Hz, 1H), 7.60 (d, J = 8.0, 1.6 Hz, 2H), 7.34 (m, 2H), 7.26 (m, 2H), 7.21 (m, 2H), 7.12 (m, 4H), 6.98 (m, 2H), 6.91 (d, J = 7.6 Hz, 1H), 4.25 (s, 1H), 3.63 (m, 2H). ^{13}C{^{1}H} NMR ($CDCl_3$, 101 MHz): δ 155.5, 149.8, 143.4, 142.0, 137.5, 137.3, 129.8, 128.9, 128.8, 128.0, 127.3, 126.5, 124.9, 123.0, 73.2, 58.6, 42.3. ^{11}B NMR ($CDCl_3$, 128 MHz): δ -1.4 (s, 2B), -4.8 (s, 1B), -11.4 (m, 7B). HRMS (DART) Calcd for $C_{22}H_{28}{}^{10}B_2{}^{11}B_8N^+$ [M+H$^+$]: 414.3219, Found: 414.3215.

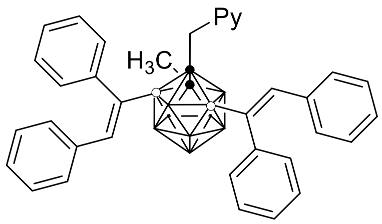

1-(2-Picolyl)-2-methyl-3,5-bis(*cis*-1,2-diphenylvinyl)-*o*-carborane (3ba) was prepared as a white solid, following the general procedure (GP-4) and purified by column chromatography on silica gel (200-300 mesh) with an eluent (*n*-hexane/ethyl acetate = 10/1) (181.9 mg, 60%). Single crystals suitable for X-ray analyses were obtained by slow evaporation of an ether acetate solution over a period of 3 days at ambient conditions. Mp: 157.1-157.9 °C. ^{1}H NMR (400 MHz, $CDCl_3$): δ 8.51 (d, J = 4.0 Hz, 1H), 7.40 (m, 5H), 7.32 (m, 1H), 7.24 (m, 4H), 7.11 (m, 8H), 6.57 (m, 7H), 4.00 (d, J = 15.2 Hz, 1H), 3.78 (d, J = 15.2 Hz, 1H), 2.00 (s, 1H). $^{13}C\{^1H\}$ NMR ($CDCl_3$, 101 MHz): δ 155.0, 148.9, 144.1, 143.6, 141.2, 140.5, 137.6, 137.0, 136.0, 129.9, 129.8, 129.7, 129.6, 128.5, 128.4, 128.0, 127.9, 127.6, 127.1, 126.9, 126.2, 122.7, 76.7, 75.8, 41.2, 22.7. ^{11}B NMR ($CDCl_3$, 128 MHz): δ -1.7 (s, 1B), -3.8 (m, 3B), -10.6 (m, 6B). HRMS (ESI) Calcd for $C_{37}H_{40}{}^{10}B_2{}^{11}B_8N^+$ [M+H$^+$]: 606.4158, Found: 606.4175.

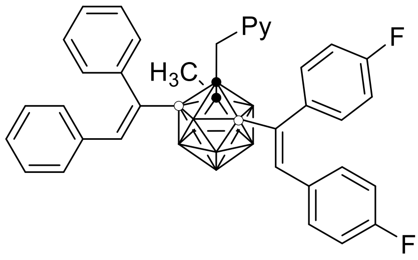

1-(2-Picolyl)-2-methyl-3-(*cis*-1,2-diphenylvinyl)-5-(*cis*-1,2-di(4-fluorophenyl)vinyl)-*o*-carborane (5a) was prepared as a white solid, following the general procedure (GP-4) and purified by column chromatography on silica gel (200-300 mesh) with an eluent (*n*-hexane/ethyl acetate = 10/1) (189.5 mg, 59%). Mp: 87.8-88.4 °C. ^{1}H NMR (400 MHz, $CDCl_3$): δ 8.46 (d, J = 4.8 Hz, 1H), 7.40 (td, J = 8.0, 2.0 Hz, 1H), 7.23 (m, 5H), 7.15 (d, J = 8.0, 1H), 7.07 (m, 7H), 6.94 (s, 1H), 6.82 (m, 8H), 3.93 (d, J = 15.6 Hz, 1H), 3.71 (d, J = 15.6 Hz, 1H),

1.92 (s, 3H). $^{13}C\{^1H\}$ NMR ($CDCl_3$, 101 MHz): δ 162.0 (d, $^1J_{CF}$ = 247.3 Hz), 161.9 (d, $^1J_{CF}$ = 244.8 Hz), 154.8, 148.9, 143.4 (d, $^3J_{CF}$ = 9.6 Hz), 140.9, 137.5, 136.8 (d, $^4J_{CF}$ = 3.5 Hz), 136.1, 133.0 (d, $^4J_{CF}$ = 3.3 Hz), 131.5 (d, $^3J_{CF}$ = 7.9 Hz), 129.7, 128.5, 128.0, 127.2, 126.8, 126.3, 122.8, 115.8 (d, $^2J_{CF}$ = 21.0 Hz), 115.0 (d, $^2J_{CF}$ = 21.3 Hz), 77.6, 75.7, 41.2, 22.8. ^{11}B NMR ($CDCl_3$, 128 MHz): δ -3.7 (m, 4B), -10.2 (m, 6B). ^{19}F NMR ($CDCl_3$, 376 MHz): δ -113.1 (m, 1F), -115.2 (m, 1F). HRMS (ESI) Calcd for $C_{37}H_{38}{}^{10}B_2{}^{11}B_8F_2N^+$ [M+H$^+$]: 642.3970, Found: 642.3986.

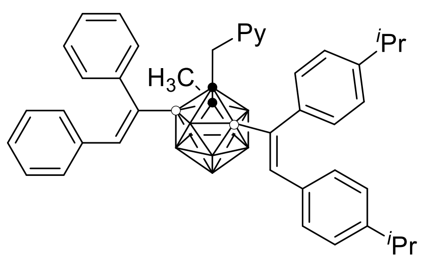

1-(2-Picolyl)-2-methyl-3-(*cis*-1,2-diphenylvinyl)-5-(*cis*-1,2-di(4-*iso*-propylphenyl)vinyl)-*o*-carborane (5b) was prepared as a white solid, following the general procedure (GP-4) and purified by column chromatography on silica gel (200-300 mesh) with an eluent (*n*-hexane/ethyl acetate = 10/1) (183.0 mg, 53%). Mp: 71.7-72.5 ºC. ^1H NMR (400 MHz, $CDCl_3$): δ 8.46 (d, J = 4.0 Hz, 1H), 7.36 (td, J = 7.6, 1.6 Hz, 1H), 7.20 (m, 7H), 7.08 (m, 6H), 6.95 (d, J = 8.0 Hz, 2H), 6.38 (m, 7H), 3.95 (d, J = 15.6 Hz, 1H), 3.74 (d, J = 15.2 Hz, 1H), 2.94 (m, 1H), 2.79 (m, 1H), 1.96 (s, 3H), 1.28 (d, J = 6.8 Hz, 6H), 1.17 (d, J = 6.8 Hz, 6H). $^{13}C\{^1H\}$ NMR ($CDCl_3$, 101 MHz): δ 155.1, 148.9, 148.6, 147.6, 143.9, 143.6, 140.4, 138.7, 137.7, 135.9, 134.7, 130.0, 129.7, 129.6, 128.6, 128.4, 128.0, 127.0, 126.8, 126.7, 126.1, 122.7, 76.6, 75.8, 41.3, 33.9, 24.3, 23.9, 22.7. ^{11}B NMR ($CDCl_3$, 128 MHz): δ -3.5 (m, 4B), -10.5 (m, 6B). HRMS (ESI) Calcd for $C_{43}H_{52}{}^{10}B_2{}^{11}B_8N^+$ [M+H$^+$]: 690.5097, Found: 690.5115.

X-ray Structure Determination. The data of **3aj** (213 K), **3ba** (213 K) and **4ba** (223 K) were collected at different temperatures on a Bruker APEX DUO diffractometer. An empirical absorption correction was applied using the SADABS program.[4] All structures were solved by direct methods and subsequent Fourier difference techniques and refined anisotropically for all non-hydrogen atoms by full-matrix least-squares on F^2 using the SHELXTL program package.[5] All hydrogen atoms were geometrically fixed using the riding model. Crystal data and details of data collection and structure refinements were given in Table S1.

CCDC 2299007-2299009 (**3aj**, **3ba** and **4ba**) contains the supplementary crystallographic data for this paper. These data can be obtained free of charge from The Cambridge Crystallographic Data Centre via www.ccdc.cam.ac.uk/data_request/cif.

Table S1. Crystal Data and Summary of Data Collection and Refinements.

compound	**3aj·C_6H_6**	**3ba**	**4ba**
formula	$C_{46}H_{39}B_{10}F_{12}N$	$C_{37}H_{39}B_{10}N$	$C_{23}H_{29}B_{10}N$
crystal size (mm)	0.07 x 0.07 x 0.05	0.08 x 0.07 x 0.07	0.07 x 0.07 x 0.05
fw	941.88	605.79	427.57
crystal system	Monoclinic	Monoclinic	Triclinic
space group	$P2_1/n$	$P2_1/c$	P-1
a, Å	15.776(1)	13.825(1)	8.174(1)
b, Å	10.776(1)	12.076(1)	11.970(1)
c, Å	27.171(1)	21.026(1)	13.598(1)
α, deg	90	90	108.438 (1)
β, deg	94.006(2)	90.115(1)	94.043(1)
γ, deg	90	90	99.404(1)
V, Å^3	4607.8(3)	3510.3(1)	1234.43(3)
Z	4	4	2
D_{calcd}, Mg/m^3	1.358	1.146	1.150
radiation (λ) Å	1.34139	1.34139	1.34139
2θ range, deg	5.5 to 110.1	5.6 to 111.0	6.91 to 110.0
μ, mm^{-1}	0.594	0.286	0.277
F(000)	1920	1272	448
no. of obsd reflns	8763	6639	4641
no. of params refnd	610	434	363
goodness of fit	1.032	1.033	1.048
R1	0.0944	0.0544	0.0504
wR2	0.2542	0.1321	0.1324

References

1 Alekseyeva, E. S.; Batsanov, A. S.; Boyd, L. A.; Fox, M. A.; Hibbert, T. G.; Howard, J. A. K.; MacBride, J. A. H.; Mackinnon, A.; Wade, K. *Dalton Trans.* **2003**, 475-482.

2 Zhang, H.; Cheng, R.; Qiu, Z.; Xie, Z. *Chem. Commun.* **2023**, *59*, 740-743.

3 Park, K.; Bae, G.; Moon, J.; Choe, J.; Song, K. H.; Lee, S. *J. Org. Chem*. **2010**, *75*, 6244-6251.

4 Sheldrick, G. M. SADABS: Program for Empirical Absorption Correction of Area Detector Data. University of Göttingen: Germany, **1996**.

5 Sheldrick, G. M. SHELXTL 5.10 for Windows NT: Structure Determination Software Programs. Bruker Analytical X-ray Systems, Inc., Madison, Wisconsin, USA, **1997**.

^{1}H NMR (400 MHz, $CDCl_3$) of **1a**

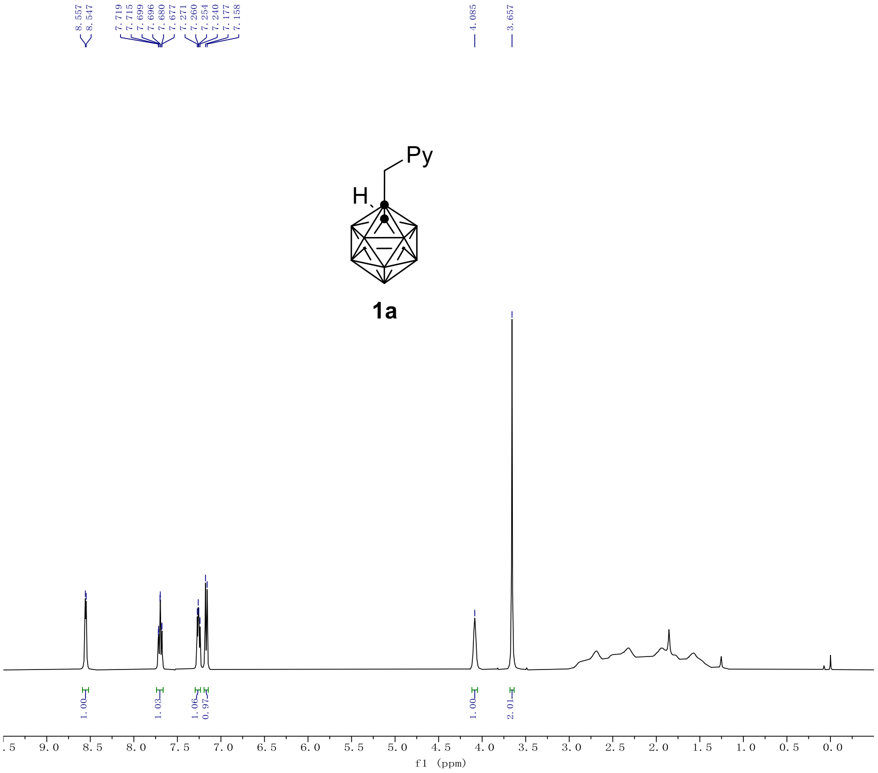

Parameter	Value
Data File Name	E:/ 核磁/ yss-6-1001-h.fid/ fid
Title	yss-6-1001-h
Comment	Std proton
Origin	Varian
Owner	
Site	
Spectrometer	vnmrs
Author	omc1
Solvent	CDCl3
Temperature	25.0
Pulse Sequence	s2pul
Experiment	1D
Probe	ATB
Number of Scans	4
Receiver Gain	24
Relaxation Delay	1.0000
Pulse Width	0.0000
Presaturation Frequency	
Acquisition Time	3.0000
Acquisition Date	2023-09-30T16:46:47
Modification Date	2023-09-30T08:47:00
Class	
Spectrometer Frequency	400.03
Spectral Width	8445.9
Lowest Frequency	-1214.4
Nucleus	1H
Acquired Size	25338
Spectral Size	65536

^{1}H NMR (400 MHz, $CDCl_3$) of **1b**

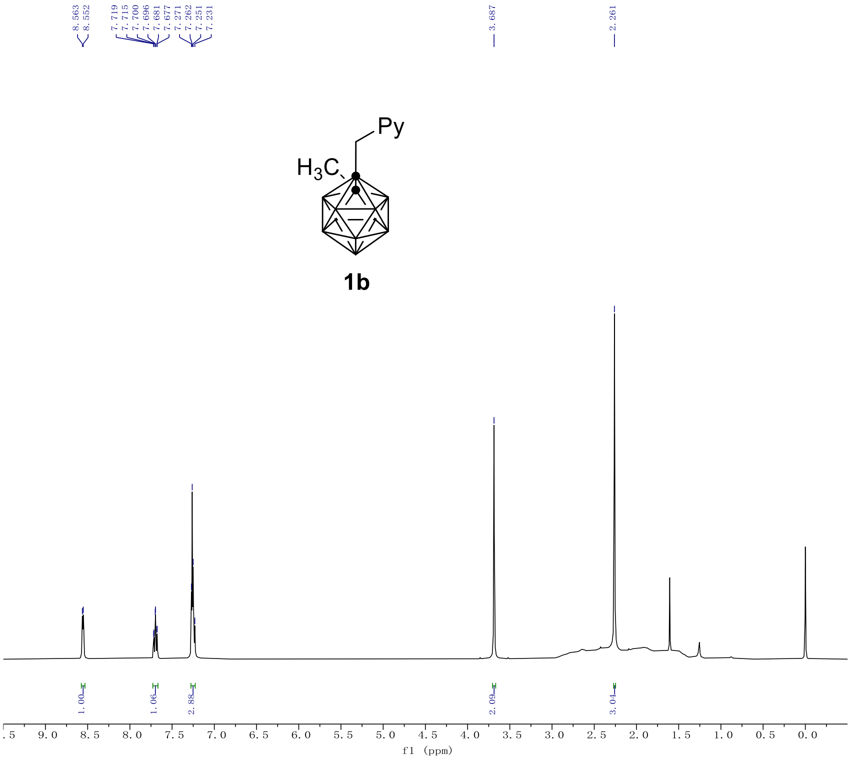

Parameter	Value
Data File Name	E:/ 核磁/ yss-1013-GZ-H/ 28/ fid
Title	yss-1013-GZ-H/ 28
Comment	
Origin	Bruker BioSpin GmbH
Owner	nmr
Site	
Spectrometer	spect
Author	
Solvent	CDCl3
Temperature	296.9
Pulse Sequence	zg30
Experiment	1D
Probe	Z116098_0640 (PA BBO 400S1 BBF-H-D-05 Z SP)
Number of Scans	8
Receiver Gain	77
Relaxation Delay	1.0000
Pulse Width	10.0000
Presaturation Frequency	
Acquisition Time	2.9998
Acquisition Date	2022-08-27T19:36:58
Modification Date	2022-08-27T19:36:59
Class	
Spectrometer Frequency	400.20
Spectral Width	7598.8
Lowest Frequency	-1207.3
Nucleus	1H
Acquired Size	22795
Spectral Size	65536

^{1}H NMR (400 MHz, $CDCl_3$) of **1c**

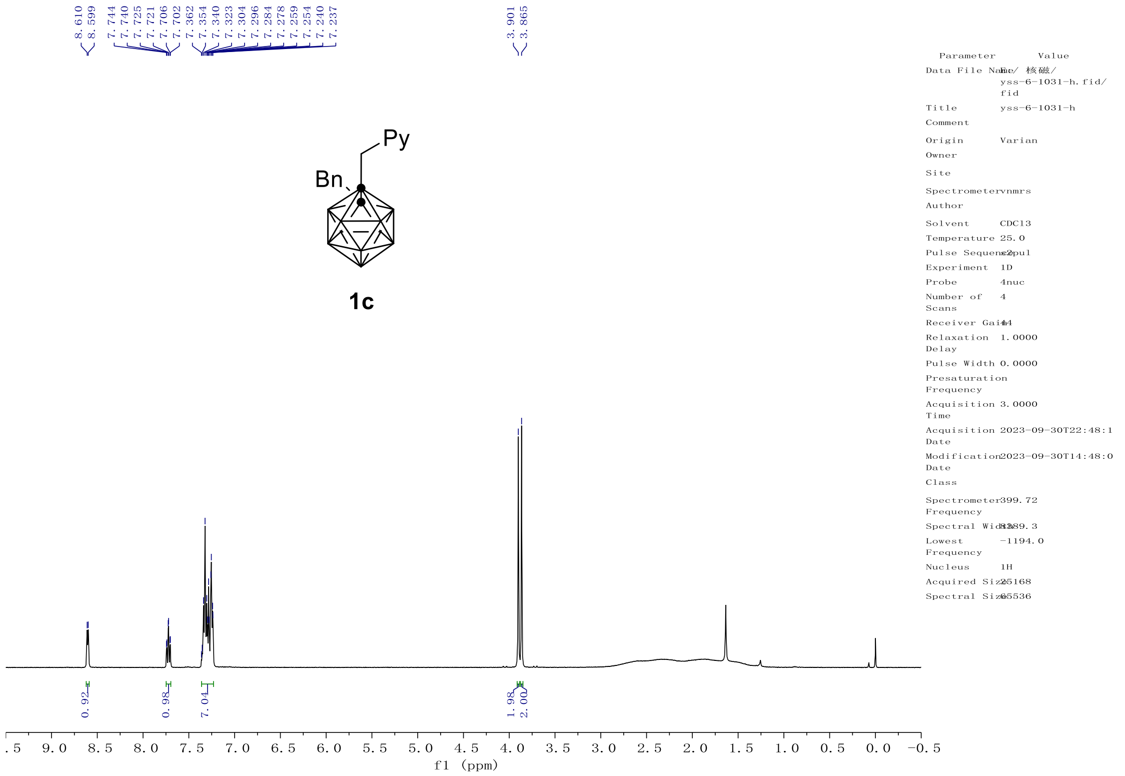

^{1}H NMR (400 MHz, $CDCl_3$) of **1d**

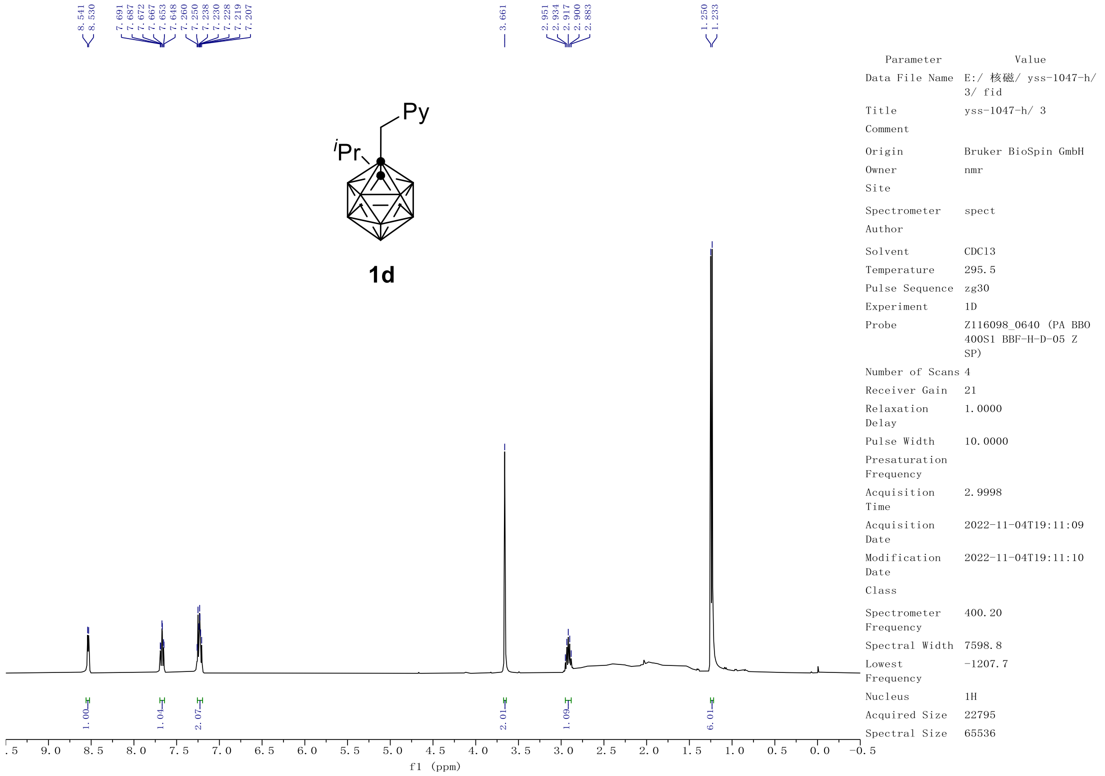

^{1}H NMR (400 MHz, $CDCl_3$) of **1e**

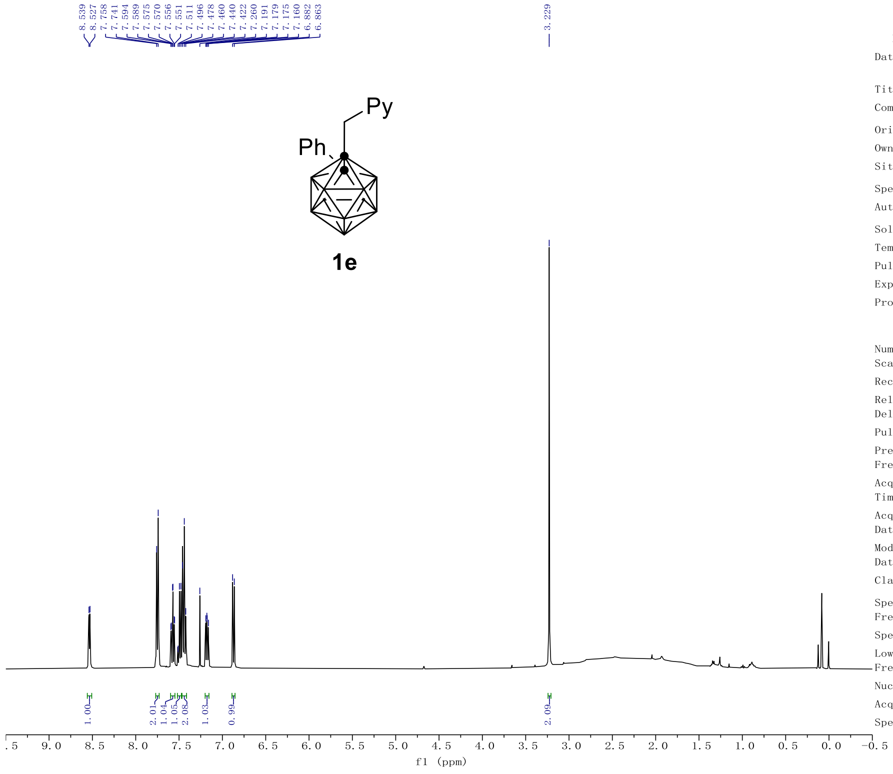

Parameter	Value
Data File Name	E:/ 核磁/ yss-1036-h-gz/ 7/ fid
Title	yss-1036-h-gz/ 7
Comment	
Origin	Bruker BioSpin GmbH
Owner	nmr
Site	
Spectrometer	spect
Author	
Solvent	CDCl3
Temperature	295.9
Pulse Sequence	zg30
Experiment	1D
Probe	Z116098_0640 (PA BBO 400S1 BBF-H-D-05 Z SP)
Number of Scans	7
Receiver Gain	36
Relaxation Delay	1.0000
Pulse Width	10.0000
Presaturation Frequency	
Acquisition Time	2.9998
Acquisition Date	2022-10-23T18:59:10
Modification Date	2022-10-23T18:59:10
Class	
Spectrometer Frequency	400.20
Spectral Width	7598.8
Lowest Frequency	-1207.7
Nucleus	1H
Acquired Size	22795
Spectral Size	65536

^{1}H NMR (400 MHz, $CDCl_3$) of **1f**

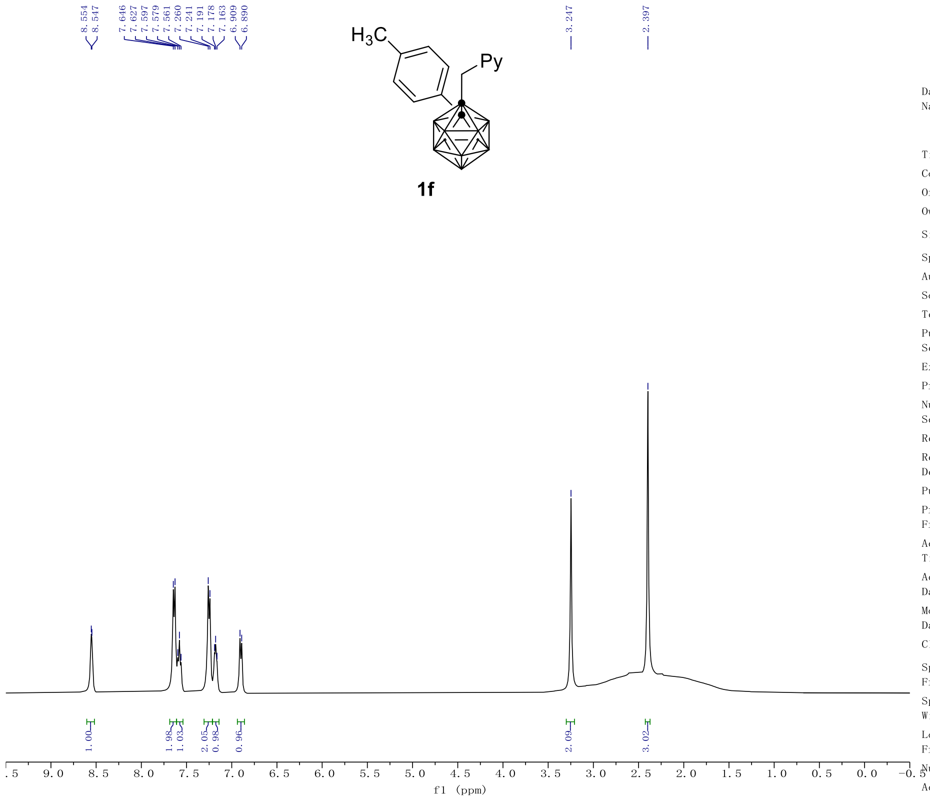

Parameter	Value
Data File Name	E:/ 积分核磁/ 2030/ 6-yss-2030-h.fid/ fid
Title	6-yss-2030-h
Comment	Std proton
Origin	Varian
Owner	
Site	
Spectrometer	vnmrs
Author	omc1
Solvent	CDCl3
Temperature	25.0
Pulse Sequence	s2pul
Experiment	1D
Probe	ATB
Number of Scans	4
Receiver Gain	22
Relaxation Delay	1.0000
Pulse Width	0.0000
Presaturation Frequency	
Acquisition Time	3.0000
Acquisition Date	2023-06-05T21:37:25
Modification Date	2023-06-05T13:38:00
Class	
Spectrometer Frequency	400.03
Spectral Width	10080.6
Lowest Frequency	-2023.6
Nucleus	1H
Acquired Size	30242

^{13}C{^{1}H} NMR (101 MHz, $CDCl_3$) of **1f**

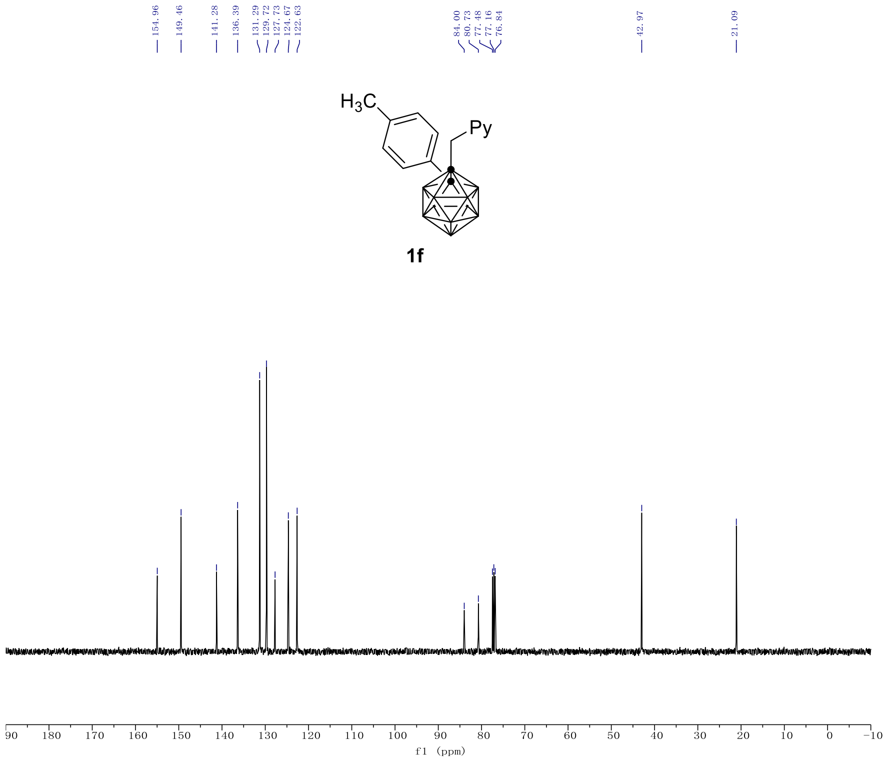

Parameter	Value
Data File Name	E:/ 积分核磁/ 2030/ yss-2030-c/ 2/ fid
Title	yss-2030-c/ 2
Comment	
Origin	Bruker BioSpin GmbH
Owner	nmr
Site	
Spectrometer	spect
Author	
Solvent	CDCl3
Temperature	296.0
Pulse Sequence	zgpg30
Experiment	1D
Probe	Z116098_0640 (PA BBO 400S1 BBF-H-D-05 Z SP)
Number of Scans	14
Receiver Gain	196
Relaxation Delay	1.0000
Pulse Width	10.0000
Presaturation Frequency	
Acquisition Time	1.2999
Acquisition Date	2023-06-05T21:53:51
Modification Date	2023-06-05T21:53:53
Class	
Spectrometer Frequency	100.64
Spectral Width	25252.5
Lowest Frequency	-1565.9
Nucleus	13C
Acquired Size	32827
Spectral Size	131072

$^{11}B\{^1H\}$ NMR (128 MHz, $CDCl_3$) of **1f**

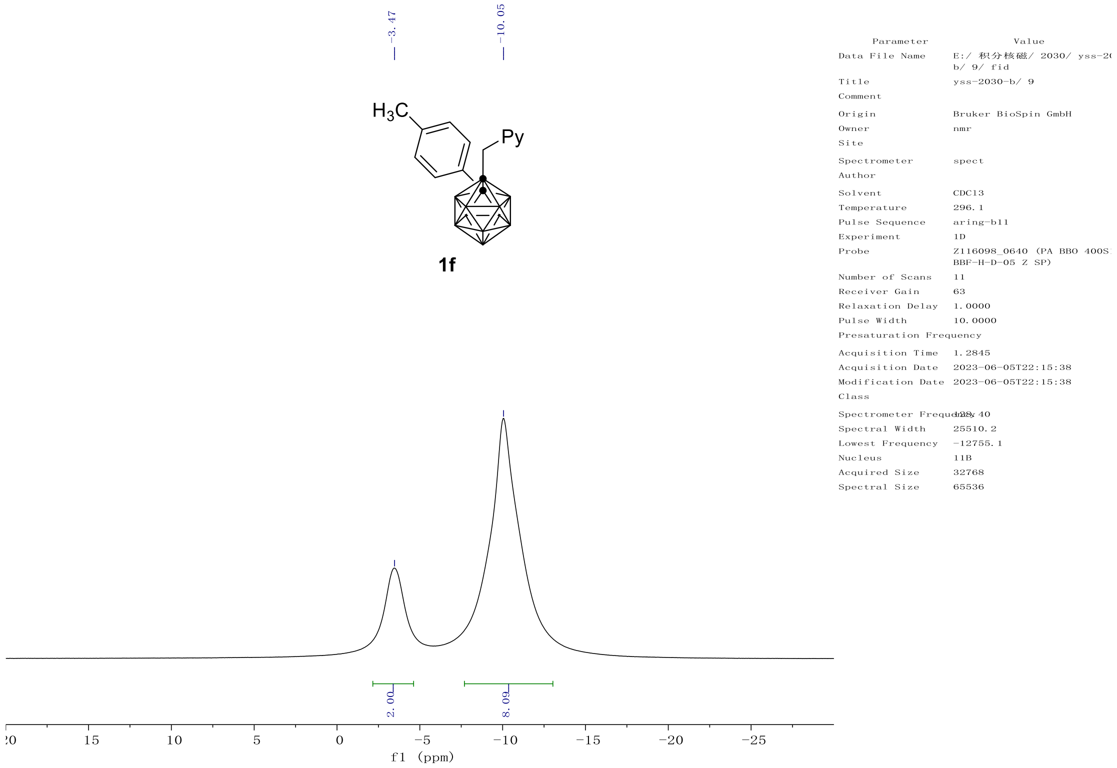

^{11}B NMR (128 MHz, $CDCl_3$) of **1f**

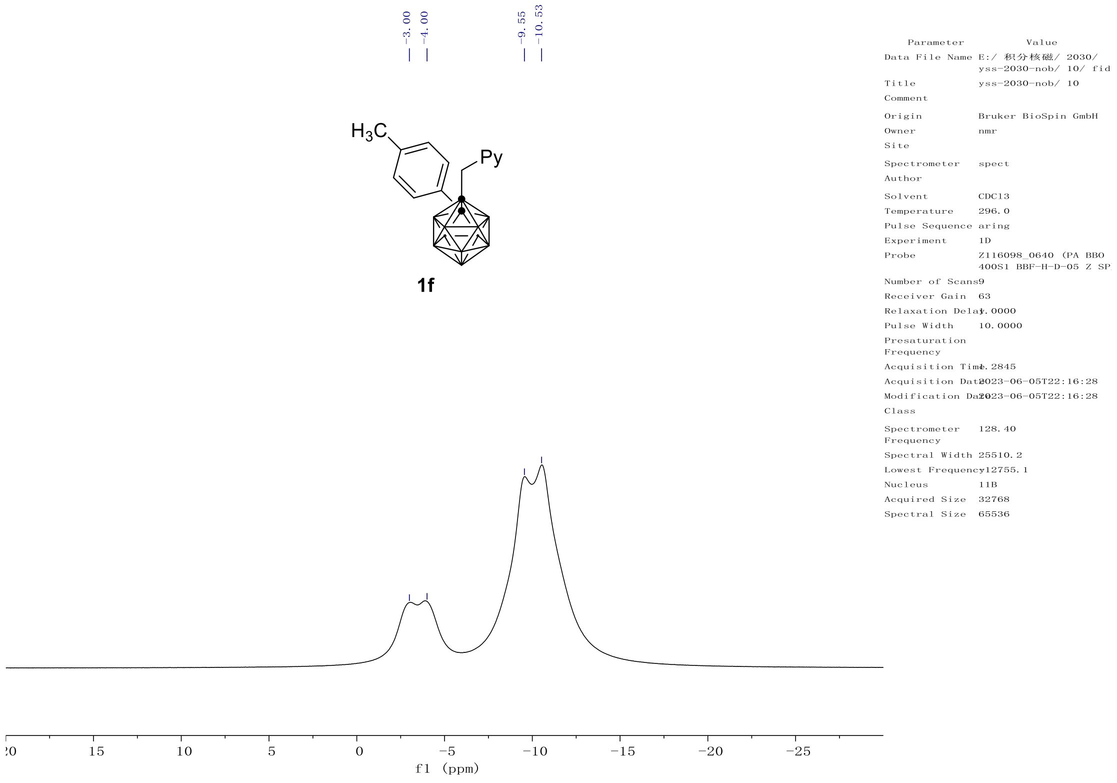

^{1}H NMR (400 MHz, $CDCl_3$) of **1g**

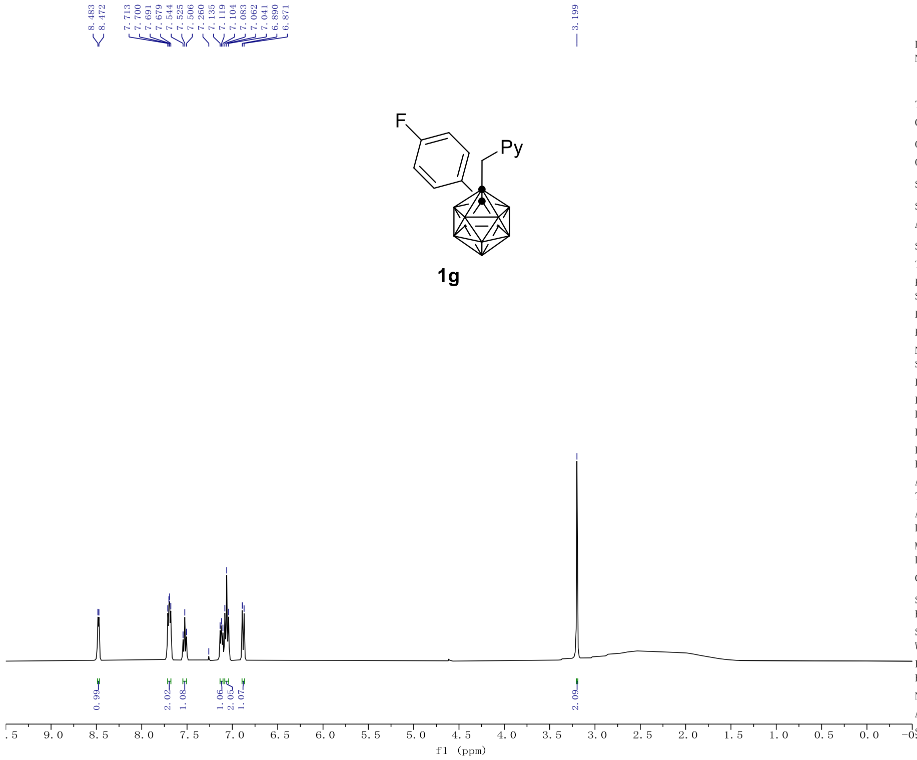

Parameter	Value
Data File Name	E:/ 积分核磁/ 2038/ yss-2038-h.fid/ fid
Title	yss-2038-h
Comment	
Origin	Varian
Owner	
Site	
Spectrometer	vnmrs
Author	
Solvent	CDCl3
Temperature	25.0
Pulse Sequence	s2pul
Experiment	1D
Probe	4nuc
Number of Scans	4
Receiver Gain	12
Relaxation Delay	1.0000
Pulse Width	0.0000
Presaturation Frequency	
Acquisition Time	2.9999
Acquisition Date	2023-05-22T16:46:38
Modification Date	2023-05-22T08:47:00
Class	
Spectrometer Frequency	399.72
Spectral Width	8012.8
Lowest Frequency	-805.1
Nucleus	1H
Acquired Size	24038
Spectral Size	65536

$^{13}C\{^{1}H\}$ NMR (101 MHz, $CDCl_3$) of **1g**

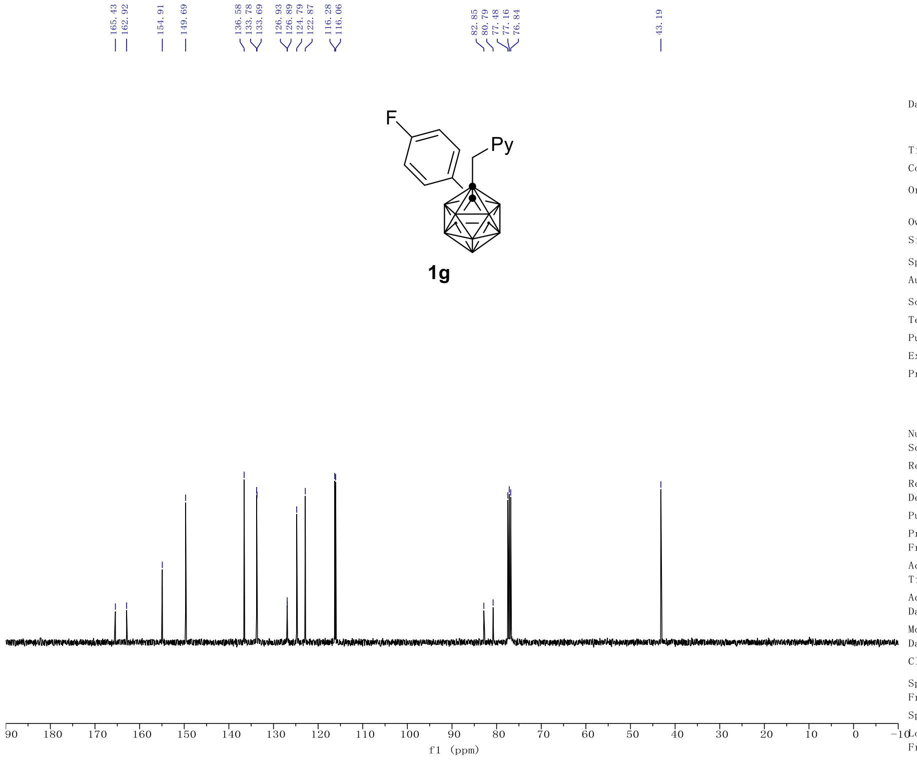

Parameter	Value
Data File Name	E:/ 积分核磁/ 2038/ yss-2038-c/ 3/ fid
Title	yss-2038-c/ 3
Comment	
Origin	Bruker BioSpin GmbH
Owner	nmr
Site	
Spectrometer	spect
Author	
Solvent	CDCl3
Temperature	296.0
Pulse Sequence	zgpg30
Experiment	1D
Probe	Z116098_0640 (PA BBO 400S1 BBF-H-D-05 Z SP)
Number of Scans	74
Receiver Gain	196
Relaxation Delay	1.0000
Pulse Width	10.0000
Presaturation Frequency	
Acquisition Time	1.2999
Acquisition Date	2023-06-05T21:58:53
Modification Date	2023-06-05T21:58:54
Class	
Spectrometer Frequency	100.64
Spectral Width	25252.5
Lowest Frequency	-1549.2

$^{11}B\{^{1}H\}$ NMR (128 MHz, $CDCl_3$) of **1g**

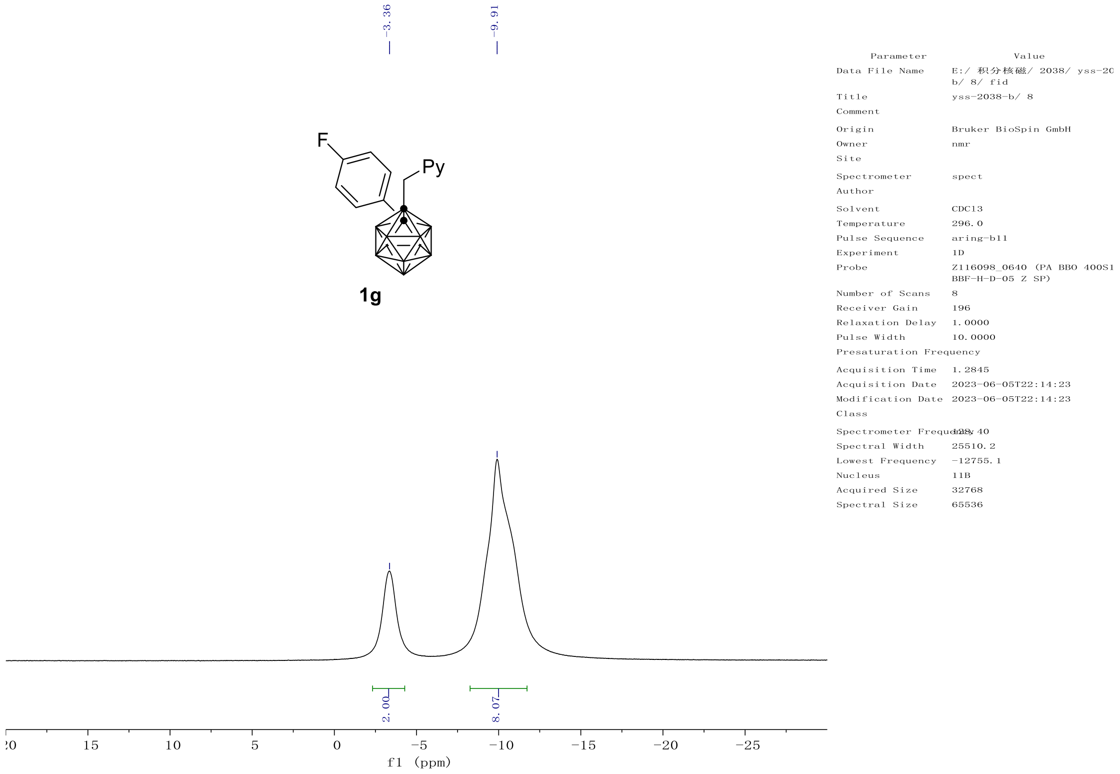

^{11}B NMR (128 MHz, $CDCl_3$) of **1g**

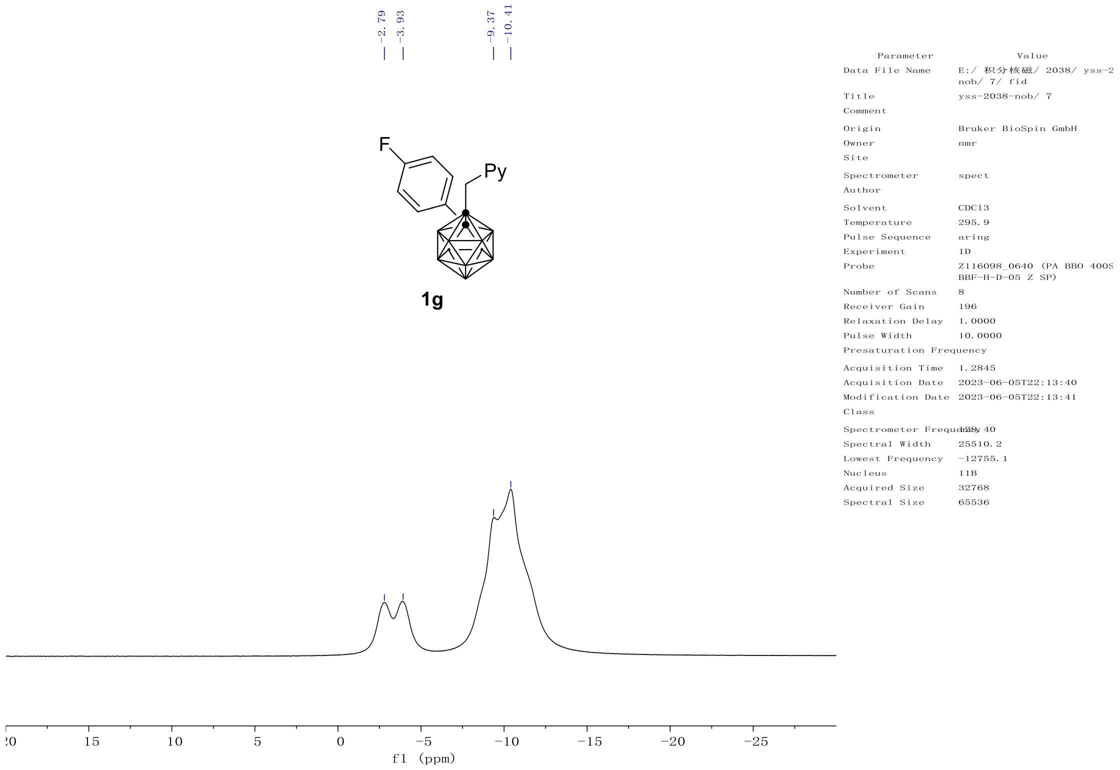

^{19}F NMR (376 MHz, $CDCl_3$) of **1g**

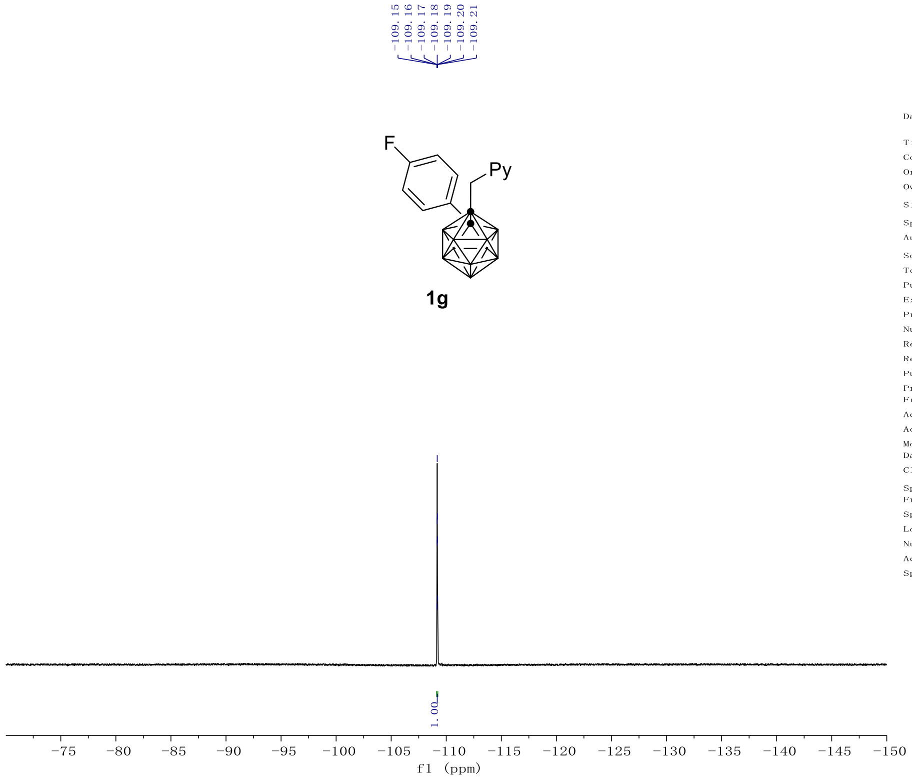

Parameter	Value
Data File Name	E:/ 积分核磁/ 2038/ yss-2038-f.fid/ fid
Title	yss-2038-f
Comment	Std Fluorine
Origin	Varian
Owner	
Site	
Spectrometer	vnmrs
Author	
Solvent	CDCl3
Temperature	25.0
Pulse Sequence	s2pul
Experiment	1D
Probe	ATB
Number of Scans	8
Receiver Gain	60
Relaxation Delay	1.0000
Pulse Width	0.0000
Presaturation Frequency	
Acquisition Time	1.0000
Acquisition Date	2023-06-05T21:38:29
Modification Date	2023-06-05T13:39:00
Class	
Spectrometer Frequency	376.37
Spectral Width	156250.0
Lowest Frequency	-115765.3
Nucleus	19F
Acquired Size	156250
Spectral Size	524288

^{1}H NMR (400 MHz, $CDCl_3$) of **1h**

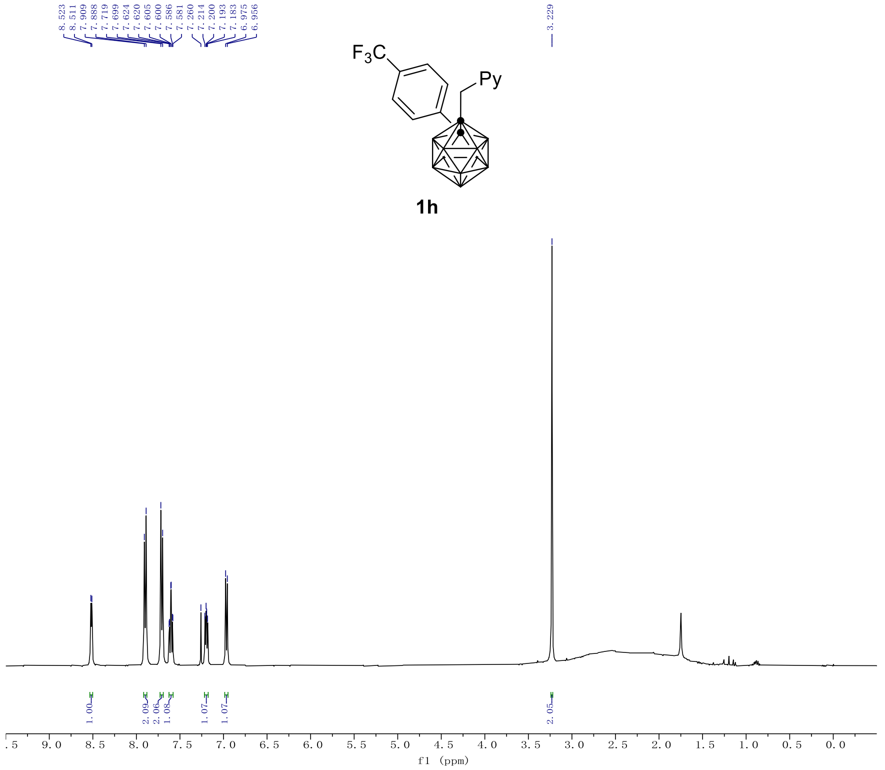

Parameter	Value
Data File Name	E:/ 积分核磁/ 2057/ yss-2057-h.fid/ fid
Title	yss-2057-h
Comment	Std proton
Origin	Varian
Owner	
Site	
Spectrometer	vnmrs
Author	omc1
Solvent	CDCl3
Temperature	25.0
Pulse Sequence	s2pul
Experiment	1D
Probe	ATB
Number of Scans	4
Receiver Gain	30
Relaxation Delay	1.0000
Pulse Width	0.0000
Presaturation Frequency	
Acquisition Time	3.0000
Acquisition Date	2023-06-30T21:22:54
Modification Date	2023-06-30T13:23:00
Class	
Spectrometer Frequency	400.03
Spectral Width	10080.6
Lowest Frequency	-2032.3
Nucleus	1H
Acquired Size	30242
Spectral Size	65536

$^{13}C\{^{1}H\}$ NMR (101 MHz, $CDCl_3$) of **1h**

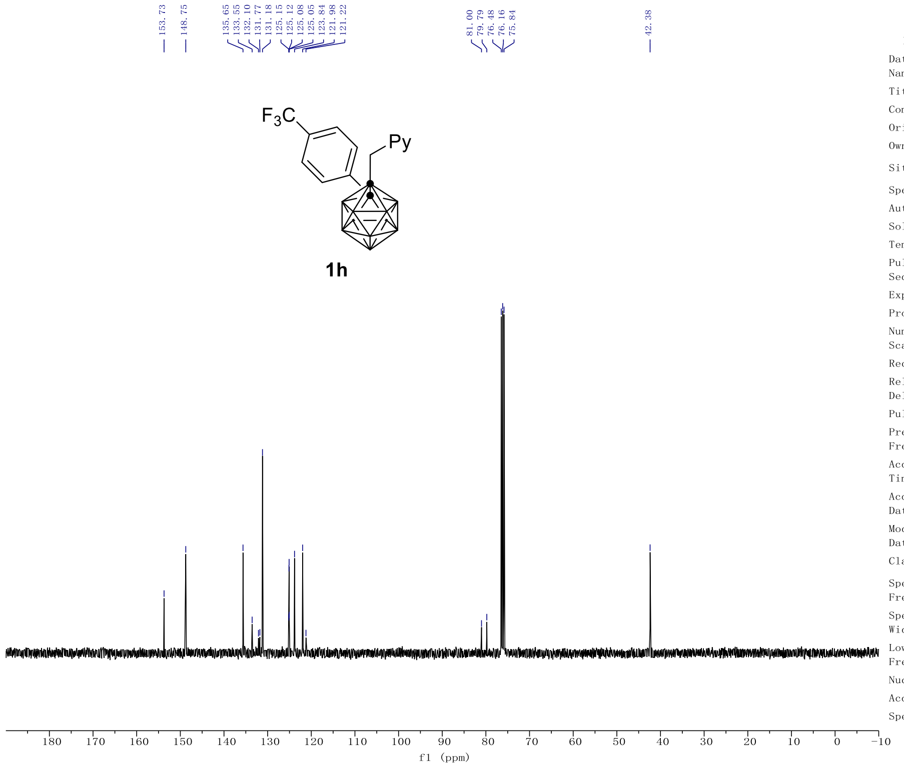

Parameter	Value
Data File Name	E:/ 积分核磁/ 2057/ yss-2057-c.fid/ fid
Title	yss-2057-c
Comment	Std carbon
Origin	Varian
Owner	
Site	
Spectrometer	vnmrs
Author	omc1
Solvent	cdcl3
Temperature	25.0
Pulse Sequence	s2pul
Experiment	1D
Probe	ATB
Number of Scans	240
Receiver Gain	60
Relaxation Delay	1.0000
Pulse Width	0.0000
Presaturation Frequency	
Acquisition Time	1.3000
Acquisition Date	2023-06-30T21:24:18
Modification Date	2023-06-30T13:33:00
Class	
Spectrometer Frequency	100.60
Spectral Width	28409.1
Lowest Frequency	-2217.0
Nucleus	13C
Acquired Size	36932
Spectral Size	131072

$^{11}B\{^1H\}$ NMR (128 MHz, $CDCl_3$) of **1h**

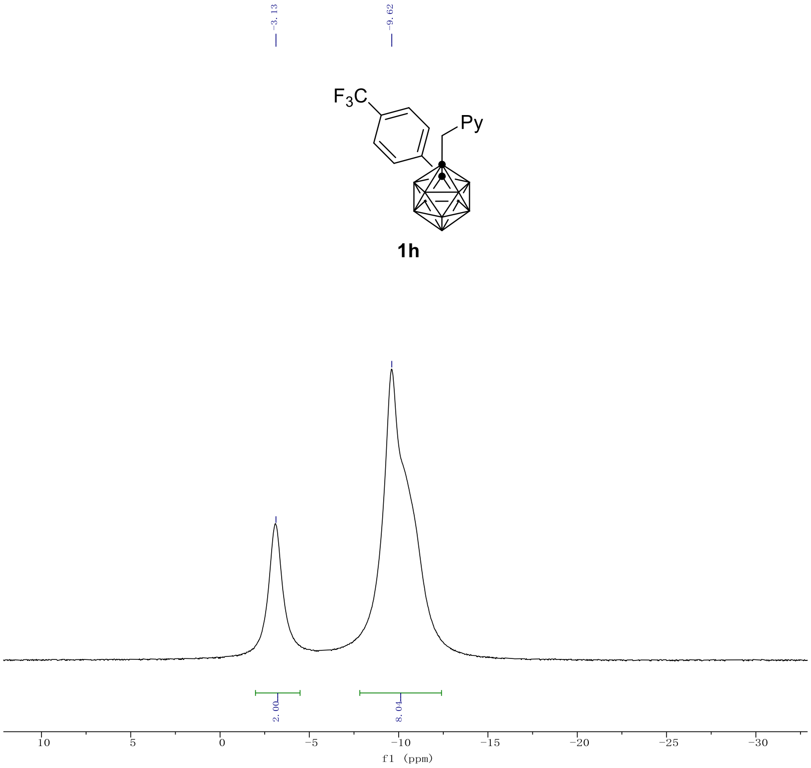

Parameter	Value
Data File Name	E:/ 积分核磁/ 2057/ yss-2057-b/ 6/ fid
Title	yss-2057-b/ 6
Comment	
Origin	Bruker BioSpin GmbH
Owner	nmr
Site	
Spectrometer	spect
Author	
Solvent	CDCl3
Temperature	296.5
Pulse Sequence	aring-b11
Experiment	1D
Probe	Z116098_0640 (PA BBO 400S1 BBF-H-D-05 Z SP)
Number of Scans	8
Receiver Gain	196
Relaxation Delay	1.0000
Pulse Width	10.0000
Presaturation Frequency	
Acquisition Time	1.2845
Acquisition Date	2023-06-30T21:52:50
Modification Date	2023-06-30T21:52:51
Class	
Spectrometer Frequency	128.40
Spectral Width	25510.2
Lowest Frequency	-12755.1
Nucleus	11B
Acquired Size	32768
Spectral Size	65536

^{11}B NMR (128 MHz, $CDCl_3$) of **1h**

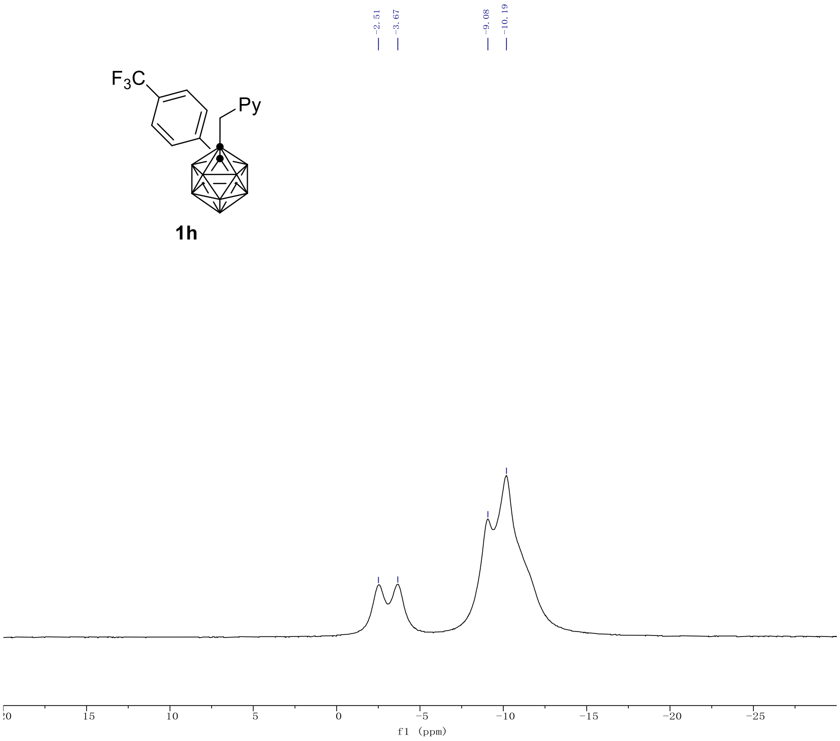

Parameter	Value
Data File Name	E:/ 积分核磁/ 2057/ yss-2057-nob/ 7/ fid
Title	yss-2057-nob/ 7
Comment	
Origin	Bruker BioSpin GmbH
Owner	nmr
Site	
Spectrometer	spect
Author	
Solvent	CDCl3
Temperature	296.5
Pulse Sequence	aring
Experiment	1D
Probe	Z116098_0640 (PA BBO 400S1 BBF-H-D-05 Z SP)
Number of Scans	8
Receiver Gain	196
Relaxation Delay	1.0000
Pulse Width	10.0000
Presaturation Frequency	
Acquisition Time	1.2845
Acquisition Date	2023-06-30T21:53:31
Modification Date	2023-06-30T21:53:33
Class	
Spectrometer Frequency	128.40
Spectral Width	25510.2
Lowest Frequency	-12755.1
Nucleus	11B
Acquired Size	32768
Spectral Size	65536

^{19}F NMR (376 MHz, $CDCl_3$) of **1h**

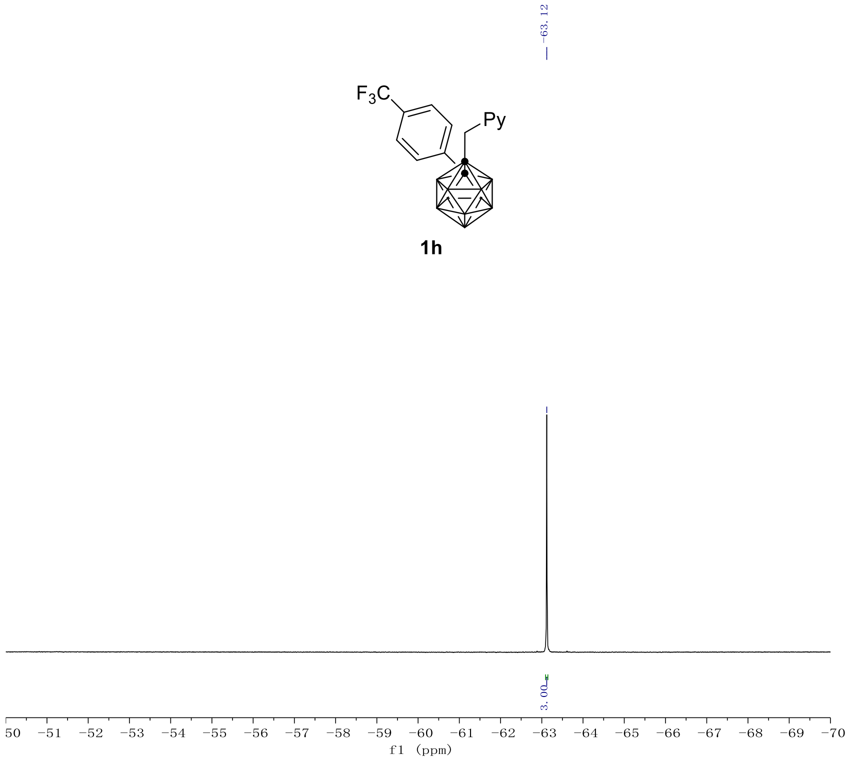

Parameter	Value
Data File Name	E:/ 积分核磁/ 2057/ yss-2057-f.fid/ fid
Title	yss-2057-f
Comment	Std Fluorine
Origin	Varian
Owner	
Site	
Spectrometer	vnmrs
Author	
Solvent	CDCl3
Temperature	25.0
Pulse Sequence	s2pul
Experiment	1D
Probe	ATB
Number of Scans	8
Receiver Gain	60
Relaxation Delay	1.0000
Pulse Width	0.0000
Presaturation Frequency	
Acquisition Time	1.0000
Acquisition Date	2023-06-30T21:33:57
Modification Date	2023-06-30T13:34:00
Class	
Spectrometer Frequency	376.37
Spectral Width	156250.0
Lowest Frequency	-115765.3
Nucleus	19F
Acquired Size	156250
Spectral Size	524288

^{1}H NMR (400 MHz, $CDCl_3$) of **3aa**

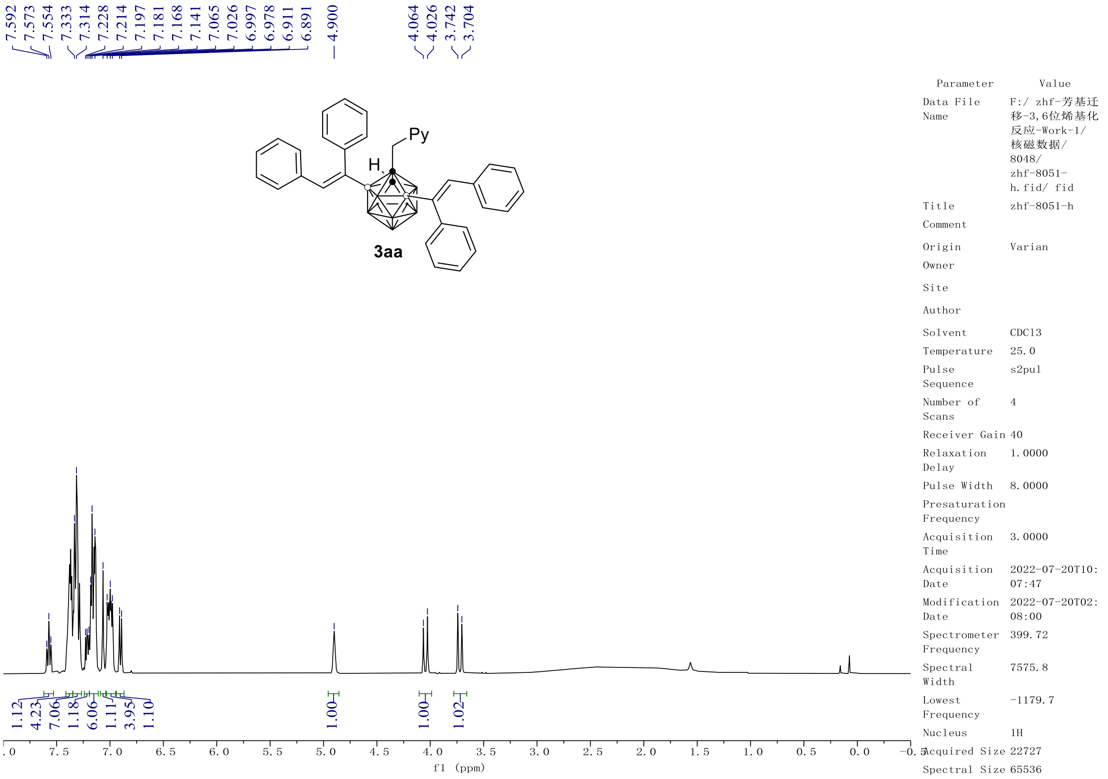

$^{13}C\{^{1}H\}$ NMR (101 MHz, $CDCl_3$) of **3aa**

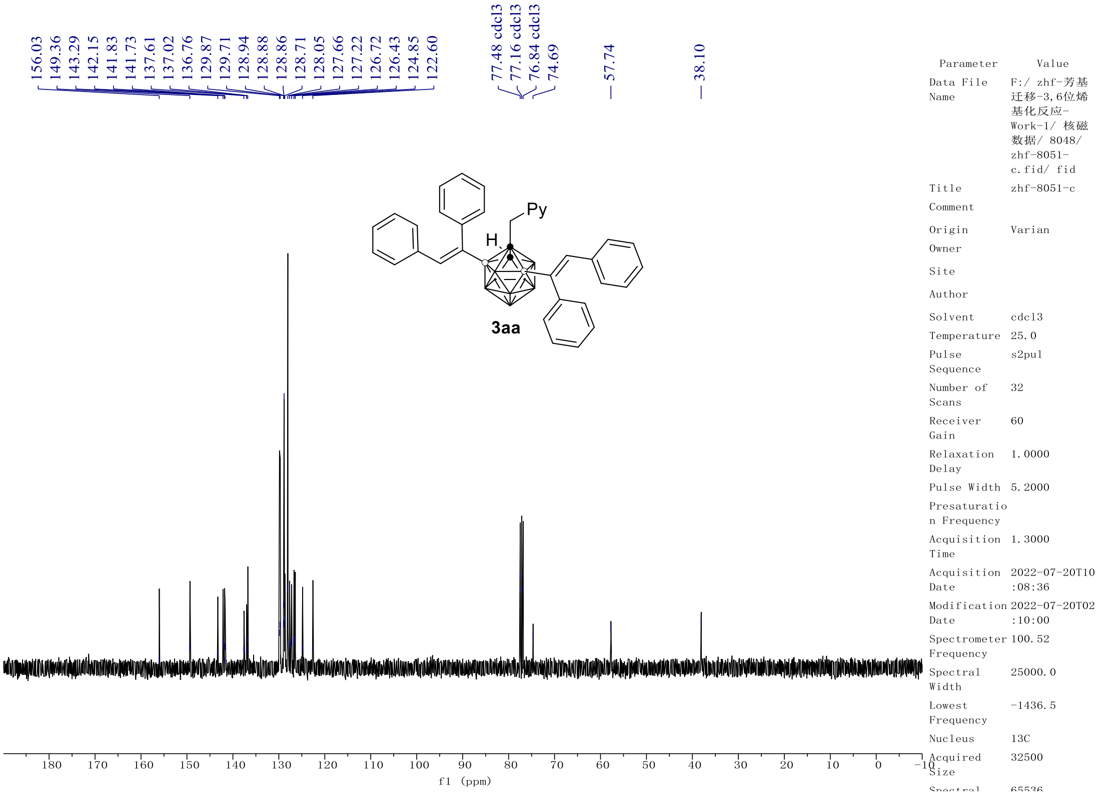

\

$^{11}B\{^1H\}$ NMR (128 MHz, $CDCl_3$) of **3aa**

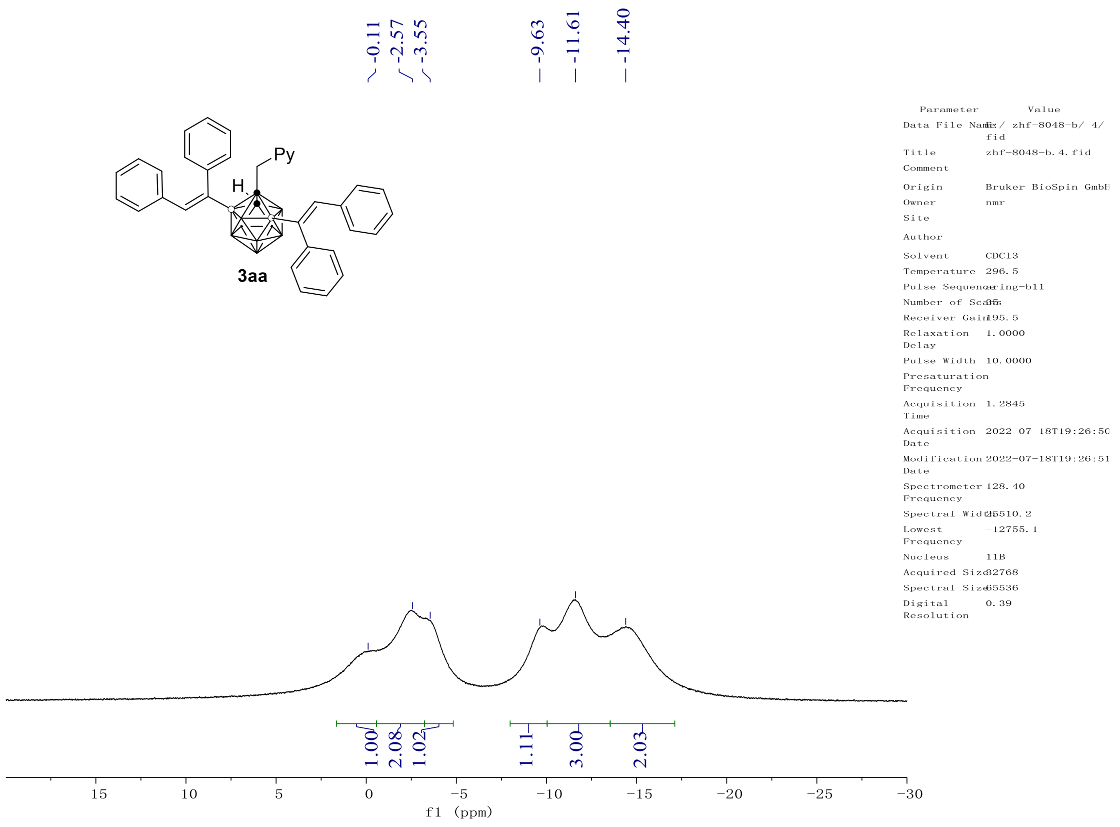

^{11}B NMR (128 MHz, $CDCl_3$) of **3aa**

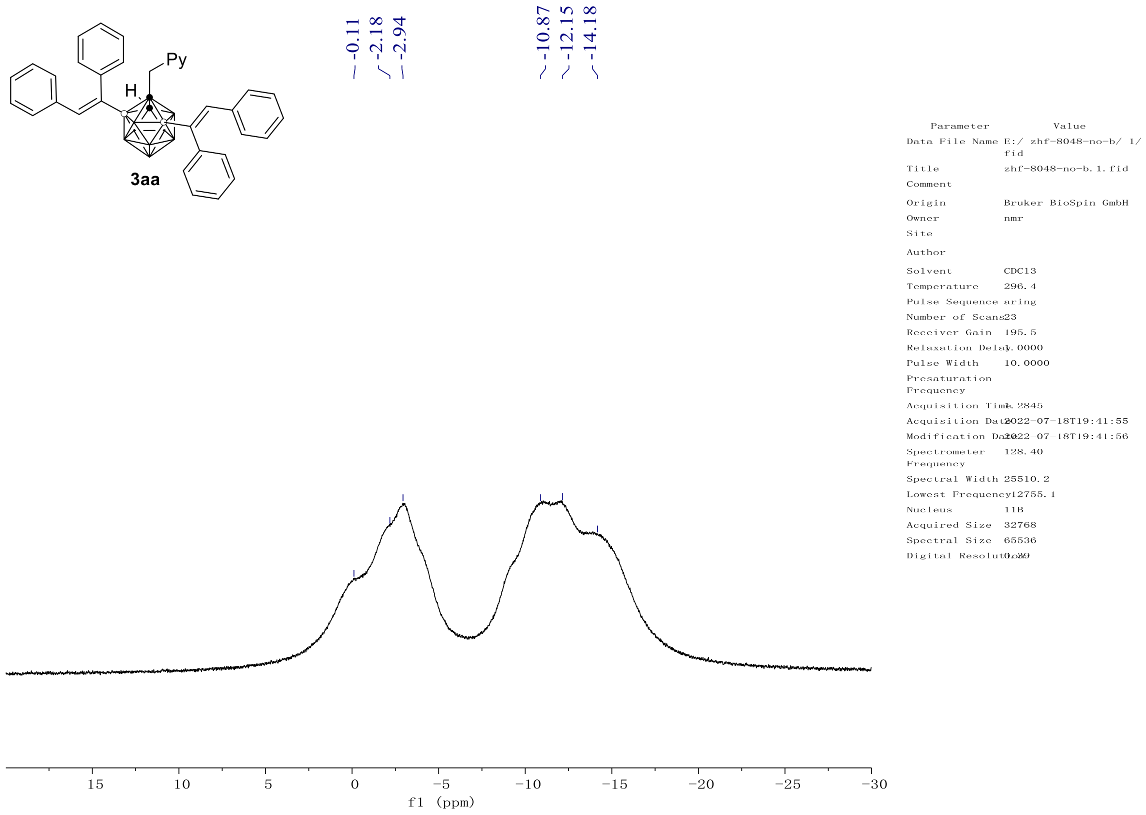

^{1}H NMR (400 MHz, $CDCl_3$) of **3ab**

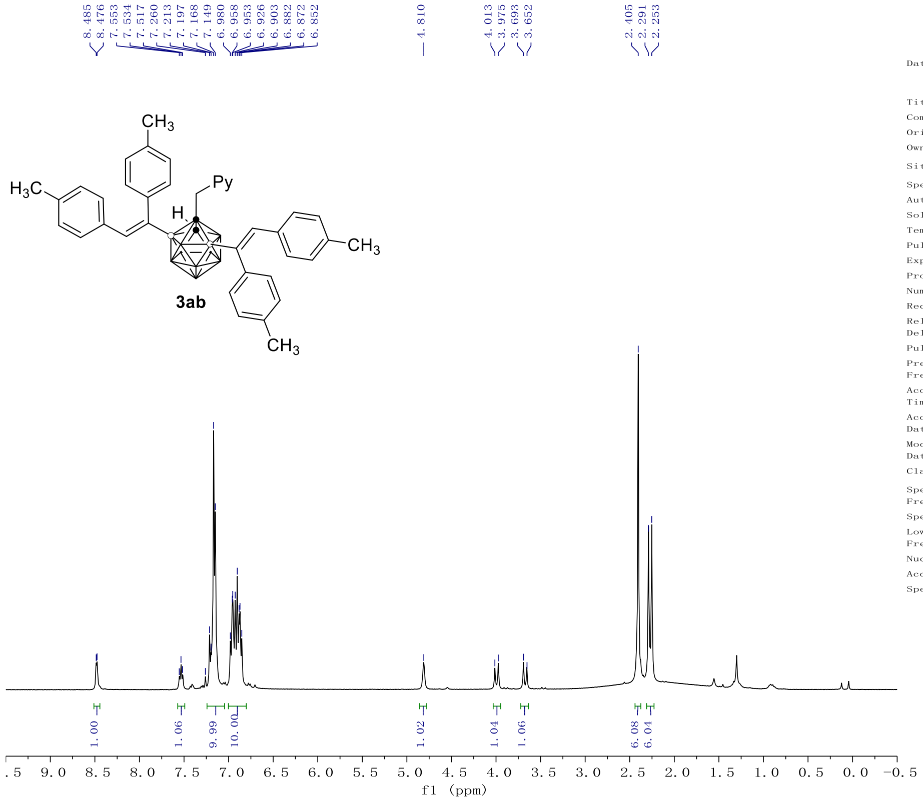

Parameter	Value
Data File Name	E:/ 积分核磁/ 2002-6yss-2002-3-h.fid/ fid
Title	6yss-2002-3-h
Comment	Std proton
Origin	Varian
Owner	
Site	
Spectrometer	vnmrs
Author	omc1
Solvent	CDCl3
Temperature	25.0
Pulse Sequence	s2pul
Experiment	1D
Probe	ATB
Number of Scans	12
Receiver Gain	32
Relaxation Delay	1.0000
Pulse Width	0.0000
Presaturation Frequency	
Acquisition Time	3.0000
Acquisition Date	2023-05-08T16:05:32
Modification Date	2023-05-08T08:06:00
Class	
Spectrometer Frequency	400.03
Spectral Width	8445.9
Lowest Frequency	-1216.0
Nucleus	1H
Acquired Size	25338
Spectral Size	65536

^{13}C{^{1}H} NMR (101 MHz, $CDCl_3$) of **3ab**

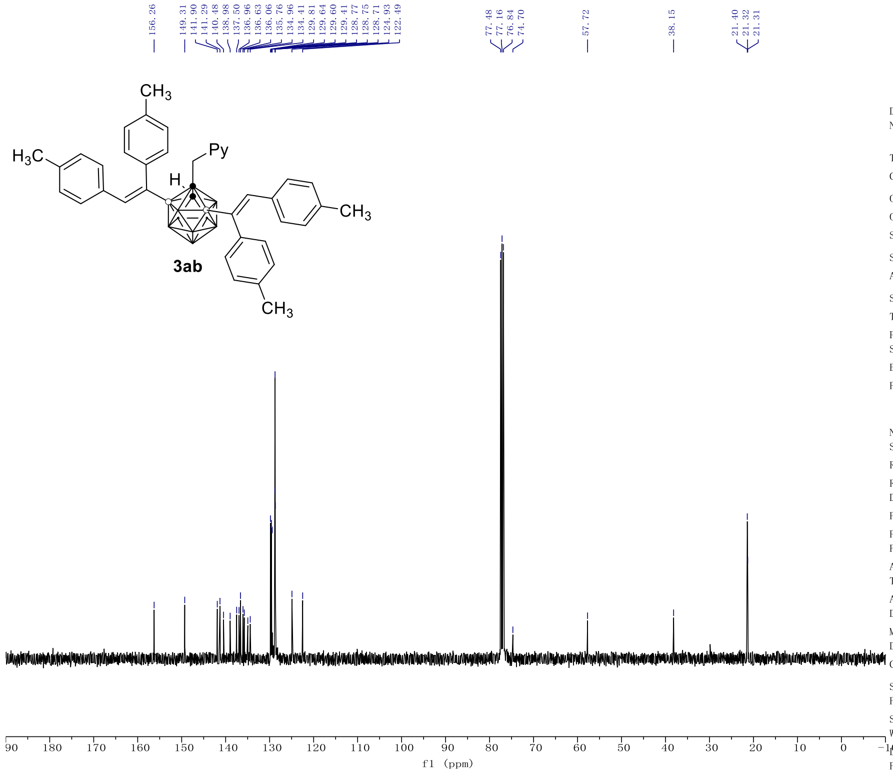

Parameter	Value
Data File Name	E:/ 积分核磁/ 2002-3/ yss-2002-3-c-2/ 38/ fid
Title	yss-2002-3-c-2/ 38
Comment	
Origin	Bruker BioSpin GmbH
Owner	nmr
Site	
Spectrometer	spect
Author	
Solvent	CDCl3
Temperature	296.5
Pulse Sequence	zgpg30
Experiment	1D
Probe	Z116098_0640 (PA BBO 400S1 BBF-H-D-05 Z SP)
Number of Scans	161
Receiver Gain	196
Relaxation Delay	1.0000
Pulse Width	10.0000
Presaturation Frequency	
Acquisition Time	1.2999
Acquisition Date	2023-06-02T22:17:04
Modification Date	2023-06-02T22:17:05
Class	
Spectrometer Frequency	100.64
Spectral Width	25252.5
Lowest Frequency	-1545.9

$^{11}B\{^{1}H\}$ NMR (128 MHz, $CDCl_3$) of **3ab**

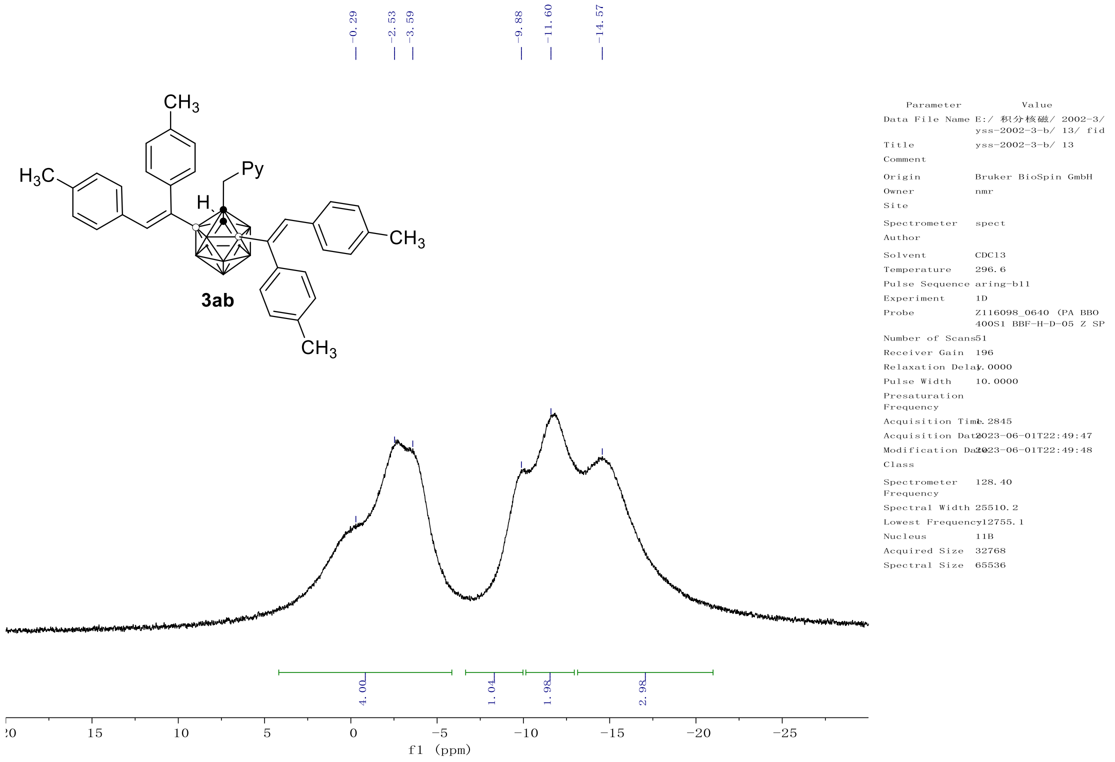

^{11}B NMR (128 MHz, $CDCl_3$) of **3ab**

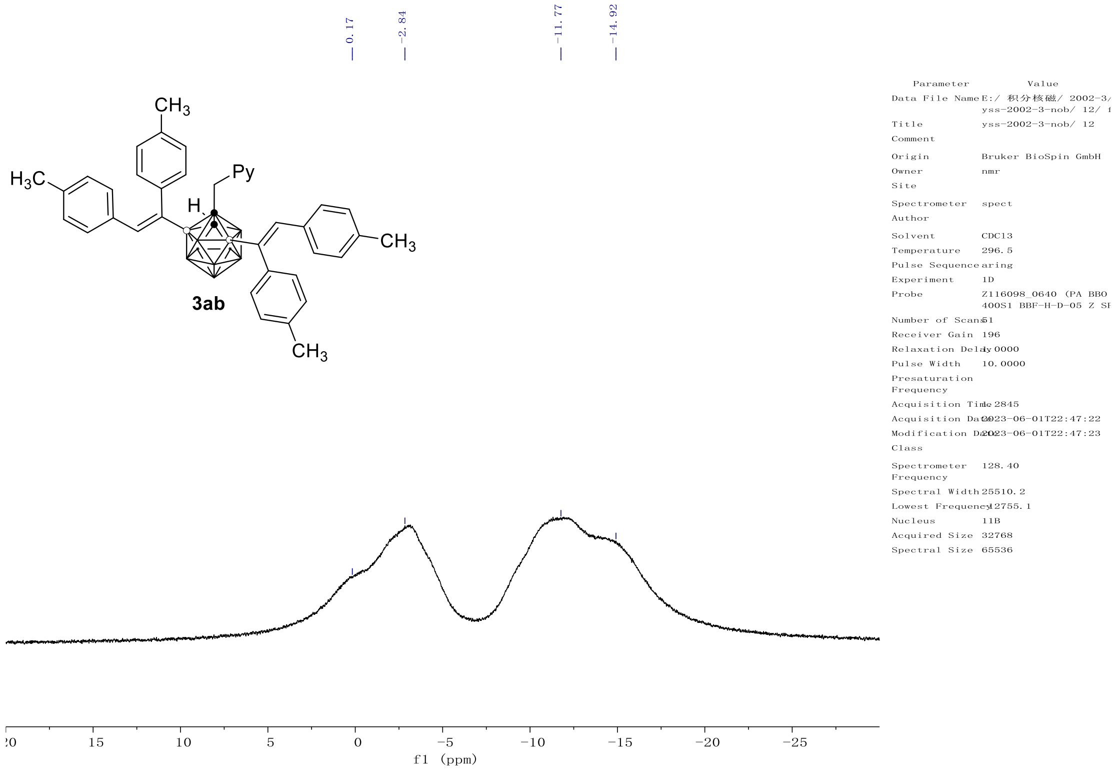

^{1}H NMR (400 MHz, $CDCl_3$) of **3ac**

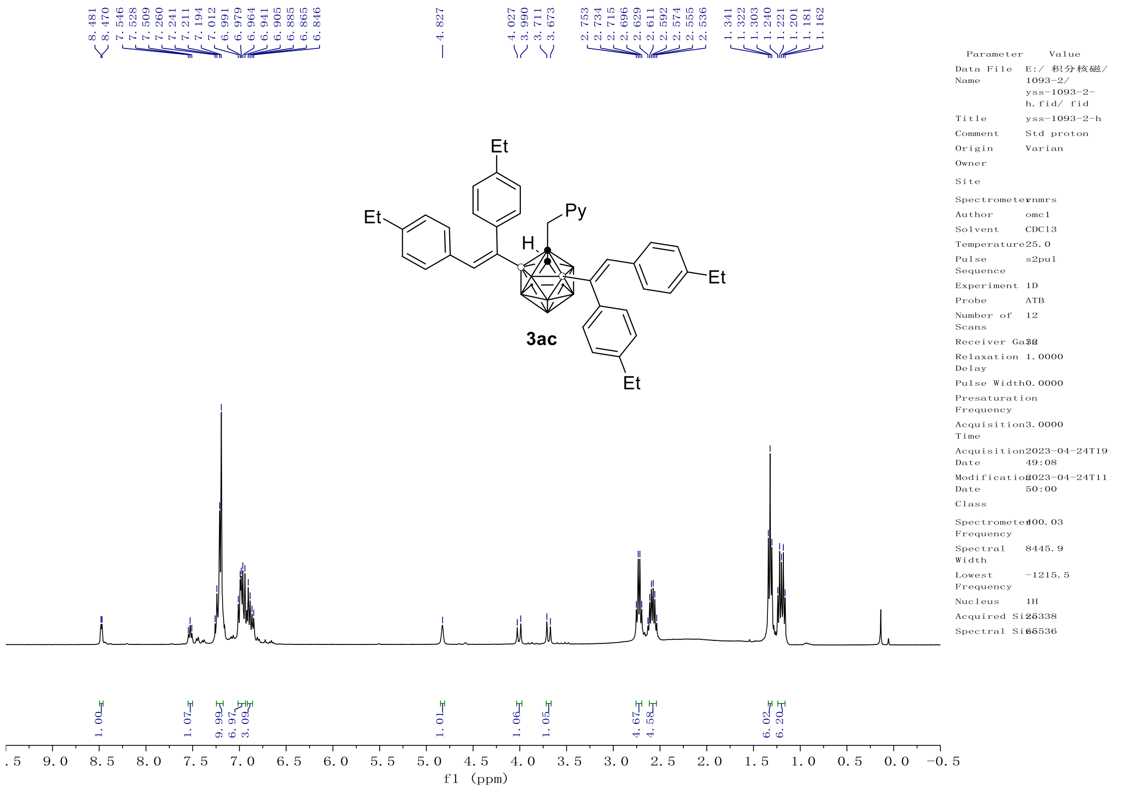

^{13}C{^{1}H} NMR (101 MHz, $CDCl_3$) of **3ac**

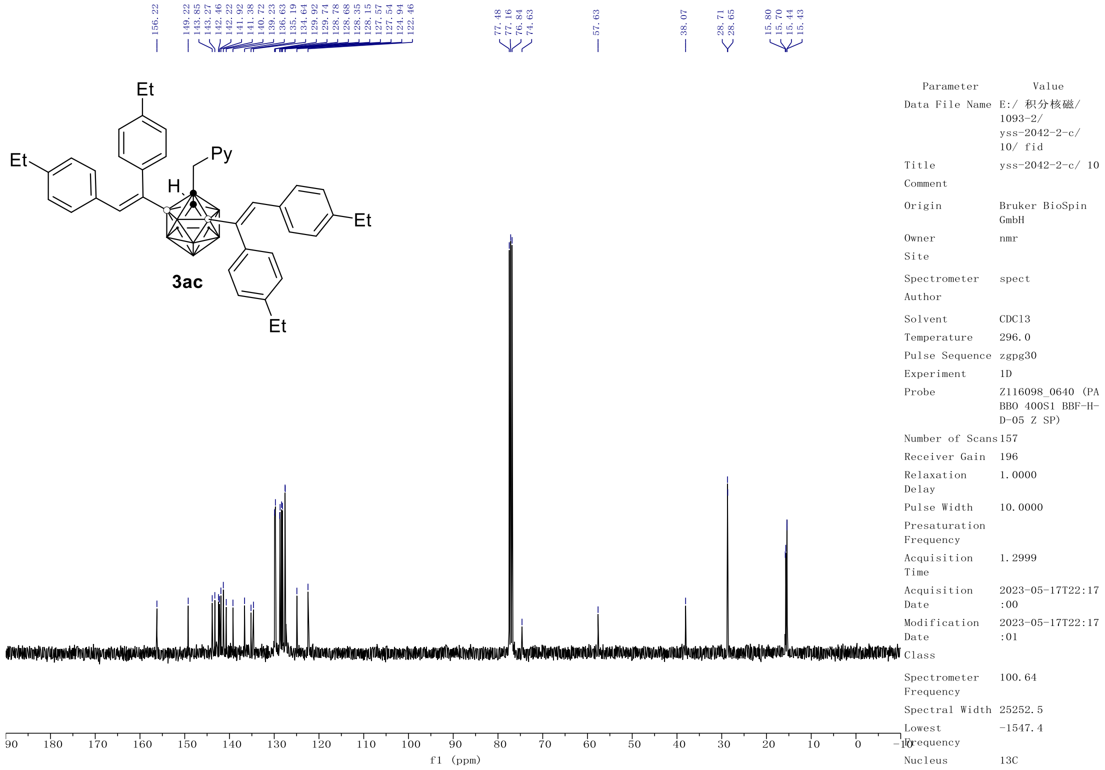

$^{11}B\{^{1}H\}$ NMR (128 MHz, $CDCl_3$) of **3ac**

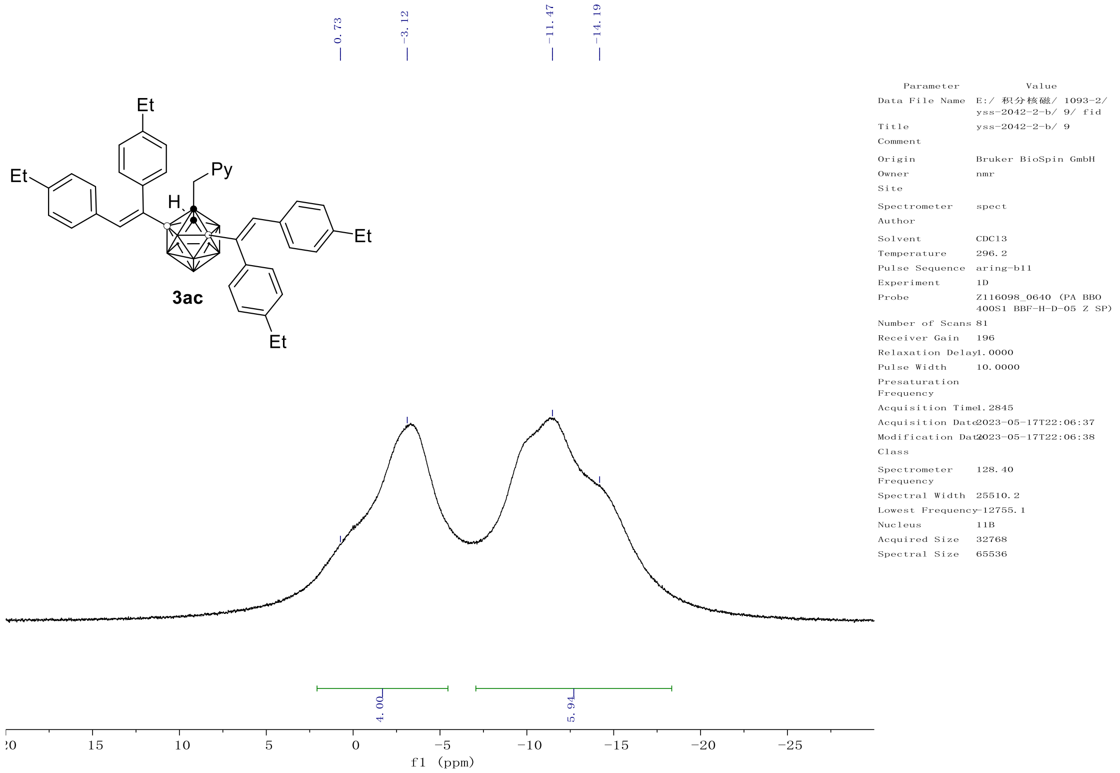

^{11}B NMR (128 MHz, $CDCl_3$) of **3ac**

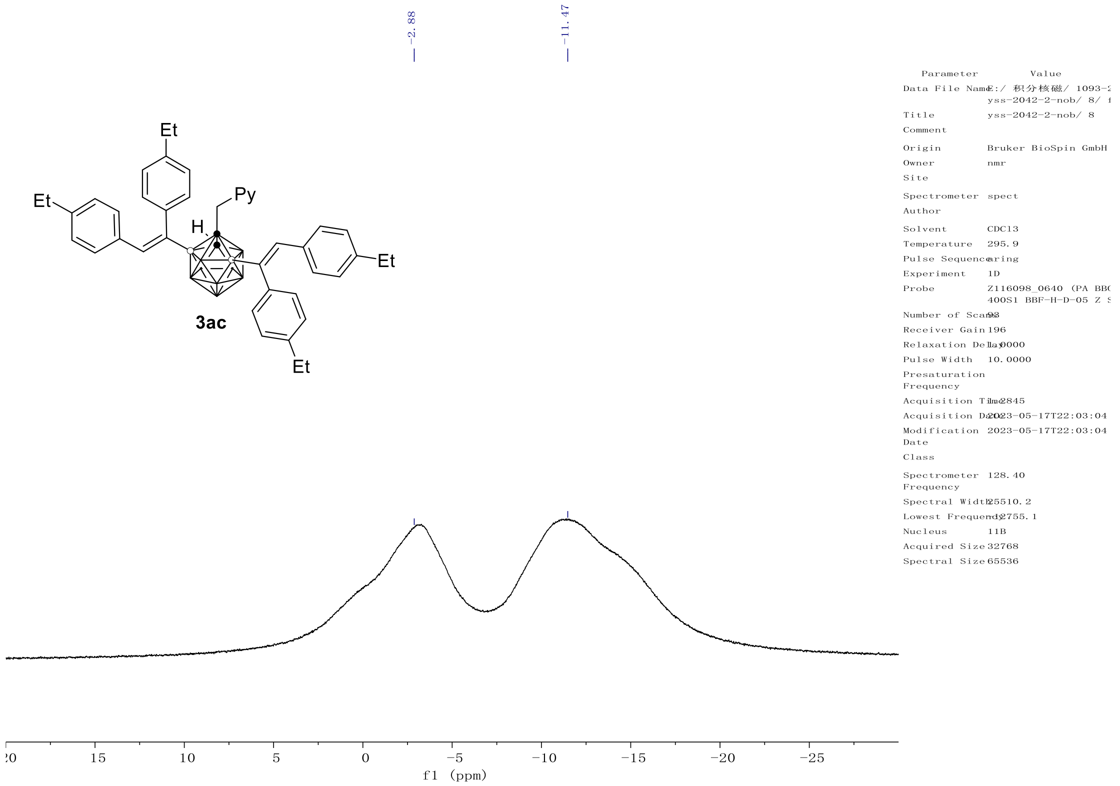

^{1}H NMR (400 MHz, $CDCl_3$) of **3ad**

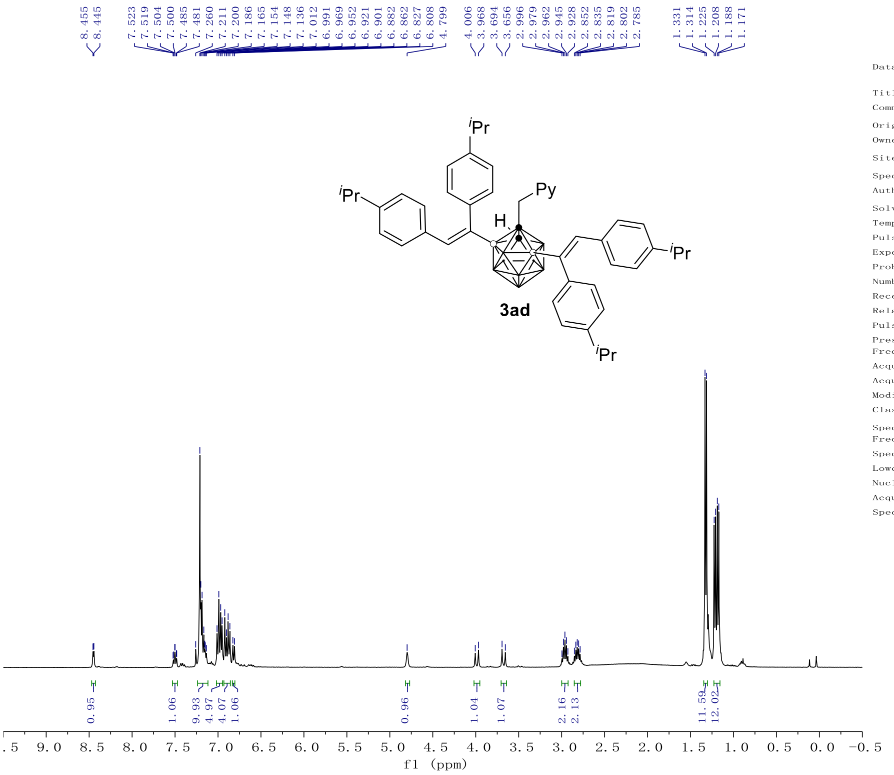

Parameter	Value
Data File Name	E:/ 积分核磁/ 1091-1/ yss-3007-h.fid/ fid
Title	yss-3007-h
Comment	
Origin	Varian
Owner	
Site	
Spectrometer	vnmrs
Author	
Solvent	CDCl3
Temperature	25.0
Pulse Sequence	s2pul
Experiment	1D
Probe	4nuc
Number of Scans	4
Receiver Gain	30
Relaxation Delay	1.0000
Pulse Width	0.0000
Presaturation Frequency	
Acquisition Time	3.0000
Acquisition Date	2023-09-21T18:32:09
Modification Date	2023-09-21T10:32:00
Class	
Spectrometer Frequency	399.72
Spectral Width	8389.3
Lowest Frequency	-1191.9
Nucleus	1H
Acquired Size	25168
Spectral Size	65536

$^{13}C\{^{1}H\}$ NMR (101 MHz, $CDCl_3$) of **3ad**

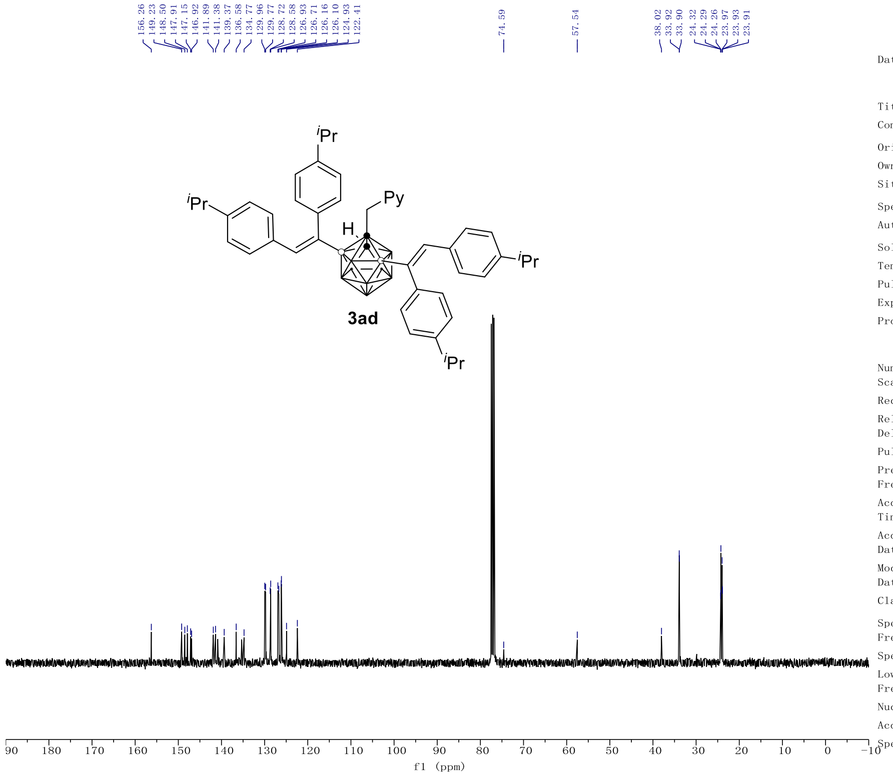

Parameter	Value
Data File Name	H:/ 积分核磁/ 1091-1/ yss-3007-c/ 3/ fid
Title	yss-3007-c/ 3
Comment	
Origin	Bruker BioSpin GmbH
Owner	nmr
Site	
Spectrometer	spect
Author	
Solvent	CDCl3
Temperature	295.8
Pulse Sequence	zgpg30
Experiment	1D
Probe	Z116098_0640 (PA BBO 400S1 BBF-H-D-05 Z SP)
Number of Scans	275
Receiver Gain	196
Relaxation Delay	1.0000
Pulse Width	10.0000
Presaturation Frequency	
Acquisition Time	1.2999
Acquisition Date	2023-10-07T12:28:42
Modification Date	2023-10-07T12:28:44
Class	
Spectrometer Frequency	100.64
Spectral Width	25252.5
Lowest Frequency	-1544.9
Nucleus	13C
Acquired Size	32827
Spectral Size	131072

$^{11}B\{^1H\}$ NMR (128 MHz, $CDCl_3$) of **3ad**

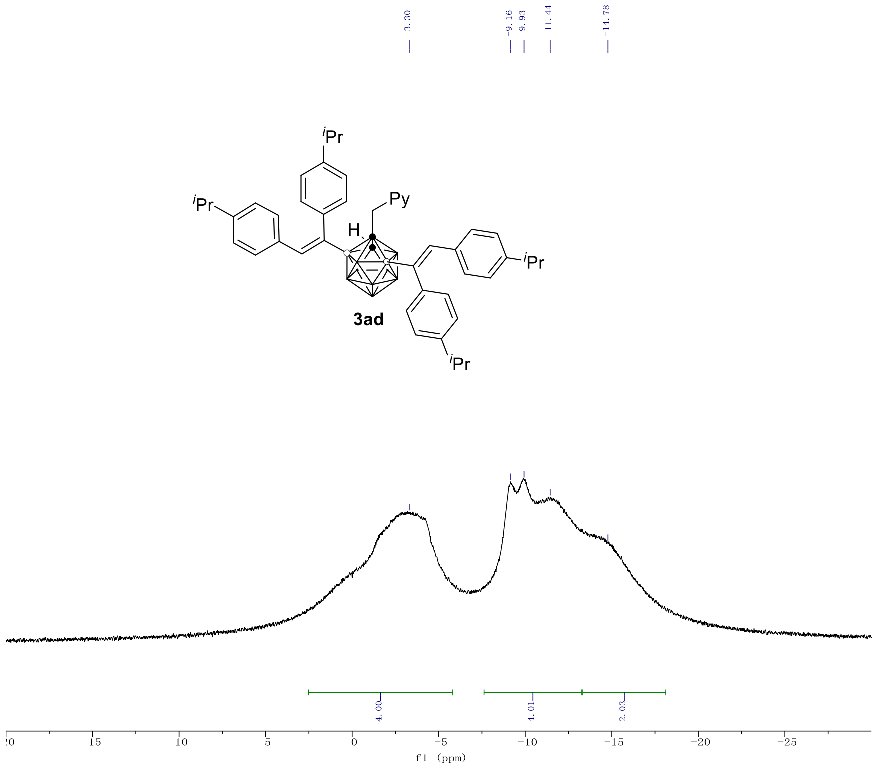

Parameter	Value
Data File Name	E:/ 积分核磁/ 1091-1/ yss-1091-1-b/ 10/ fid
Title	yss-1091-1-b/ 10
Comment	
Origin	Bruker BioSpin GmbH
Owner	nmr
Site	
Spectrometer	spect
Author	
Solvent	CDCl3
Temperature	296.6
Pulse Sequence	aring-b11
Experiment	1D
Probe	Z116098_0640 (PA BBO 400S1 BBF-H-D-05 Z SP)
Number of Scans	129
Receiver Gain	196
Relaxation Delay	1.0000
Pulse Width	10.0000
Presaturation Frequency	
Acquisition Time	1.2845
Acquisition Date	2023-06-01T22:39:25
Modification Date	2023-06-01T22:39:25
Class	
Spectrometer Frequency	128.40
Spectral Width	25510.2
Lowest Frequency	-12755.1
Nucleus	11B
Acquired Size	32768
Spectral Size	65536

^{11}B NMR (128 MHz, $CDCl_3$) of **3ad**

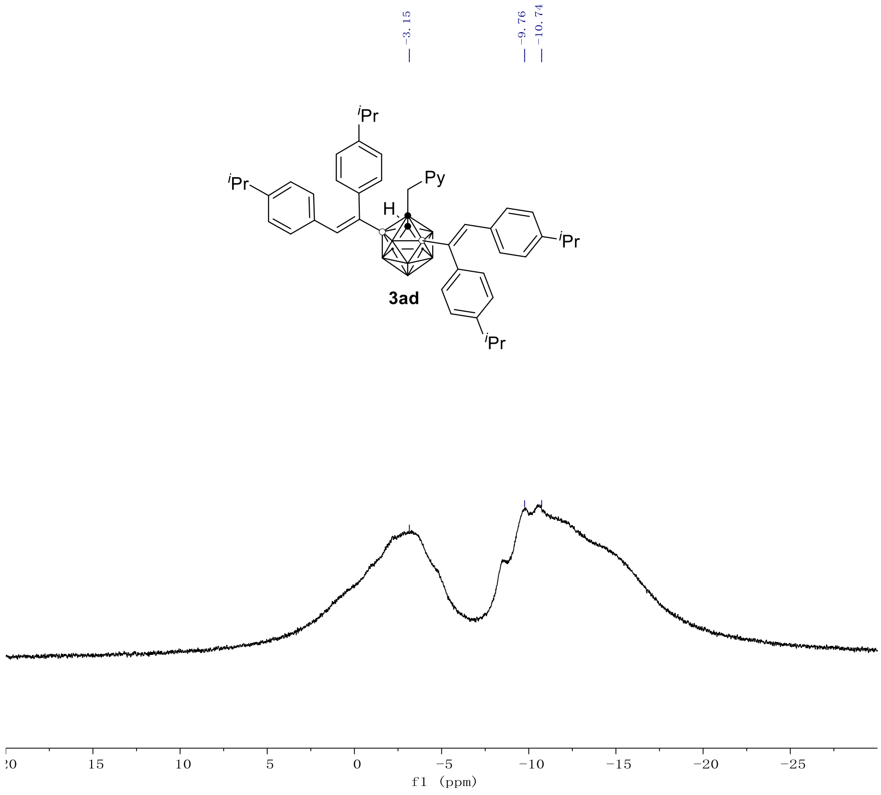

Parameter	Value
Data File Name	E:/ 积分核磁/ 1091-1/ yss-1091-1-nob/ 11/ f
Title	yss-1091-1-nob/ 11
Comment	
Origin	Bruker BioSpin GmbH
Owner	nmr
Site	
Spectrometer	spect
Author	
Solvent	CDCl3
Temperature	296.6
Pulse Sequence	aring
Experiment	1D
Probe	Z116098_0640 (PA BBO 400S1 BBF-H-D-05 Z SP
Number of Scans	128
Receiver Gain	196
Relaxation Delay	1.0000
Pulse Width	10.0000
Presaturation Frequency	
Acquisition Time	1.2845
Acquisition Date	2023-06-01T22:44:41
Modification Date	2023-06-01T22:44:43
Class	
Spectrometer Frequency	128.40
Spectral Width	25510.2
Lowest Frequency	-12755.1
Nucleus	11B
Acquired Size	32768
Spectral Size	65536

^{1}H NMR (400 MHz, CDCl$_3$) of **3ae**

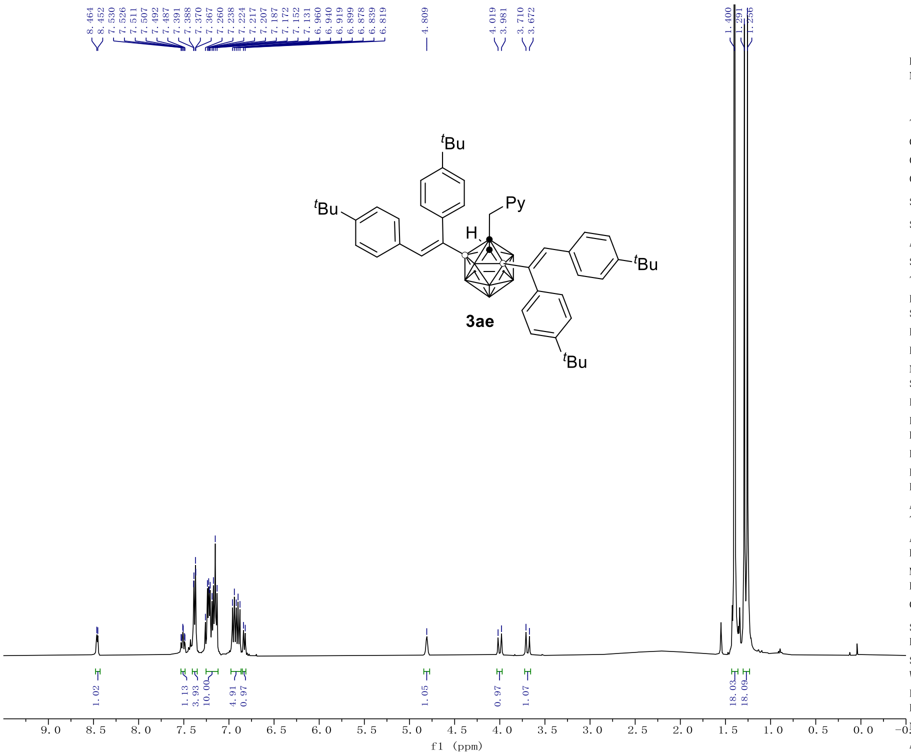

Parameter	Value
Data File Name	E:/ 积分核磁/ 2004-1/ 6-yss-2004-1-h.fid/ fid
Title	6-yss-2004-1-h
Comment	Std proton
Origin	Varian
Owner	
Site	
Spectrometer	vnmrs
Author	omc1
Solvent	CDCl3
Temperature	25.0
Pulse Sequence	s2pul
Experiment	1D
Probe	ATB
Number of Scans	4
Receiver Gain	34
Relaxation Delay	1.0000
Pulse Width	0.0000
Presaturation Frequency	
Acquisition Time	3.0000
Acquisition Date	2023-05-18T21:39:19
Modification Date	2023-05-18T13:40:00
Class	
Spectrometer Frequency	400.03
Spectral Width	10080.6
Lowest Frequency	-2032.9
Nucleus	1H
Acquired Size	30242

^{13}C{^{1}H} NMR (101 MHz, CDCl$_3$) of **3ae**

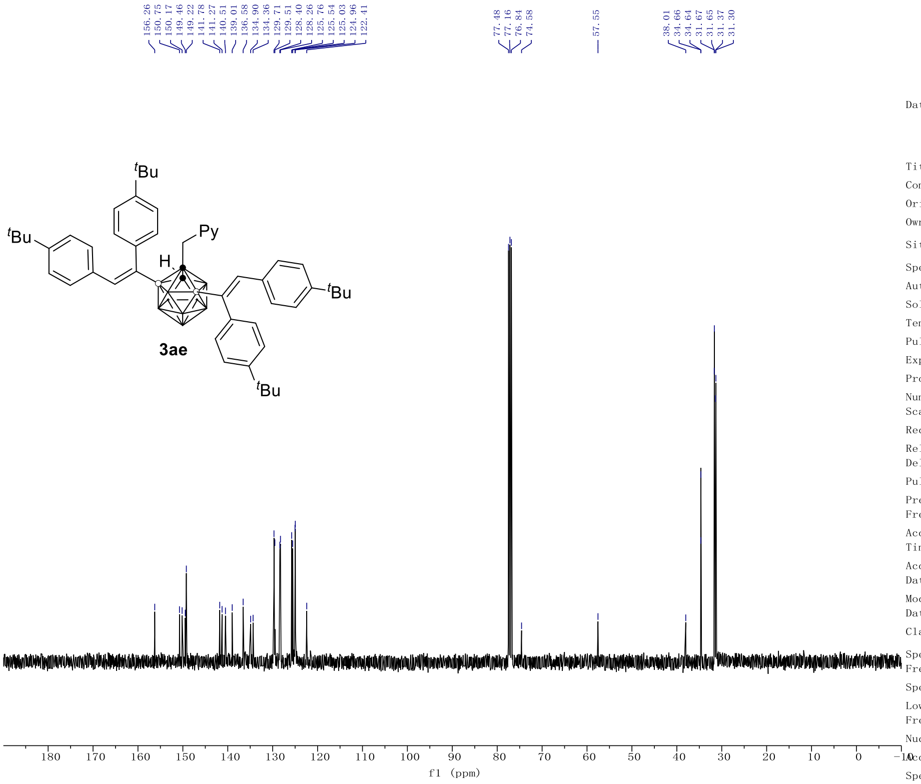

Parameter	Value
Data File Name	E:/ 积分核磁/ 2004-1/ yss-2004-1-c.fid/ fid
Title	yss-2004-1-c
Comment	Std carbon
Origin	Varian
Owner	
Site	
Spectrometer	vnmrs
Author	omc1
Solvent	cdcl3
Temperature	25.0
Pulse Sequence	s2pul
Experiment	1D
Probe	ATB
Number of Scans	168
Receiver Gain	60
Relaxation Delay	1.0000
Pulse Width	0.0000
Presaturation Frequency	
Acquisition Time	1.3000
Acquisition Date	2023-05-18T21:40:30
Modification Date	2023-05-18T13:47:00
Class	
Spectrometer Frequency	100.60
Spectral Width	28409.1
Lowest Frequency	-2120.5
Nucleus	13C
Acquired Size	36932
Spectral Size	131072

$^{11}B\{^{1}H\}$ NMR (128 MHz, $CDCl_3$) of **3ae**

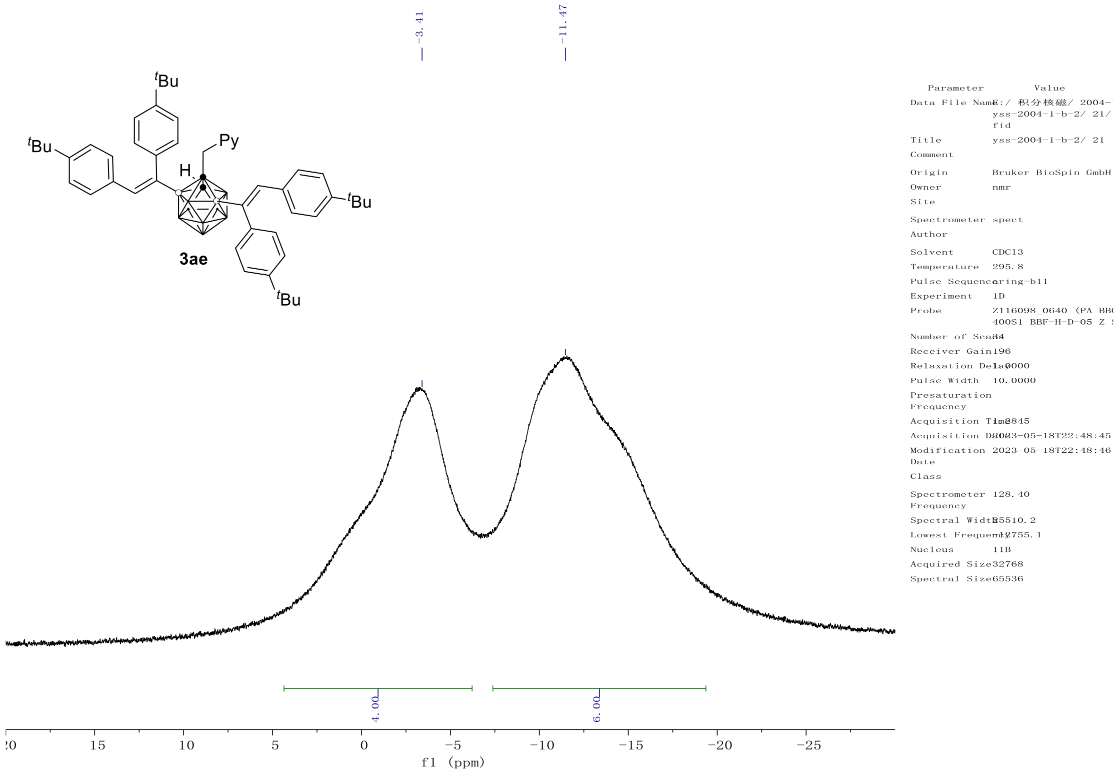

^{11}B NMR (128 MHz, $CDCl_3$) of **3ae**

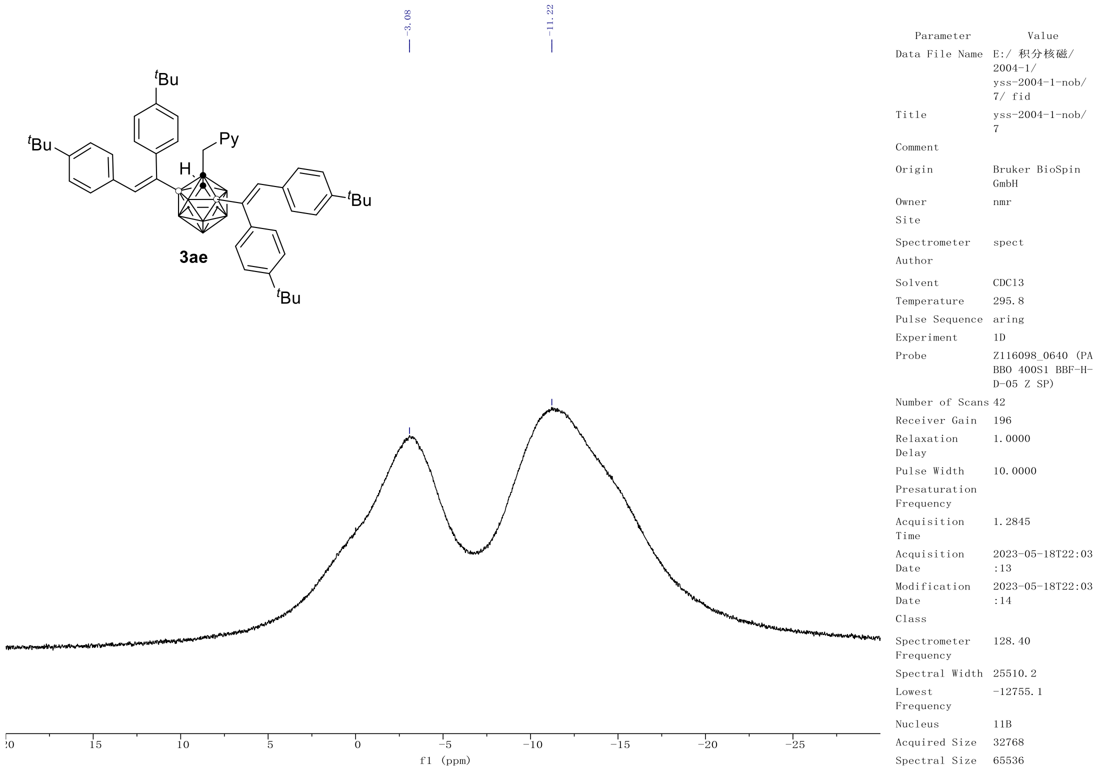

^{1}H NMR (400 MHz, $CDCl_3$) of **3af**

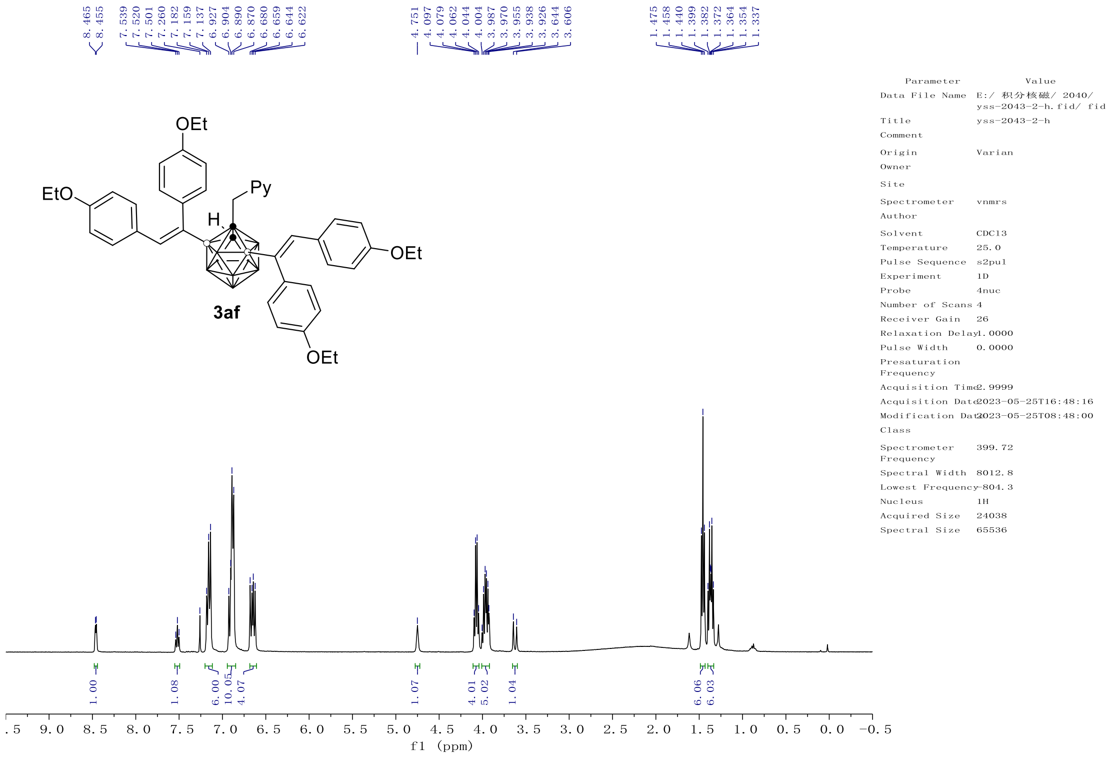

$^{13}C\{^{1}H\}$ NMR (101 MHz, $CDCl_3$) of **3af**

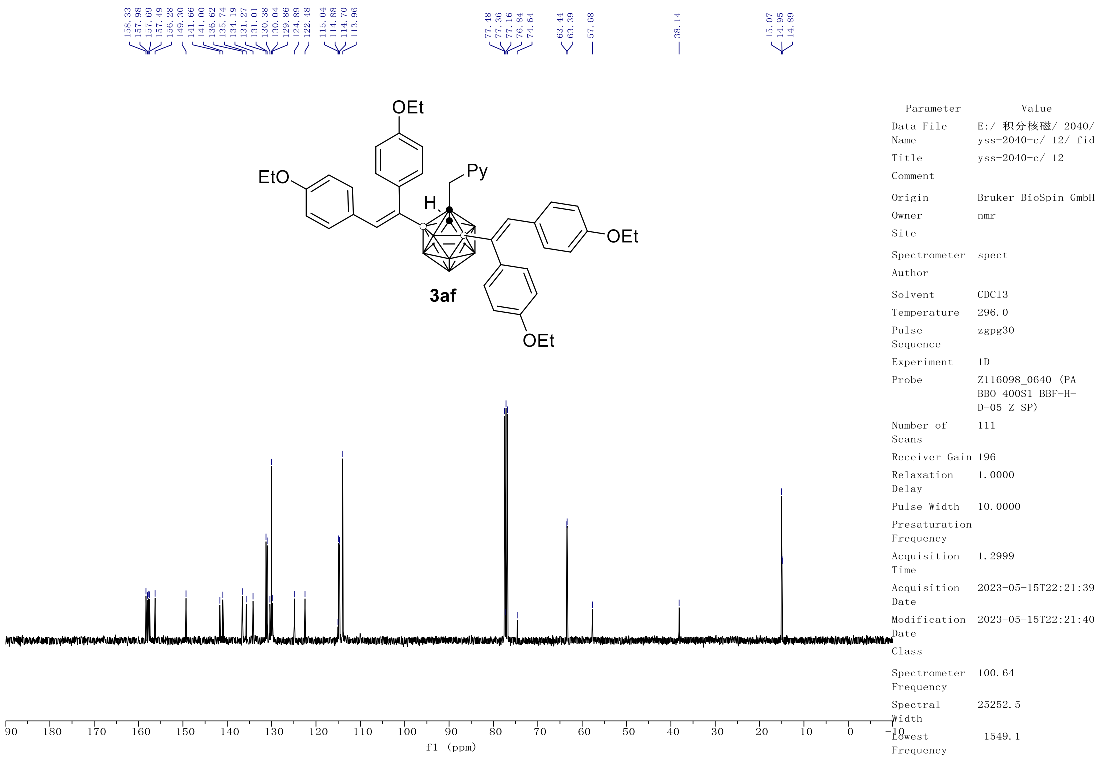

$^{11}B\{^{1}H\}$ NMR (128 MHz, $CDCl_3$) of **3af**

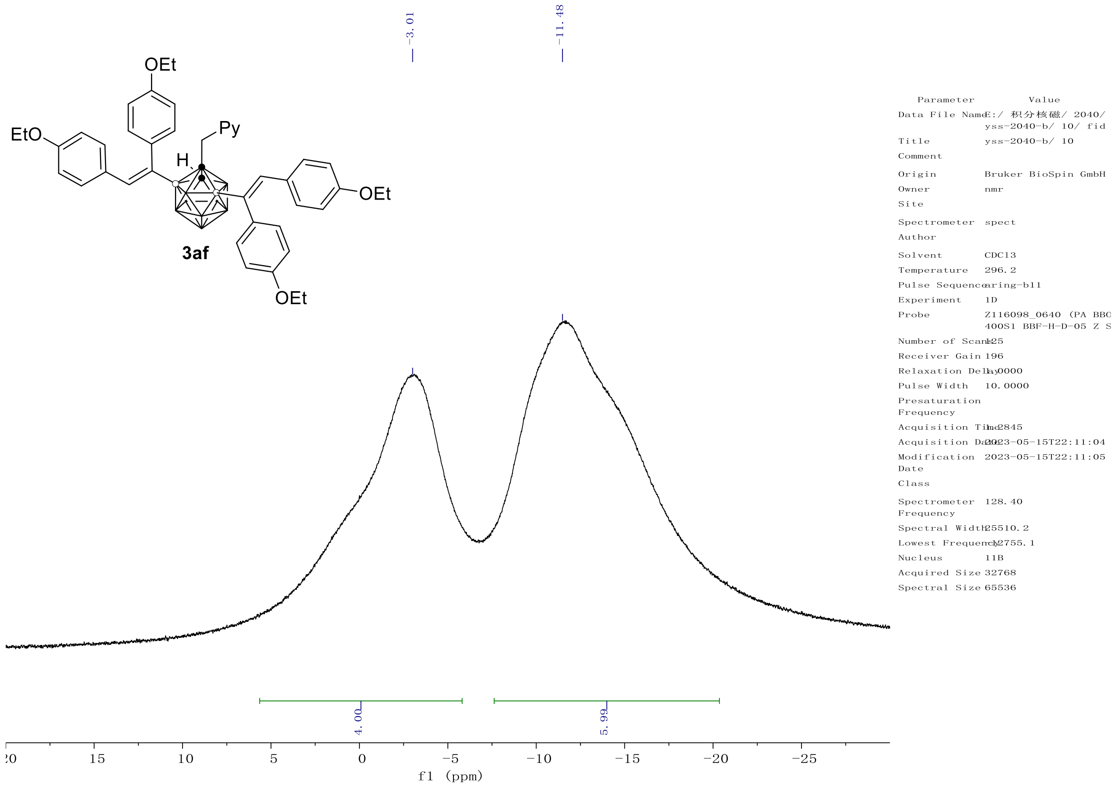

^{11}B NMR (128 MHz, $CDCl_3$) of **3af**

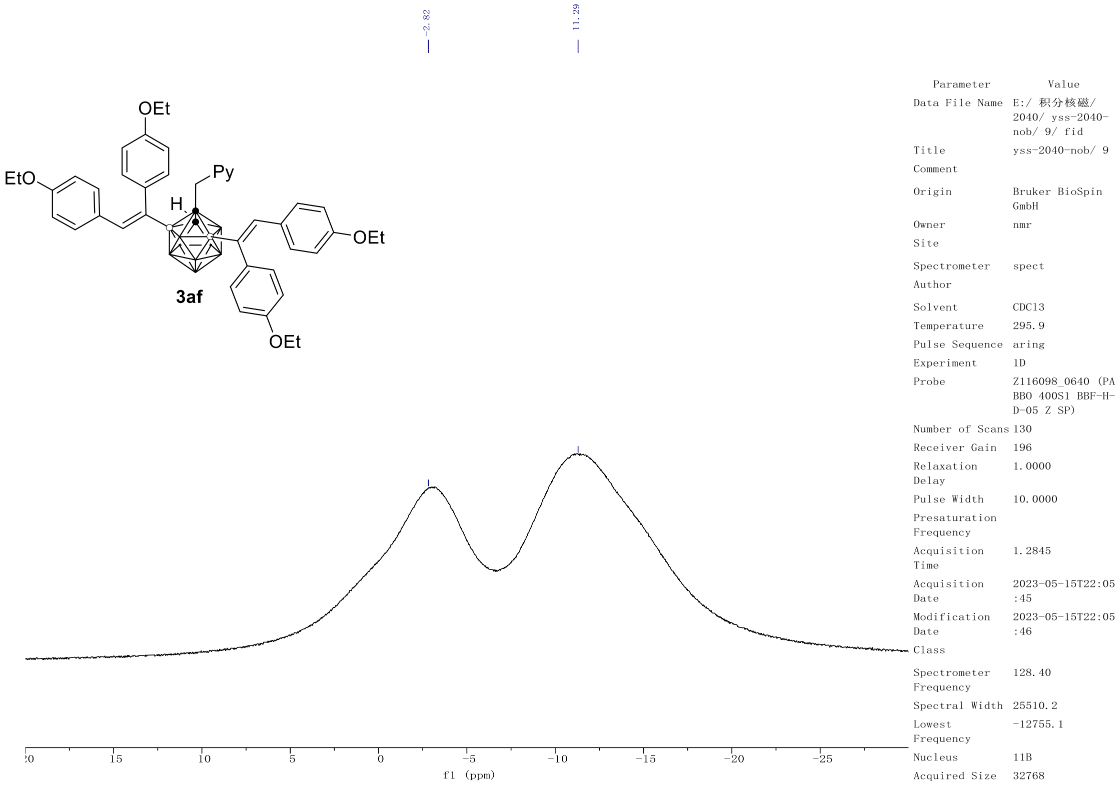

^{1}H NMR (400 MHz, $CDCl_3$) of **3ag**

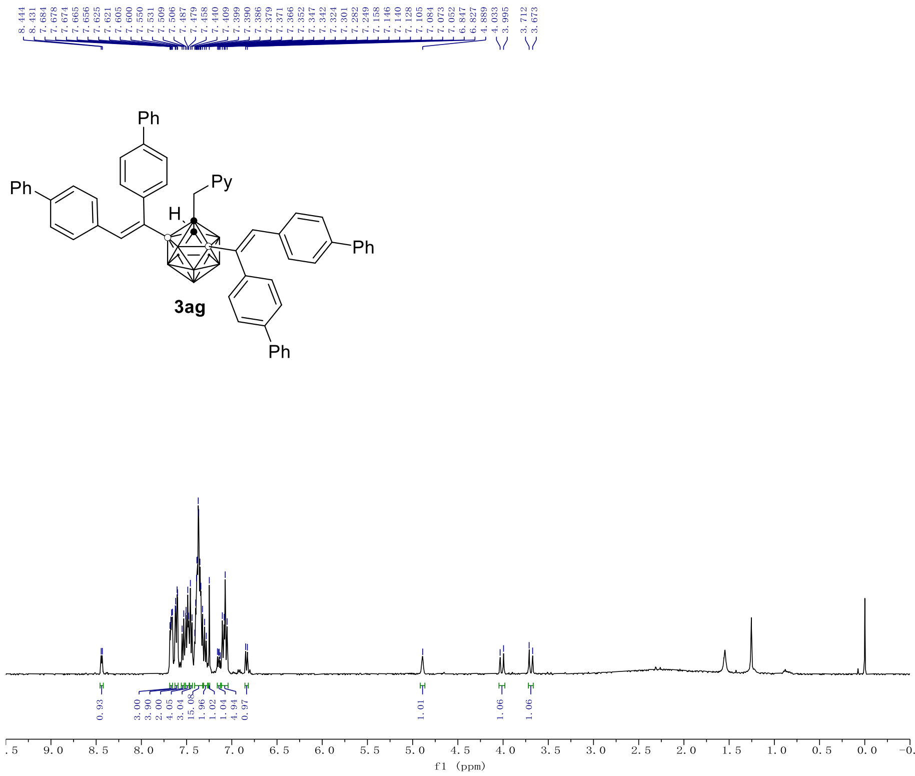

Parameter	Value
Data File Name	E:/ 积分核磁/ 3001/ yss-3001- h.fid/ fid
Title	yss-3001-h
Comment	
Origin	Varian
Owner	
Site	
Spectrometer	vnmrs
Author	
Solvent	CDCl3
Temperature	25.0
Pulse Sequence	s2pul
Experiment	1D
Probe	4nuc
Number of Scans	4
Receiver Gain	46
Relaxation Delay	1.0000
Pulse Width	0.0000
Presaturation Frequency	
Acquisition Time	3.0000
Acquisition Date	2023-09-11T18:31:03
Modification Date	2023-09-11T10:31:00
Class	
Spectrometer Frequency	399.72
Spectral Width	8389.3
Lowest Frequency	-1197.2
Nucleus	1H
Acquired Size	25168
Spectral Size	65536

^{13}C{^{1}H} NMR (101 MHz, $CDCl_3$) of **3ag**

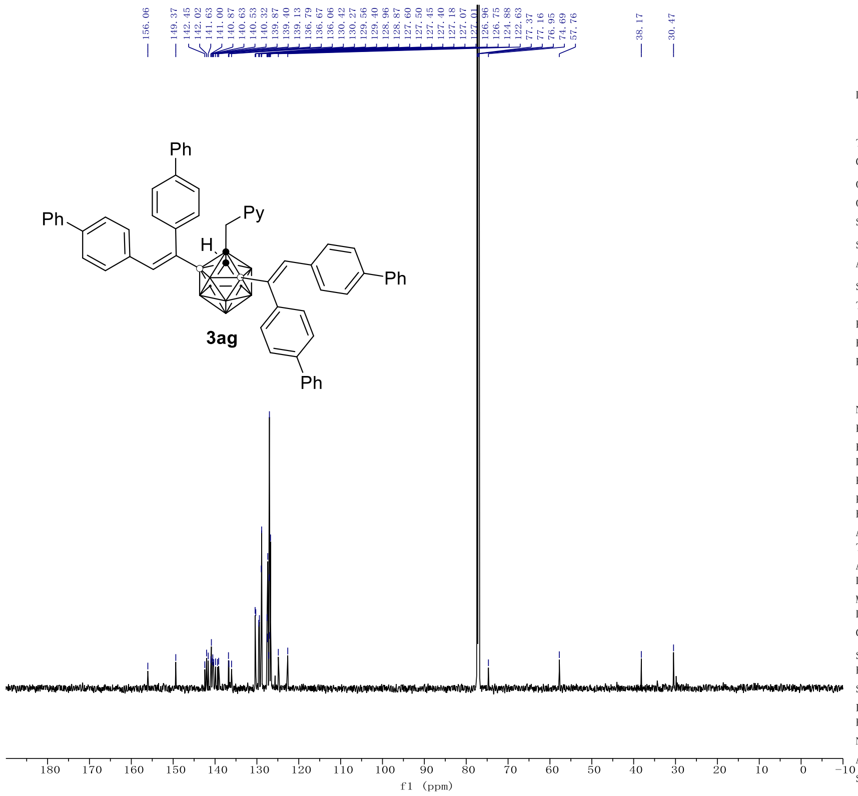

Parameter	Value
Data File Name	E:/ 积分核磁/ 3001/ YSS-3001/ 1/ pdata/ 1/ 1r
Title	pdata/ 1
Comment	
Origin	Bruker BioSpin GmbH
Owner	nmr
Site	
Spectrometer	Avance NEO
Author	
Solvent	Acetone
Temperature	298.2
Pulse Sequence	zgpg30
Experiment	1D
Probe	Z114607_0307 (PA BBO 600S3 BBF-H-D-05 Z SP)
Number of Scans	454
Receiver Gain	101
Relaxation Delay	2.0000
Pulse Width	12.0000
Presaturation Frequency	
Acquisition Time	0.7209
Acquisition Date	2023-09-12T10:56:26
Modification Date	2023-09-12T10:55:22
Class	
Spectrometer Frequency	150.90
Spectral Width	45454.5
Lowest Frequency	-6834.5
Nucleus	13C
Acquired Size	32768
Spectral Size	32768

$^{11}B\{^{1}H\}$ NMR (128 MHz, $CDCl_3$) of **3ag**

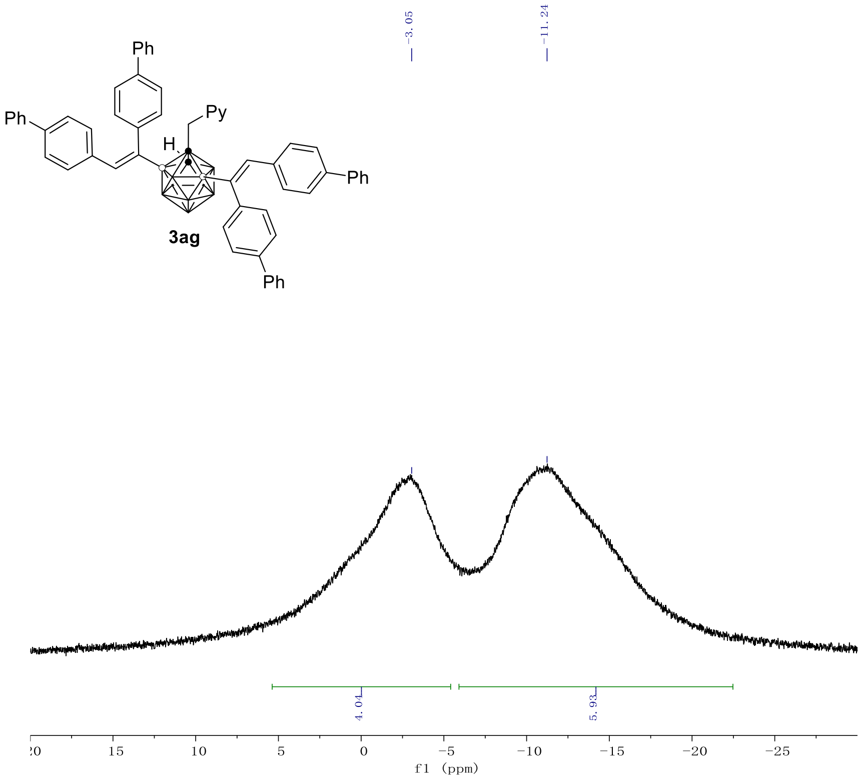

Parameter	Value
Data File Name	E:/ 积分核磁/ 3001/ yss-3001-b/ 7/ fid
Title	yss-3001-b/ 7
Comment	
Origin	Bruker BioSpin GmbH
Owner	nmr
Site	
Spectrometer	spect
Author	
Solvent	CDCl3
Temperature	296.0
Pulse Sequence	aring-b11
Experiment	1D
Probe	Z116098_0640 (PA BBO 400S BBF-H-D-05 Z SP)
Number of Scans	99
Receiver Gain	196
Relaxation Delay	1.0000
Pulse Width	10.0000
Presaturation Frequency	
Acquisition Time	1.2845
Acquisition Date	2023-09-21T18:15:52
Modification Date	2023-09-21T18:15:53
Class	
Spectrometer Frequency	128.40
Spectral Width	25510.2
Lowest Frequency	-12755.1
Nucleus	11B
Acquired Size	32768
Spectral Size	65536

^{11}B NMR (128 MHz, $CDCl_3$) of **3ag**

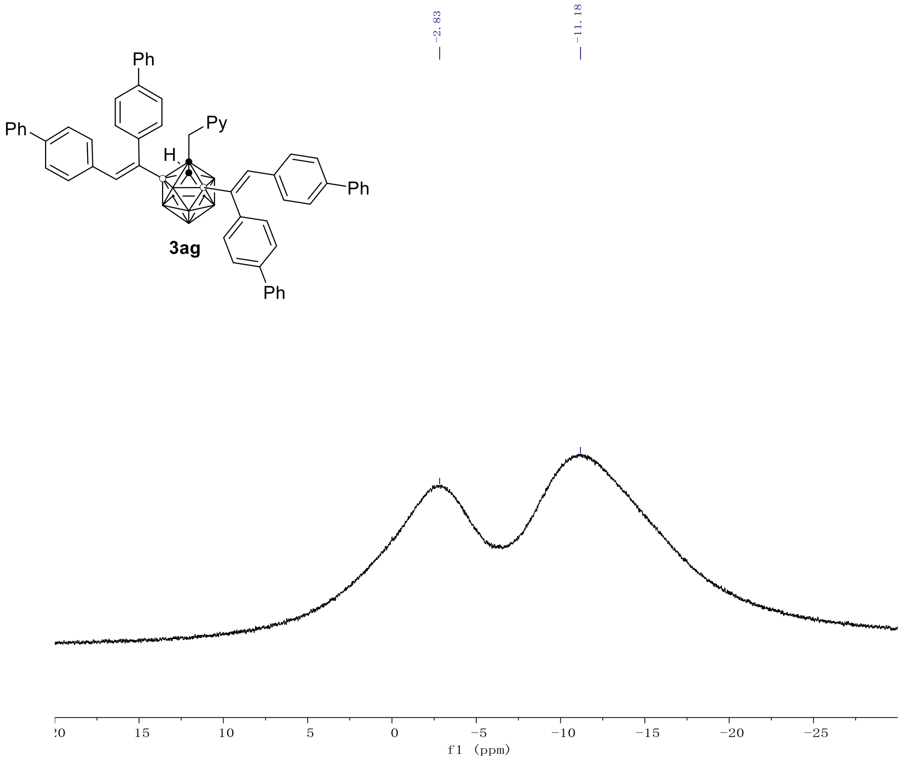

Parameter	Value
Data File Name	E:/ 积分核磁/ 3001/ yss-2051-2-nob/ 7/ fid
Title	yss-2051-2-nob/ 7
Comment	
Origin	Bruker BioSpin GmbH
Owner	nmr
Site	
Spectrometer	spect
Author	
Solvent	C6D6
Temperature	296.2
Pulse Sequence	aring
Experiment	1D
Probe	Z116098_0640 (PA BBO 400S BBF-H-D-05 Z SP)
Number of Scans	36
Receiver Gain	196
Relaxation Delay	1.0000
Pulse Width	10.0000
Presaturation Frequency	
Acquisition Time	1.2845
Acquisition Date	2023-06-17T16:22:04
Modification Date	2023-06-17T16:22:05
Class	
Spectrometer Frequency	128.40
Spectral Width	25510.2
Lowest Frequency	-12755.1
Nucleus	11B
Acquired Size	32768
Spectral Size	65536

^{1}H NMR (400 MHz, $CDCl_3$) of **3ah**

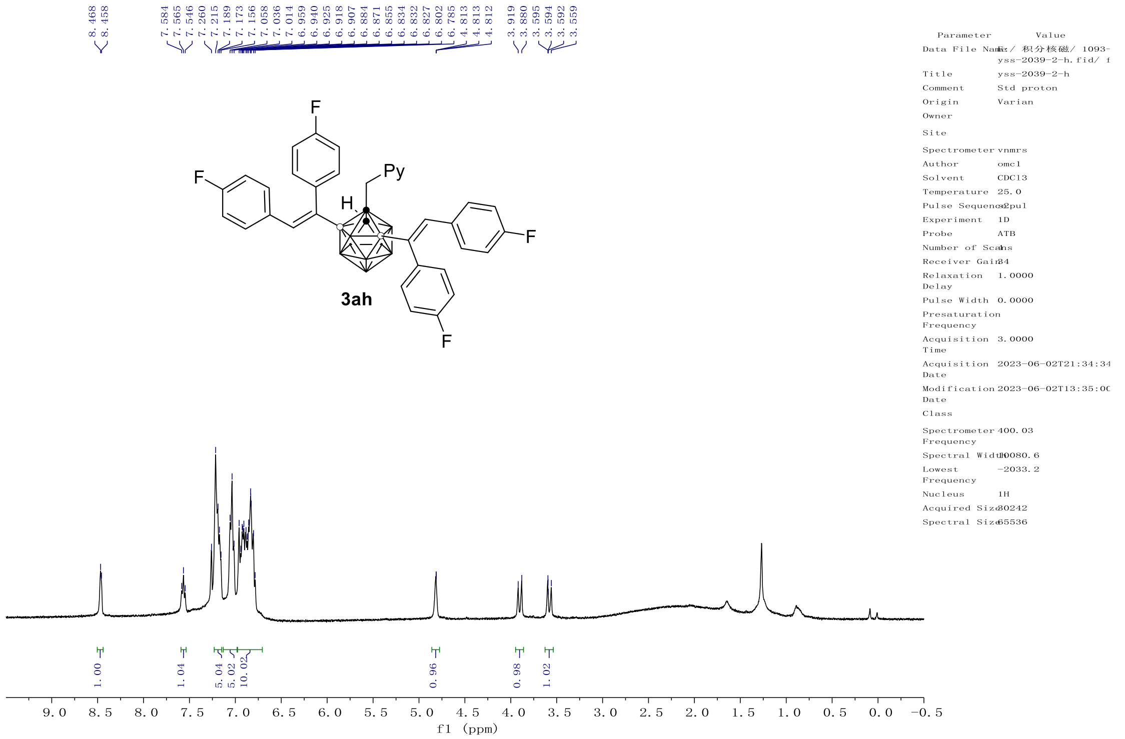

^{13}C{^{1}H} NMR (101 MHz, $CDCl_3$) of **3ah**

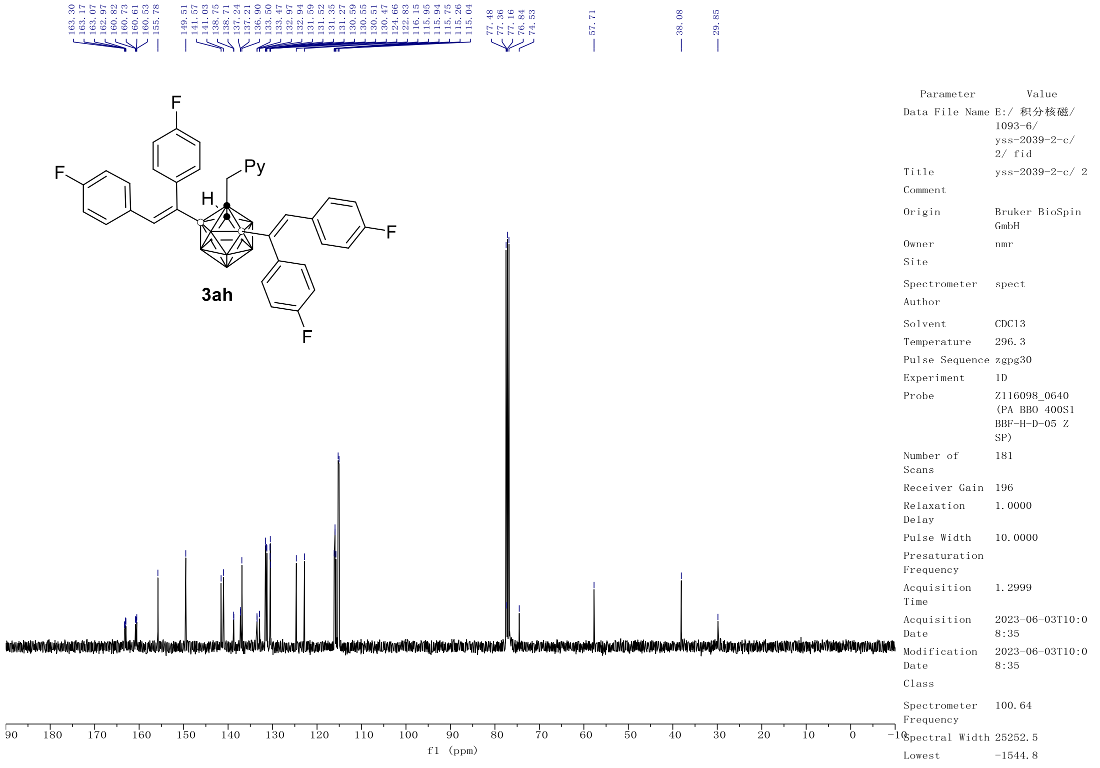

$^{11}B\{^{1}H\}$ NMR (128 MHz, $CDCl_3$) of **3ah**

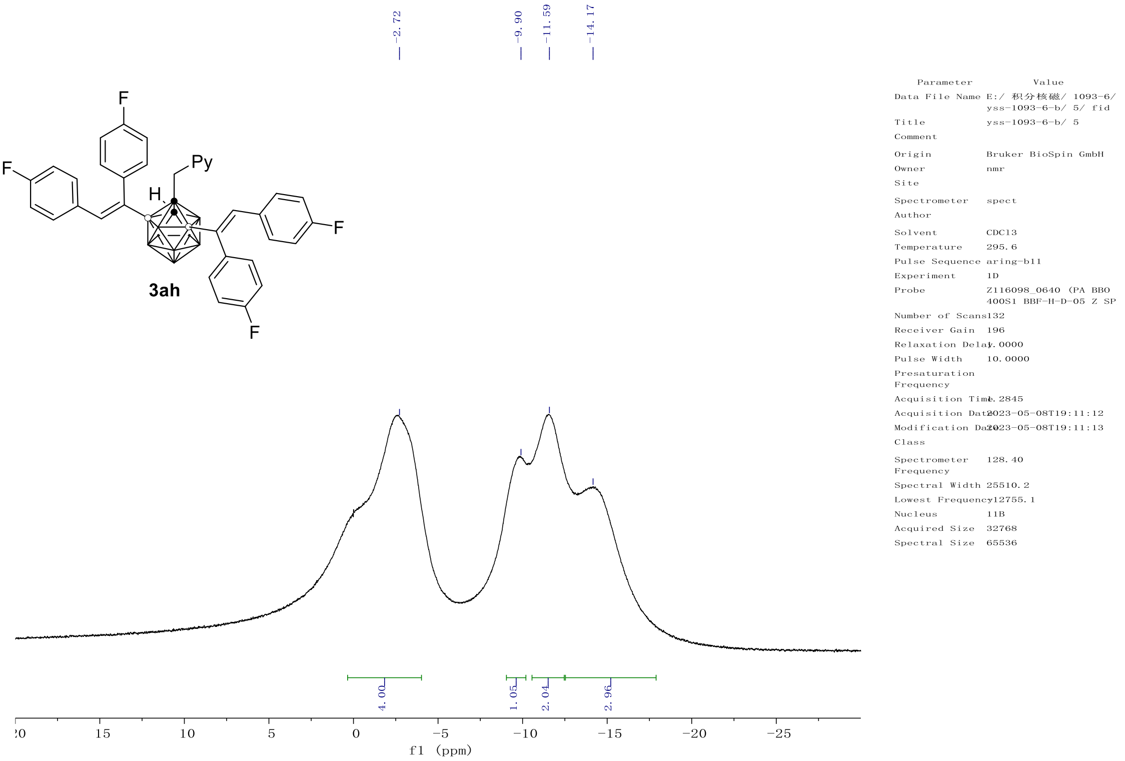

^{11}B NMR (128 MHz, $CDCl_3$) of **3ah**

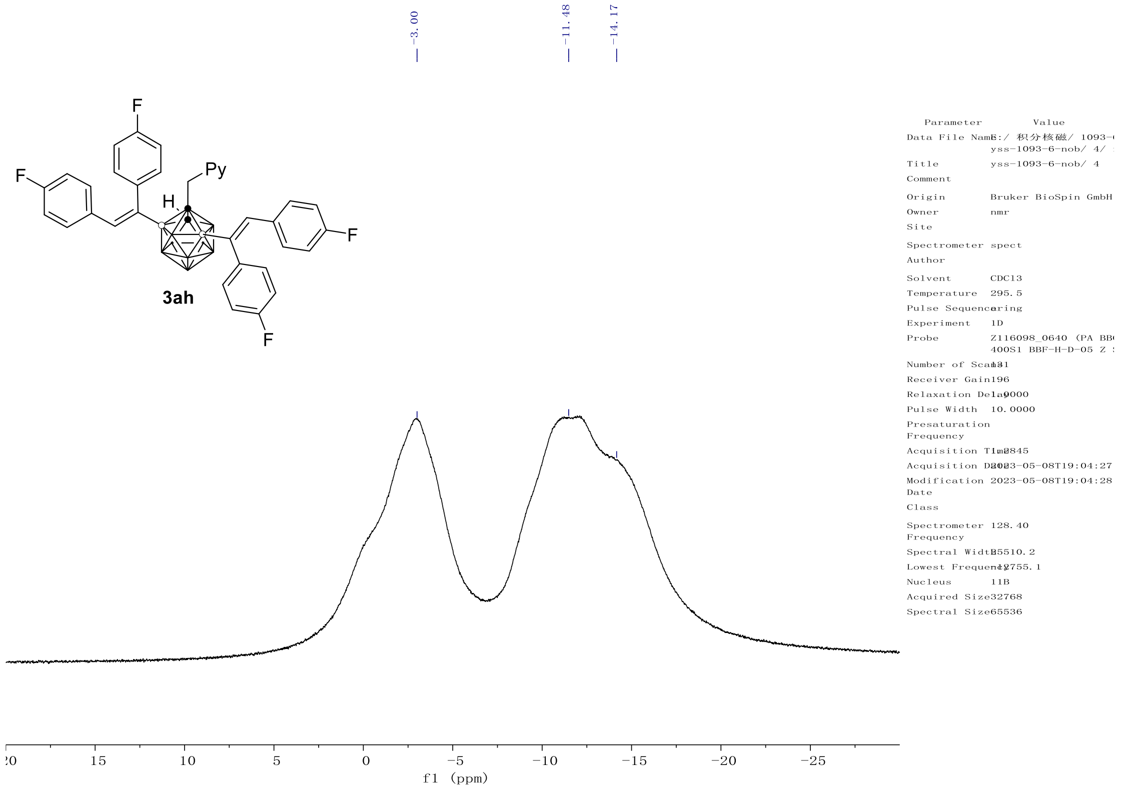

^{19}F NMR (376 MHz, $CDCl_3$) of **3ah**

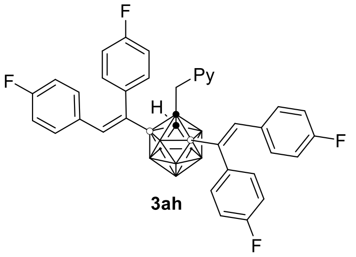

3ah

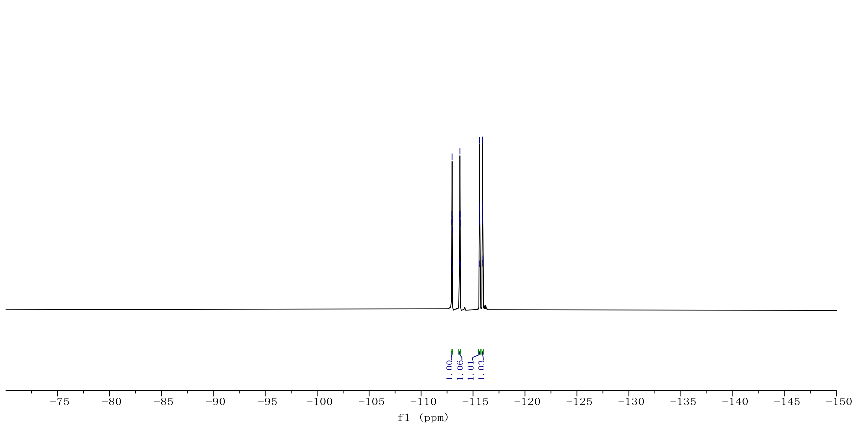

Parameter	Value
Data File Name	E:/ 积分核磁/ 1093-6/ yss-2039-2-f/ 3/ fid
Title	yss-2039-2-f/ 3
Comment	
Origin	Bruker BioSpin GmbH
Owner	nmr
Site	
Spectrometer	spect
Author	
Solvent	CDCl3
Temperature	296.3
Pulse Sequence	zgflqn
Experiment	1D
Probe	Z116098_0640 (PA BBO 400S1 BBF-H-D-05 Z SP)
Number of Scans	19
Receiver Gain	196
Relaxation Delay	1.0000
Pulse Width	18.0000
Presaturation Frequency	
Acquisition Time	0.9999
Acquisition Date	2023-06-03T10:12:41
Modification Date	2023-06-03T10:12:42
Class	
Spectrometer Frequency	376.53
Spectral Width	150000.0
Lowest Frequency	-112656.4
Nucleus	19F
Acquired Size	149992
Spectral Size	524288

^{1}H NMR (400 MHz, $CDCl_3$) of **3ai**

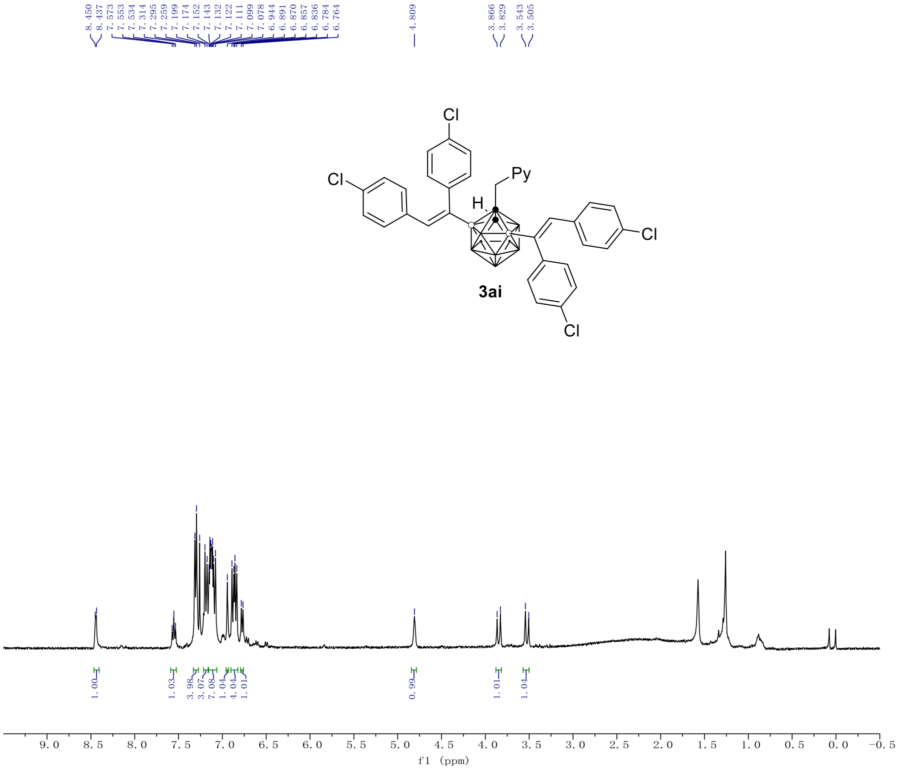

Parameter	Value
Data File Name	E:/ 积分核磁/ 1094-2/ yss-2043-3-h.fid/ fid
Title	yss-2043-3-h
Comment	Std proton
Origin	Varian
Owner	
Site	
Spectrometer	vnmrs
Author	omc1
Solvent	CDCl3
Temperature	25.0
Pulse Sequence	s2pul
Experiment	1D
Probe	ATB
Number of Scans	4
Receiver Gain	38
Relaxation Delay	1.0000
Pulse Width	0.0000
Presaturation Frequency	
Acquisition Time	3.0000
Acquisition Date	2023-06-01T21:40:37
Modification Date	2023-06-01T13:41:00
Class	
Spectrometer Frequency	400.03
Spectral Width	10080.6
Lowest Frequency	-2033.2
Nucleus	1H
Acquired Size	30242

$^{13}C\{^{1}H\}$ NMR (101 MHz, $CDCl_3$) of **3ai**

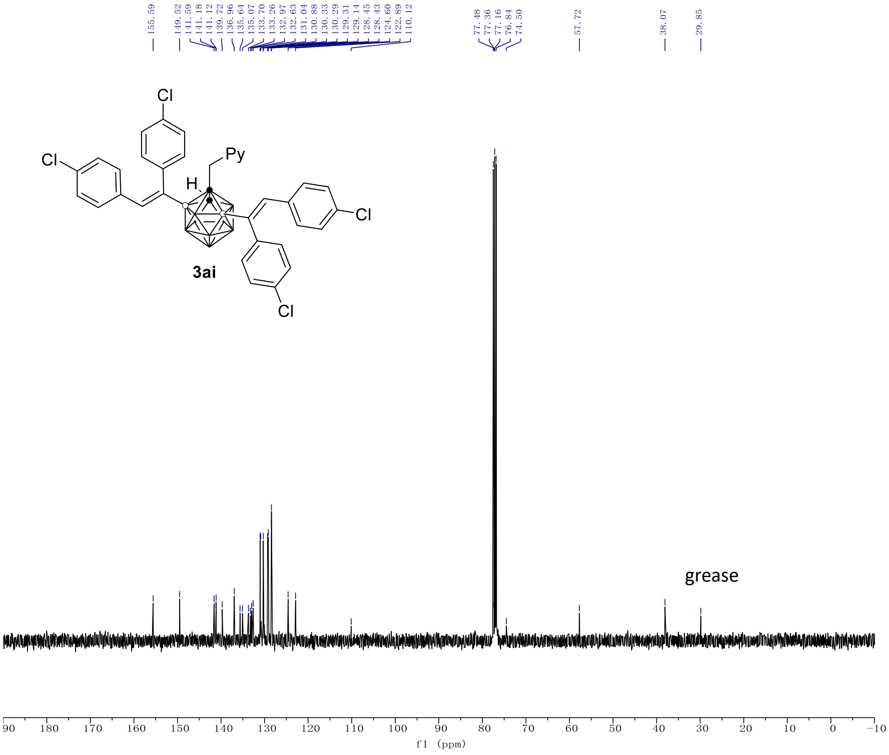

Parameter	Value
Data File Name	E:/ 积分核磁/ 1094-2/ yss-2043-c/ 14/ fid
Title	yss-2043-c/ 14
Comment	
Origin	Bruker BioSpin GmbH
Owner	nmr
Site	
Spectrometer	spect
Author	
Solvent	CDCl3
Temperature	296.5
Pulse Sequence	zgpg30
Experiment	1D
Probe	Z116098_0640 (PA BBO 400S1 BBF-H-D-05 Z SP)
Number of Scans	201
Receiver Gain	196
Relaxation Delay	1.0000
Pulse Width	10.0000
Presaturation Frequency	
Acquisition Time	1.2999
Acquisition Date	2023-06-01T23:00:55
Modification Date	2023-06-01T23:00:56
Class	
Spectrometer Frequency	100.64
Spectral Width	25252.5
Lowest Frequency	-1544.3
Nucleus	13C

$^{11}B\{^{1}H\}$ NMR (128 MHz, $CDCl_3$) of **3ai**

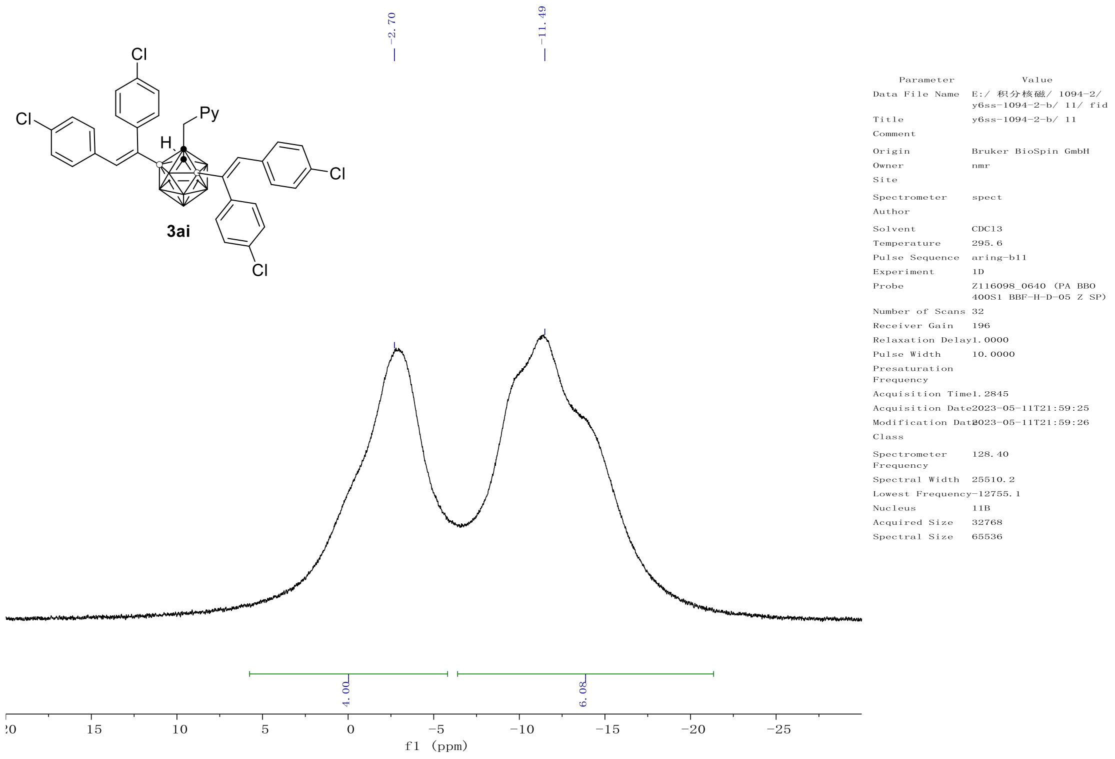

^{11}B NMR (128 MHz, $CDCl_3$) of **3ai**

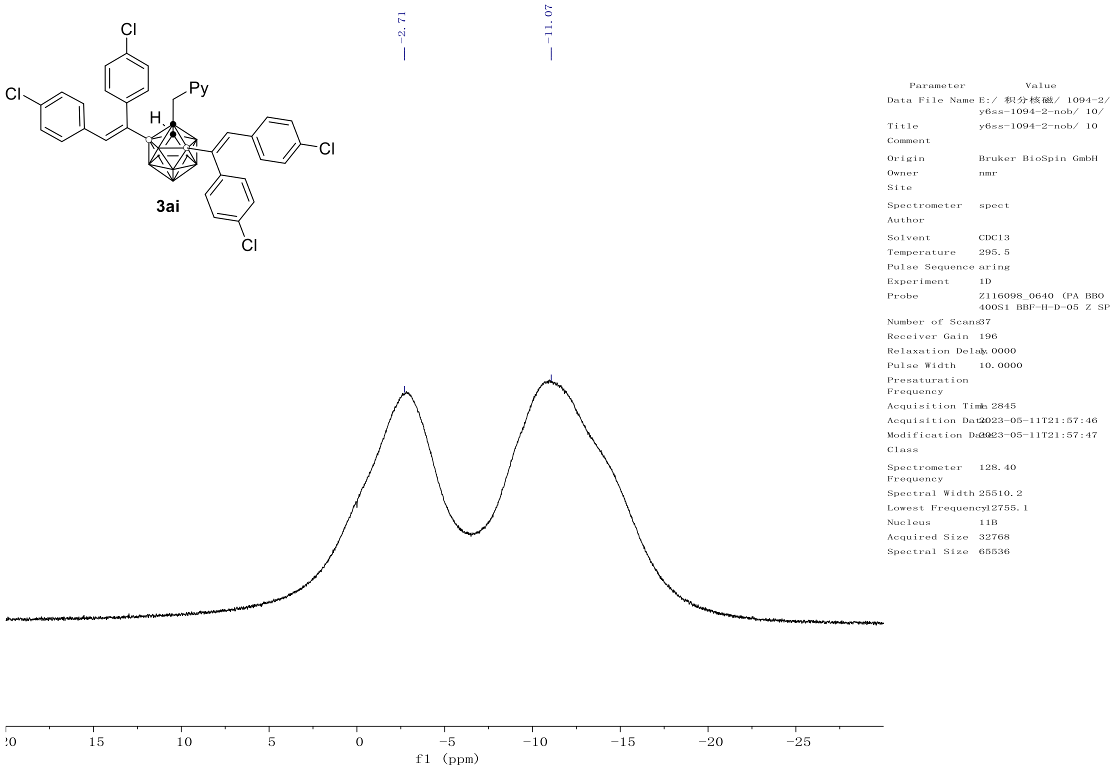

^{1}H NMR (400 MHz, $CDCl_3$) of **3aj**

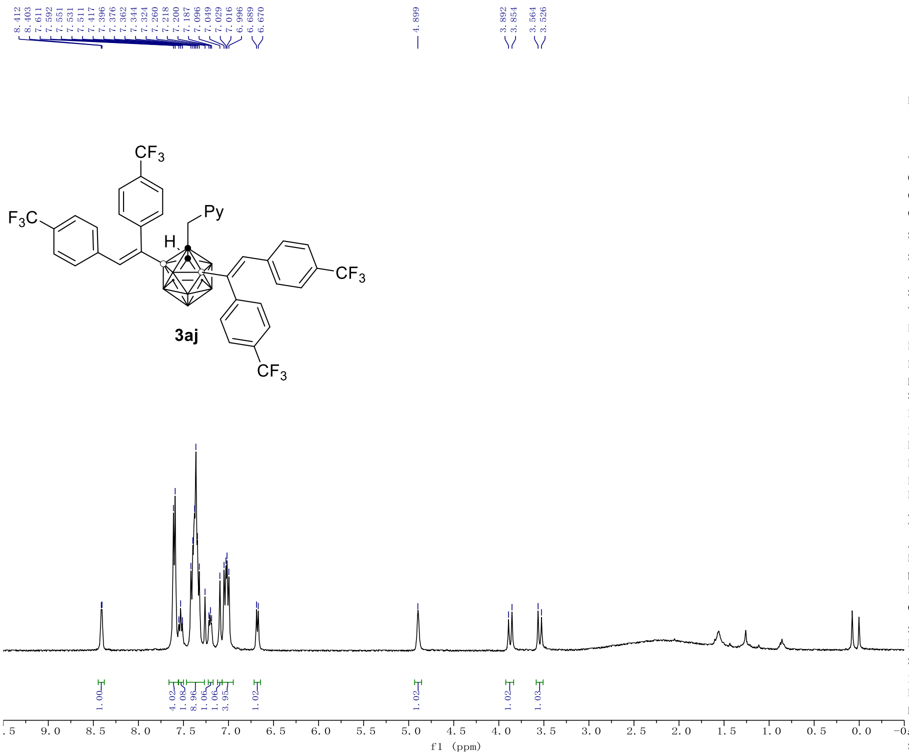

Parameter	Value
Data File Name	E:/ 积分核磁/ 1093-5/ 6yss-1093-5-h.fid/ fid
Title	6yss-1093-5-h
Comment	Std proton
Origin	Varian
Owner	
Site	
Spectrometer	vnmrs
Author	omc1
Solvent	CDC13
Temperature	25.0
Pulse Sequence	s2pul
Experiment	1D
Probe	ATB
Number of Scans	16
Receiver Gain	32
Relaxation Delay	1.0000
Pulse Width	0.0000
Presaturation Frequency	
Acquisition Time	3.0000
Acquisition Date	2023-05-08T16:00:36
Modification Date	2023-05-08T08:02:00
Class	
Spectrometer Frequency	400.03
Spectral Width	8445.9
Lowest Frequency	-1216.0
Nucleus	1H
Acquired Size	25338
Spectral Size	65536

$^{13}C\{^1H\}$ NMR (151 MHz, $CDCl_3$) of **3aj**

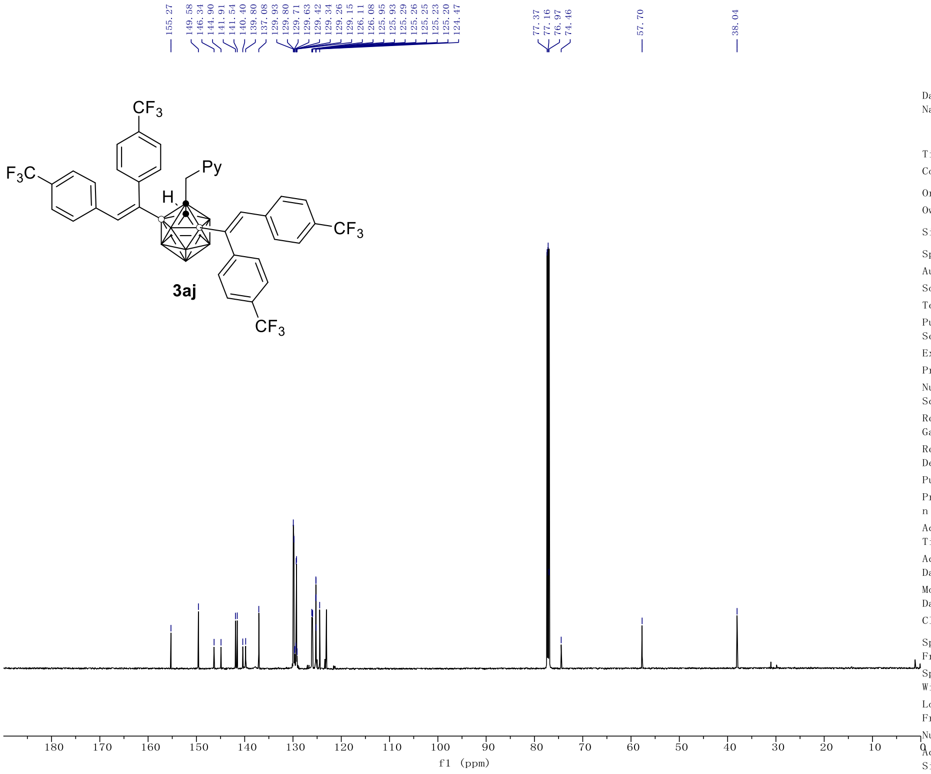

Parameter	Value
Data File Name	E:/ 积分核磁/ 1093-5/ 1093-5_carbon-1-2.jdf
Title	1093-5
Comment	
Origin	JEOL
Owner	
Site	
Spectrometer	ECA
Author	delta
Solvent	CHLOROFORM-D
Temperature	25.0
Pulse Sequence	carbon.jxp
Experiment	1D
Probe	2777
Number of Scans	8000
Receiver Gain	80
Relaxation Delay	1.0000
Pulse Width	5.9300
Presaturation Frequency	
Acquisition Time	1.3200
Acquisition Date	2023-05-09T17:31:07
Modification Date	2023-05-10T08:21:07
Class	
Spectrometer Frequency	150.91
Spectral Width	37878.2
Lowest Frequency	-3835.8
Nucleus	Carbon13
Acquired Size	62500

$^{11}B\{^{1}H\}$ NMR (128 MHz, $CDCl_3$) of **3aj**

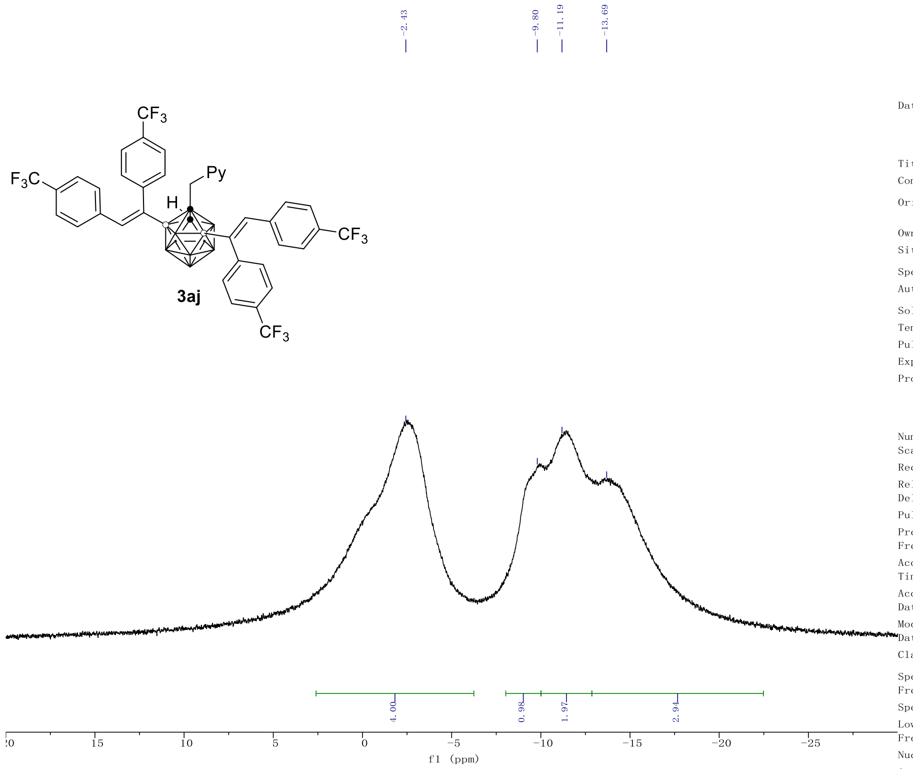

Parameter	Value
Data File Name	E:/ 积分核磁/ 1093-5/ yss-1093-5-b/ 2/ fid
Title	yss-1093-5-b/ 2
Comment	
Origin	Bruker BioSpin GmbH
Owner	nmr
Site	
Spectrometer	spect
Author	
Solvent	CDCl3
Temperature	295.8
Pulse Sequence	aring-b11
Experiment	1D
Probe	Z116098_0640 (PA BBO 400S1 BBF-H-D-05 Z SP)
Number of Scans	129
Receiver Gain	196
Relaxation Delay	1.0000
Pulse Width	10.0000
Presaturation Frequency	
Acquisition Time	1.2845
Acquisition Date	2023-05-08T18:50:01
Modification Date	2023-05-08T18:50:02
Class	
Spectrometer Frequency	128.40
Spectral Width	25510.2
Lowest Frequency	-12755.1
Nucleus	11B
[illegible]	[illegible]

^{11}B NMR (128 MHz, $CDCl_3$) of **3aj**

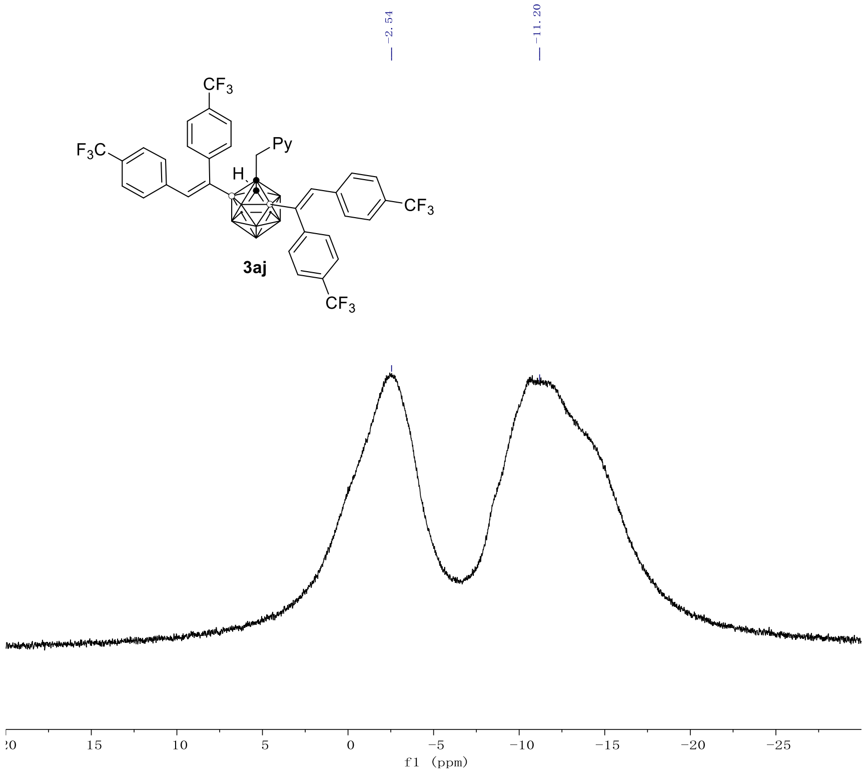

Parameter	Value
Data File Name	E:/ 积分核磁/ 1093-5/ yss-1093-5-nob/ 3/ fi[illegible]
Title	yss-1093-5-nob/ 3
Comment	
Origin	Bruker BioSpin GmbH
Owner	nmr
Site	
Spectrometer	spect
Author	
Solvent	CDCl3
Temperature	295.5
Pulse Sequence	aring
Experiment	1D
Probe	Z116098_0640 (PA BBO 400S1 BBF-H-D-05 Z SP
Number of Scans	130
Receiver Gain	196
Relaxation Delay	1.0000
Pulse Width	10.0000
Presaturation Frequency	
Acquisition Time	1.2845
Acquisition Date	2023-05-08T18:56:50
Modification Date	2023-05-08T18:56:51
Class	
Spectrometer Frequency	128.40
Spectral Width	25510.2
Lowest Frequency	-12755.1
Nucleus	11B
Acquired Size	32768
Spectral Size	65536

^{19}F NMR (565 MHz, $CDCl_3$) of **3aj**

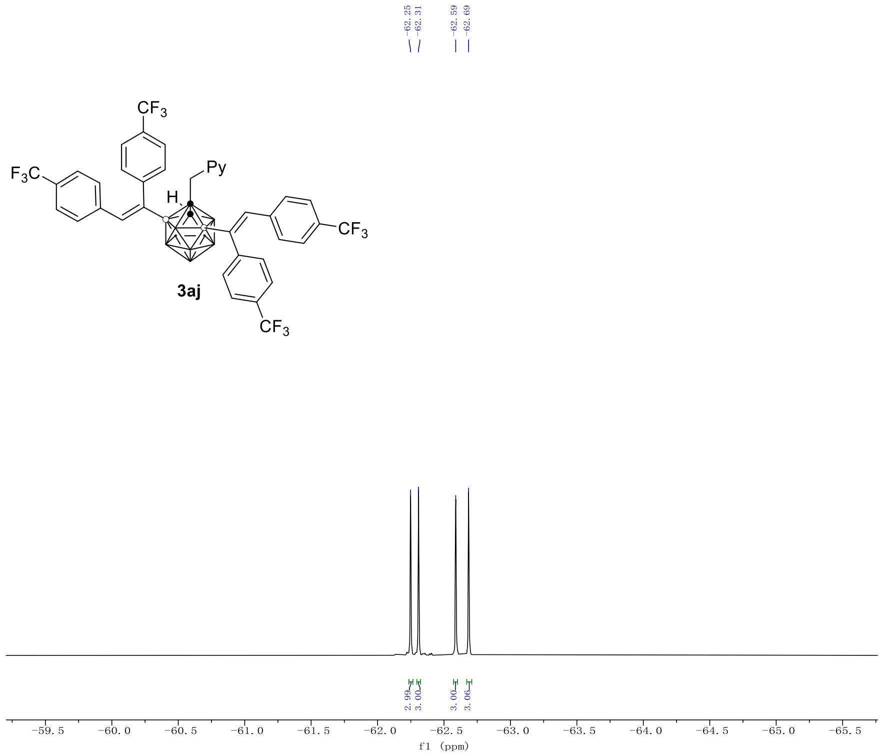

Parameter	Value
Data File Name	E:/ 积分核磁/1093-5/1093-5_19f-1-1.jdf
Title	1093-5
Comment	single_pulse
Origin	JEOL
Owner	
Site	
Spectrometer	ECA
Author	delta
Solvent	CHLOROFORM-D
Temperature	25.0
Pulse Sequence	single_pulse.jxp
Experiment	1D
Probe	2777
Number of Scans	10
Receiver Gain	56
Relaxation Delay	1.0000
Pulse Width	4.6300
Presaturation Frequency	
Acquisition Time	0.9227
Acquisition Date	2023-05-09T17:29:21
Modification Date	2023-05-09T17:30:09
Class	
Spectrometer Frequency	564.73
Spectral Width	227272.7
Lowest Frequency	-170109.0
Nucleus	Fluorine19
Acquired Size	262144
Spectral Size	209716

^{1}H NMR (400 MHz, $CDCl_3$) of **3ak**

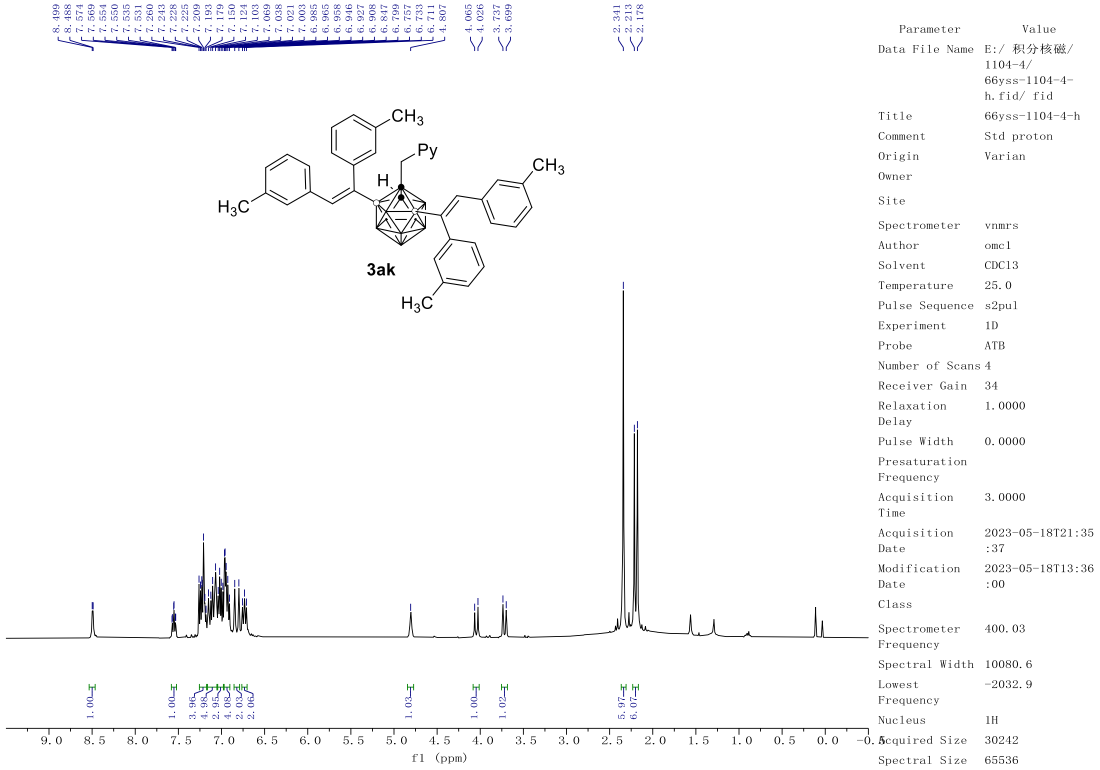

$^{13}C\{^{1}H\}$ NMR (101 MHz, $CDCl_3$) of **3ak**

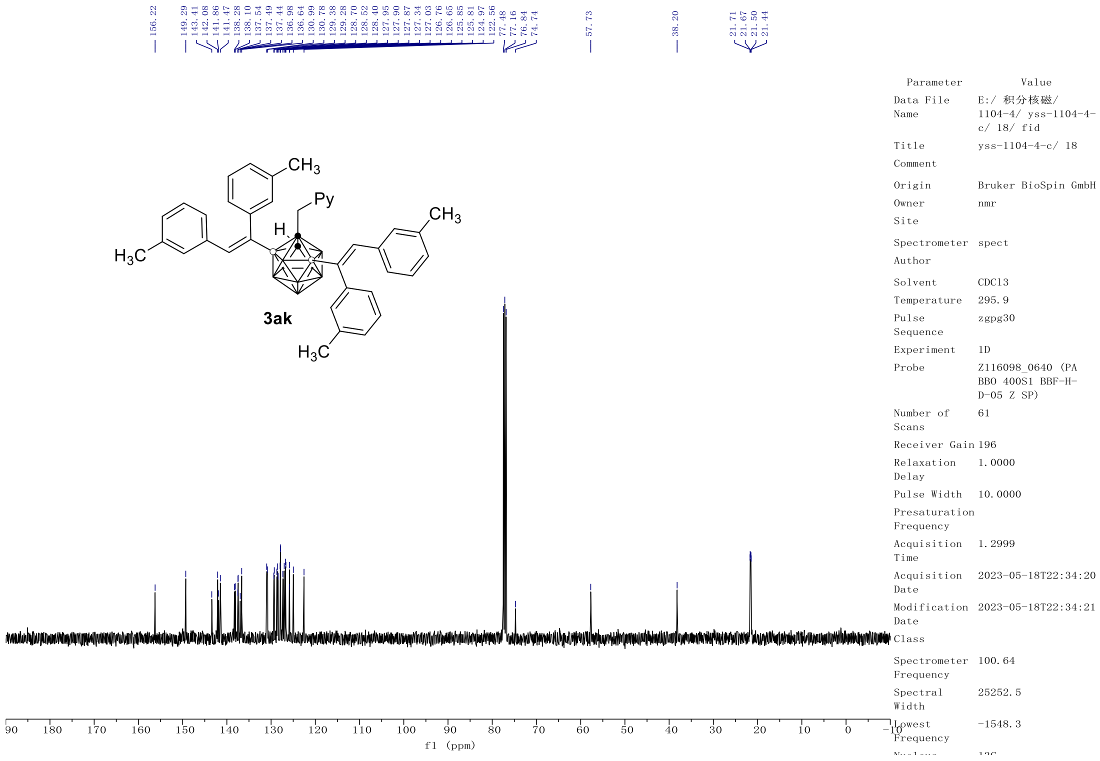

$^{11}B\{^{1}H\}$ NMR (128 MHz, $CDCl_3$) of **3ak**

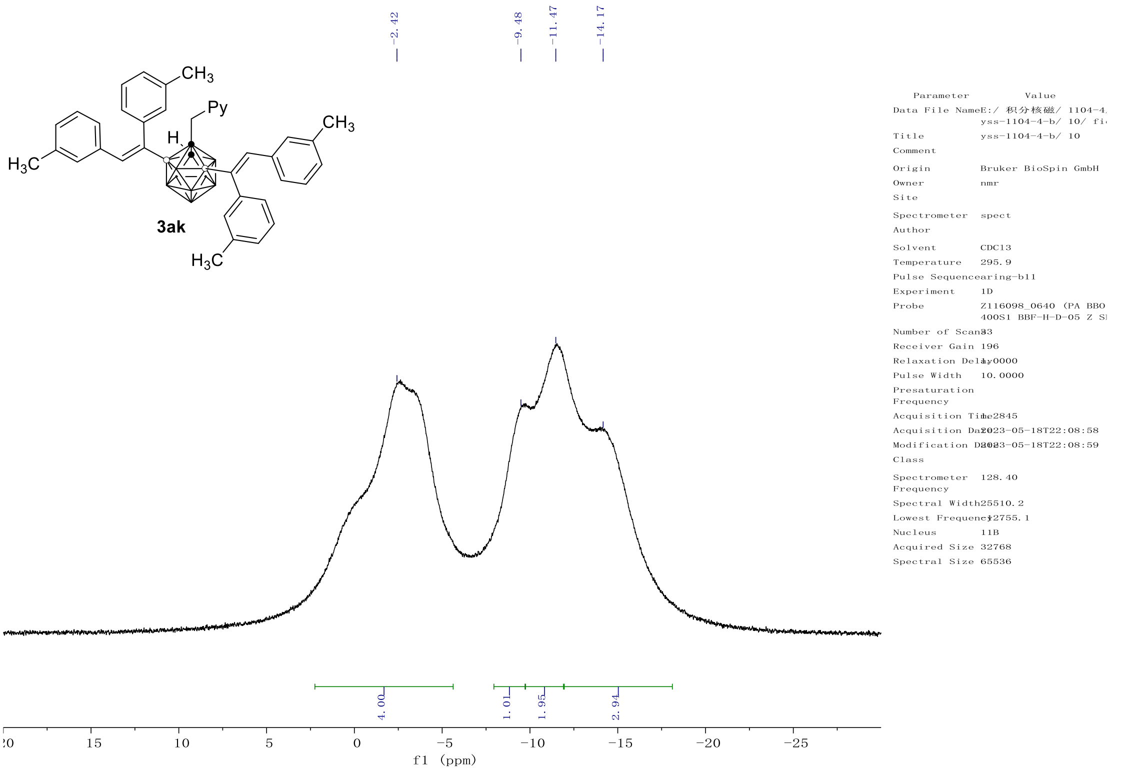

^{11}B NMR (128 MHz, $CDCl_3$) of **3ak**

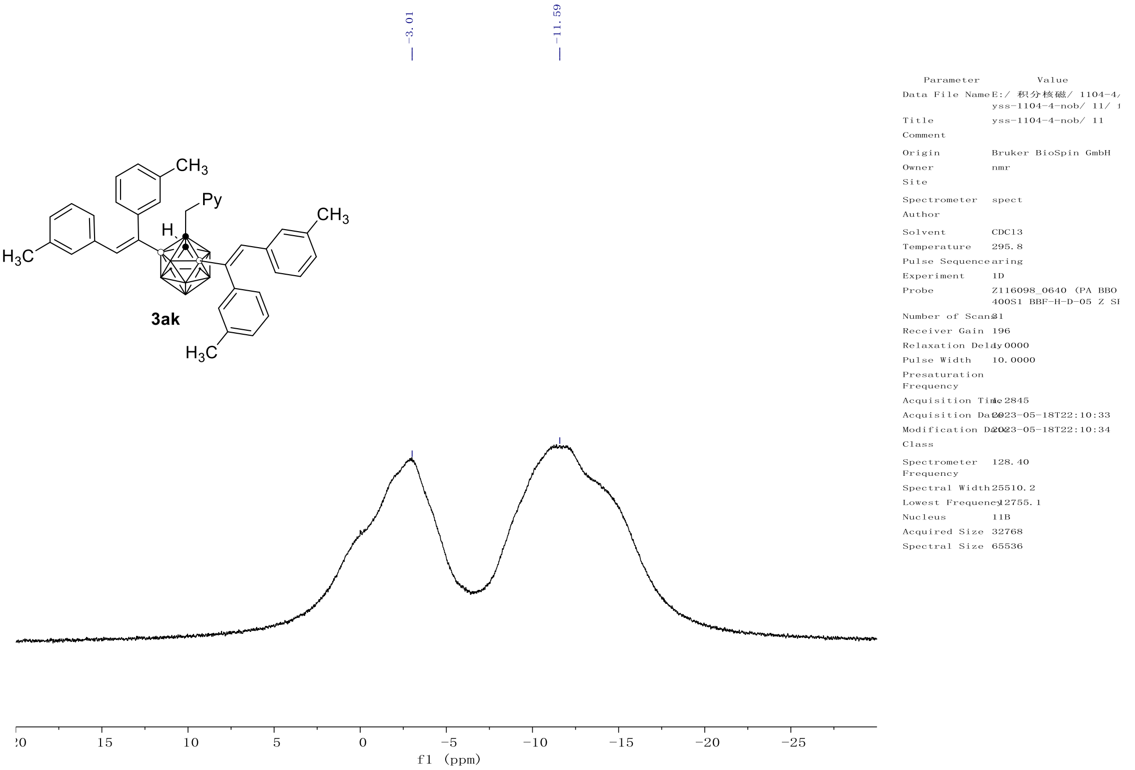

^{1}H NMR (400 MHz, $CDCl_3$) of **3al**

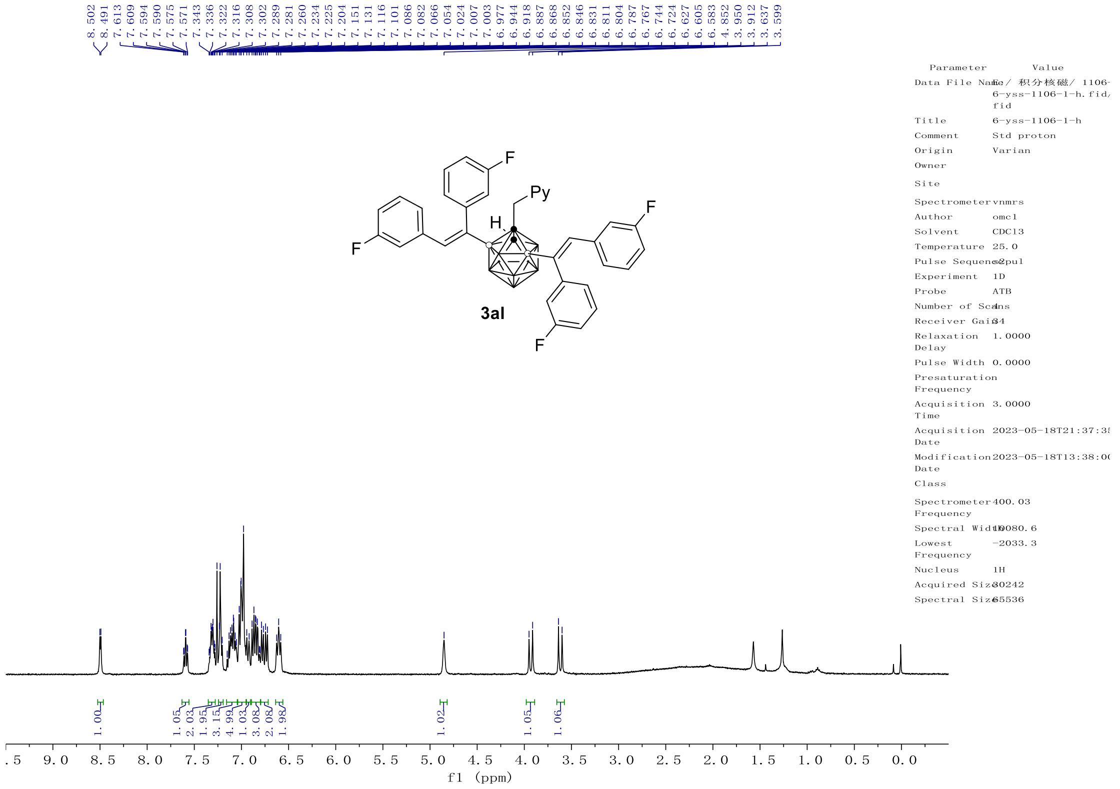

^{13}C{^{1}H} NMR (101 MHz, $CDCl_3$) of **3al**

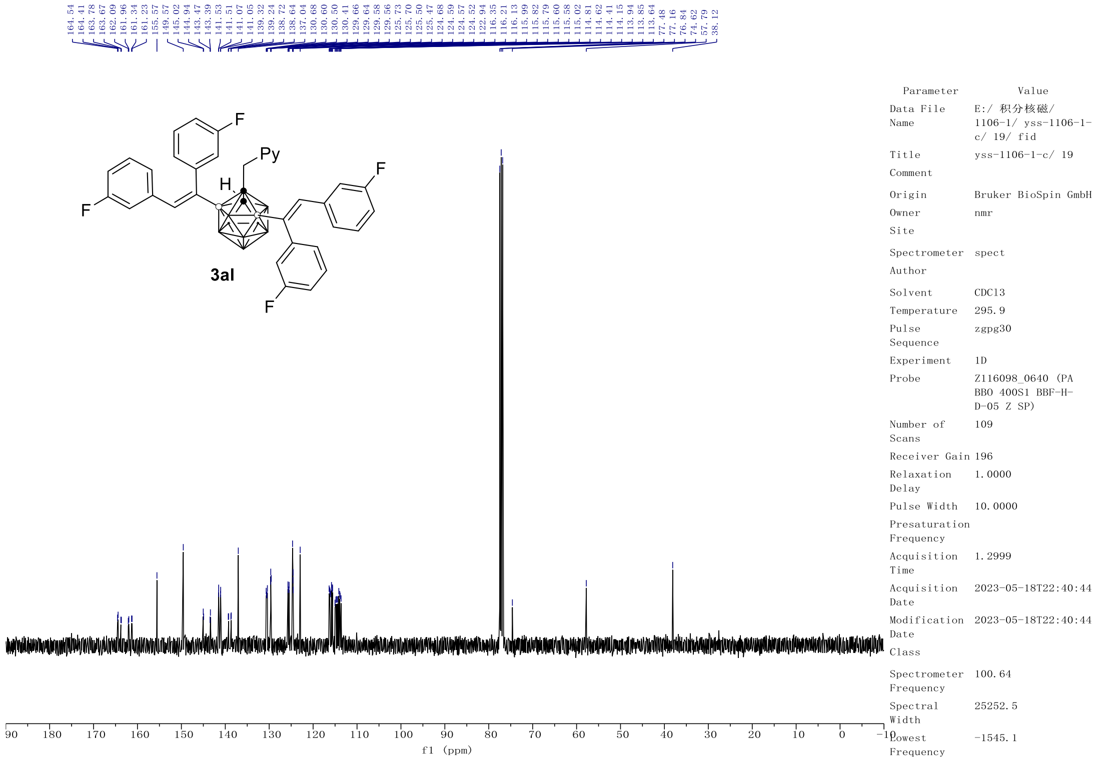

$^{11}B\{^1H\}$ NMR (128 MHz, $CDCl_3$) of **3al**

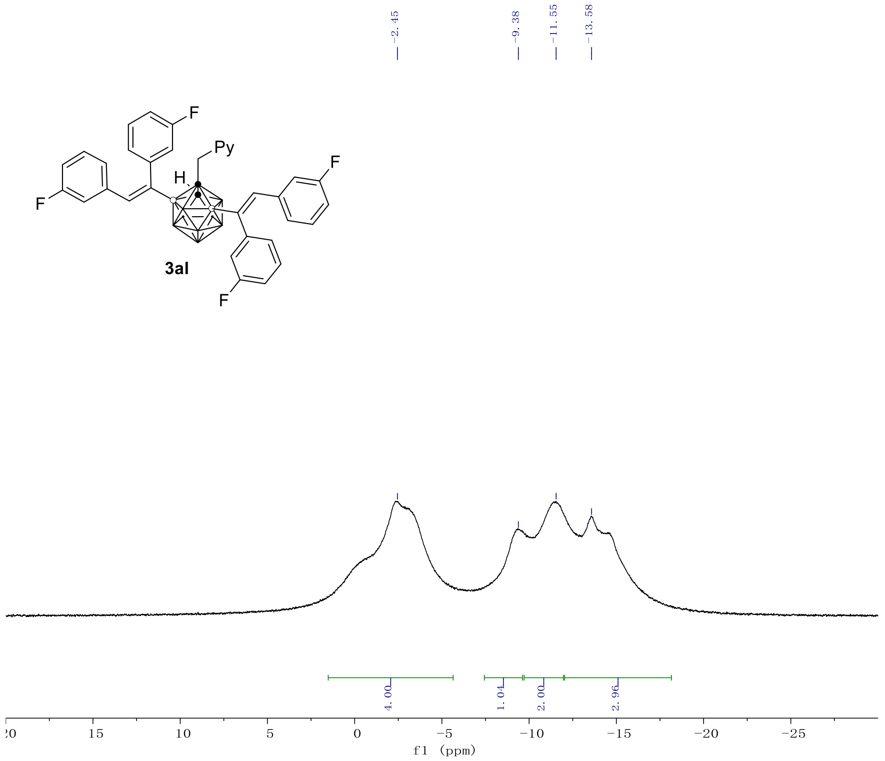

Parameter	Value
Data File Name	E:/ 积分核磁/ 1106-1/ yss-1106-1-b/ 9/ fid
Title	yss-1106-1-b/ 9
Comment	
Origin	Bruker BioSpin GmbH
Owner	nmr
Site	
Spectrometer	spect
Author	
Solvent	CDCl3
Temperature	295.8
Pulse Sequence	aring-b11
Experiment	1D
Probe	Z116098_0640 (PA BBO 400S1 BBF-H-D-05 Z SP
Number of Scans	32
Receiver Gain	196
Relaxation Delay	1.0000
Pulse Width	10.0000
Presaturation Frequency	
Acquisition Time	1.2845
Acquisition Date	2023-05-18T22:06:53
Modification Date	2023-05-18T22:06:54
Class	
Spectrometer Frequency	128.40
Spectral Width	25510.2
Lowest Frequency	-12755.1
Nucleus	11B
Acquired Size	32768
Spectral Size	65536

^{11}B NMR (128 MHz, $CDCl_3$) of **3al**

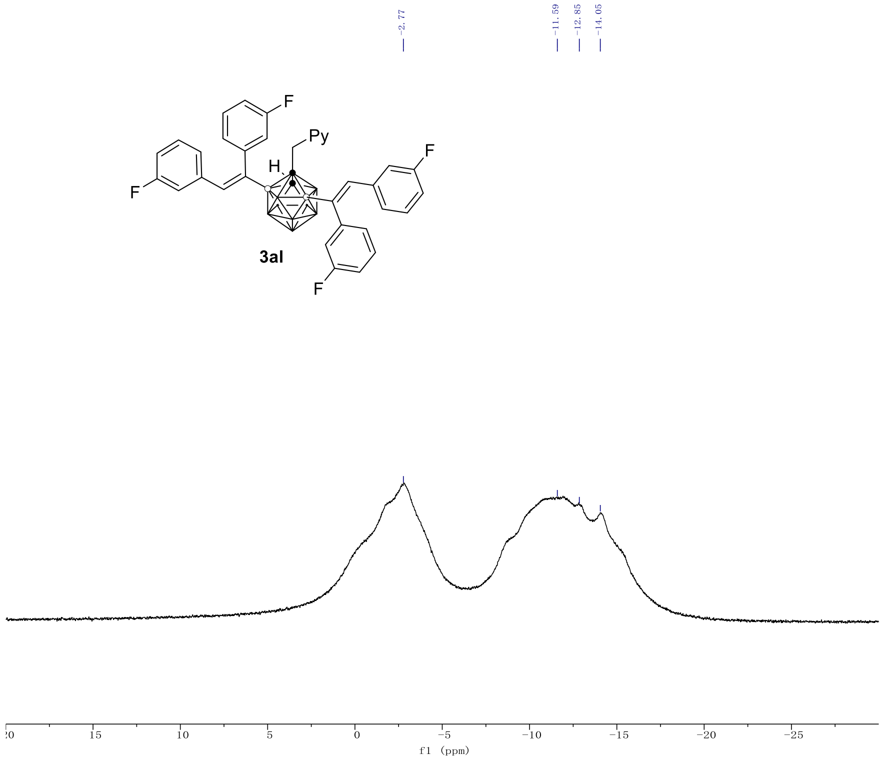

Parameter	Value
Data File Name	E:/ 积分核磁/ 1106-1/ yss-1106-1-nob/ 8/ fid
Title	yss-1106-1-nob/ 8
Comment	
Origin	Bruker BioSpin GmbH
Owner	nmr
Site	
Spectrometer	spect
Author	
Solvent	CDCl3
Temperature	295.7
Pulse Sequence	aring
Experiment	1D
Probe	Z116098_0640 (PA BBO 400S1 BBF-H-D-05 Z SP)
Number of Scans	35
Receiver Gain	196
Relaxation Delay	1.0000
Pulse Width	10.0000
Presaturation Frequency	
Acquisition Time	1.2845
Acquisition Date	2023-05-18T22:05:16
Modification Date	2023-05-18T22:05:17
Class	
Spectrometer Frequency	128.40
Spectral Width	25510.2
Lowest Frequency	-12755.1

^{19}F NMR (376 MHz, $CDCl_3$) of **3al**

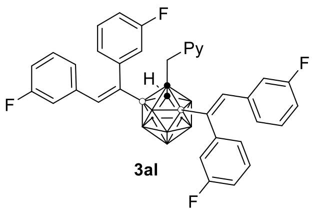

3al

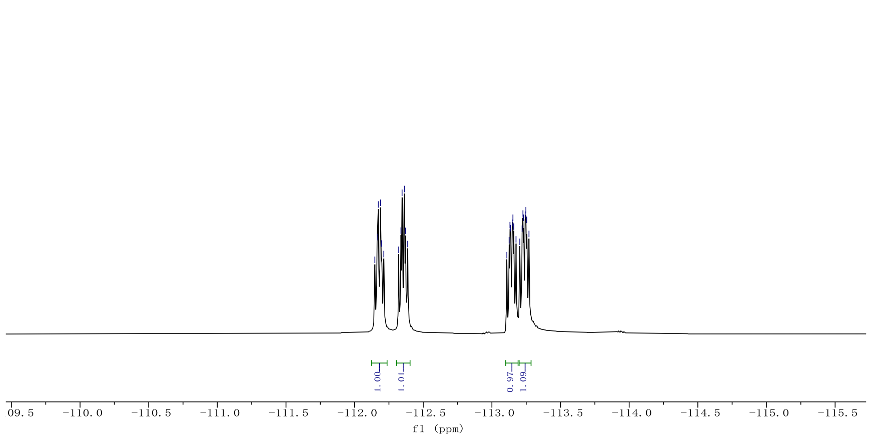

Parameter	Value
Data File Name	E:/ 积分核磁/ 1106-1/ yss-1106-1-f/ 20/ fid
Title	yss-1106-1-f/ 20
Comment	
Origin	Bruker BioSpin GmbH
Owner	nmr
Site	
Spectrometer	spect
Author	
Solvent	CDCl3
Temperature	295.8
Pulse Sequence	zgflqn
Experiment	1D
Probe	Z116098_0640 (PA BBO 400S1 BBF-H-D-05 Z SP)
Number of Scans	14
Receiver Gain	196
Relaxation Delay	1.0000
Pulse Width	18.0000
Presaturation Frequency	
Acquisition Time	0.9999
Acquisition Date	2023-05-18T22:43:52
Modification Date	2023-05-18T22:43:52
Class	
Spectrometer Frequency	376.53
Spectral Width	150000.0
Lowest Frequency	-112656.4
Nucleus	19F
Acquired Size	149992
Spectral Size	524288

^{1}H NMR (400 MHz, $CDCl_3$) of **3am**

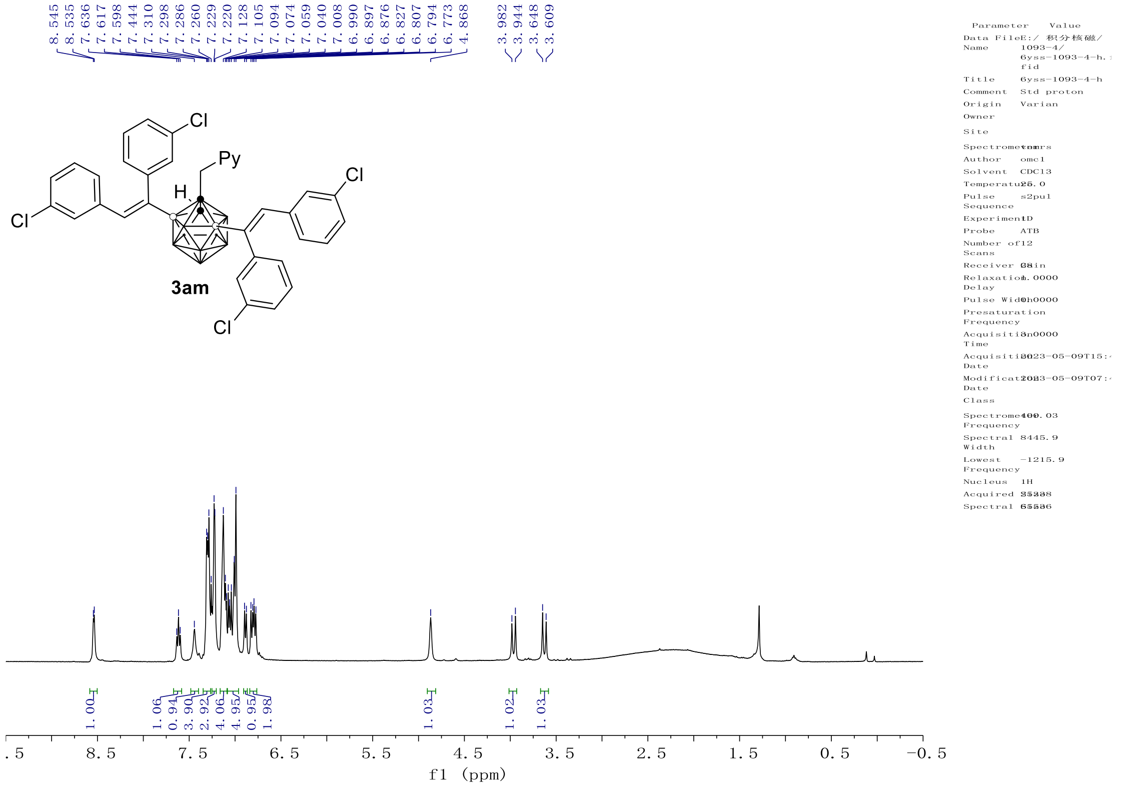

$^{13}C\{^{1}H\}$ NMR (101 MHz, $CDCl_3$) of **3am**

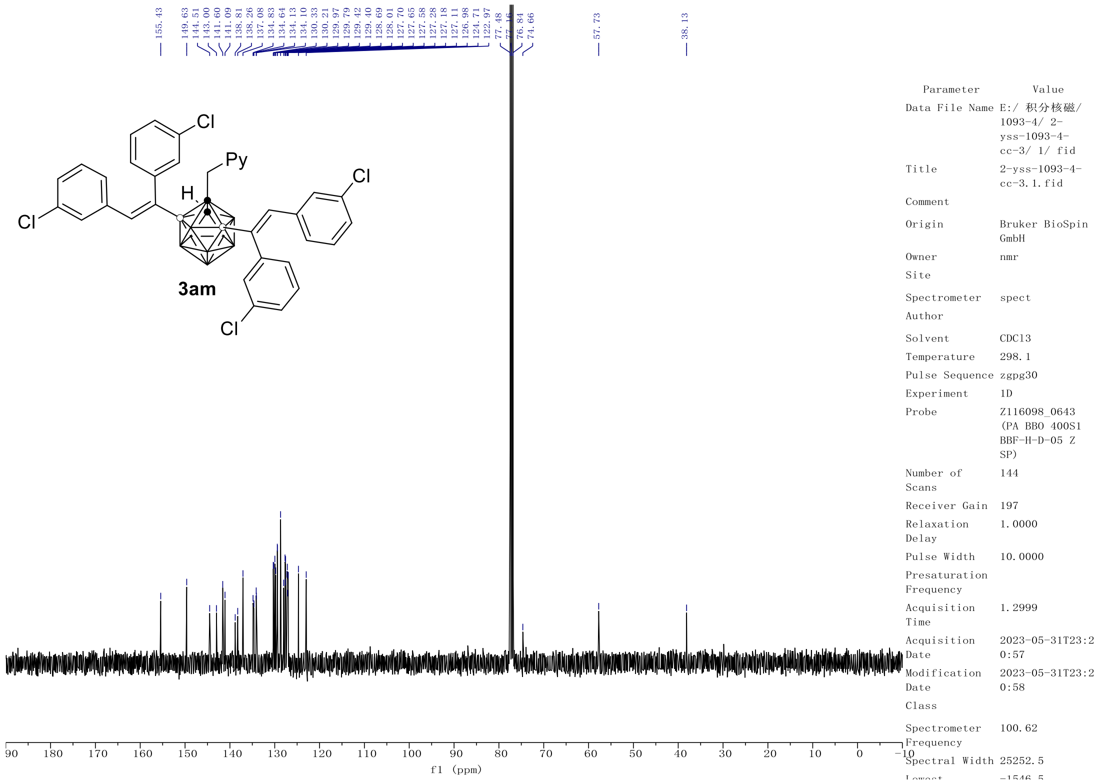

$^{11}B\{^{1}H\}$ NMR (128 MHz, $CDCl_3$) of **3am**

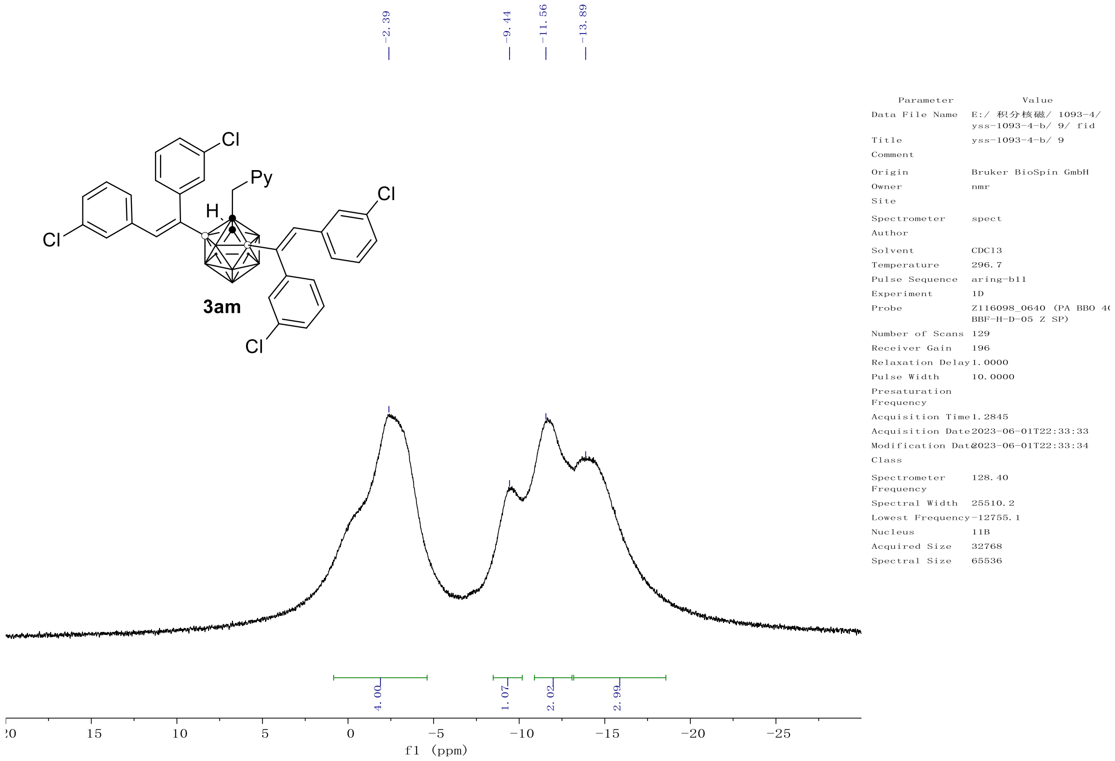

^{11}B NMR (128 MHz, $CDCl_3$) of **3am**

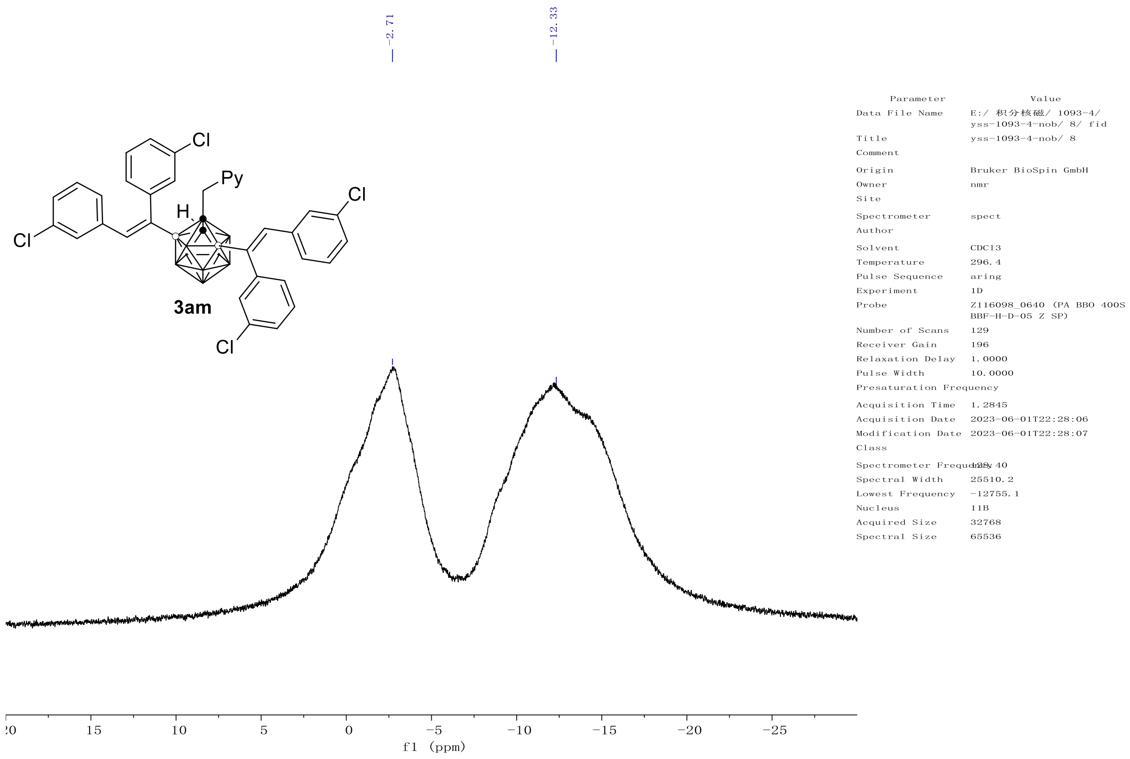

^{1}H NMR (400 MHz, $CDCl_3$) of **3an**

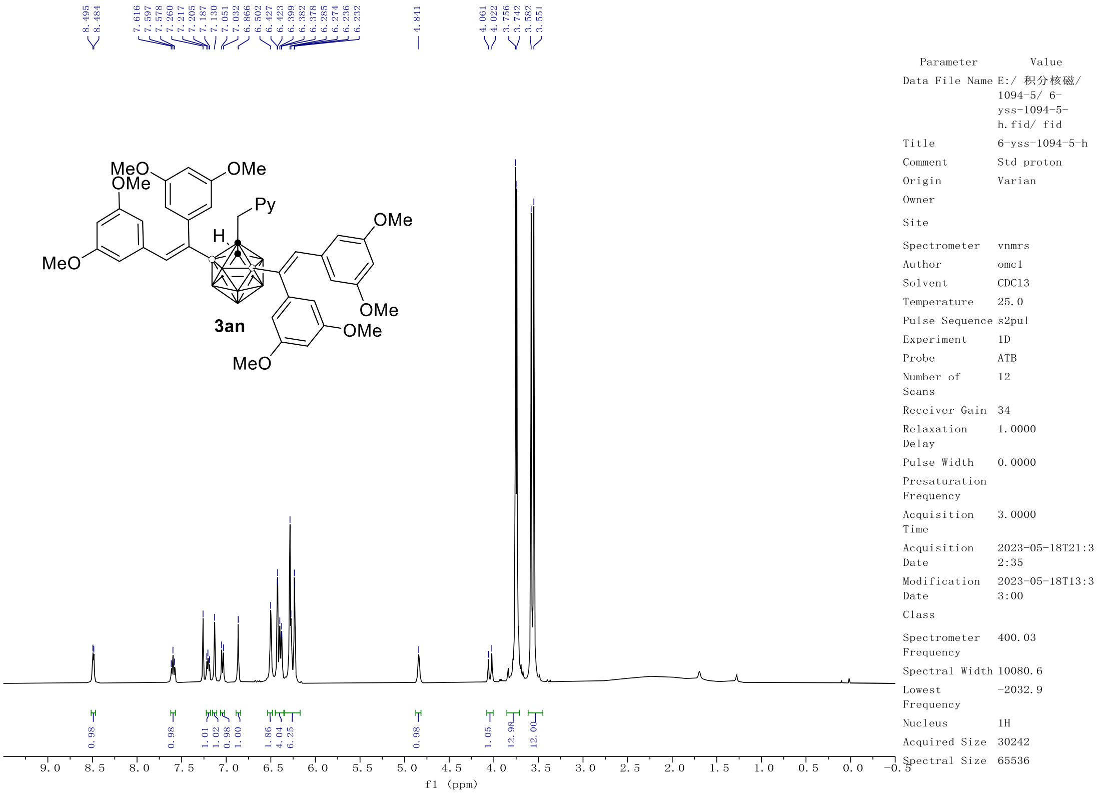

$^{13}C\{^{1}H\}$ NMR (101 MHz, $CDCl_3$) of **3an**

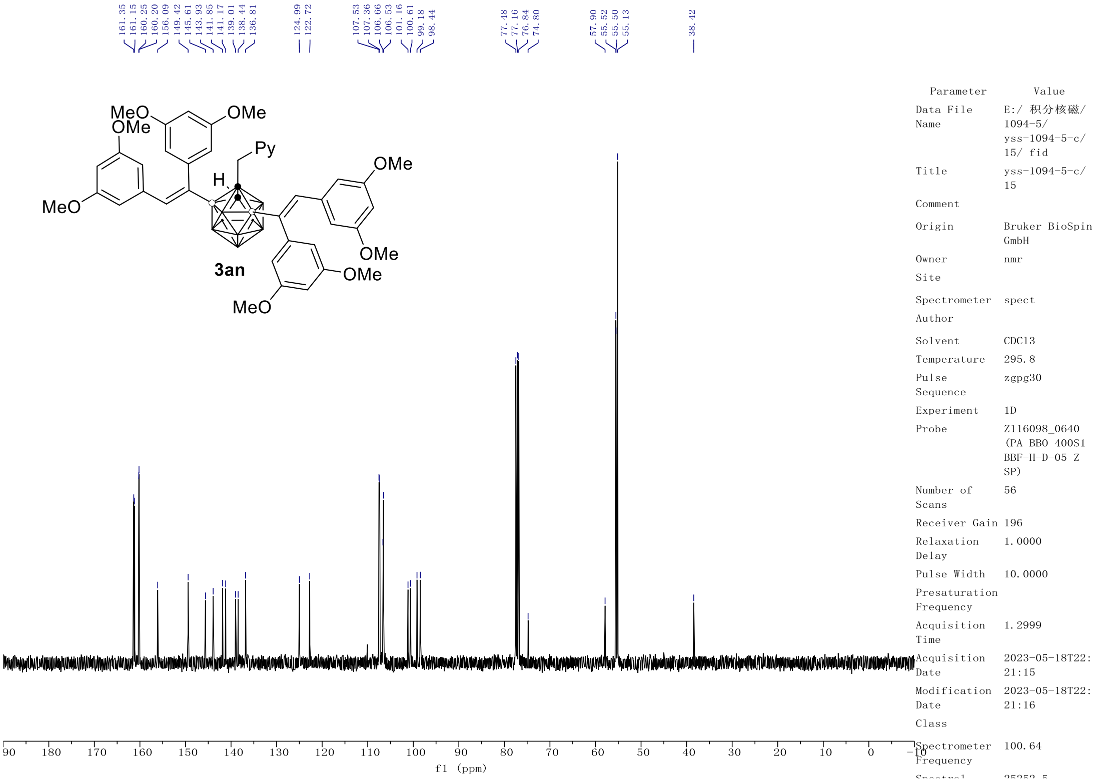

$^{11}B\{^{1}H\}$ NMR (128 MHz, $CDCl_3$) of **3an**

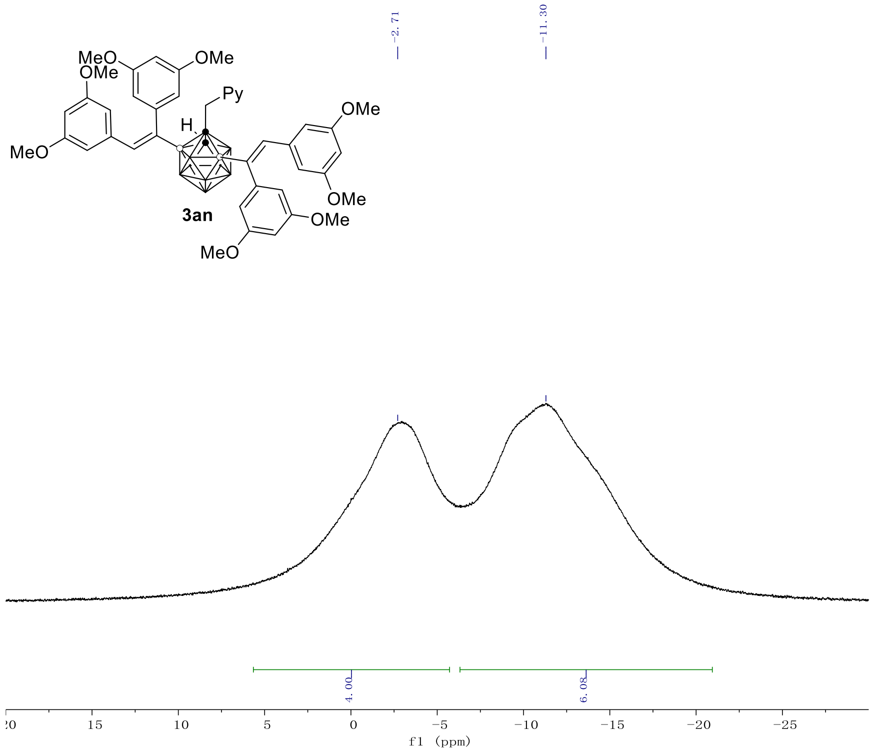

Parameter	Value
Data File Name	E:/ 积分核磁/ 1094-5/ yss-1094-5-b/ 13/ fid
Title	yss-1094-5-b/ 13
Comment	
Origin	Bruker BioSpin GmbH
Owner	nmr
Site	
Spectrometer	spect
Author	
Solvent	CDCl3
Temperature	296.0
Pulse Sequence	aring-b11
Experiment	1D
Probe	Z116098_0640 (PA BBO 4(BBF-H-D-05 Z SP)
Number of Scans	31
Receiver Gain	196
Relaxation Delay	1.0000
Pulse Width	10.0000
Presaturation Frequency	
Acquisition Time	1.2845
Acquisition Date	2023-05-18T22:14:02
Modification Date	2023-05-18T22:14:03
Class	
Spectrometer Frequency	128.40
Spectral Width	25510.2
Lowest Frequency	-12755.1
Nucleus	11B
Acquired Size	32768
Spectral Size	65536

^{11}B NMR (128 MHz, $CDCl_3$) of **3an**

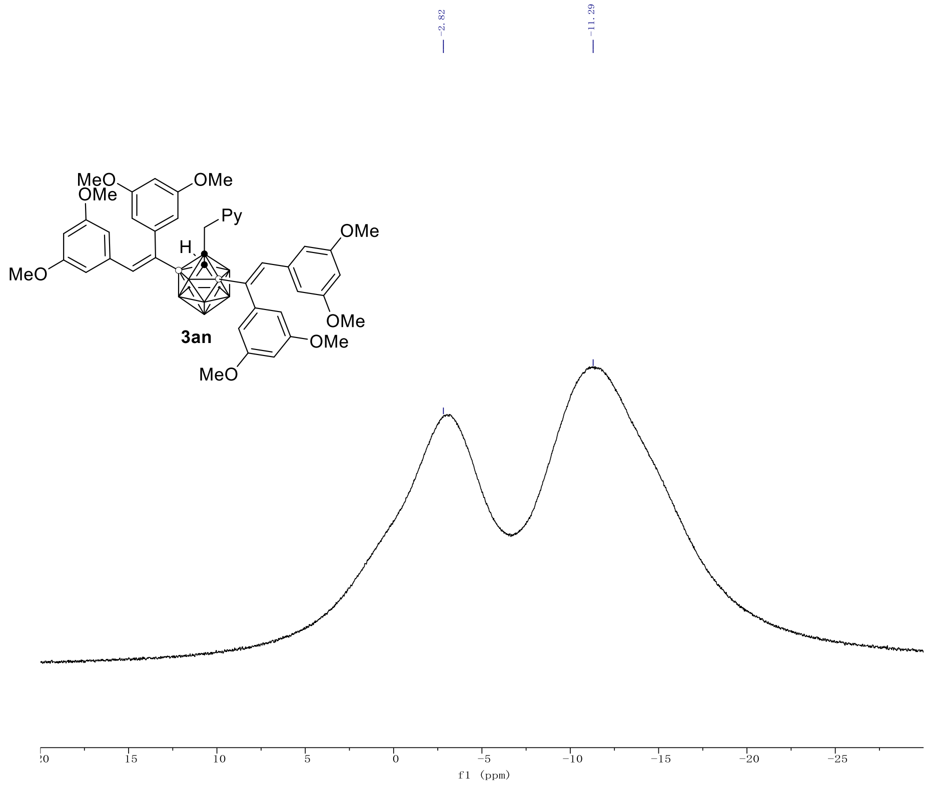

Parameter	Value
Data File Name	E:/ 积分核磁/ 2040/ yss-2040-nob/ 9/ fid
Title	yss-2040-nob/ 9
Comment	
Origin	Bruker BioSpin GmbH
Owner	nmr
Site	
Spectrometer	spect
Author	
Solvent	CDCl3
Temperature	295.9
Pulse Sequence	aring
Experiment	1D
Probe	Z116098_0640 (PA BBO 400S1 BBF-H-D-05 Z SP)
Number of Scans	130
Receiver Gain	196
Relaxation Delay	1.0000
Pulse Width	10.0000
Presaturation Frequency	
Acquisition Time	1.2845
Acquisition Date	2023-05-15T22:05:45
Modification Date	2023-05-15T22:05:46
Class	
Spectrometer Frequency	128.40
Spectral Width	25510.2
Lowest Frequency	-12755.1
Nucleus	11B
Acquired Size	32768

^{1}H NMR (400 MHz, $CDCl_3$) of **3ao**

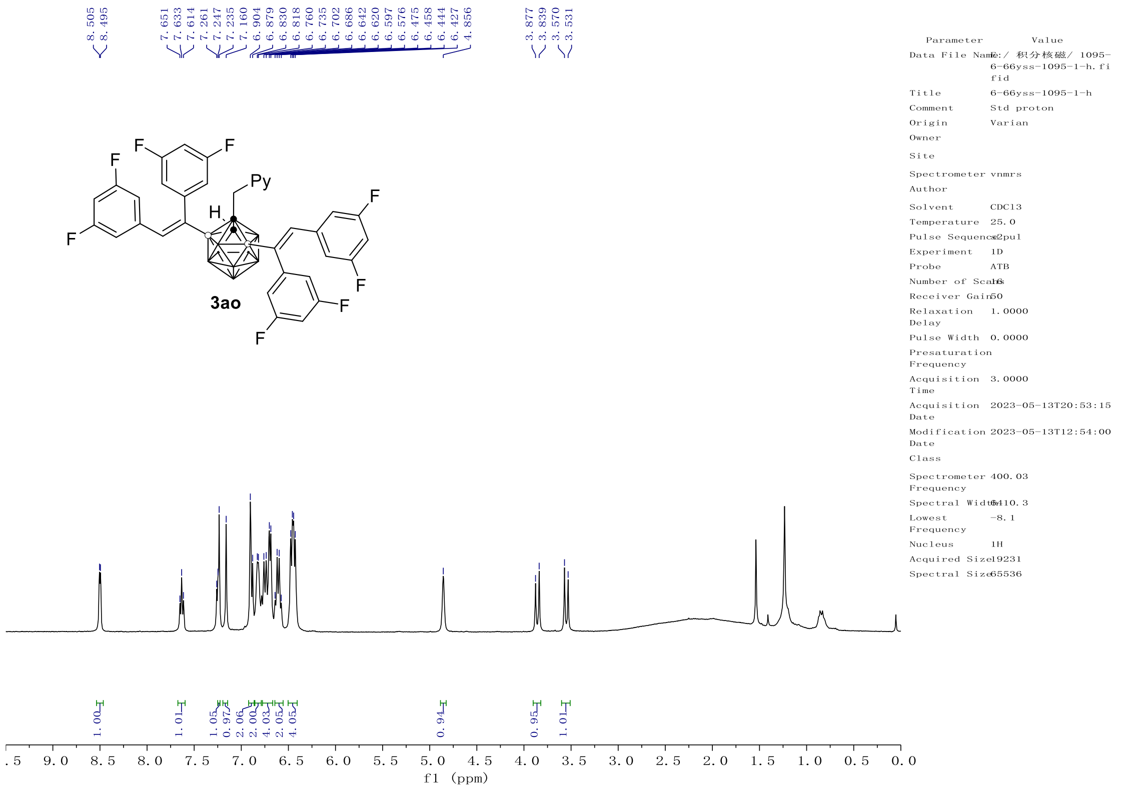

$^{13}C\{^{1}H\}$ NMR (101 MHz, $CDCl_3$) of **3ao**

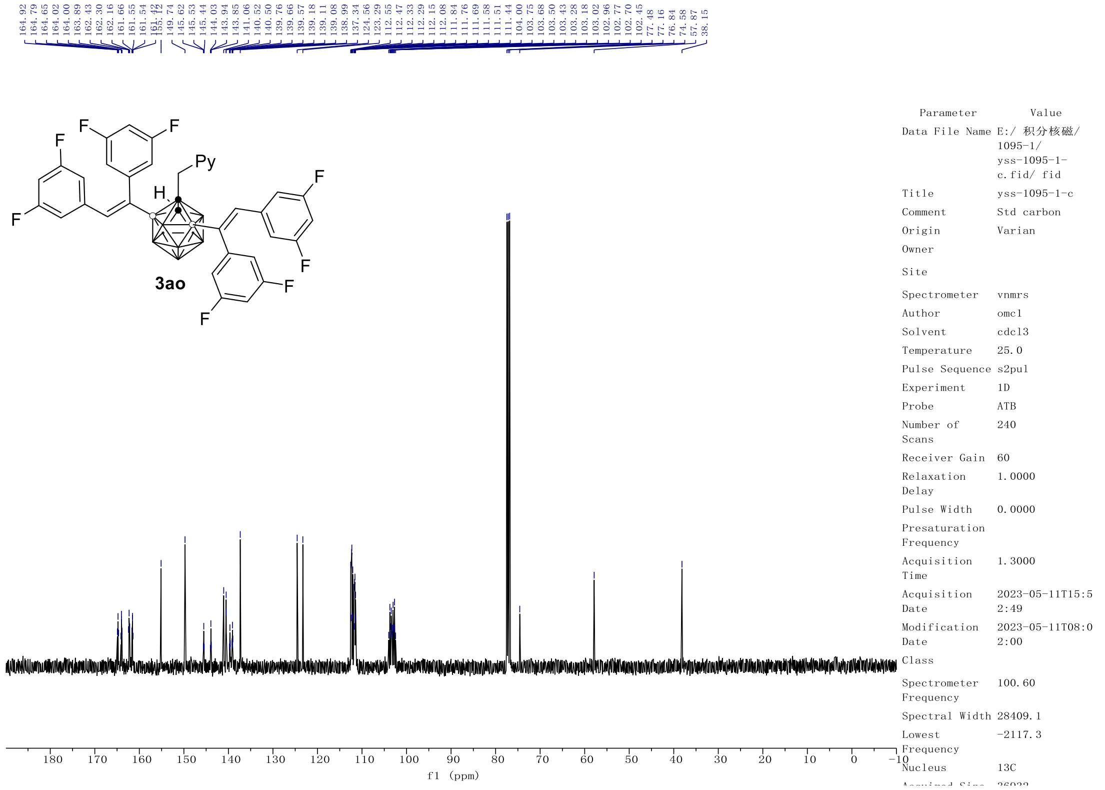

$^{11}B\{^{1}H\}$ NMR (128 MHz, $CDCl_3$) of **3ao**

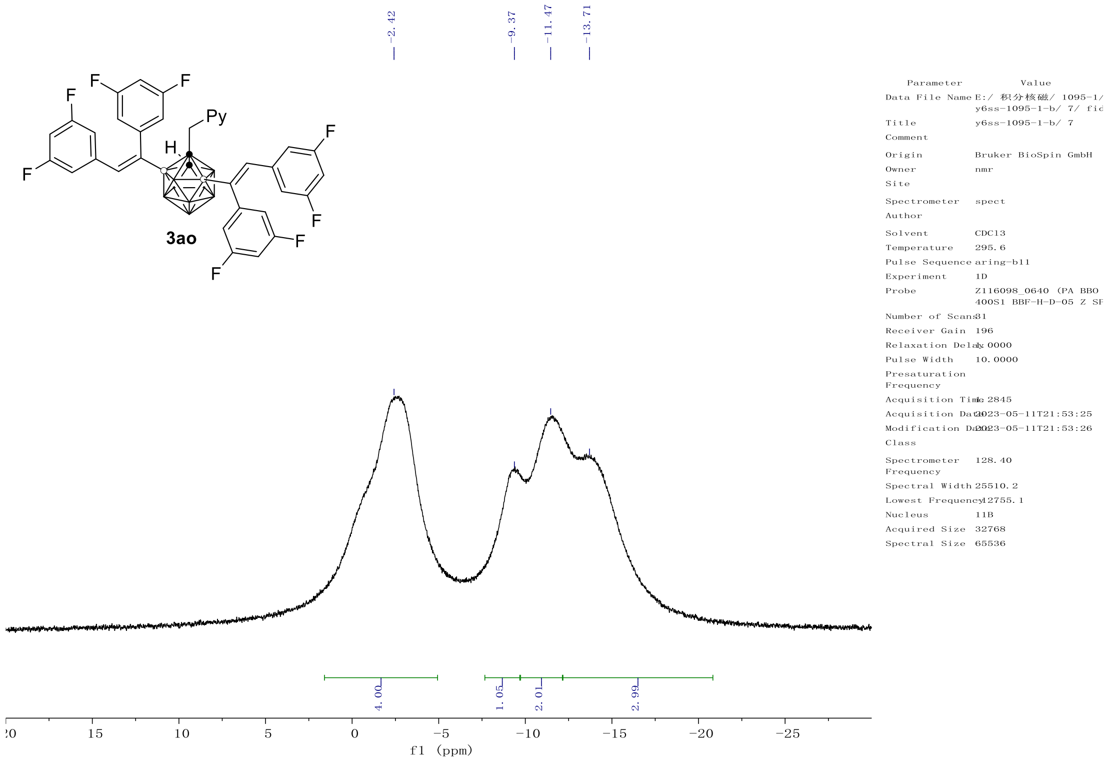

^{11}B NMR (128 MHz, $CDCl_3$) of **3ao**

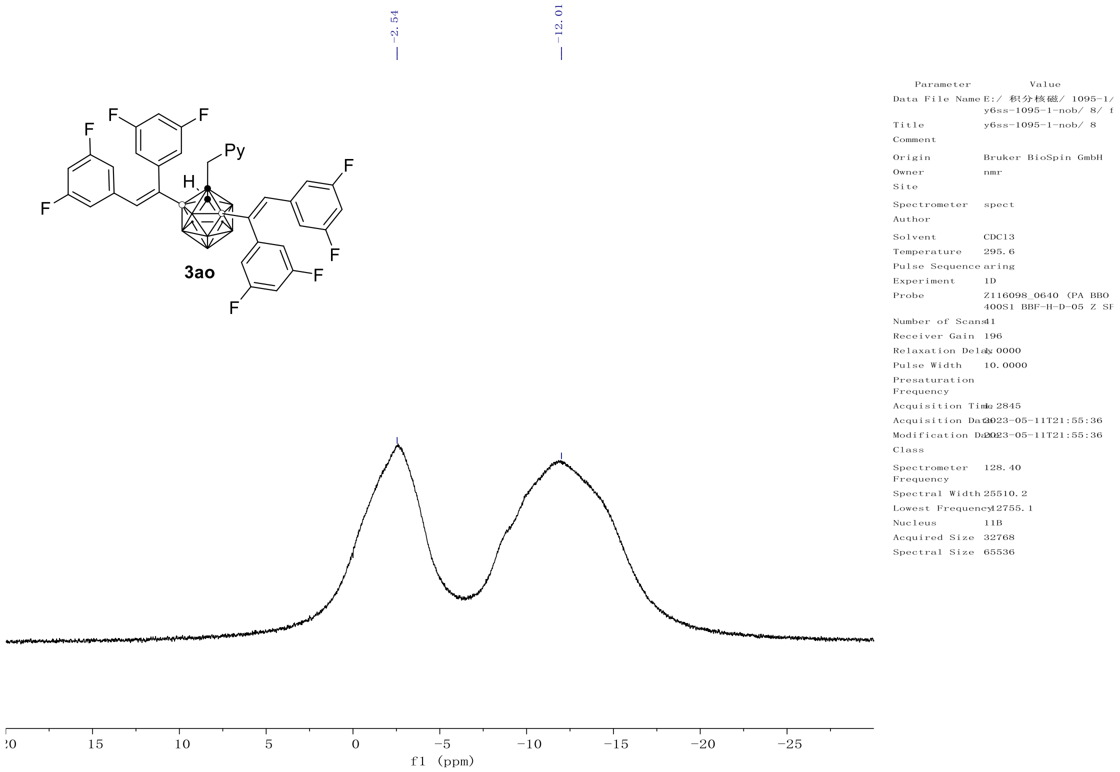

^{19}F NMR (376 MHz, $CDCl_3$) of **3ao**

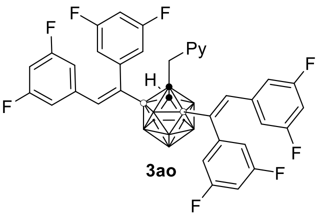

3ao

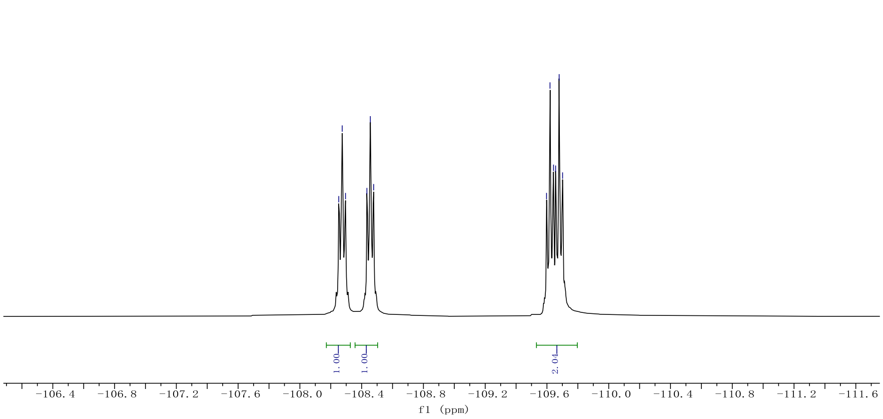

Parameter	Value
Data File Name	E:/ 积分核磁/ 1095-1/ yss-1095-1-f/ 4/ fid
Title	yss-1095-1-f/ 4
Comment	
Origin	Bruker BioSpin GmbH
Owner	nmr
Site	
Spectrometer	spect
Author	
Solvent	CDCl3
Temperature	295.6
Pulse Sequence	zgflqn
Experiment	1D
Probe	Z116098_0640 (PA BBO 400S1 BBF-H-D-05 Z SP)
Number of Scans	19
Receiver Gain	196
Relaxation Delay	1.0000
Pulse Width	18.0000
Presaturation Frequency	
Acquisition Time	0.8000
Acquisition Date	2023-05-13T21:54:59
Modification Date	2023-05-13T21:54:59
Class	
Spectrometer Frequency	376.53
Spectral Width	300000.0
Lowest Frequency	-187656.4

^{1}H NMR (400 MHz, $CDCl_3$) of **3aq**

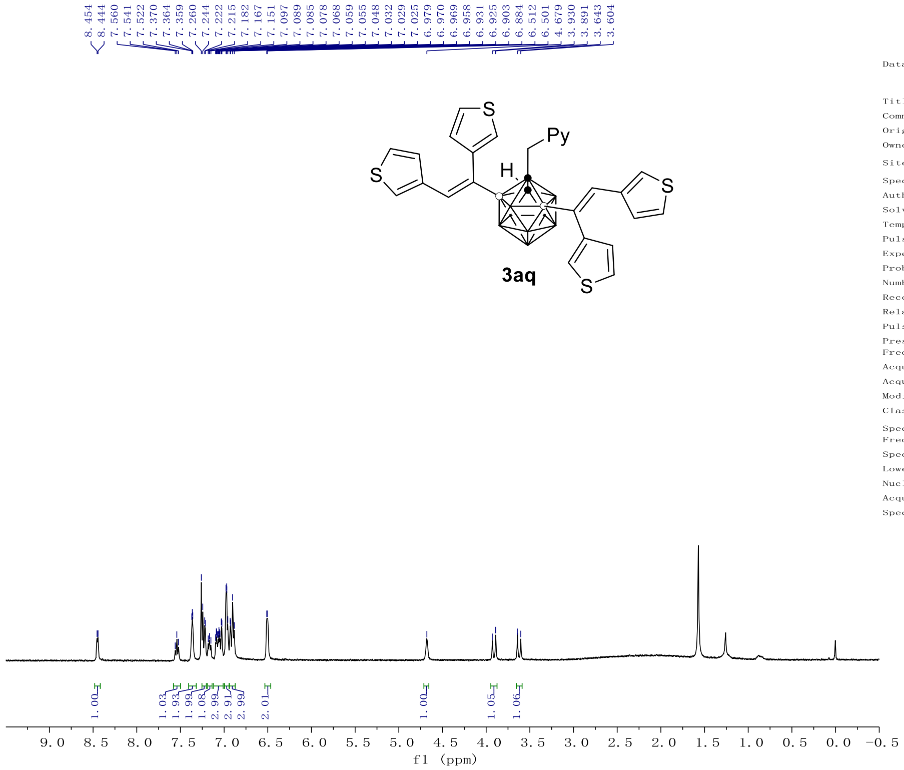

Parameter	Value
Data File Name	E:/ 积分核磁/ 2002-2/ 666-yss-2002-h-hh.fid fid
Title	666-yss-2002-h-hh
Comment	Std proton
Origin	Varian
Owner	
Site	
Spectrometer	vnmrs
Author	omcl
Solvent	CDCl3
Temperature	25.0
Pulse Sequence	s2pul
Experiment	1D
Probe	ATB
Number of Scans	8
Receiver Gain	38
Relaxation Delay	1.0000
Pulse Width	0.0000
Presaturation Frequency	
Acquisition Time	3.0000
Acquisition Date	2023-05-31T21:34:23
Modification Date	2023-05-31T13:35:00
Class	
Spectrometer Frequency	400.03
Spectral Width	10080.6
Lowest Frequency	2032.8
Nucleus	1H
Acquired Size	30242
Spectral Size	65536

$^{13}C\{^{1}H\}$ NMR (101 MHz, $CDCl_3$) of **3aq**

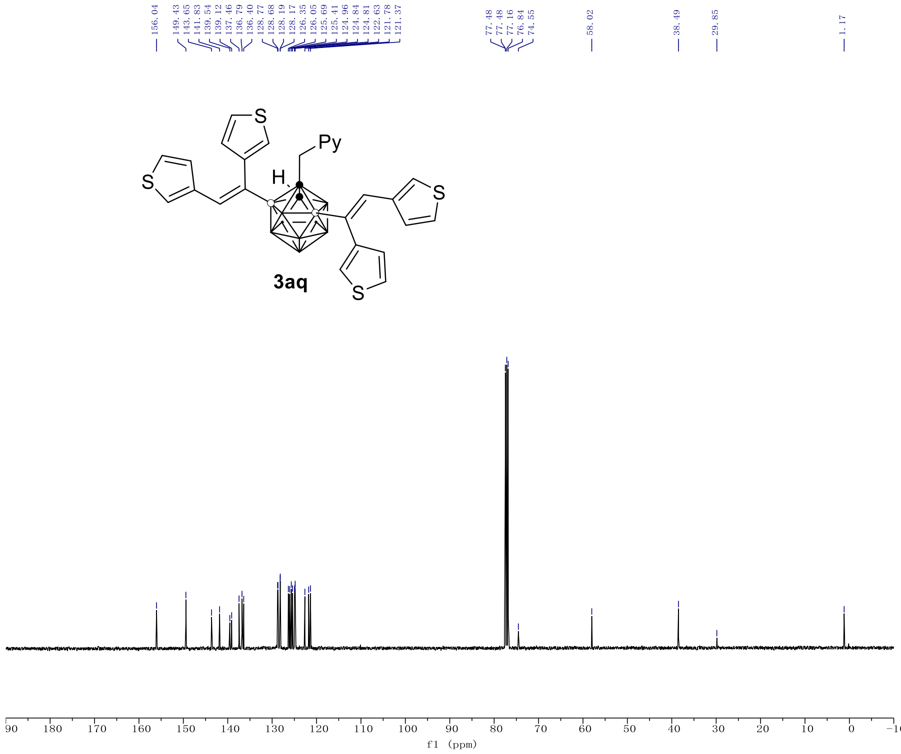

Parameter	Value
Data File Name	E:/ 积分核磁/ 2002-2/ yss-2002-2-c-2/ 1/ fid
Title	yss-2022-2-c-2.1.fid
Comment	
Origin	Bruker BioSpin GmbH
Owner	nmr
Site	
Spectrometer	spect
Author	
Solvent	CDCl3
Temperature	298.2
Pulse Sequence	zgpg30
Experiment	1D
Probe	Z116098_0643 (PA BBO 400S1 BBF-H-D-05 Z SP)
Number of Scans	1024
Receiver Gain	197
Relaxation Delay	1.0000
Pulse Width	10.0000
Presaturation Frequency	
Acquisition Time	1.2999
Acquisition Date	2023-05-14T19:50:16
Modification Date	2023-05-14T19:50:16
Class	
Spectrometer Frequency	100.62
Spectral Width	25252.5
Lowest Frequency	-1547.7

$^{11}B\{^{1}H\}$ NMR (128 MHz, $CDCl_3$) of **3aq**

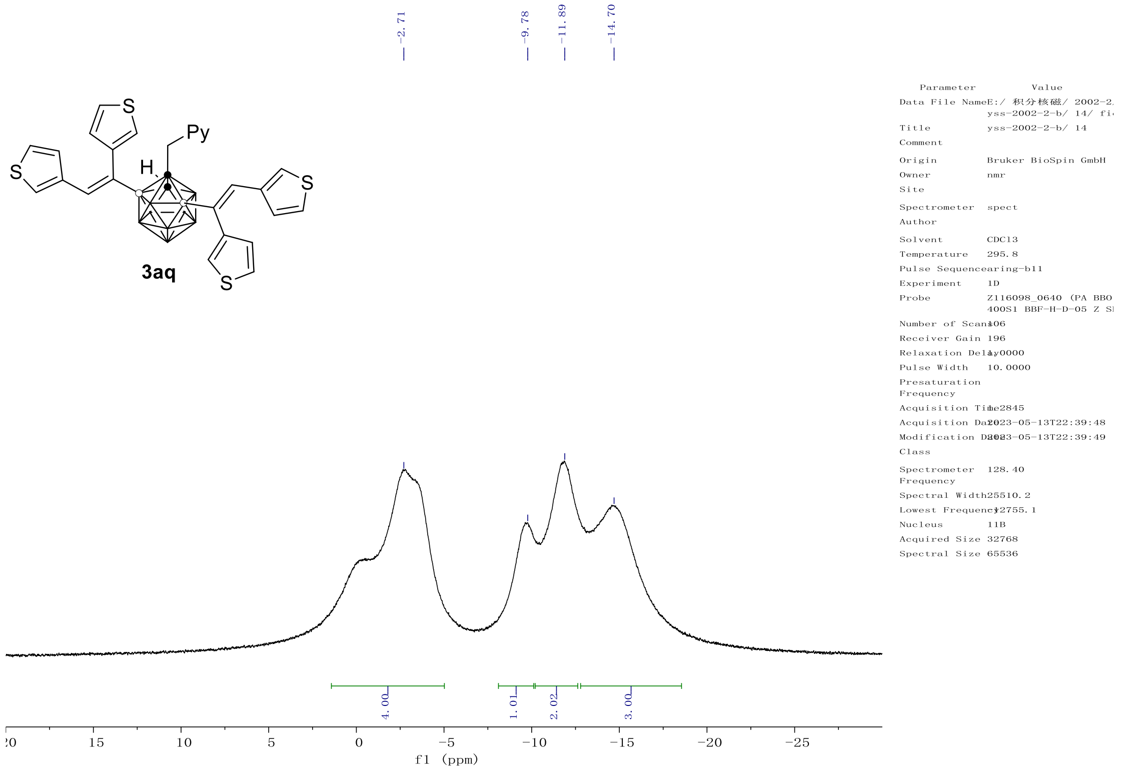

^{11}B NMR (128 MHz, $CDCl_3$) of **3aq**

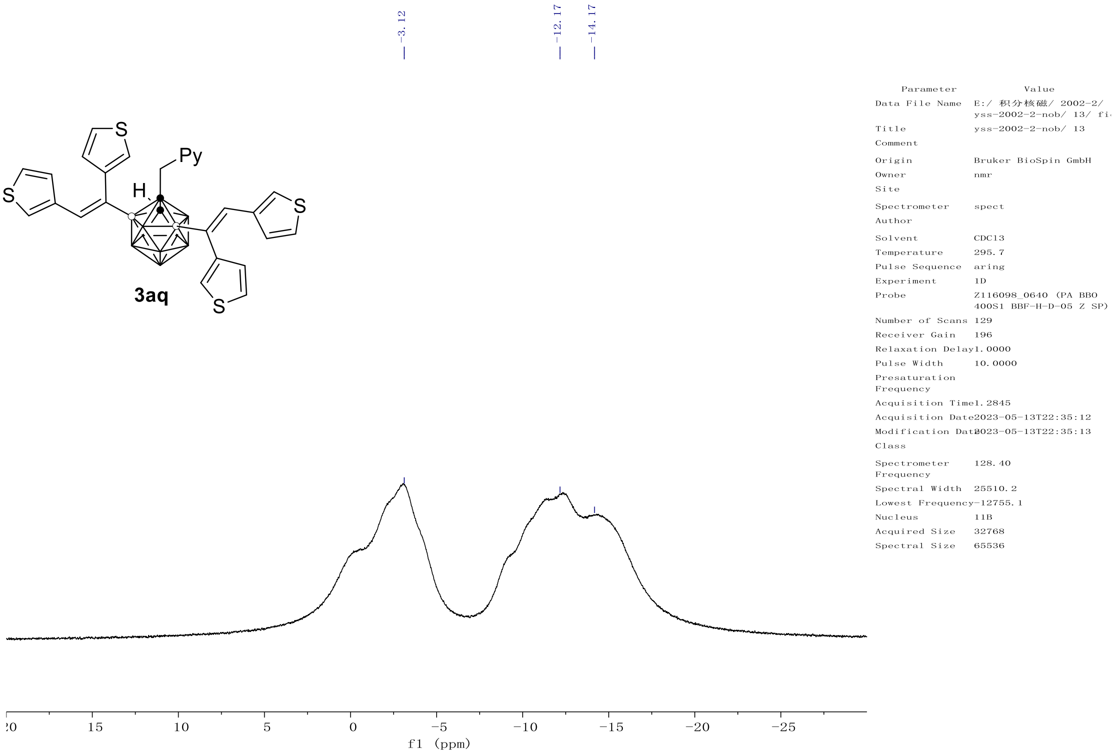

^{1}H NMR (400 MHz, $CDCl_3$) of **4ba**

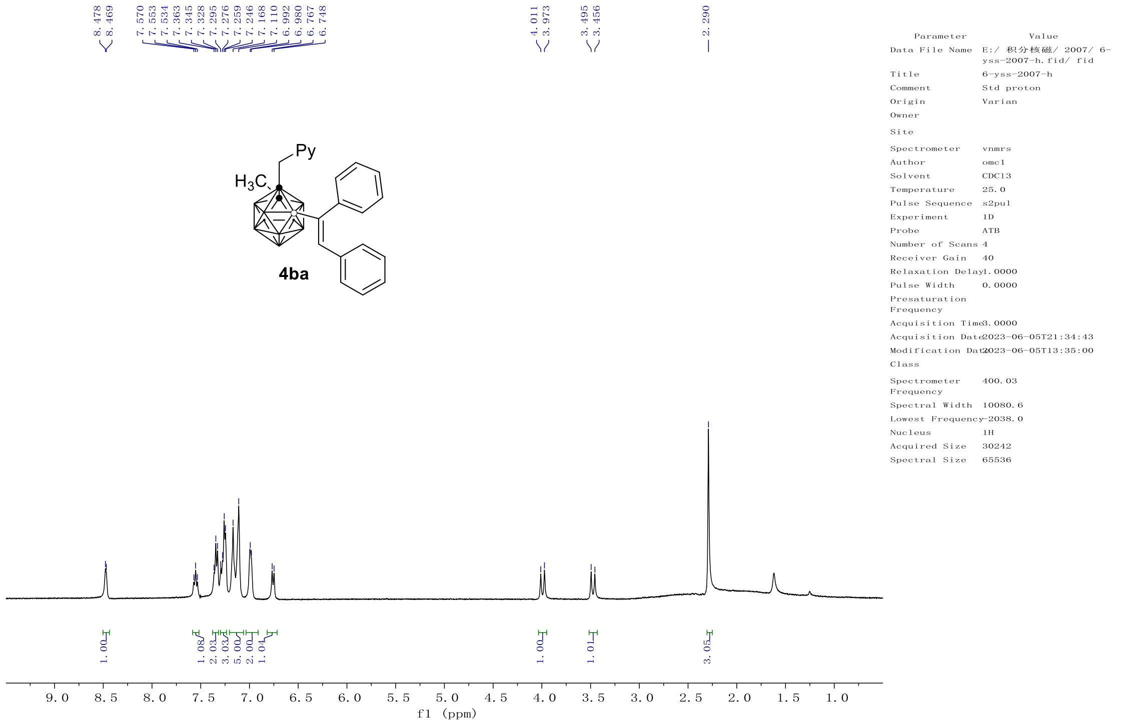

^{13}C{^{1}H} NMR (101 MHz, $CDCl_3$) of **4ba**

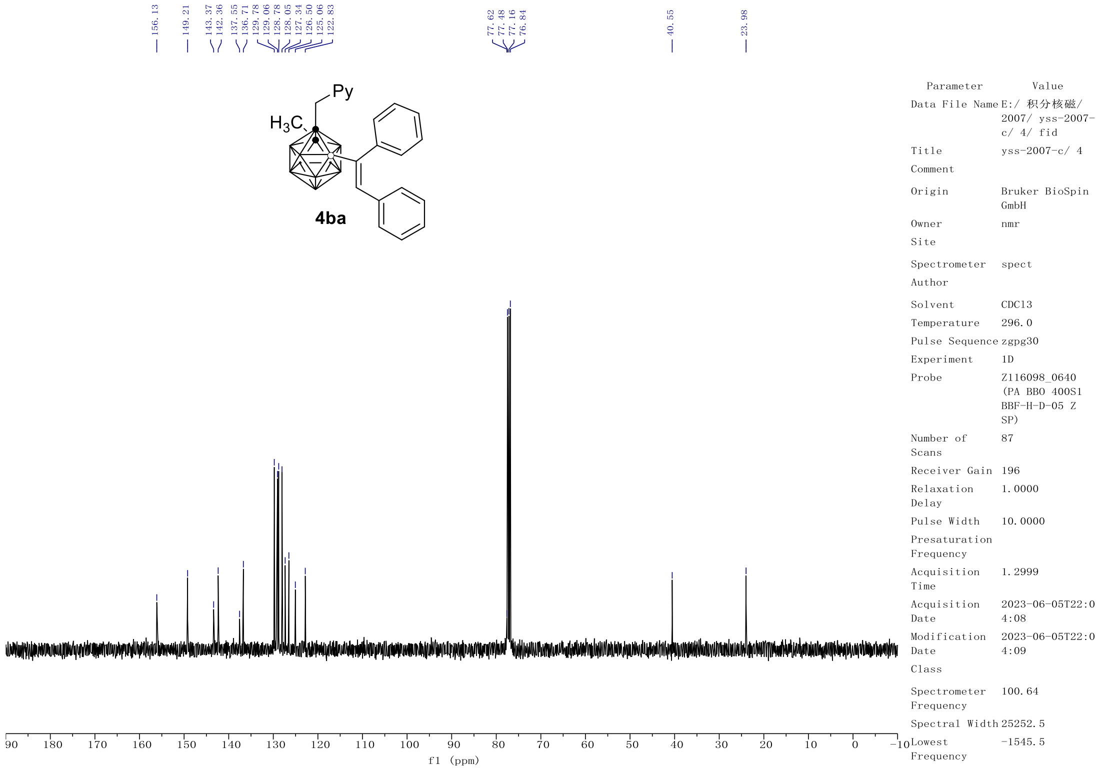

$^{11}B\{^{1}H\}$ NMR (128 MHz, $CDCl_3$) of **4ba**

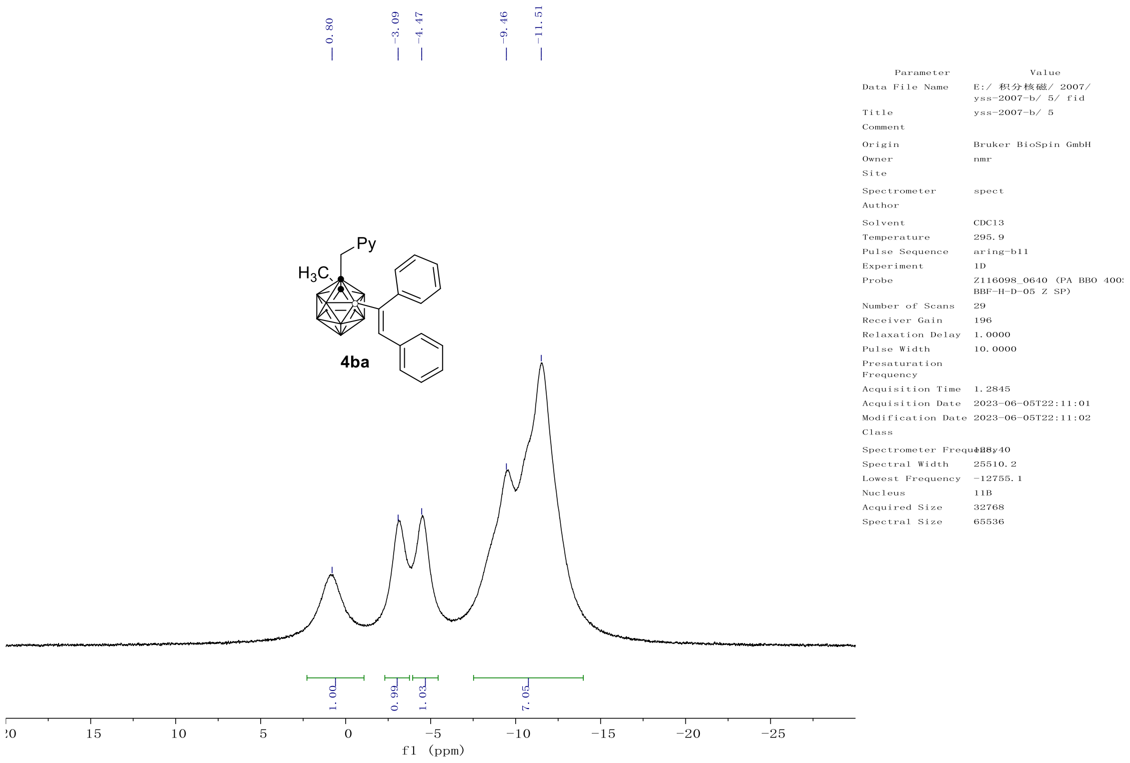

^{11}B NMR (128 MHz, $CDCl_3$) of **4ba**

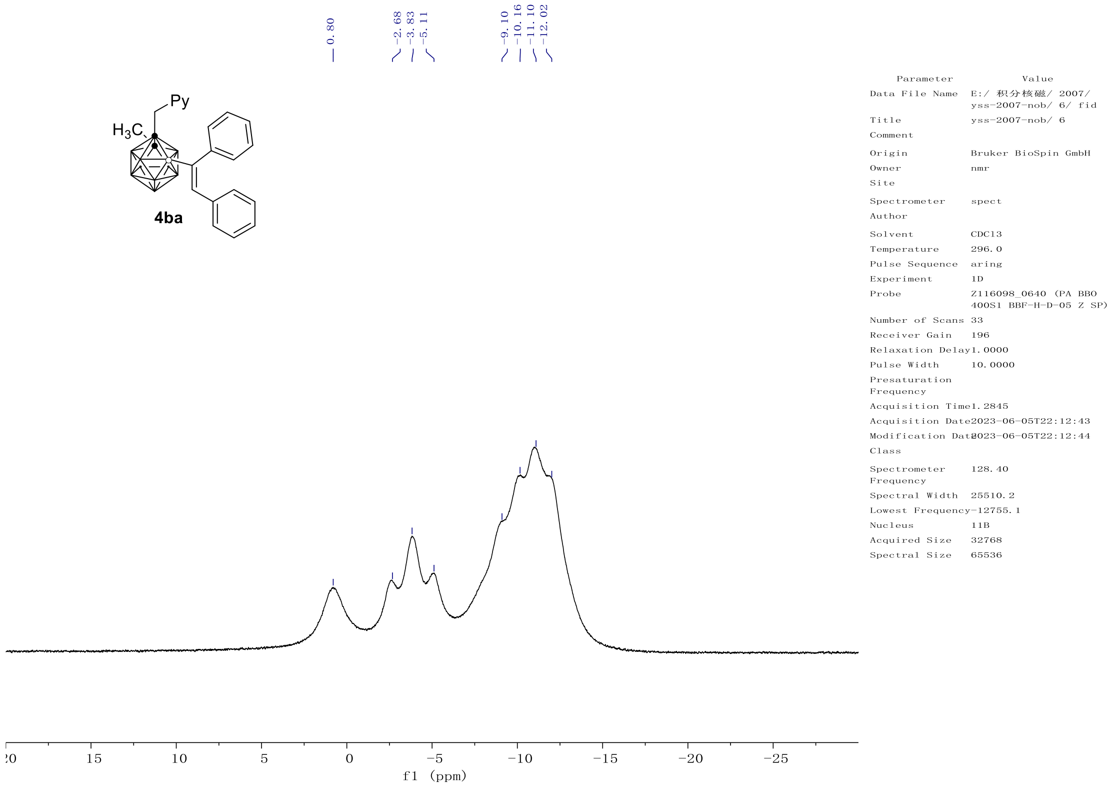

^{1}H NMR (400 MHz, $CDCl_3$) of **4bb**

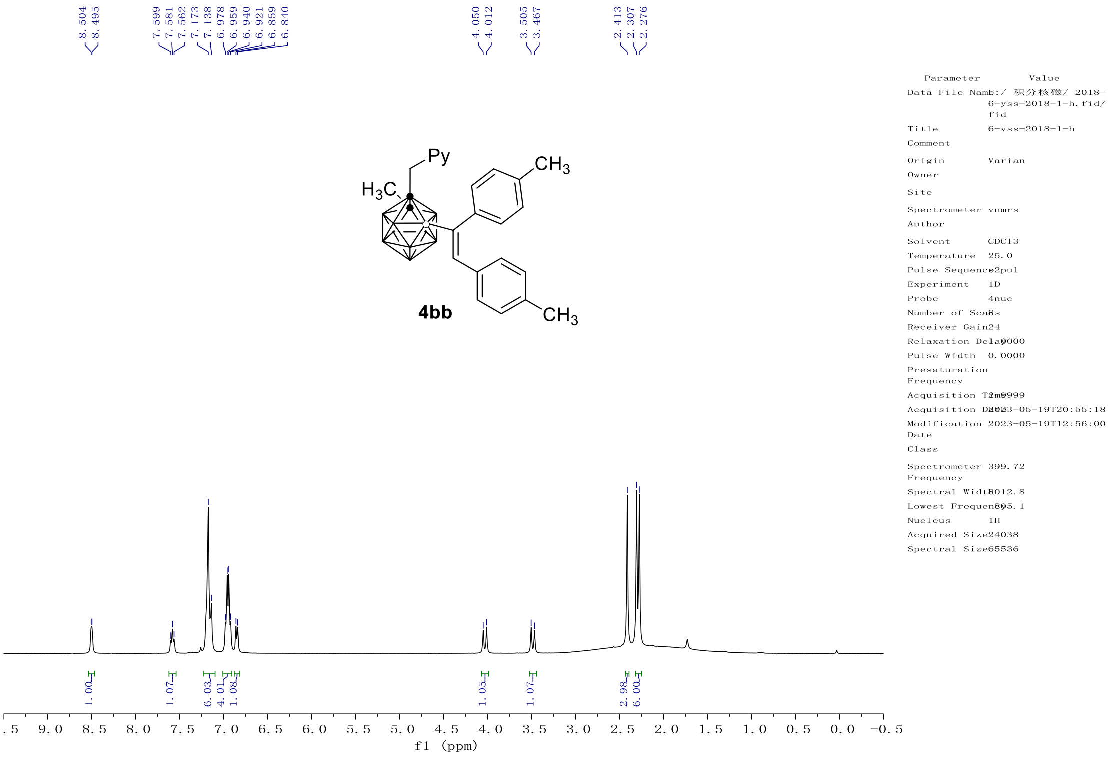

$^{13}C\{^{1}H\}$ NMR (101 MHz, $CDCl_3$) of **4bb**

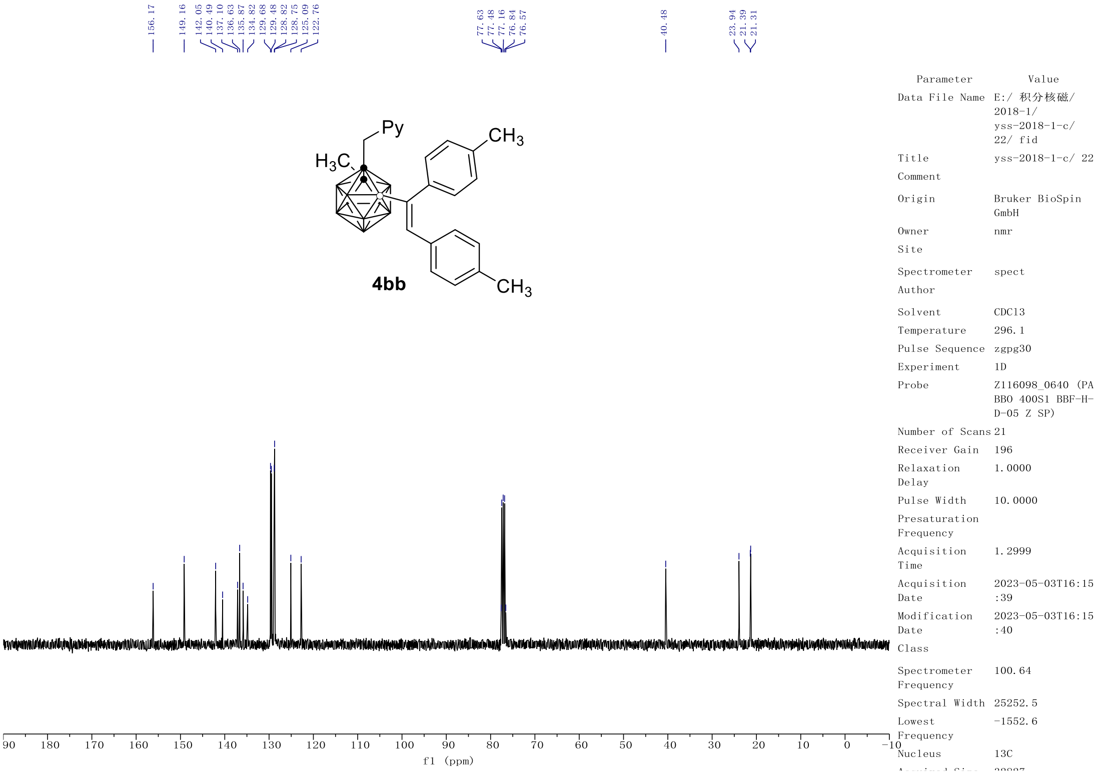

$^{11}B\{^{1}H\}$ NMR (128 MHz, $CDCl_3$) of **4bb**

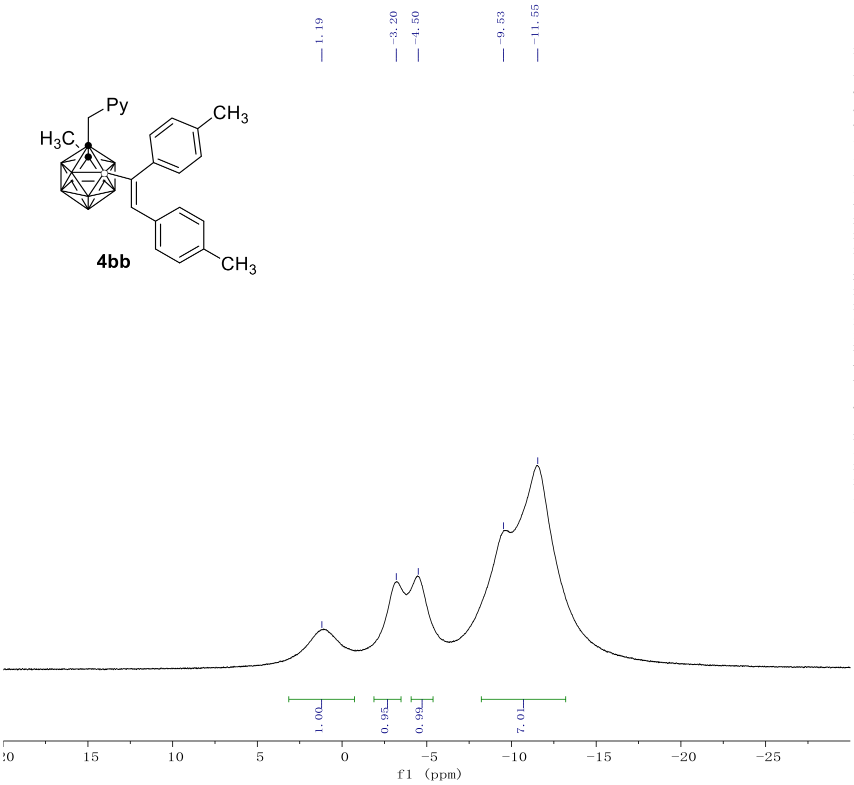

Parameter	Value
Data File Name	E:/ 积分核磁/ 2018-1/ yss-2018-1-b/ 23/ fid
Title	yss-2018-1-b/ 23
Comment	
Origin	Bruker BioSpin GmbH
Owner	nmr
Site	
Spectrometer	spect
Author	
Solvent	CDCl3
Temperature	296.1
Pulse Sequence	aring-b11
Experiment	1D
Probe	Z116098_0640 (PA BBO 40[illegible] BBF-H-D-05 Z SP)
Number of Scans	8
Receiver Gain	196
Relaxation Delay	1.0000
Pulse Width	10.0000
Presaturation Frequency	
Acquisition Time	1.2845
Acquisition Date	2023-05-03T16:18:37
Modification Date	2023-05-03T16:18:37
Class	
Spectrometer Frequency	128.40
Spectral Width	25510.2
Lowest Frequency	-12755.1
Nucleus	11B
Acquired Size	32768
Spectral Size	65536

^{11}B NMR (128 MHz, $CDCl_3$) of **4bb**

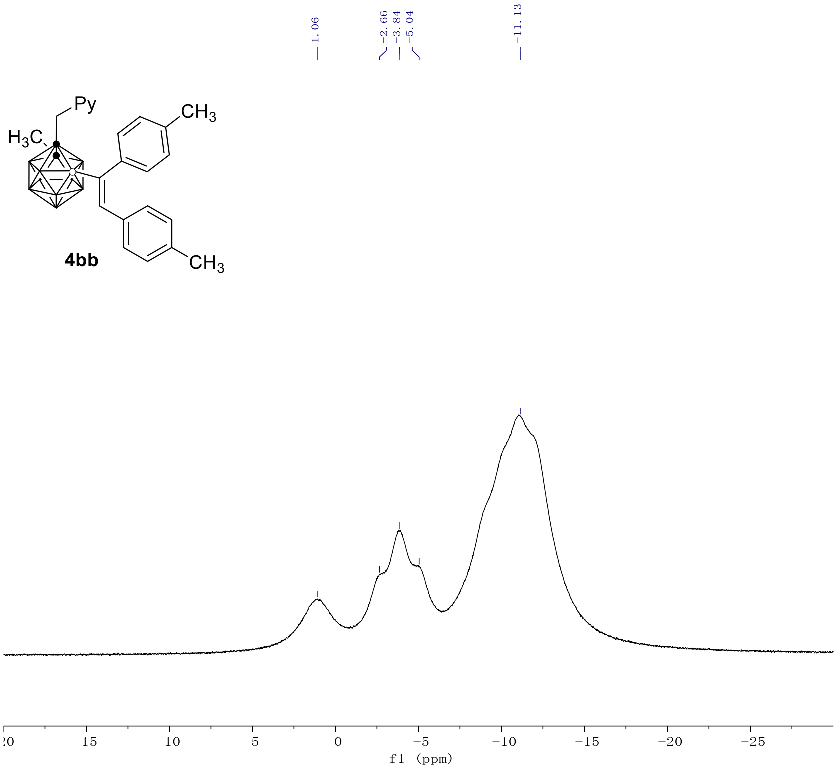

Parameter	Value
Data File Name	E:/ 积分核磁/ 2018-1/ yss-2018-1-nob/ 24/ fid
Title	yss-2018-1-nob/ 24
Comment	
Origin	Bruker BioSpin GmbH
Owner	nmr
Site	
Spectrometer	spect
Author	
Solvent	CDCl3
Temperature	296.0
Pulse Sequence	aring
Experiment	1D
Probe	Z116098_0640 (PA BBO 400[illegible] BBF-H-D-05 Z SP)
Number of Scans	8
Receiver Gain	196
Relaxation Delay	1.0000
Pulse Width	10.0000
Presaturation Frequency	
Acquisition Time	1.2845
Acquisition Date	2023-05-03T16:20:41
Modification Date	2023-05-03T16:20:42
Class	
Spectrometer Frequency	128.40
Spectral Width	25510.2
Lowest Frequency	-12755.1
Nucleus	11B
Acquired Size	32768
Spectral Size	65536

^{1}H NMR (400 MHz, $CDCl_3$) of **4bc**

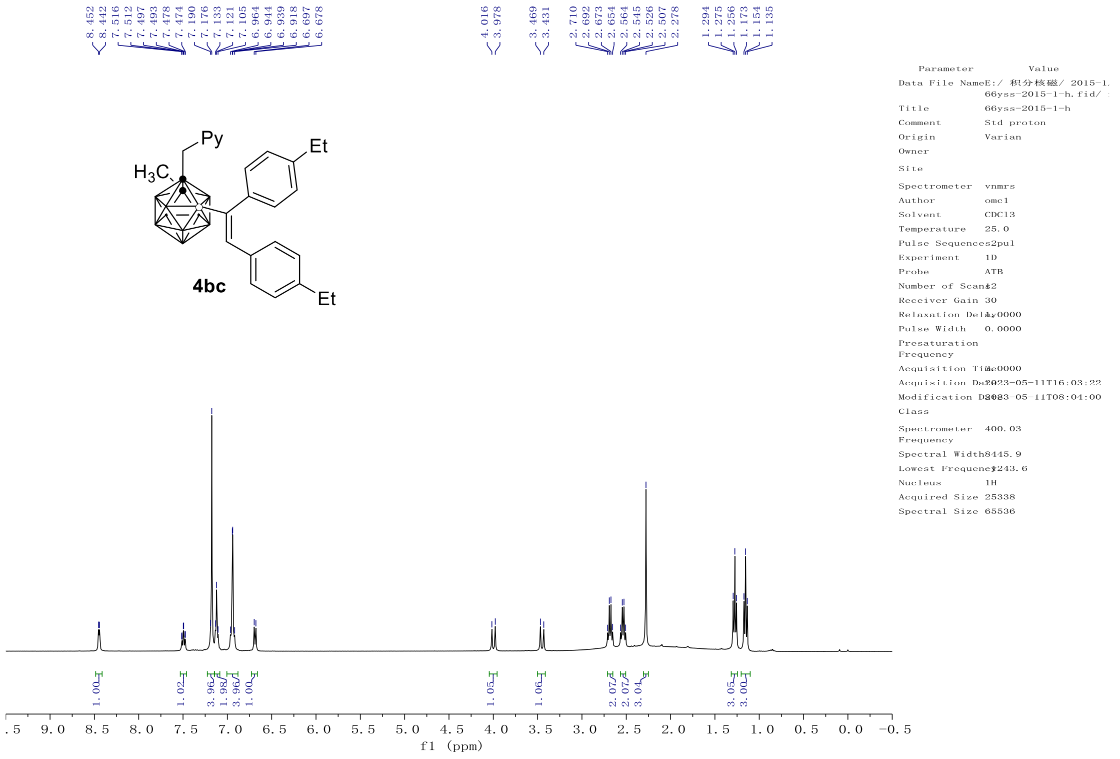

$^{13}C\{^{1}H\}$ NMR (101 MHz, $CDCl_3$) of **4bc**

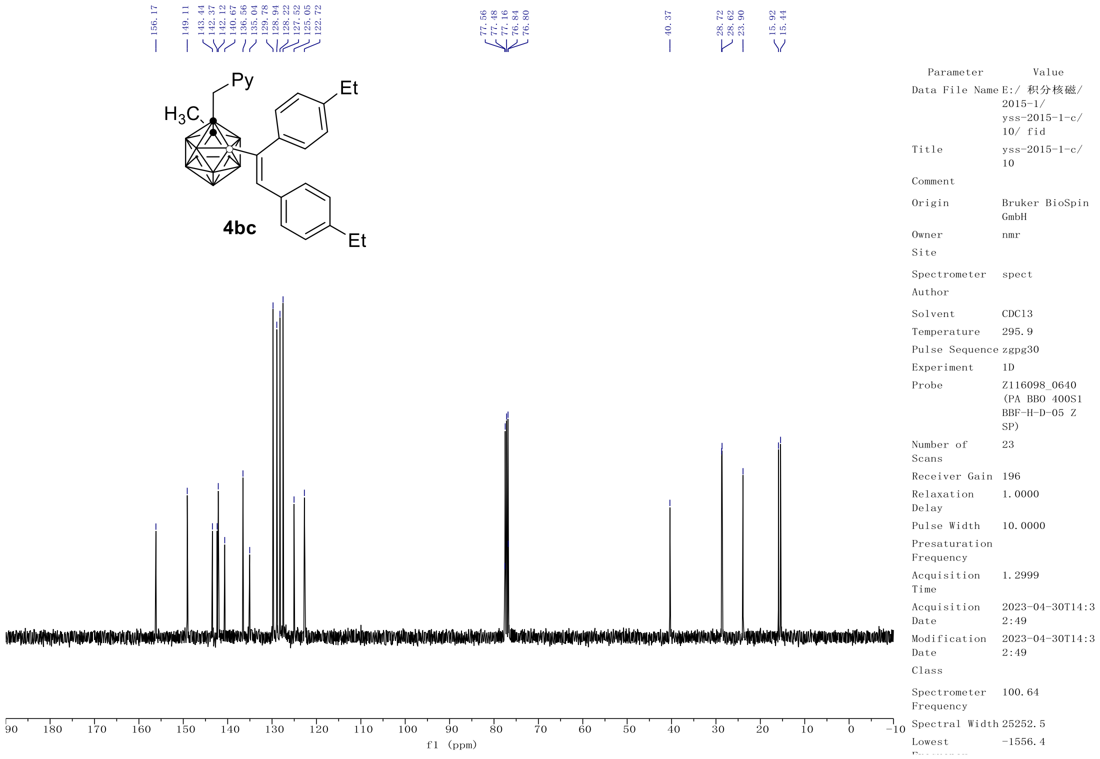

$^{11}B\{^{1}H\}$ NMR (128 MHz, $CDCl_3$) of **4bc**

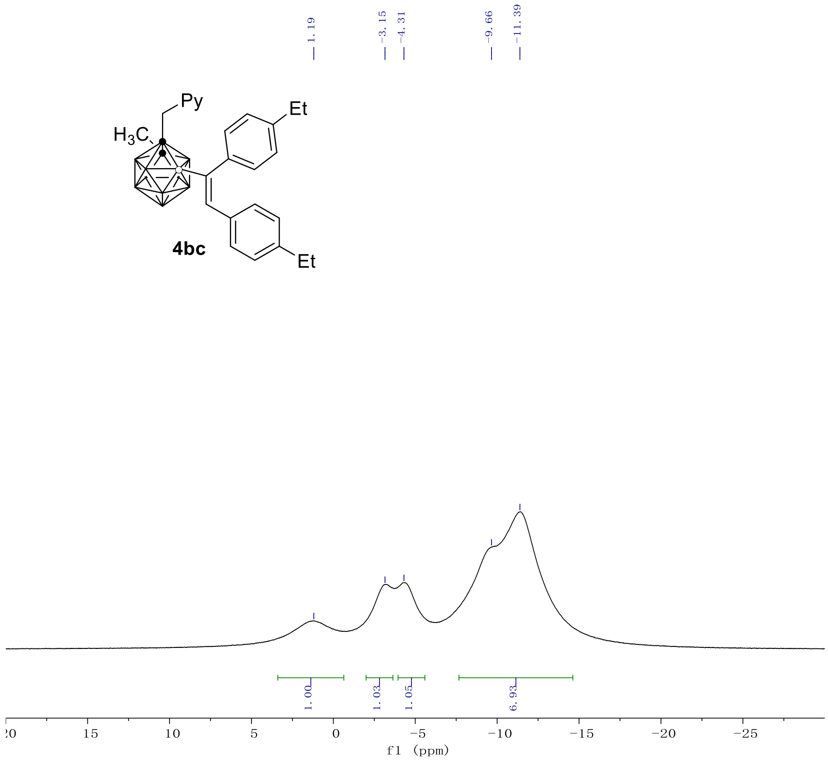

Parameter	Value
Data File Name	E:/ 积分核磁/ 2015-1/ yss-2015-1-b/ 11/ fid
Title	yss-2015-1-b/ 11
Comment	
Origin	Bruker BioSpin GmbH
Owner	nmr
Site	
Spectrometer	spect
Author	
Solvent	CDCl3
Temperature	295.9
Pulse Sequence	aring-b11
Experiment	1D
Probe	Z116098_0640 (PA BBO 400S1 I H-D-05 Z SP)
Number of Scans	12
Receiver Gain	196
Relaxation Delay	1.0000
Pulse Width	10.0000
Presaturation Frequency	
Acquisition Time	1.2845
Acquisition Date	2023-04-30T14:35:56
Modification Date	2023-04-30T14:35:57
Class	
Spectrometer Frequency	128.40
Spectral Width	25510.2
Lowest Frequency	-12755.1
Nucleus	11B
Acquired Size	32768
Spectral Size	65536

^{11}B NMR (128 MHz, $CDCl_3$) of **4bc**

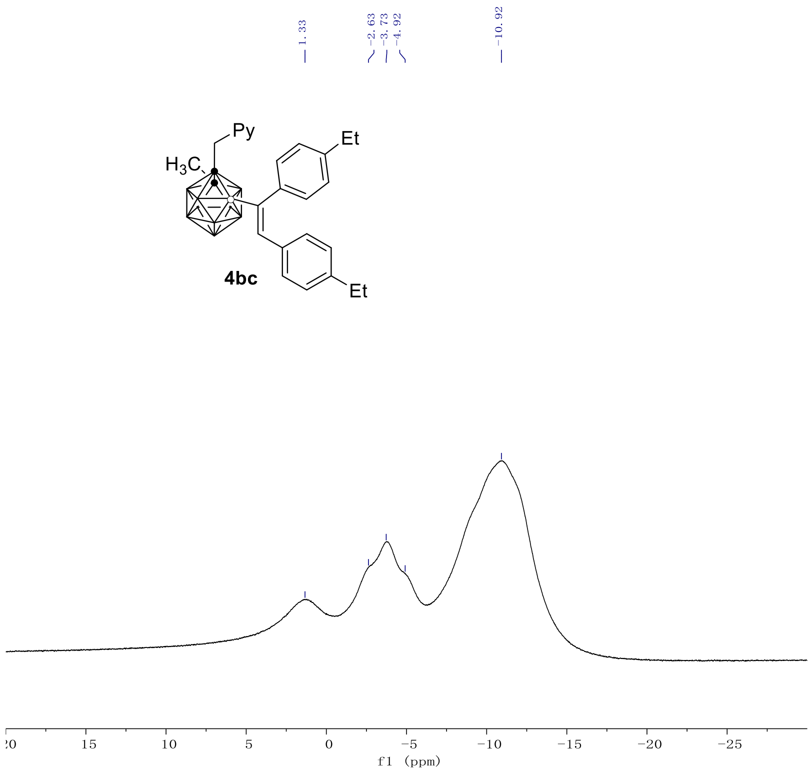

Parameter	Value
Data File Name	E:/ 积分核磁/ 2015-1/ yss-2015-1-nob/ 3/ fid
Title	yss-2015-1-nob/ 3
Comment	
Origin	Bruker BioSpin GmbH
Owner	nmr
Site	
Spectrometer	spect
Author	
Solvent	C6D6
Temperature	295.7
Pulse Sequence	aring
Experiment	1D
Probe	Z116098_0640 (PA BBO 40(BBF-H-D-05 Z SP)
Number of Scans	8
Receiver Gain	196
Relaxation Delay	1.0000
Pulse Width	10.0000
Presaturation Frequency	
Acquisition Time	1.2845
Acquisition Date	2023-04-30T15:23:52
Modification Date	2023-04-30T15:23:53
Class	
Spectrometer Frequency	128.40
Spectral Width	25510.2
Lowest Frequency	-12755.1
Nucleus	11B
Acquired Size	32768
Spectral Size	65536

^{1}H NMR (400 MHz, $CDCl_3$) of **4bd**

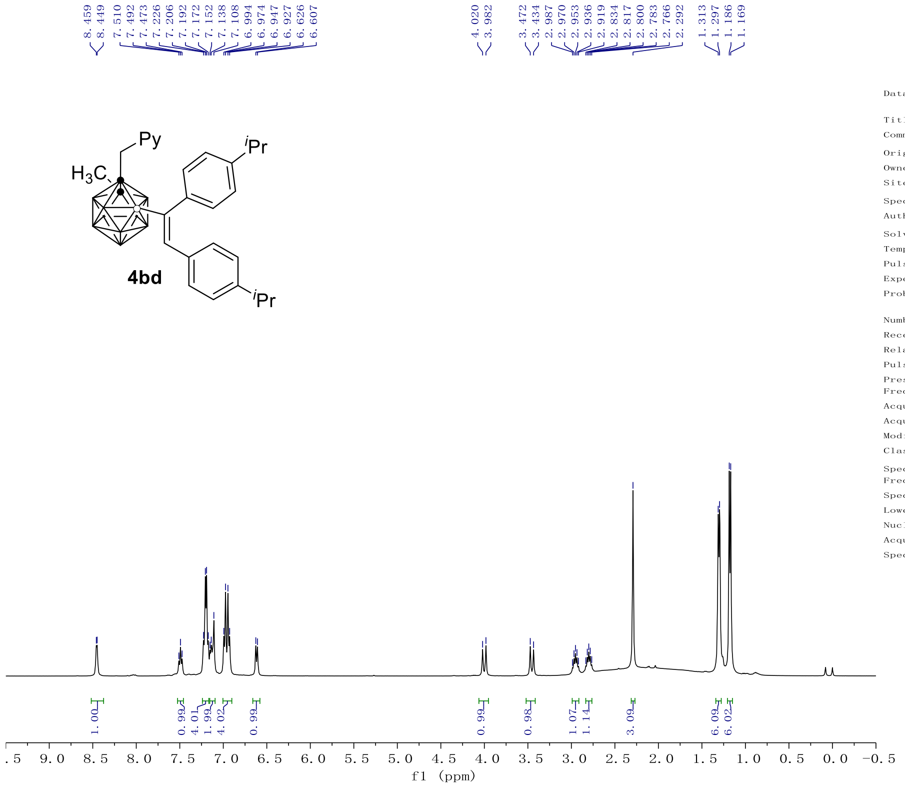

Parameter	Value
Data File Name	E:/ 积分核磁/ 2014-2/ yss-2014-2-h/ 1/ fid
Title	yss-2014-2-h.1.fid
Comment	
Origin	Bruker BioSpin GmbH
Owner	nmr
Site	
Spectrometer	spect
Author	
Solvent	CDCl3
Temperature	298.2
Pulse Sequence	zg30
Experiment	1D
Probe	Z116098_0643 (PA BBO 400S1 BBF-H-D-05 Z SP
Number of Scans	5
Receiver Gain	25
Relaxation Delay	1.0000
Pulse Width	10.0000
Presaturation Frequency	
Acquisition Time	2.9998
Acquisition Date	2023-03-03T19:16:55
Modification Date	2023-03-03T19:16:55
Class	
Spectrometer Frequency	400.10
Spectral Width	7598.8
Lowest Frequency	-1250.0
Nucleus	1H
Acquired Size	22795
Spectral Size	65536

$^{13}C\{^{1}H\}$ NMR (101 MHz, $CDCl_3$) of **4bd**

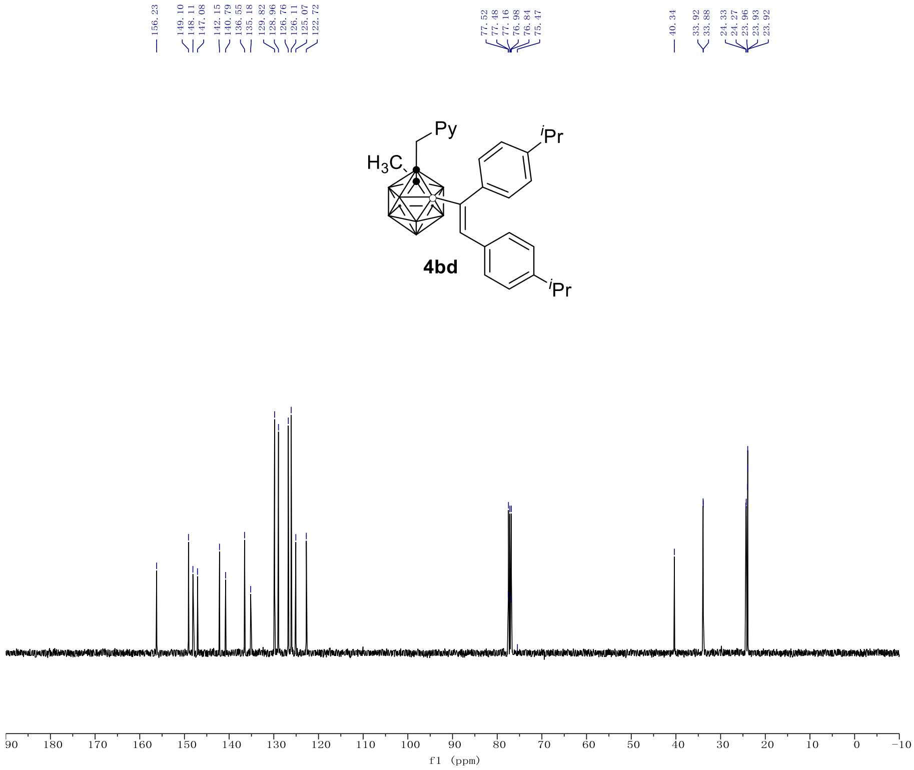

Parameter	Value
Data File Name	E:/ 积分核磁/ 2014-2/ yss-2014-2-c/ 1/ fid
Title	yss-2014-2-c.1.fid
Comment	
Origin	Bruker BioSpin GmbH
Owner	nmr
Site	
Spectrometer	spect
Author	
Solvent	CDCl3
Temperature	298.1
Pulse Sequence	zgpg30
Experiment	1D
Probe	Z116098_0643 (PA BBO 400S1 BBF-H-D-05 Z SP)
Number of Scans	50
Receiver Gain	197
Relaxation Delay	1.0000
Pulse Width	10.0000
Presaturation Frequency	
Acquisition Time	1.2999
Acquisition Date	2023-04-30T14:02:47
Modification Date	2023-04-30T14:02:47
Class	
Spectrometer Frequency	100.62
Spectral Width	25252.5
Lowest Frequency	-1554.7

$^{11}B\{^1H\}$ NMR (128 MHz, $CDCl_3$) of **4bd**

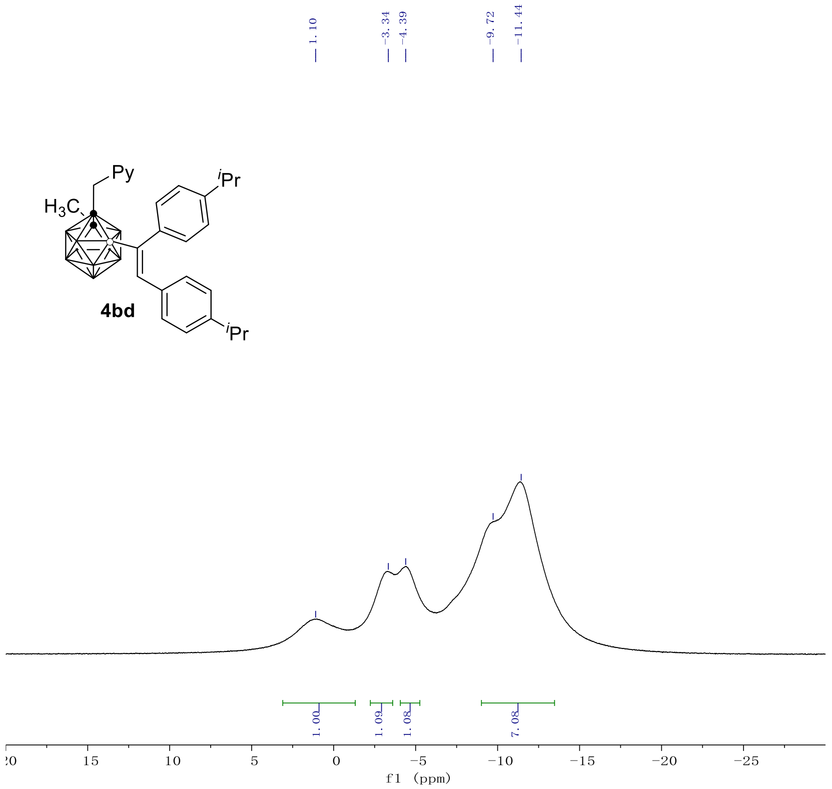

Parameter	Value
Data File Name	E:/ 积分核磁/ 2014-2/ yss-2014-2-b-3/ 6/ fid
Title	yss-2014-2-b-3/ 6
Comment	
Origin	Bruker BioSpin GmbH
Owner	nmr
Site	
Spectrometer	spect
Author	
Solvent	CDCl3
Temperature	295.9
Pulse Sequence	aring-b11
Experiment	1D
Probe	Z116098_0640 (PA BBO 400S BBF-H-D-05 Z SP)
Number of Scans	13
Receiver Gain	196
Relaxation Delay	1.0000
Pulse Width	10.0000
Presaturation Frequency	
Acquisition Time	1.2845
Acquisition Date	2023-04-30T14:14:08
Modification Date	2023-04-30T14:14:09
Class	
Spectrometer Frequency	128.40
Spectral Width	25510.2
Lowest Frequency	-12755.1
Nucleus	11B
Acquired Size	32768
Spectral Size	65536

^{11}B NMR (128 MHz, $CDCl_3$) of **4bd**

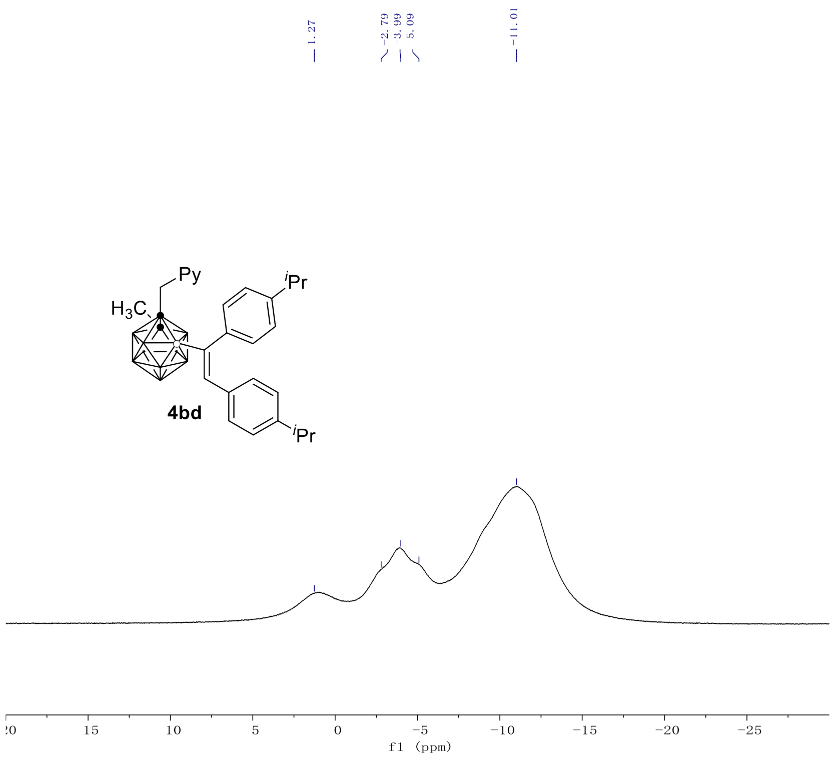

Parameter	Value
Data File Name	E:/ 积分核磁/ 2014-2/ yss-2014-2-nob/ 2/ fid
Title	yss-2014-2-nob/ 2
Comment	
Origin	Bruker BioSpin GmbH
Owner	nmr
Site	
Spectrometer	spect
Author	
Solvent	C6D6
Temperature	295.7
Pulse Sequence	aring
Experiment	1D
Probe	Z116098_0640 (PA BBO 400S1 BBF-H-D-05 Z SP)
Number of Scans	10
Receiver Gain	196
Relaxation Delay	1.0000
Pulse Width	10.0000
Presaturation Frequency	
Acquisition Time	1.2845
Acquisition Date	2023-04-30T15:21:21
Modification Date	2023-04-30T15:21:21
Class	
Spectrometer Frequency	128.40
Spectral Width	25510.2
Lowest Frequency	-12755.1
Nucleus	11B
Acquired Size	32768
Spectral Size	65536

^{1}H NMR (400 MHz, $CDCl_3$) of **4be**

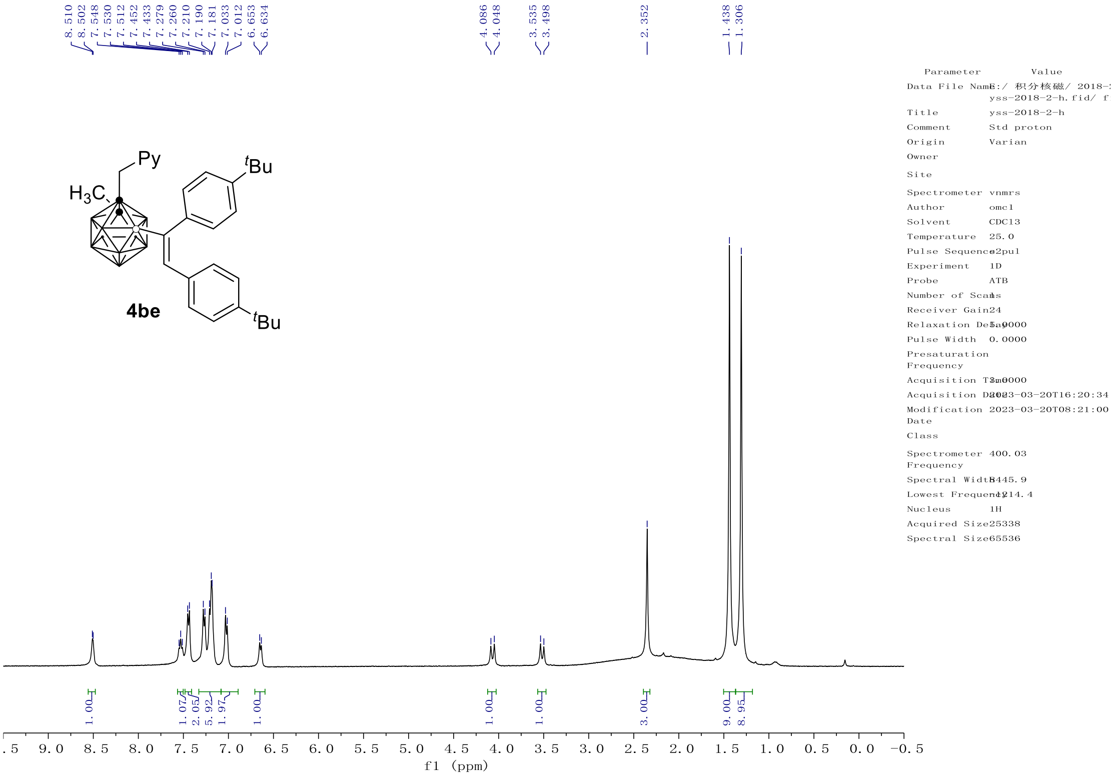

$^{13}C\{^{1}H\}$ NMR (101 MHz, $CDCl_3$) of **4be**

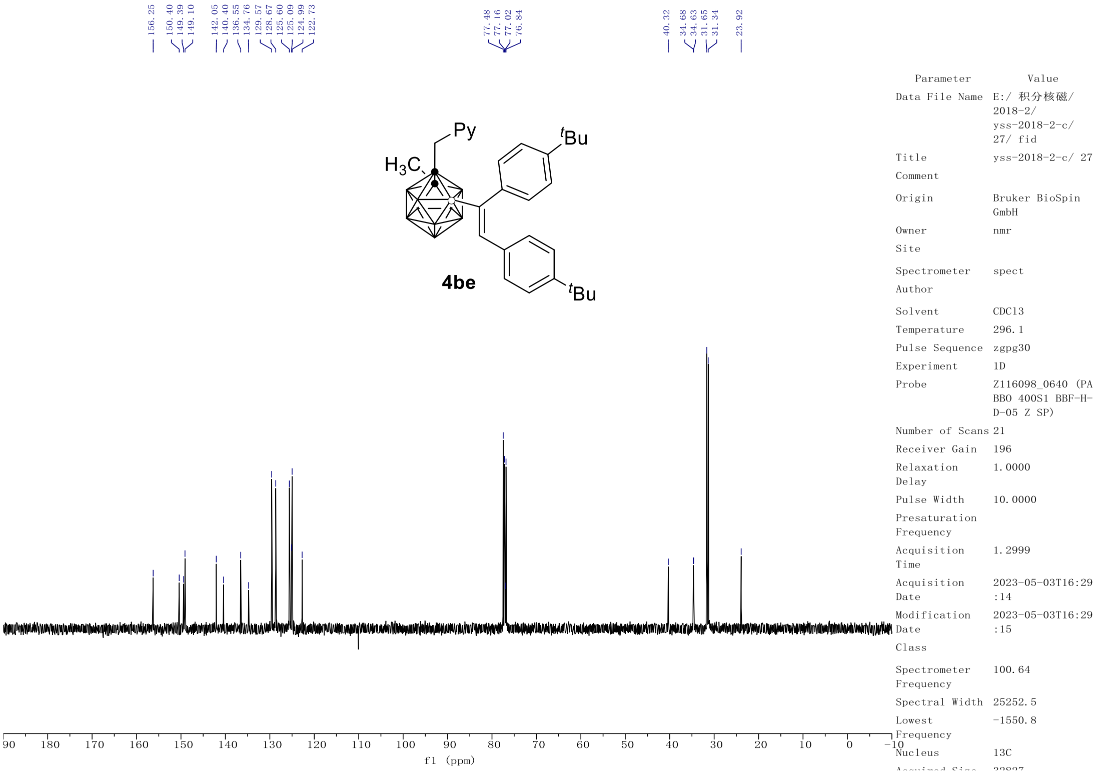

$^{11}B\{^{1}H\}$ NMR (128 MHz, $CDCl_3$) of **4be**

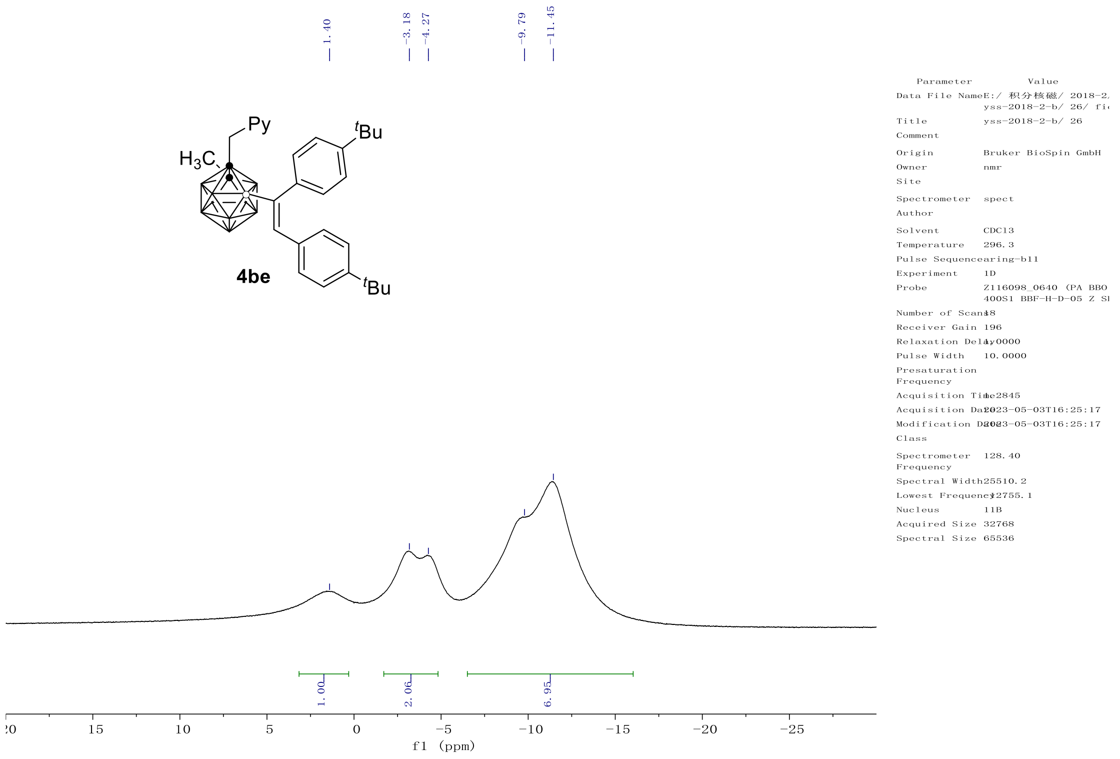

^{11}B NMR (128 MHz, $CDCl_3$) of **4be**

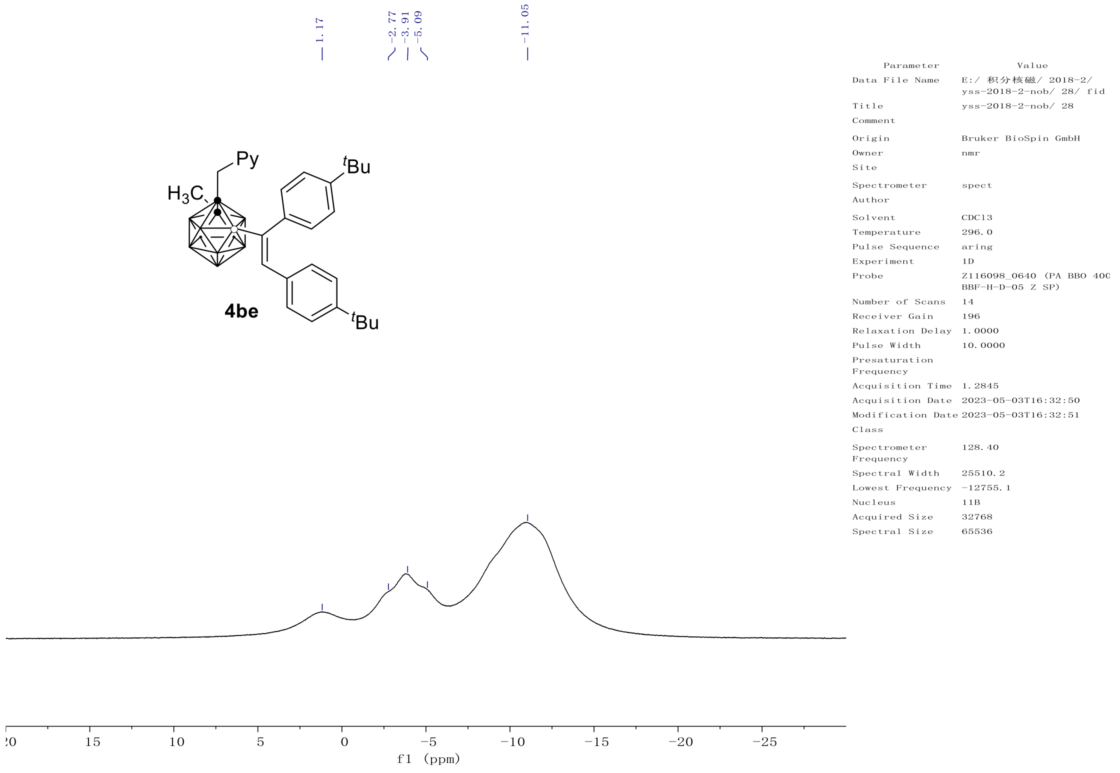

^1H NMR (400 MHz, $CDCl_3$) of **4bf**

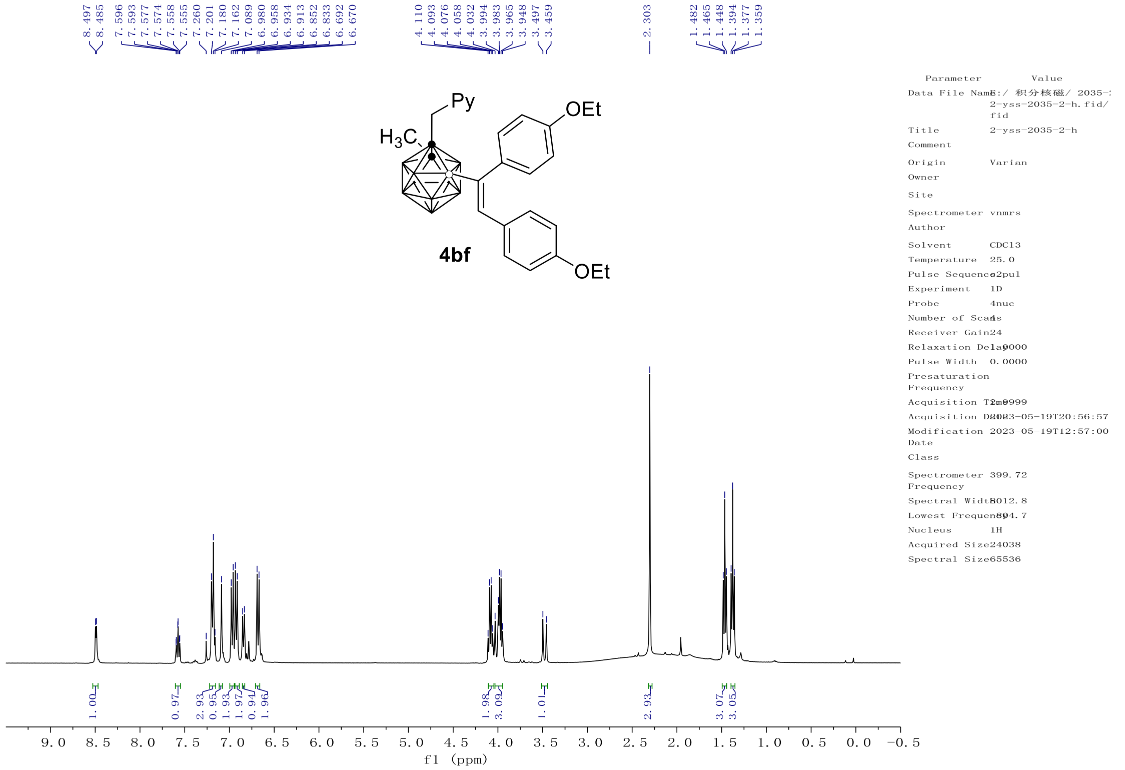

$^{13}C\{^1H\}$ NMR (101 MHz, $CDCl_3$) of **4bf**

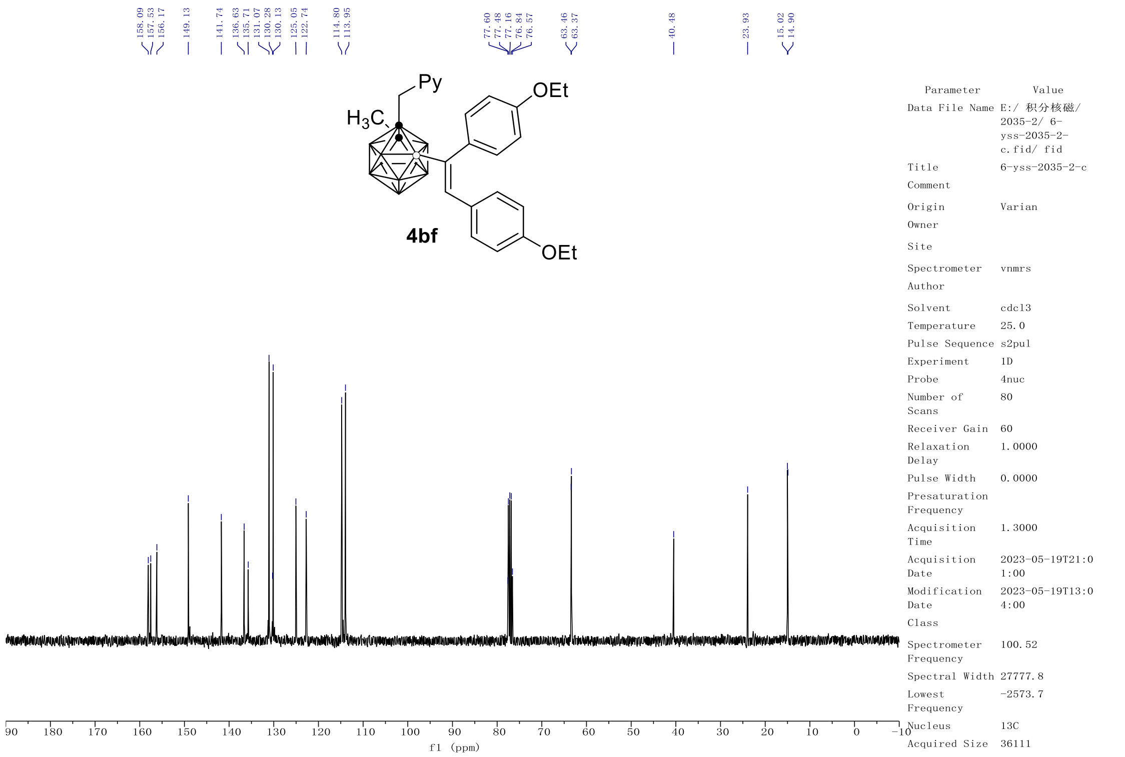

$^{11}B\{^{1}H\}$ NMR (128 MHz, $CDCl_3$) of **4bf**

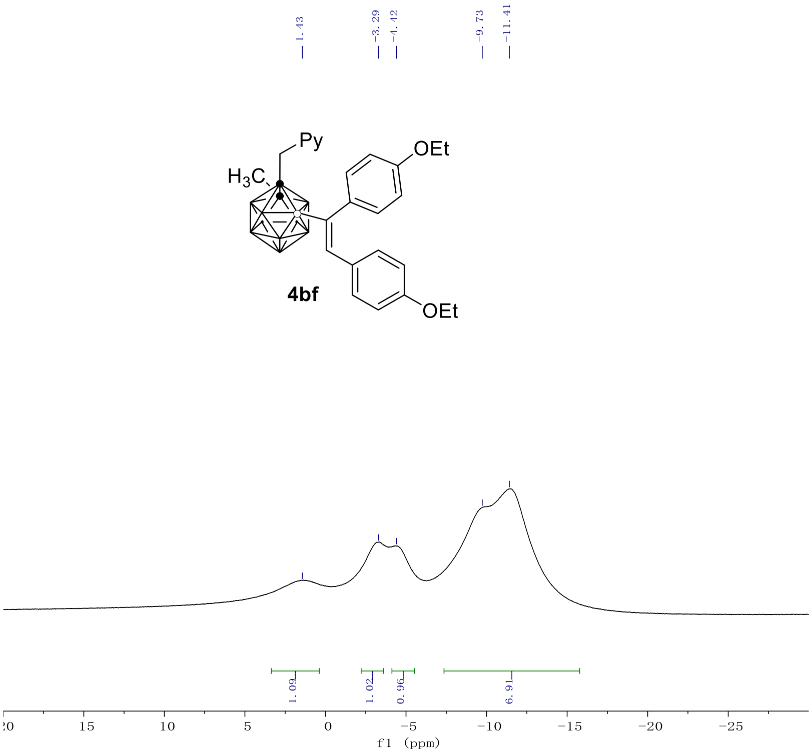

Parameter	Value
Data File Name	E:/ 积分核磁/ 2035-2/ yss-203b/ 8/ fid
Title	yss-2035-2-b/ 8
Comment	
Origin	Bruker BioSpin GmbH
Owner	nmr
Site	
Spectrometer	spect
Author	
Solvent	CDCl3
Temperature	296.0
Pulse Sequence	aring-b11
Experiment	1D
Probe	Z116098_0640 (PA BBO 400S1 BBD-05 Z SP)
Number of Scans	24
Receiver Gain	196
Relaxation Delay	1.0000
Pulse Width	10.0000
Presaturation Frequency	
Acquisition Time	1.2845
Acquisition Date	2023-05-19T21:04:06
Modification Date	2023-05-19T21:04:07
Class	
Spectrometer Frequency	128.40
Spectral Width	25510.2
Lowest Frequency	-12755.1
Nucleus	11B
Acquired Size	32768
Spectral Size	65536

^{11}B NMR (128 MHz, $CDCl_3$) of **4bf**

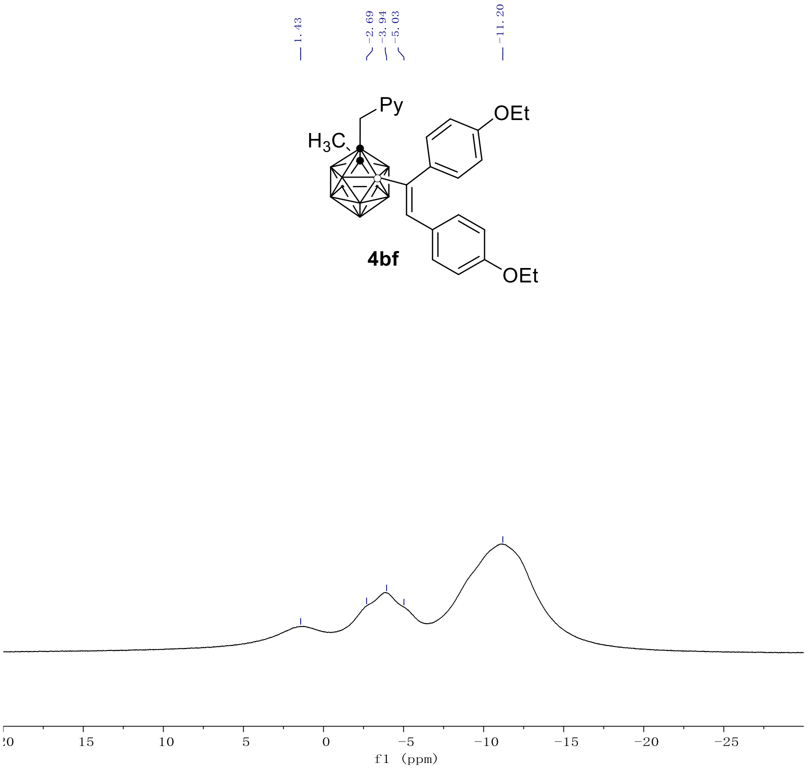

Parameter	Value
Data File Name	E:/ 积分核磁/ 2035-2/ yss-203nob/ 9/ fid
Title	yss-2035-2-nob/ 9
Comment	
Origin	Bruker BioSpin GmbH
Owner	nmr
Site	
Spectrometer	spect
Author	
Solvent	CDCl3
Temperature	296.0
Pulse Sequence	aring
Experiment	1D
Probe	Z116098_0640 (PA BBO 400S1 BBD-05 Z SP)
Number of Scans	16
Receiver Gain	196
Relaxation Delay	1.0000
Pulse Width	10.0000
Presaturation Frequency	
Acquisition Time	1.2845
Acquisition Date	2023-05-19T21:05:06
Modification Date	2023-05-19T21:05:06
Class	
Spectrometer Frequency	128.40
Spectral Width	25510.2
Lowest Frequency	-12755.1
Nucleus	11B
Acquired Size	32768
Spectral Size	65536

^{1}H NMR (400 MHz, $CDCl_3$) of **4bg**

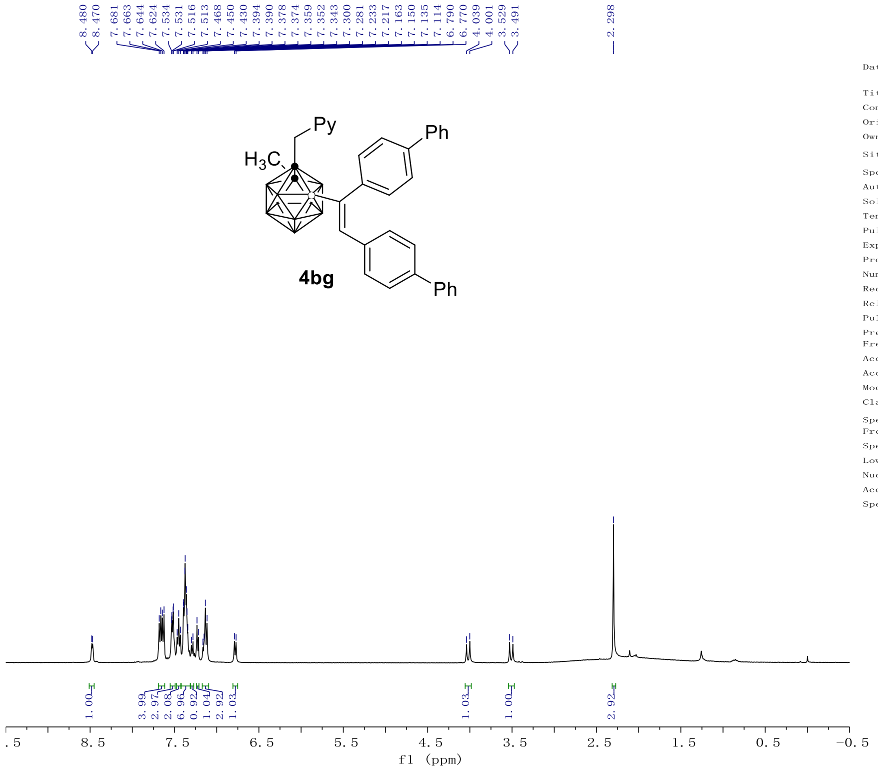

Parameter	Value
Data File Name	E:/ 积分核磁/ 2052-4/ 66yss-2052-4-h.fid/ fid
Title	66yss-2052-4-h
Comment	Std proton
Origin	Varian
Owner	
Site	
Spectrometer	vnmrs
Author	omc1
Solvent	CDCl3
Temperature	25.0
Pulse Sequence	s2pul
Experiment	1D
Probe	ATB
Number of Scans	4
Receiver Gain	32
Relaxation Delay	1.0000
Pulse Width	0.0000
Presaturation Frequency	
Acquisition Time	2.9999
Acquisition Date	2023-07-17T18:30:54
Modification Date	2023-07-17T10:31:00
Class	
Spectrometer Frequency	400.03
Spectral Width	8012.8
Lowest Frequency	-816.6
Nucleus	1H
Acquired Size	24038
Spectral Size	65536

$^{13}C\{^{1}H\}$ NMR (101 MHz, $CDCl_3$) of **4bg**

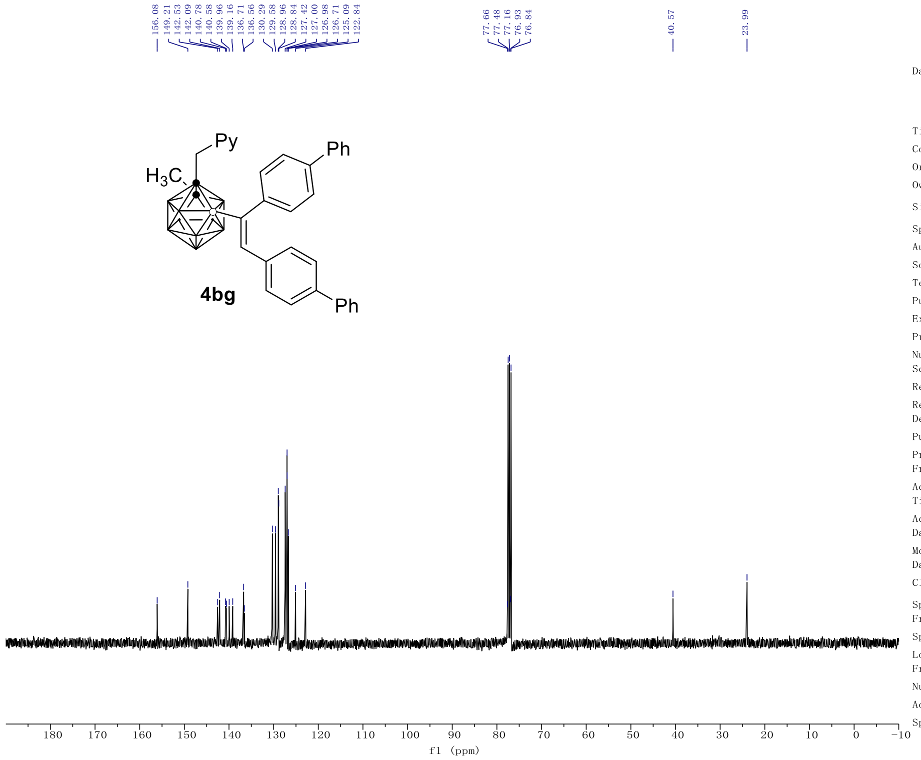

Parameter	Value
Data File Name	E:/ 积分核磁/ 2052-4/ yss-2052-4c.fid / fid
Title	yss-2052-4c
Comment	Std carbon
Origin	Varian
Owner	
Site	
Spectrometer	vnmrs
Author	omc1
Solvent	cdcl3
Temperature	25.0
Pulse Sequence	s2pul
Experiment	1D
Probe	ATB
Number of Scans	188
Receiver Gain	60
Relaxation Delay	1.0000
Pulse Width	0.0000
Presaturation Frequency	
Acquisition Time	1.3000
Acquisition Date	2023-07-17T18:32:21
Modification Date	2023-07-17T10:39:00
Class	
Spectrometer Frequency	100.60
Spectral Width	28409.1
Lowest Frequency	-2122.0
Nucleus	13C
Acquired Size	36932
Spectral Size	131072

$^{11}B\{^{1}H\}$ NMR (128 MHz, $CDCl_3$) of **4bg**

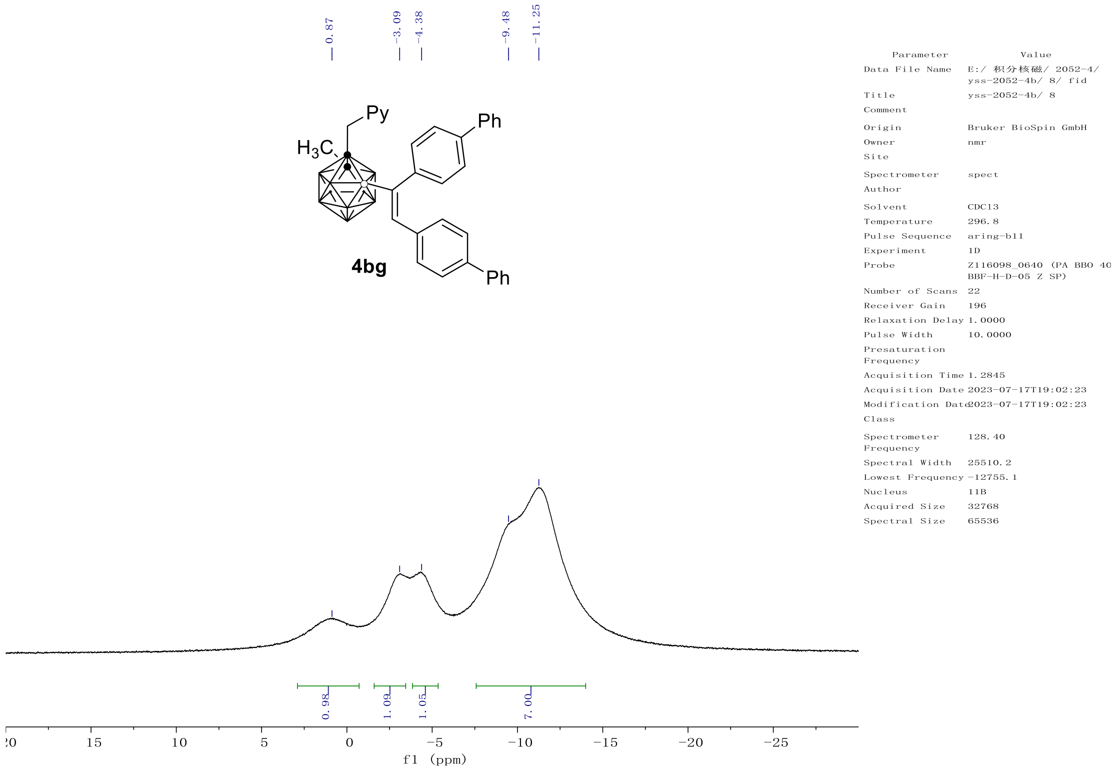

^{11}B NMR (128 MHz, $CDCl_3$) of **4bg**

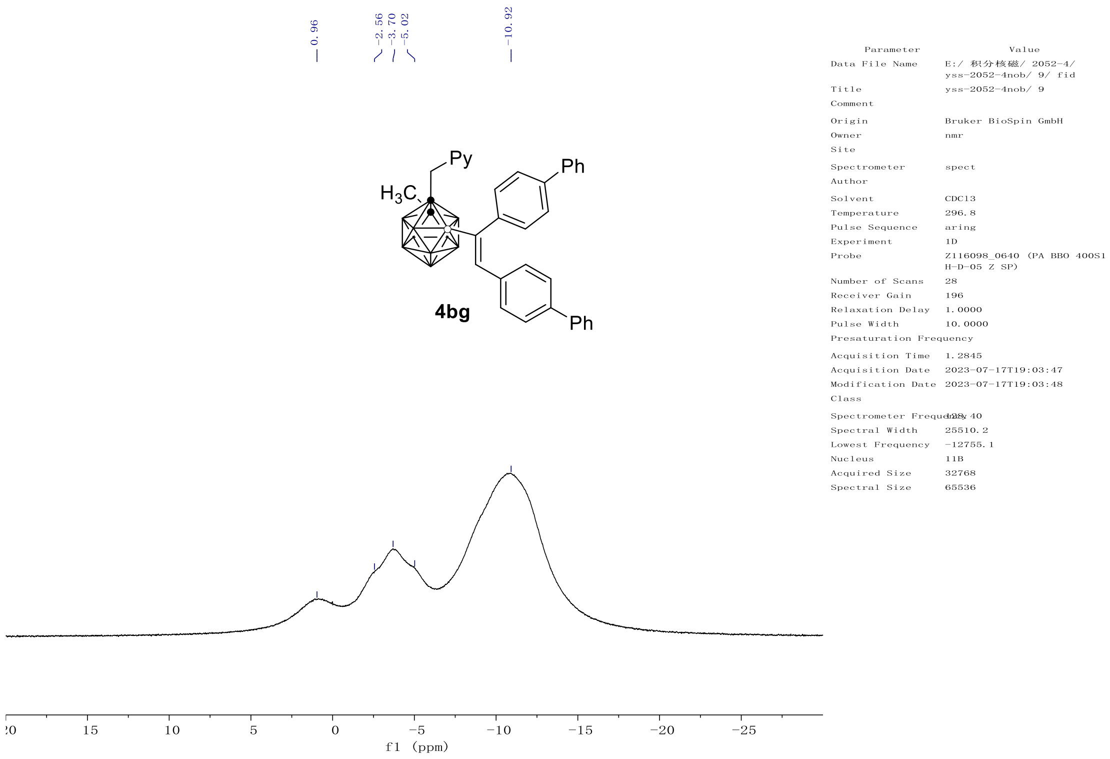

^{1}H NMR (400 MHz, $CDCl_3$) of **4bh**

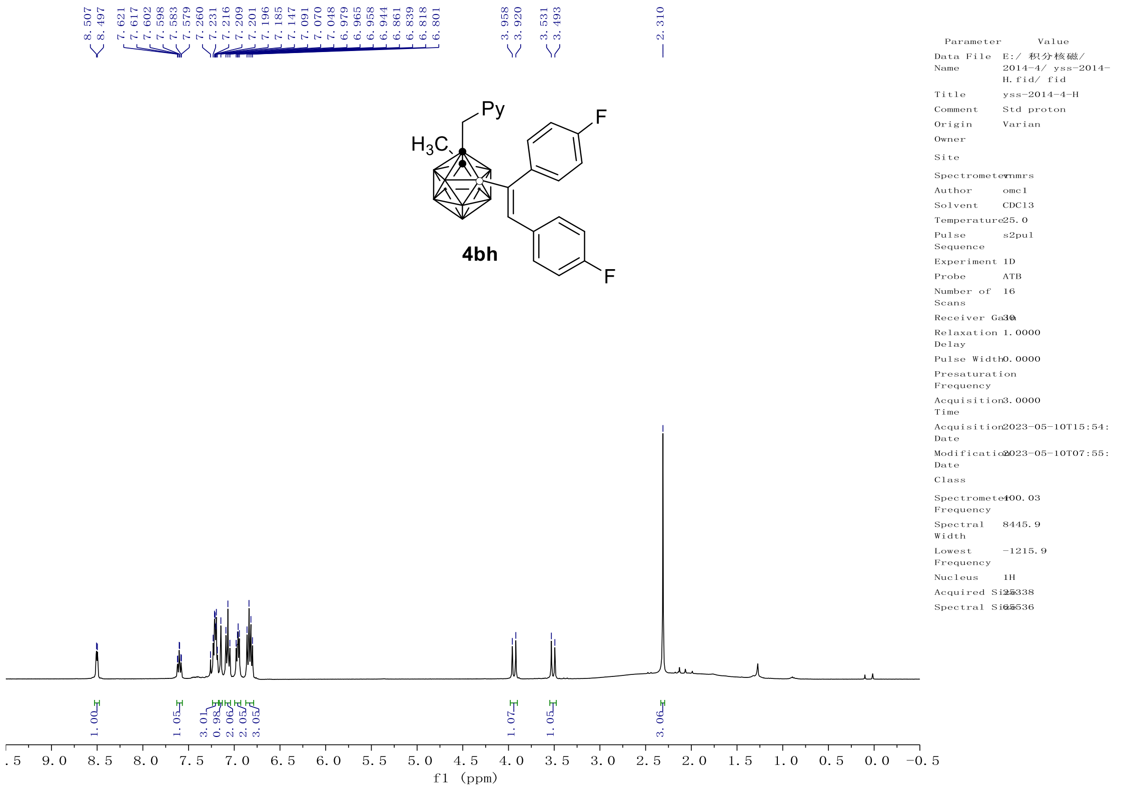

^{13}C{^{1}H} NMR (101 MHz, $CDCl_3$) of **4bh**

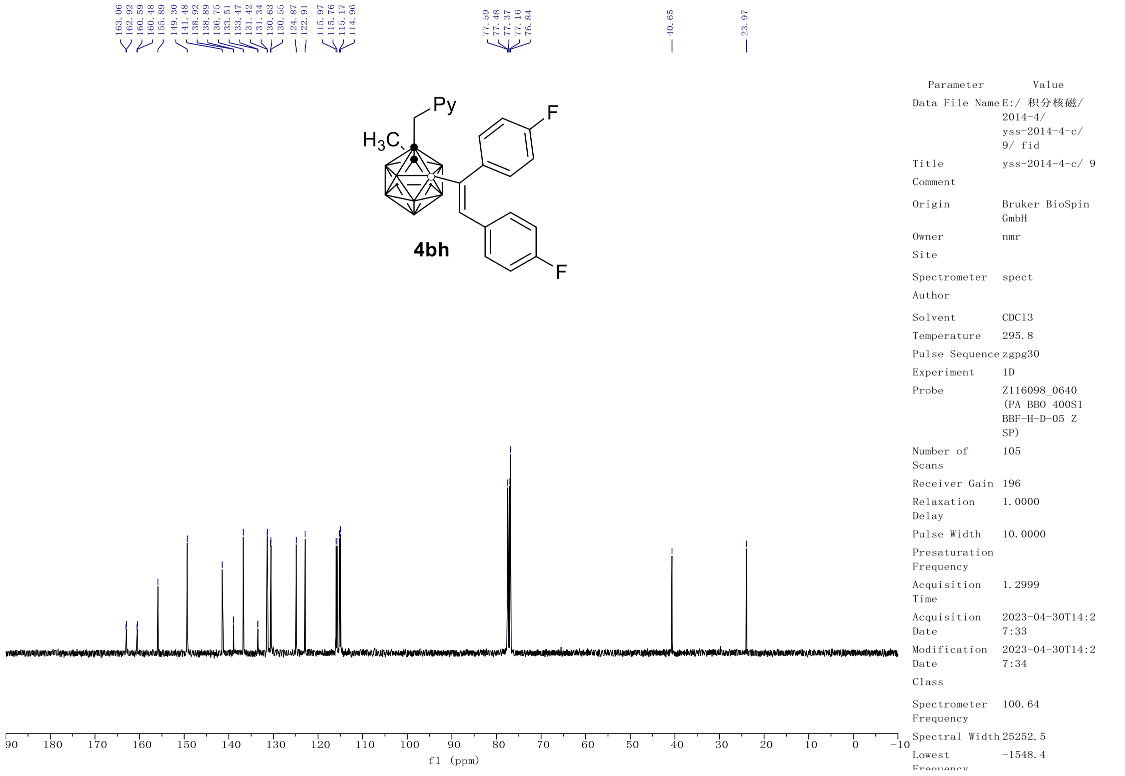

$^{11}B\{^{1}H\}$ NMR (128 MHz, $CDCl_3$) of **4bh**

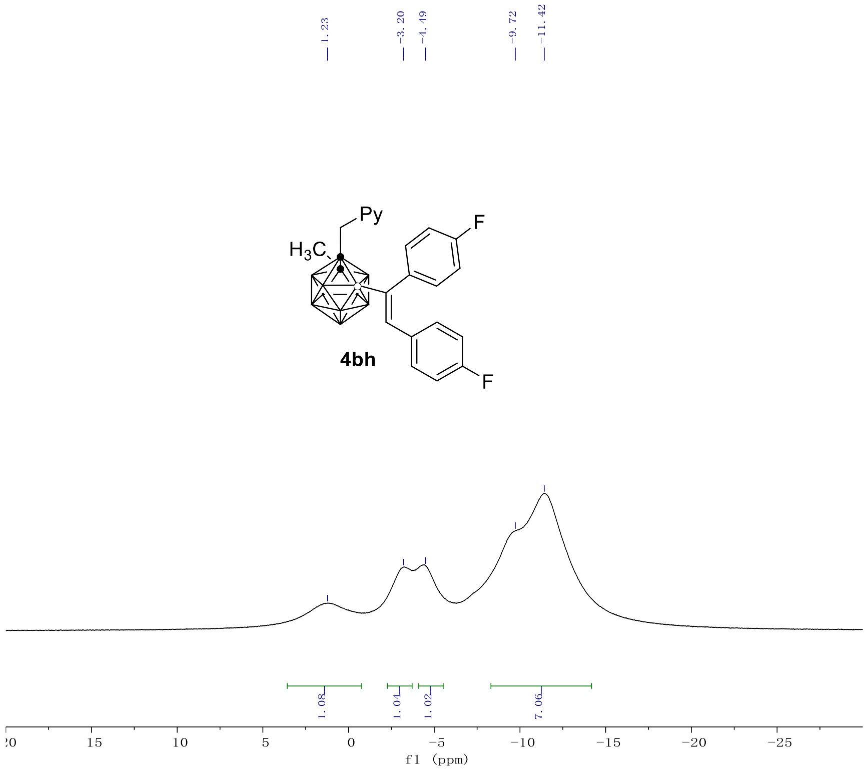

Parameter	Value
Data File Name	E:/ 积分核磁/ 2014-4/ yss-2014-4b-3/ 6/ fid
Title	yss-2014-4b-3/ 6
Comment	
Origin	Bruker BioSpin GmbH
Owner	nmr
Site	
Spectrometer	spect
Author	
Solvent	CDCl3
Temperature	295.9
Pulse Sequence	aring-b11
Experiment	1D
Probe	Z116098_0640 (PA BBO 400S1 BBF-H-D-05 Z SP)
Number of Scans	13
Receiver Gain	196
Relaxation Delay	1.0000
Pulse Width	10.0000
Presaturation Frequency	
Acquisition Time	1.2845
Acquisition Date	2023-04-30T14:14:08
Modification Date	2023-04-30T14:14:09
Class	
Spectrometer Frequency	128.40
Spectral Width	25510.2
Lowest Frequency	-12755.1
Nucleus	11B
Acquired Size	32768
Spectral Size	65536

^{11}B NMR (128 MHz, $CDCl_3$) of **4bh**

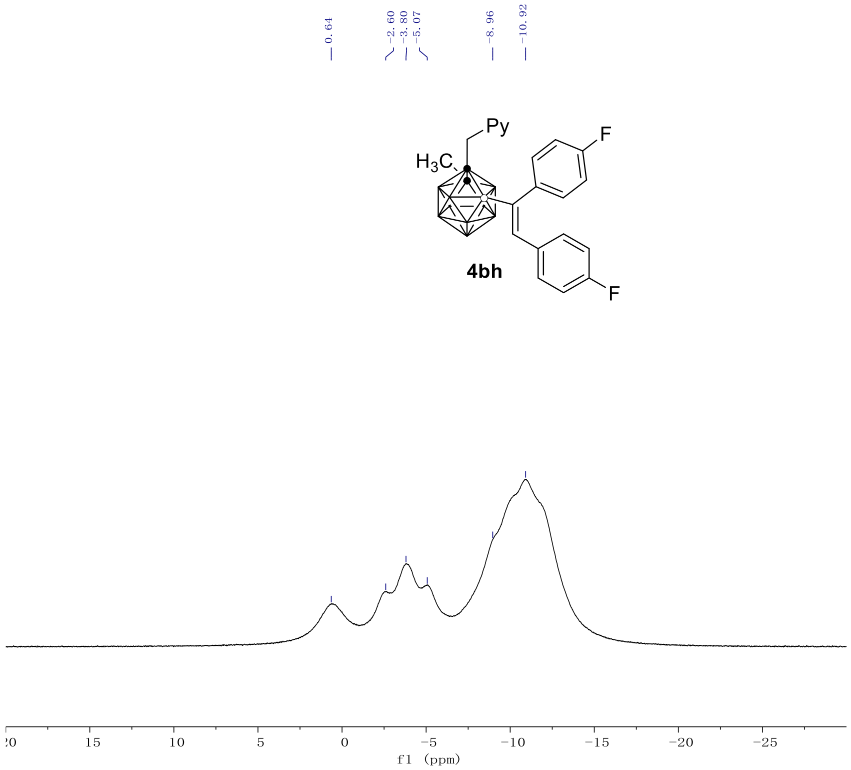

Parameter	Value
Data File Name	E:/ 积分核磁/ 2014-4/ yss-2014-4-nob/ 8/ fid
Title	yss-2014-4-nob/ 8
Comment	
Origin	Bruker BioSpin GmbH
Owner	nmr
Site	
Spectrometer	spect
Author	
Solvent	CDCl3
Temperature	295.7
Pulse Sequence	aring
Experiment	1D
Probe	Z116098_0640 (PA BBO 4C BBF-H-D-05 Z SP)
Number of Scans	8
Receiver Gain	196
Relaxation Delay	1.0000
Pulse Width	10.0000
Presaturation Frequency	
Acquisition Time	1.2845
Acquisition Date	2023-04-30T14:20:34
Modification Date	2023-04-30T14:20:35
Class	
Spectrometer Frequency	128.40
Spectral Width	25510.2
Lowest Frequency	-12755.1
Nucleus	11B
Acquired Size	32768
Spectral Size	65536

^{19}F NMR (376 MHz, $CDCl_3$) of **4bh**

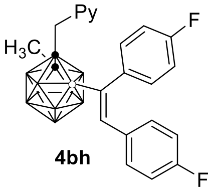

4bh

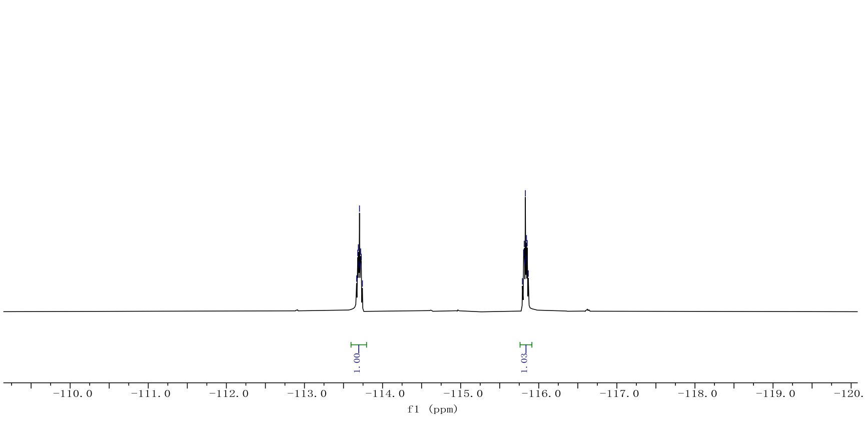

Parameter	Value
Data File Name	E:/ 积分核磁/ 2014-4/ 6-yss-2014-4-f/ 5/ fid
Title	6-yss-2014-4-f/ 5
Comment	
Origin	Bruker BioSpin GmbH
Owner	nmr
Site	
Spectrometer	spect
Author	
Solvent	CDCl3
Temperature	296.0
Pulse Sequence	zgflqn
Experiment	1D
Probe	Z116098_0640 (PA BBO 400S1 BBF-H-D-05 Z SP)
Number of Scans	8
Receiver Gain	196
Relaxation Delay	1.0000
Pulse Width	18.0000
Presaturation Frequency	
Acquisition Time	0.9999
Acquisition Date	2023-09-16T19:58:59
Modification Date	2023-09-16T19:59:00
Class	
Spectrometer Frequency	376.53
Spectral Width	75000.0
Lowest Frequency	-75156.4
Nucleus	19F
Acquired Size	74996
Spectral Size	262144

^{1}H NMR (400 MHz, $CDCl_3$) of **4bi**

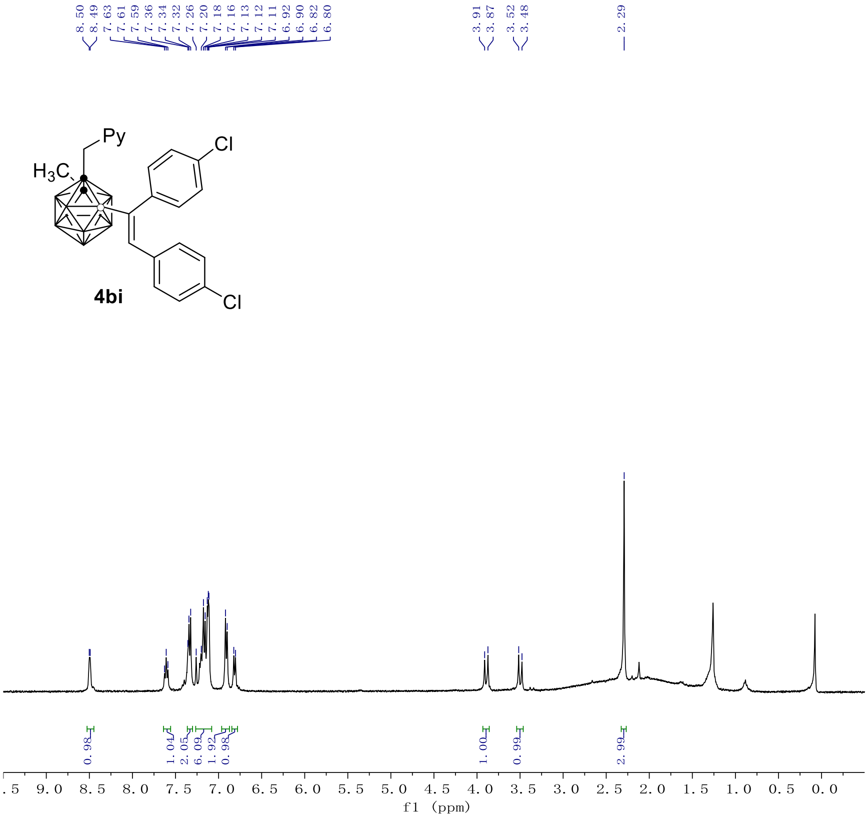

Parameter	Value
Data File Name	E:/ 积分核磁/ 2052-1/ yss-2052-1-h.fid/ fi
Title	yss-2052-1-h
Comment	Std proton
Origin	Varian
Owner	
Site	
Spectrometer	vnmrs
Author	omc1
Solvent	CDCl3
Temperature	25.0
Pulse Sequence	s2pul
Experiment	1D
Probe	ATB
Number of Scans	4
Receiver Gain	38
Relaxation Delay	1.0000
Pulse Width	0.0000
Presaturation Frequency	
Acquisition Time	3.0000
Acquisition Date	2023-07-04T15:02:10
Modification Date	2023-07-04T07:02:00
Class	
Spectrometer Frequency	400.03
Spectral Width	10080.6
Lowest Frequency	-2032.3
Nucleus	1H
Acquired Size	30242
Spectral Size	65536

^{13}C{^{1}H} NMR (101 MHz, $CDCl_3$) of **4bi**

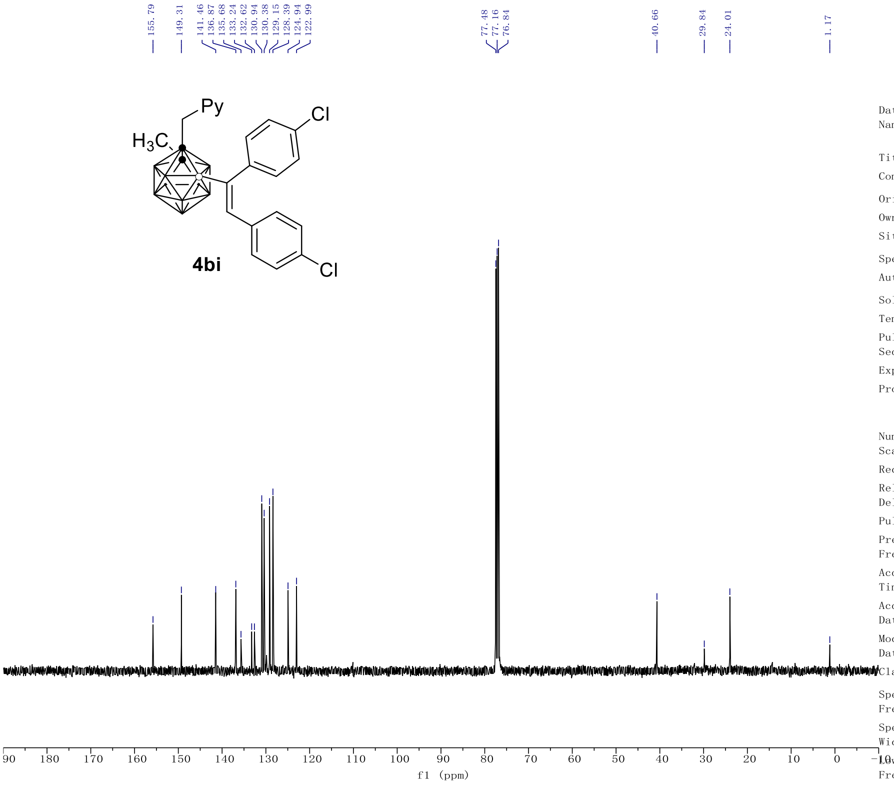

Parameter	Value
Data File Name	E:/ 积分核磁/ 2052-1/ yss-2052-1-c/ 4/ fid
Title	yss-2052-1-c/ 4
Comment	
Origin	Bruker BioSpin GmbH
Owner	nmr
Site	
Spectrometer	spect
Author	
Solvent	CDCl3
Temperature	296.8
Pulse Sequence	zgpg30
Experiment	1D
Probe	Z116098_0640 (PA BBO 400S1 BBF-H-D-05 Z SP)
Number of Scans	455
Receiver Gain	196
Relaxation Delay	1.0000
Pulse Width	10.0000
Presaturation Frequency	
Acquisition Time	1.2999
Acquisition Date	2023-07-04T19:08:18
Modification Date	2023-07-04T19:08:19
Class	
Spectrometer Frequency	100.64
Spectral Width	25252.5
Lowest Frequency	-1544.3

$^{11}B\{^1H\}$ NMR (128 MHz, $CDCl_3$) of **4bi**

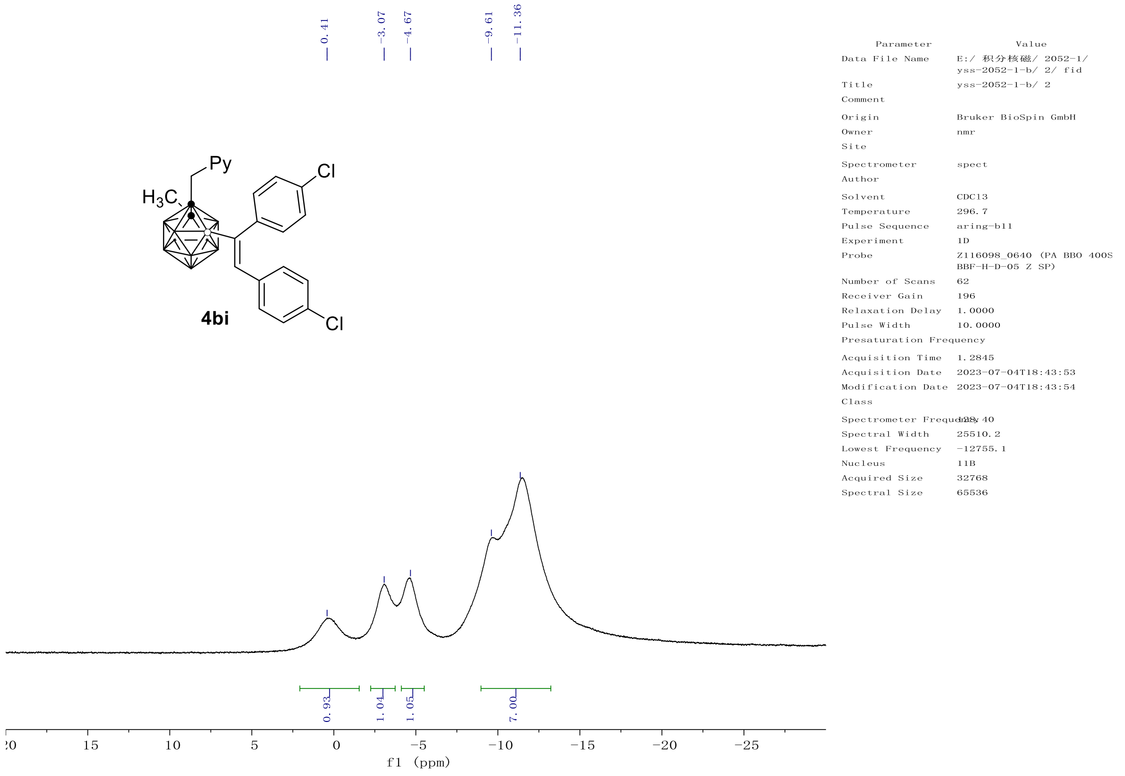

^{11}B NMR (128 MHz, $CDCl_3$) of **4bi**

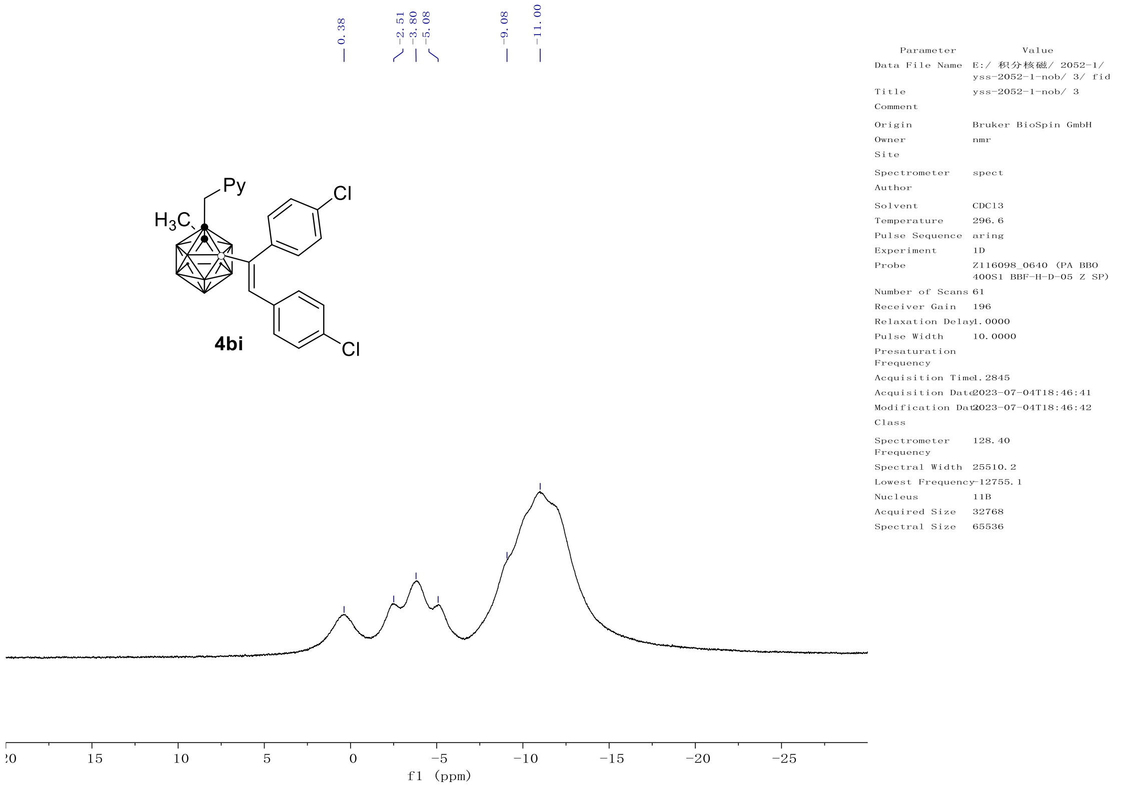

^{1}H NMR (400 MHz, $CDCl_3$) of **4bj**

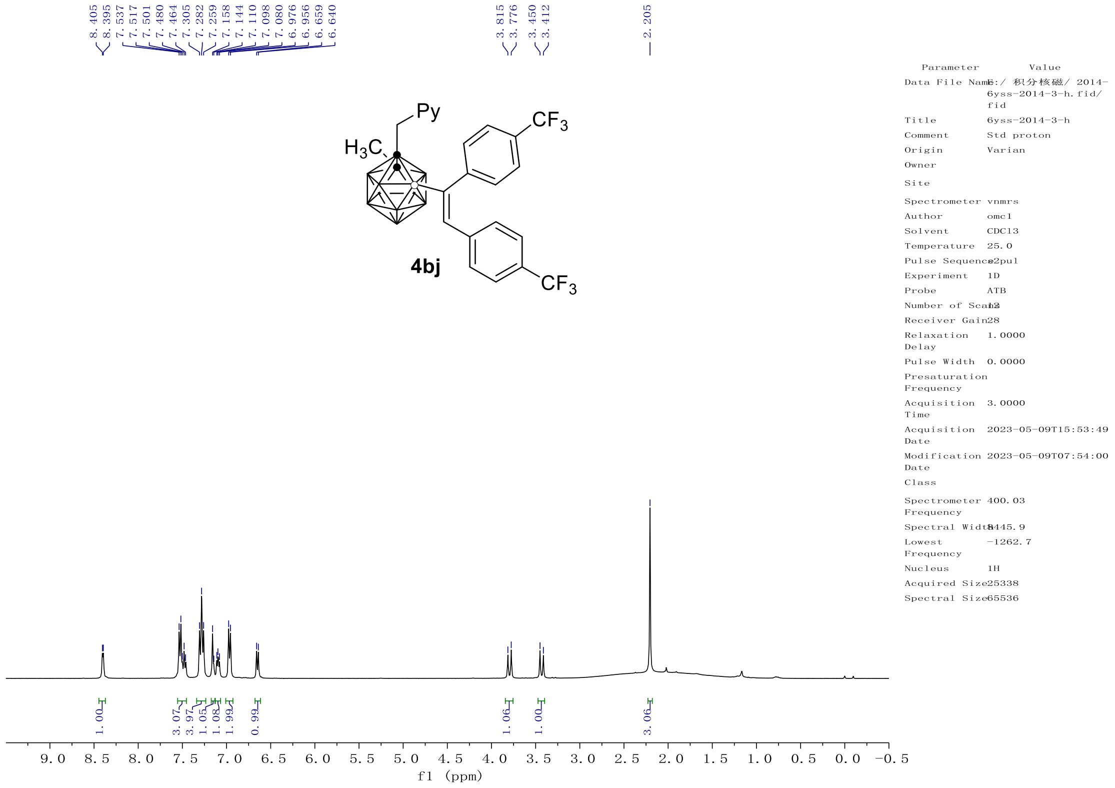

$^{13}C\{^{1}H\}$ NMR (101 MHz, $CDCl_3$) of **4bj**

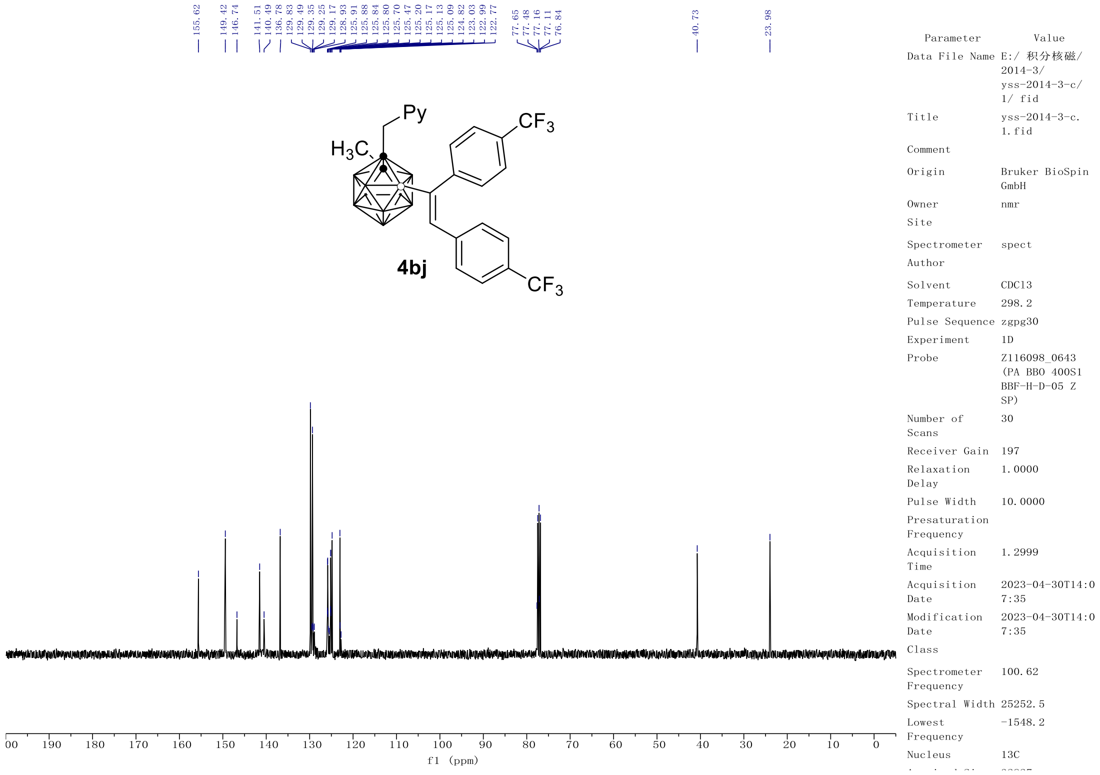

$^{11}B\{^1H\}$ NMR (128 MHz, $CDCl_3$) of **4bj**

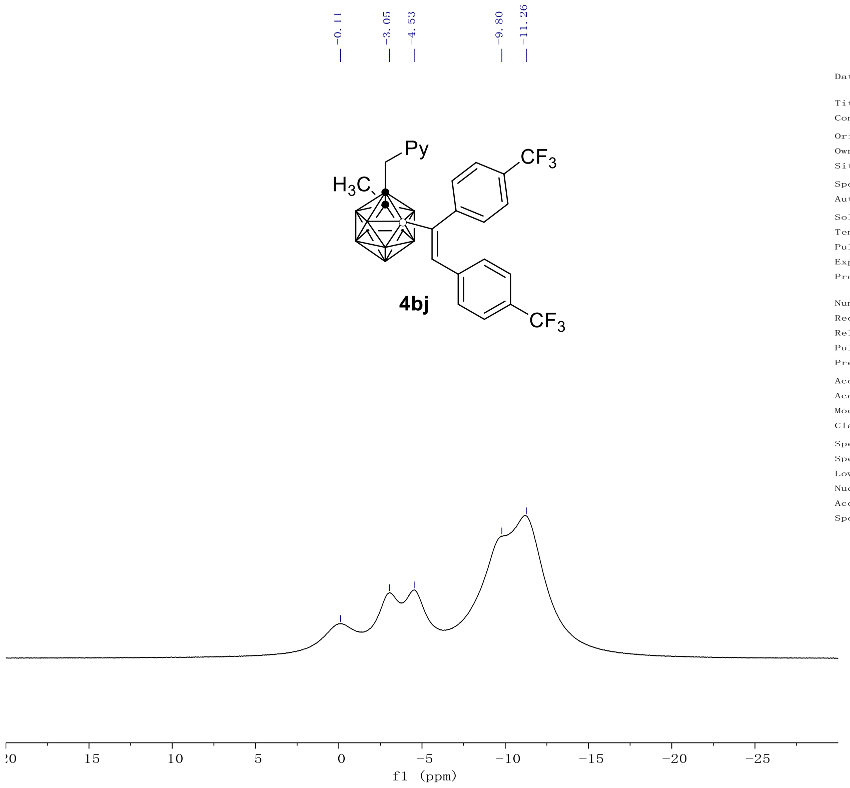

Parameter	Value
Data File Name	E:/ 积分核磁/ 2014-3/ yss-2014-3-b-2/ 6/ fid
Title	yss-2014-3-b-2/ 6
Comment	
Origin	Bruker BioSpin GmbH
Owner	nmr
Site	
Spectrometer	spect
Author	
Solvent	CDCl3
Temperature	295.9
Pulse Sequence	aring-bl1
Experiment	1D
Probe	Z116098_0640 (PA BBO 400S BBF-H-D-05 Z SP)
Number of Scans	13
Receiver Gain	196
Relaxation Delay	1.0000
Pulse Width	10.0000
Presaturation Frequency	
Acquisition Time	1.2845
Acquisition Date	2023-04-30T15:31:09
Modification Date	2023-04-30T15:31:10
Class	
Spectrometer Frequency	128.40
Spectral Width	25510.2
Lowest Frequency	-12755.1
Nucleus	11B
Acquired Size	32768
Spectral Size	65536

^{11}B NMR (128 MHz, $CDCl_3$) of **4bj**

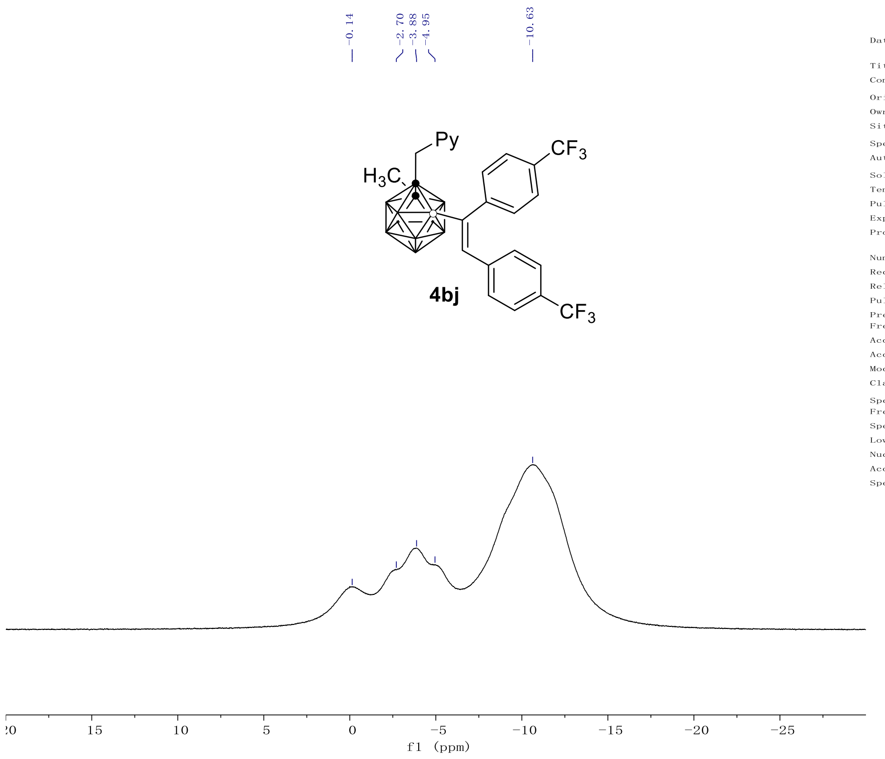

Parameter	Value
Data File Name	E:/ 积分核磁/ 2014-3/ yss-2014-3-nob/ 4/ fid
Title	yss-2014-3-nob/ 4
Comment	
Origin	Bruker BioSpin GmbH
Owner	nmr
Site	
Spectrometer	spect
Author	
Solvent	C6D6
Temperature	295.7
Pulse Sequence	aring
Experiment	1D
Probe	Z116098_0640 (PA BBO 4(BBF-H-D-05 Z SP)
Number of Scans	8
Receiver Gain	196
Relaxation Delay	1.0000
Pulse Width	10.0000
Presaturation Frequency	
Acquisition Time	1.2845
Acquisition Date	2023-04-30T15:26:40
Modification Date	2023-04-30T15:26:41
Class	
Spectrometer Frequency	128.40
Spectral Width	25510.2
Lowest Frequency	-12755.1
Nucleus	11B
Acquired Size	32768
Spectral Size	65536

^{19}F NMR (376 MHz, $CDCl_3$) of **4bj**

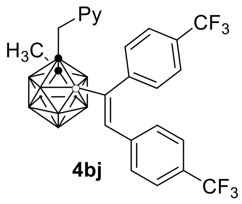

4bj

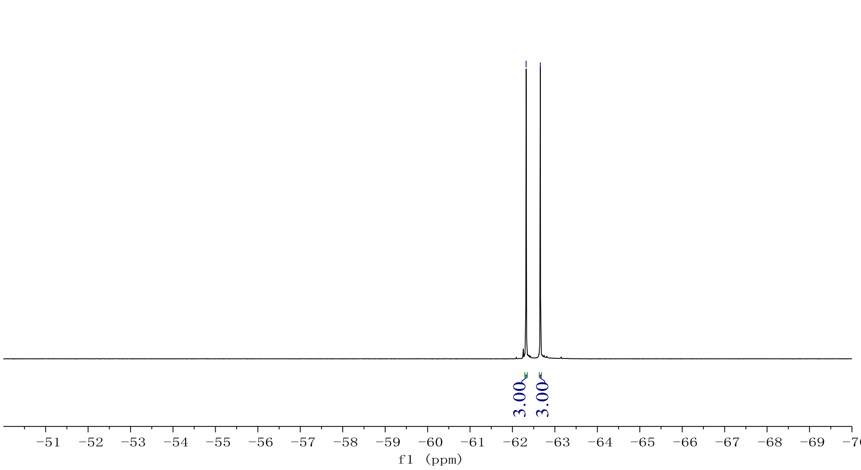

Parameter	Value
Data File Name	E:/ 积分核磁/ 2014-3/ yss-2014-3-f/ 2/ fid
Title	yss-2014-3-f/ 2
Comment	
Origin	Bruker BioSpin GmbH
Owner	nmr
Site	
Spectrometer	spect
Author	
Solvent	CDCl3
Temperature	295.4
Pulse Sequence	zgflqn
Experiment	1D
Probe	Z116098_0640 (PA BBO 40 BBF-H-D-05 Z SP)
Number of Scans	13
Receiver Gain	196
Relaxation Delay	1.0000
Pulse Width	18.0000
Presaturation Frequency	
Acquisition Time	0.8000
Acquisition Date	2023-05-10T22:18:37
Modification Date	2023-05-10T22:18:37
Class	
Spectrometer Frequency	376.53
Spectral Width	300000.0
Lowest Frequency	-187656.4
Nucleus	19F
Acquired Size	239985
Spectral Size	524288

^{1}H NMR (400 MHz, $CDCl_3$) of **4bk**

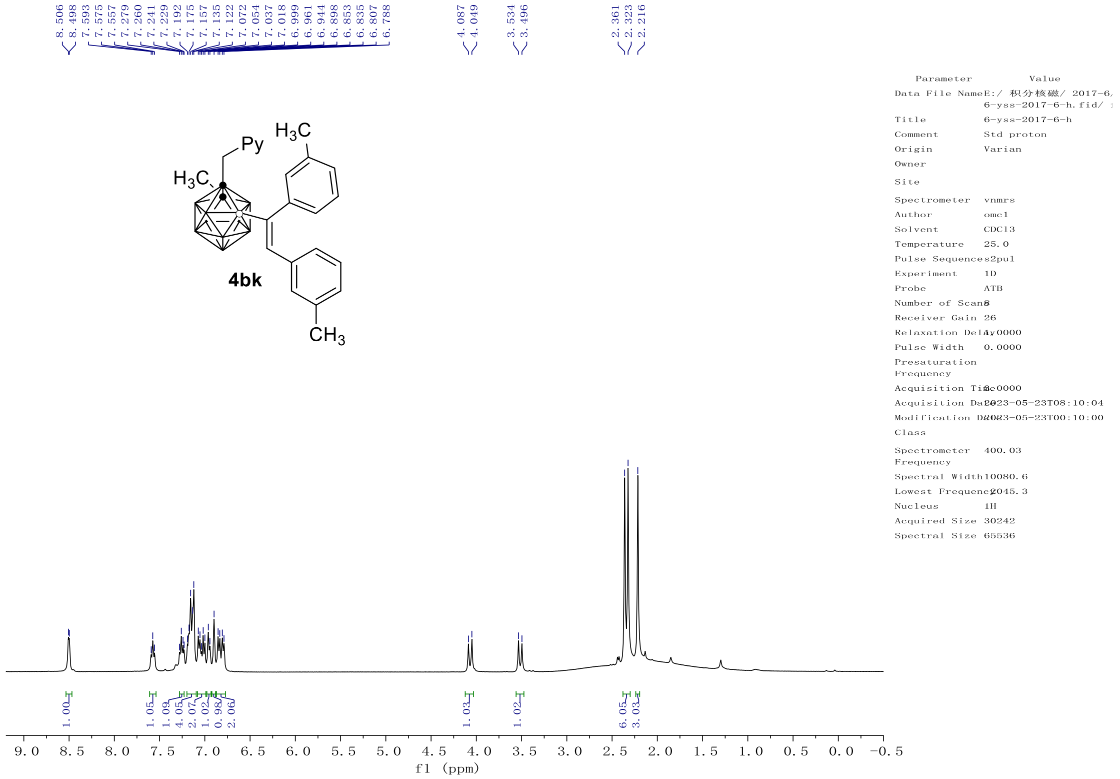

^{13}C{^{1}H} NMR (101 MHz, $CDCl_3$) of **4bk**

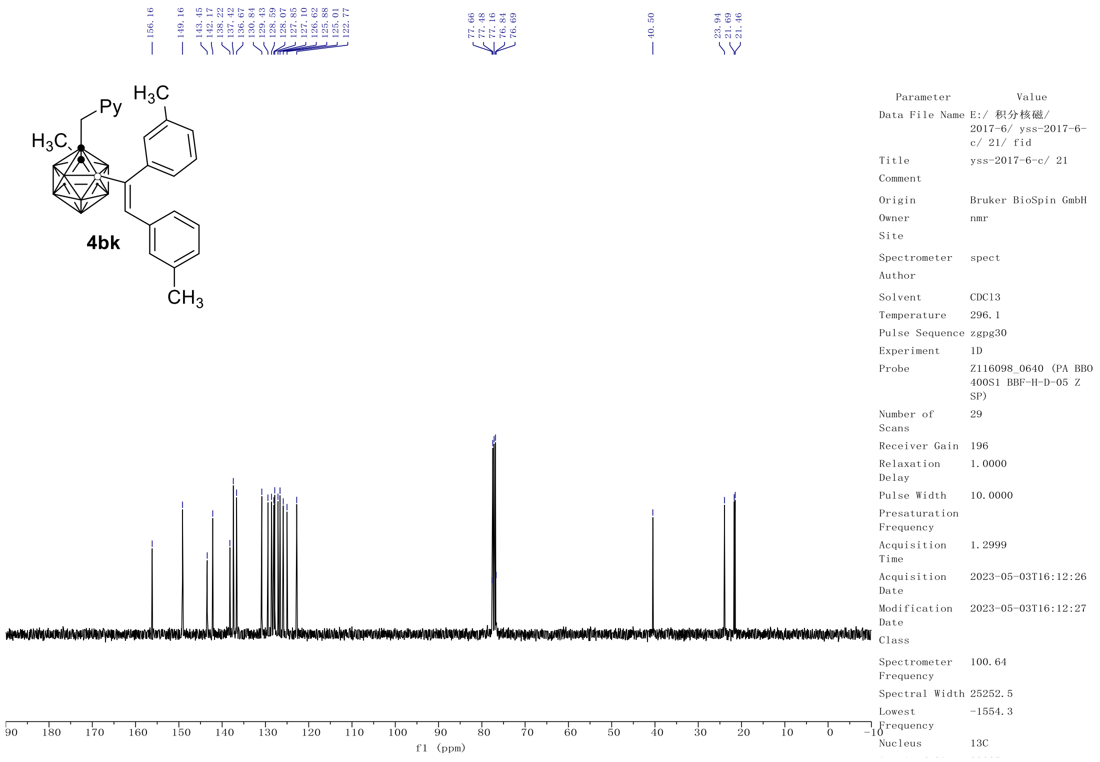

$^{11}B\{^{1}H\}$ NMR (128 MHz, $CDCl_3$) of **4bk**

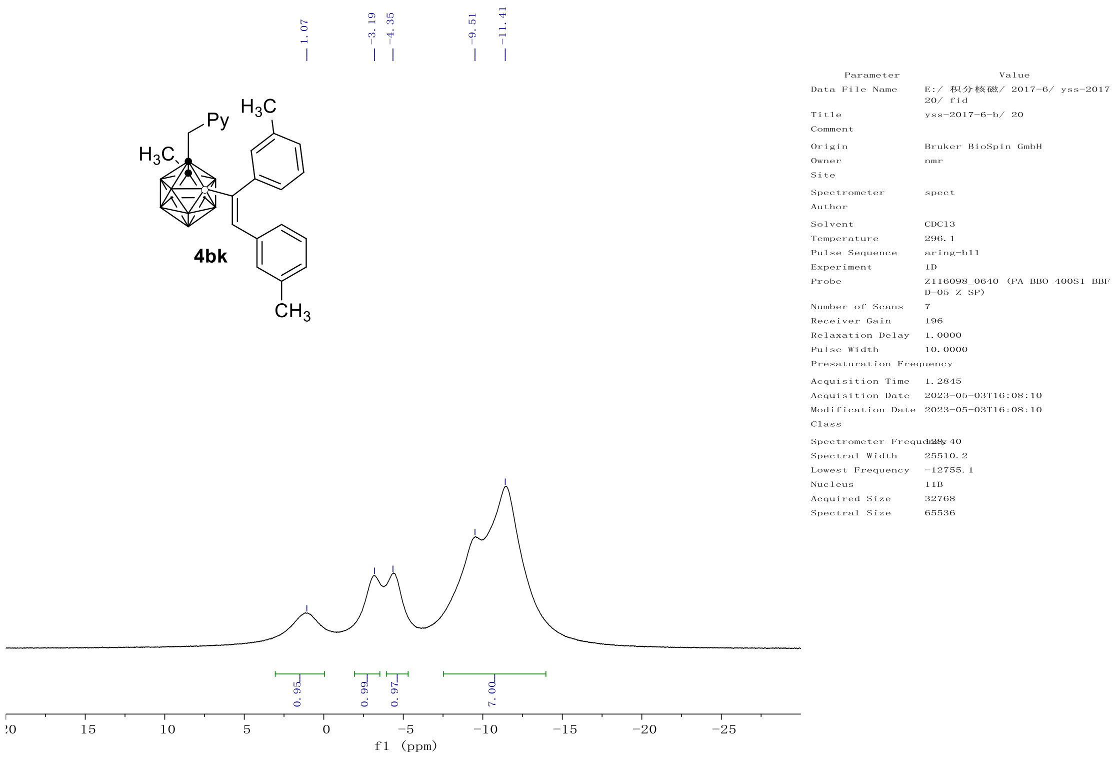

^{11}B NMR (128 MHz, $CDCl_3$) of **4bk**

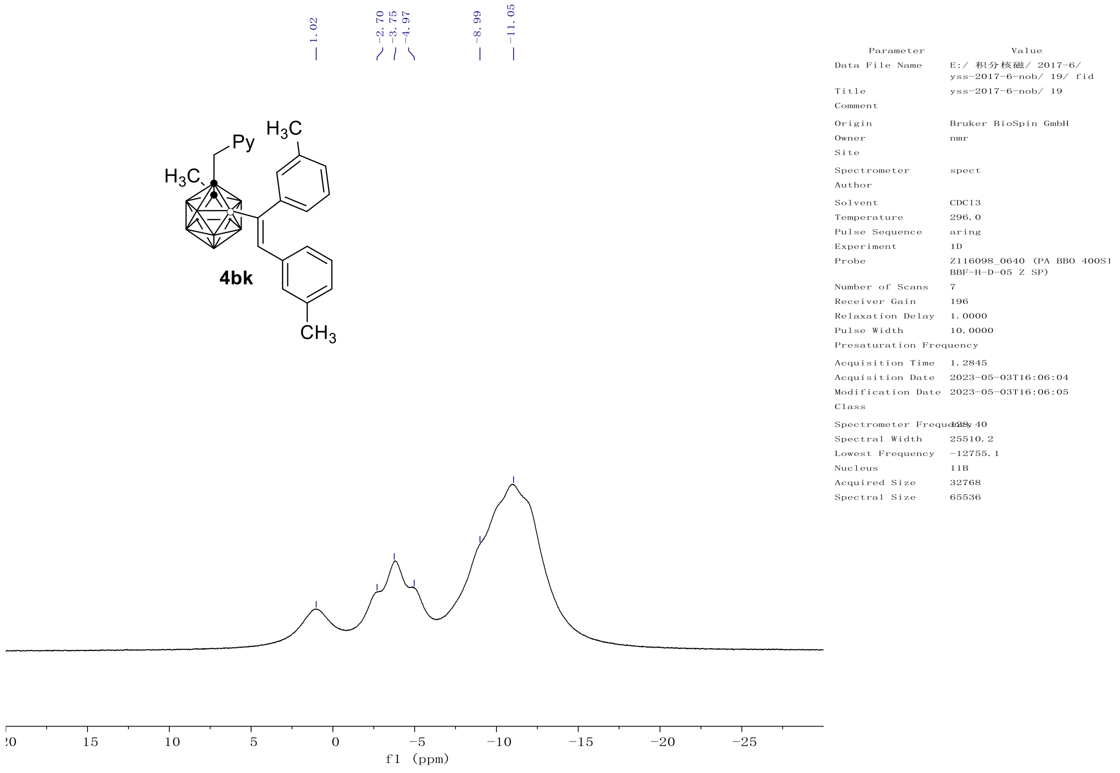

^{1}H NMR (400 MHz, $CDCl_3$) of **4bl**

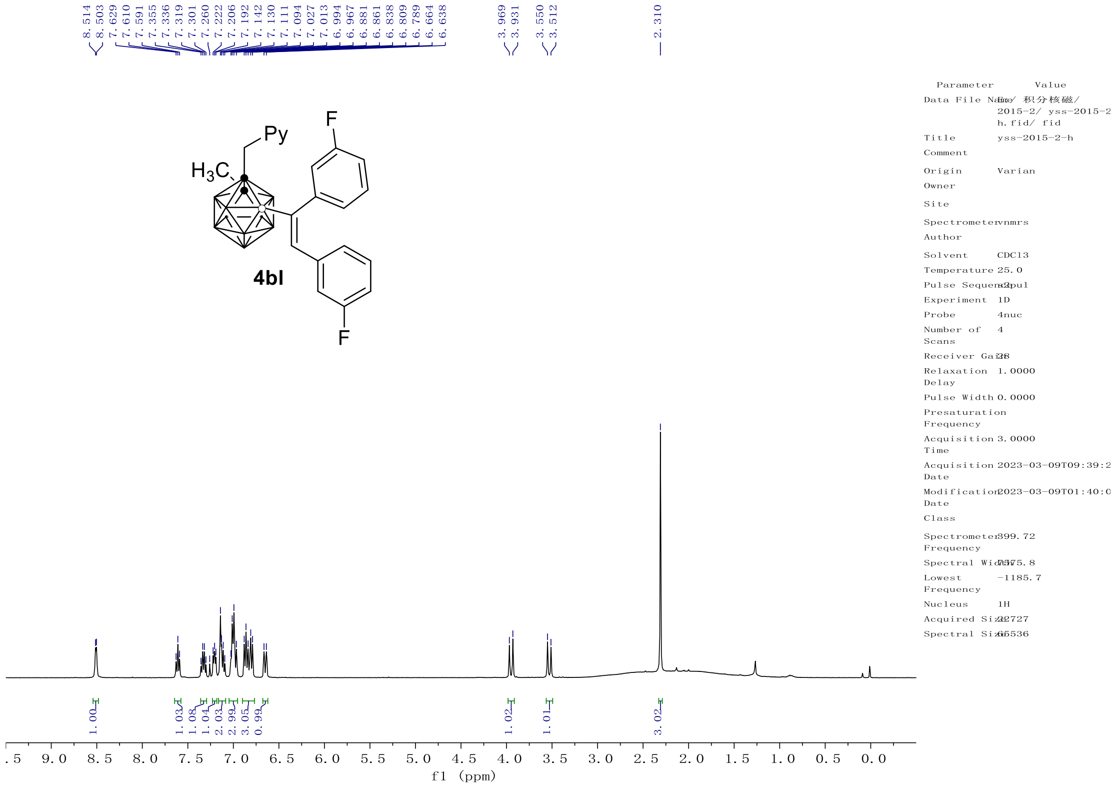

$^{13}C\{^{1}H\}$ NMR (101 MHz, $CDCl_3$) of **4bl**

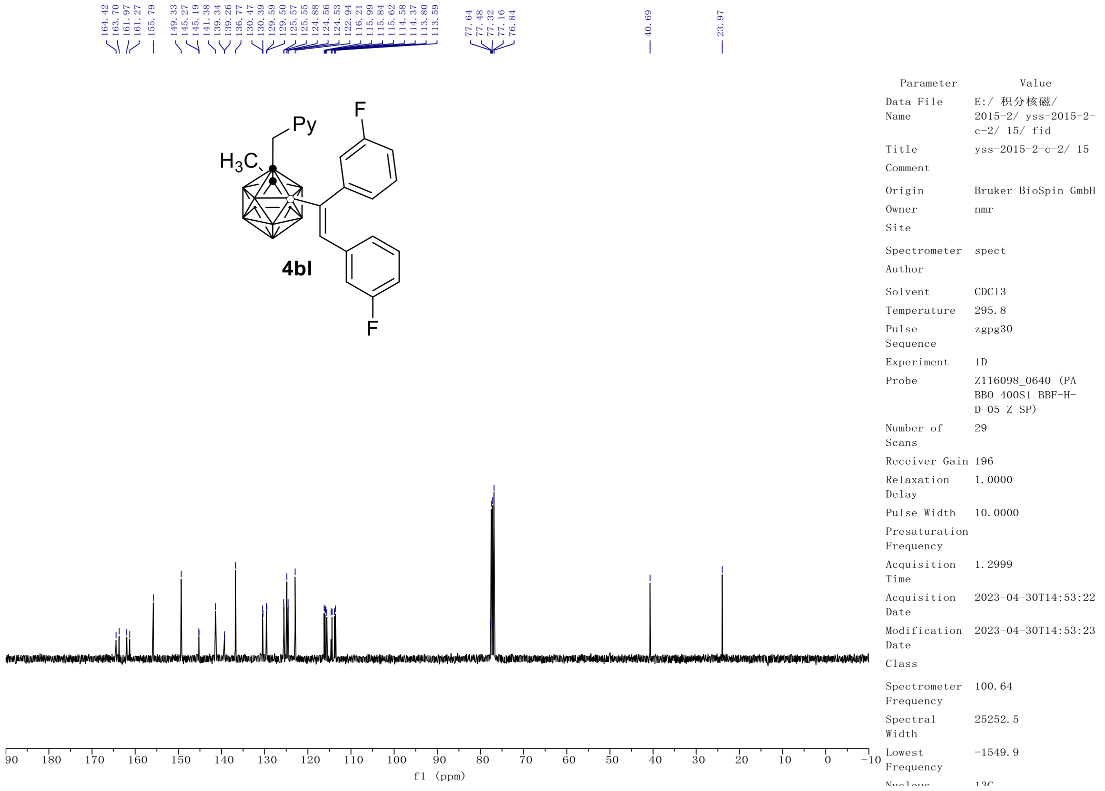

$^{11}B\{^{1}H\}$ NMR (128 MHz, $CDCl_3$) of **4bl**

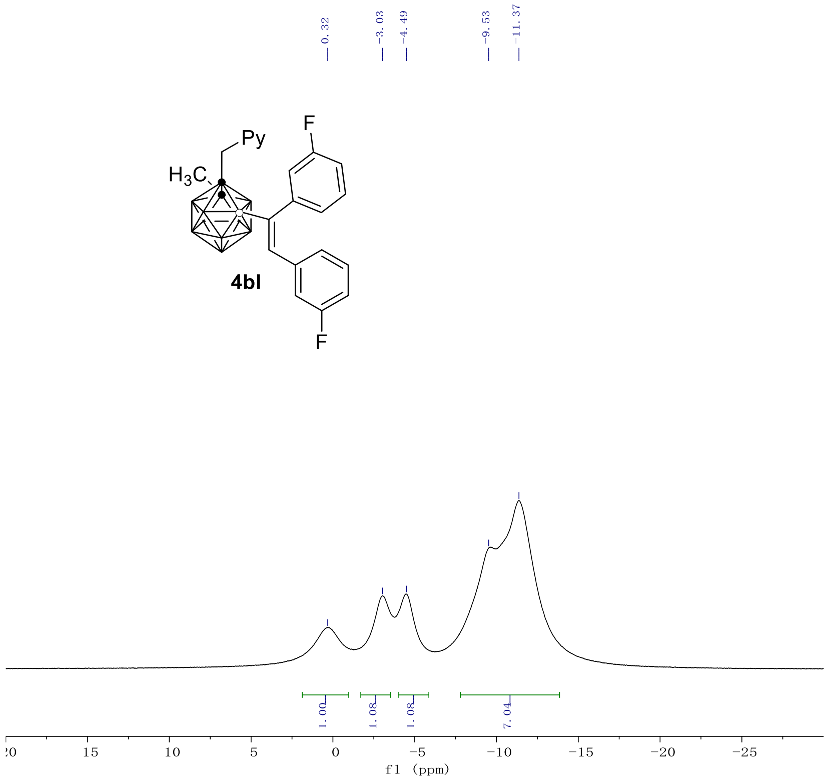

Parameter	Value
Data File Name	E:/ 积分核磁/ 2015-2/ 2015 b/ fid
Title	2015-2/ 2015-2-b
Comment	
Origin	Bruker BioSpin GmbH
Owner	nmr
Site	
Spectrometer	spect
Author	
Solvent	CDC13
Temperature	296.0
Pulse Sequence	aring-b11
Experiment	1D
Probe	Z116098_0640 (PA BBO 400S1 BBF-H-D-05 Z SP)
Number of Scans	18
Receiver Gain	196
Relaxation Delay	1.0000
Pulse Width	10.0000
Presaturation Frequency	
Acquisition Time	1.2845
Acquisition Date	2023-04-30T14:42:59
Modification Date	2023-04-30T14:42:59
Class	
Spectrometer Frequency	128.40
Spectral Width	25510.2
Lowest Frequency	-12755.1
Nucleus	11B
Acquired Size	32768
Spectral Size	65536

^{11}B NMR (128 MHz, $CDCl_3$) of **4bl**

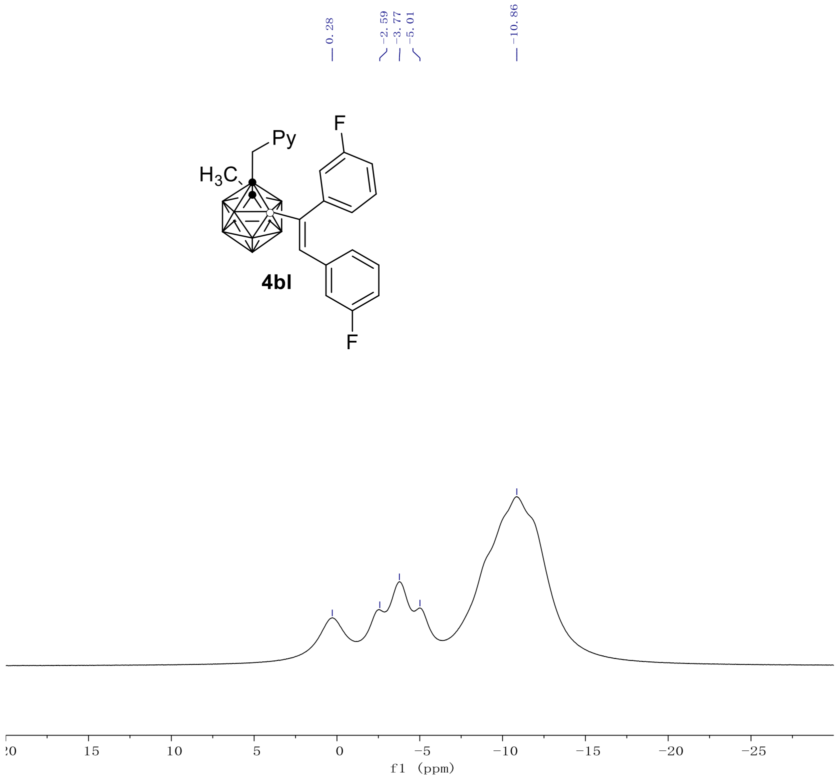

Parameter	Value
Data File Name	E:/ 积分核磁/ 2015-2/ 201 nob/ fid
Title	2015-2/ 2015-2-nob
Comment	
Origin	Bruker BioSpin GmbH
Owner	nmr
Site	
Spectrometer	spect
Author	
Solvent	CDC13
Temperature	295.7
Pulse Sequence	aring
Experiment	1D
Probe	Z116098_0640 (PA BBO 400S BBF-H-D-05 Z SP)
Number of Scans	70
Receiver Gain	196
Relaxation Delay	1.0000
Pulse Width	10.0000
Presaturation Frequency	
Acquisition Time	1.2845
Acquisition Date	2023-04-30T14:48:08
Modification Date	2023-04-30T14:48:09
Class	
Spectrometer Frequency	128.40
Spectral Width	25510.2
Lowest Frequency	-12755.1
Nucleus	11B
Acquired Size	32768
Spectral Size	65536

^{19}F NMR (376 MHz, $CDCl_3$) of **4bl**

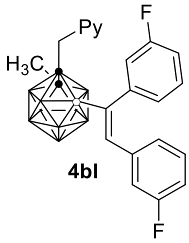

4bl

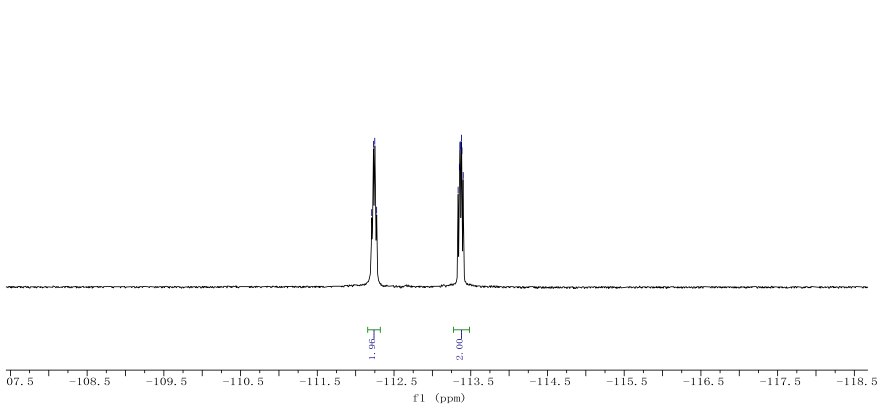

Parameter	Value
Data File Name	E:/ 积分核磁/ 2015-2/ yss-2015-2-f/ 4/ fid
Title	yss-2015-2-f/ 4
Comment	
Origin	Bruker BioSpin GmbH
Owner	nmr
Site	
Spectrometer	spect
Author	
Solvent	CDCl3
Temperature	295.5
Pulse Sequence	zgflqn
Experiment	1D
Probe	Z116098_0640 (PA BBO 400S1 BBF-H-D-05 Z SP)
Number of Scans	6
Receiver Gain	196
Relaxation Delay	1.0000
Pulse Width	18.0000
Presaturation Frequency	
Acquisition Time	0.8000
Acquisition Date	2023-05-10T22:23:59
Modification Date	2023-05-10T22:23:59
Class	
Spectrometer Frequency	376.53
Spectral Width	300000.0
Lowest Frequency	-187656.4

^{1}H NMR (400 MHz, $CDCl_3$) of **4bm**

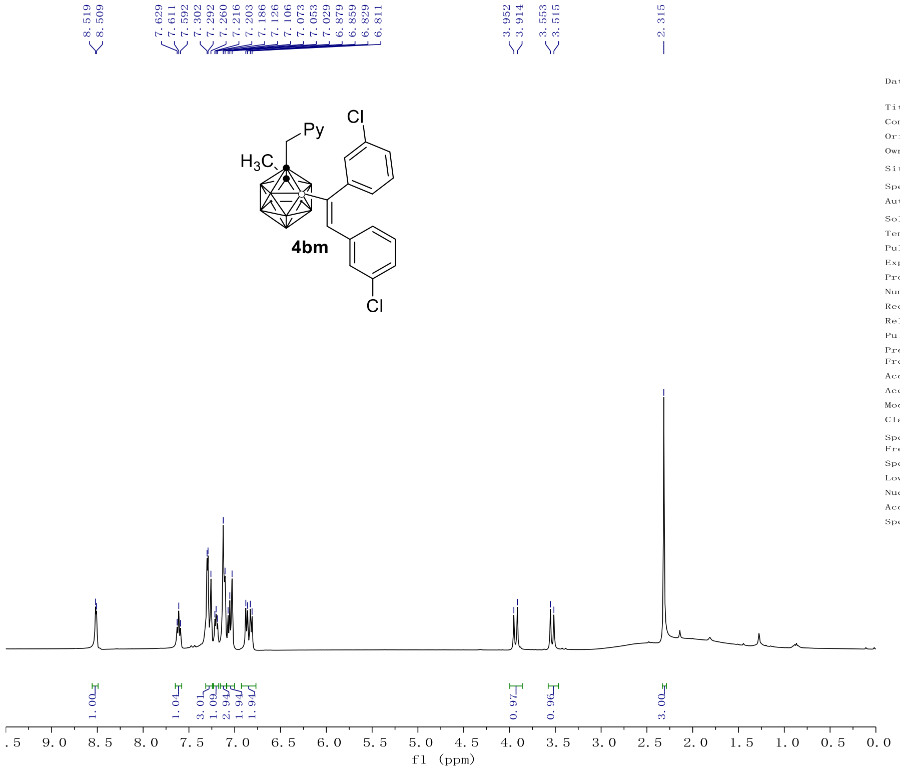

Parameter	Value
Data File Name	E:/ 积分核磁/ 2015-6/ 6yss-2015-6-h.fid/ fi[illegible]
Title	6yss-2015-6-h
Comment	Std proton
Origin	Varian
Owner	
Site	
Spectrometer	vnmrs
Author	
Solvent	CDCl3
Temperature	25.0
Pulse Sequence	s2pul
Experiment	1D
Probe	ATB
Number of Scans	8
Receiver Gain	34
Relaxation Delay	1.0000
Pulse Width	0.0000
Presaturation Frequency	
Acquisition Time	3.0000
Acquisition Date	2023-05-15T21:31:19
Modification Date	2023-05-15T13:32:00
Class	
Spectrometer Frequency	400.03
Spectral Width	6410.3
Lowest Frequency	-1.3
Nucleus	1H
Acquired Size	19231
Spectral Size	65536

^{13}C{^{1}H} NMR (101 MHz, $CDCl_3$) of **4bm**

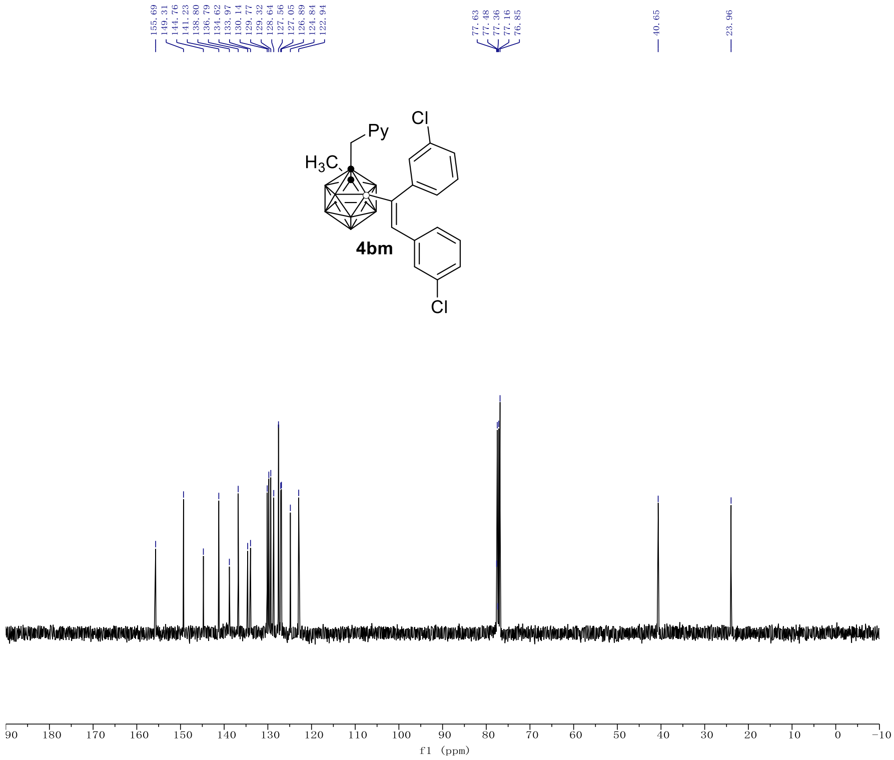

Parameter	Value
Data File Name	E:/ 积分核磁/ 2015-6/ yss-2015-6-c/ 8/ fid
Title	yss-2015-6-c/ 8
Comment	
Origin	Bruker BioSpin GmbH
Owner	nmr
Site	
Spectrometer	spect
Author	
Solvent	CDCl3
Temperature	296.1
Pulse Sequence	zgpg30
Experiment	1D
Probe	Z116098_0640 (PA BBO 400S1 BBF-H-D-05 Z SP)
Number of Scans	28
Receiver Gain	196
Relaxation Delay	1.0000
Pulse Width	10.0000
Presaturation Frequency	
Acquisition Time	1.2999
Acquisition Date	2023-05-03T15:38:20
Modification Date	2023-05-03T15:38:20
Class	
Spectrometer Frequency	100.64
Spectral Width	25252.5
Lowest Frequency	-1553.9
Nucleus	13C
Acquired Size	32827
[illegible]	[illegible]

$^{11}B\{^{1}H\}$ NMR (128 MHz, $CDCl_3$) of **4bm**

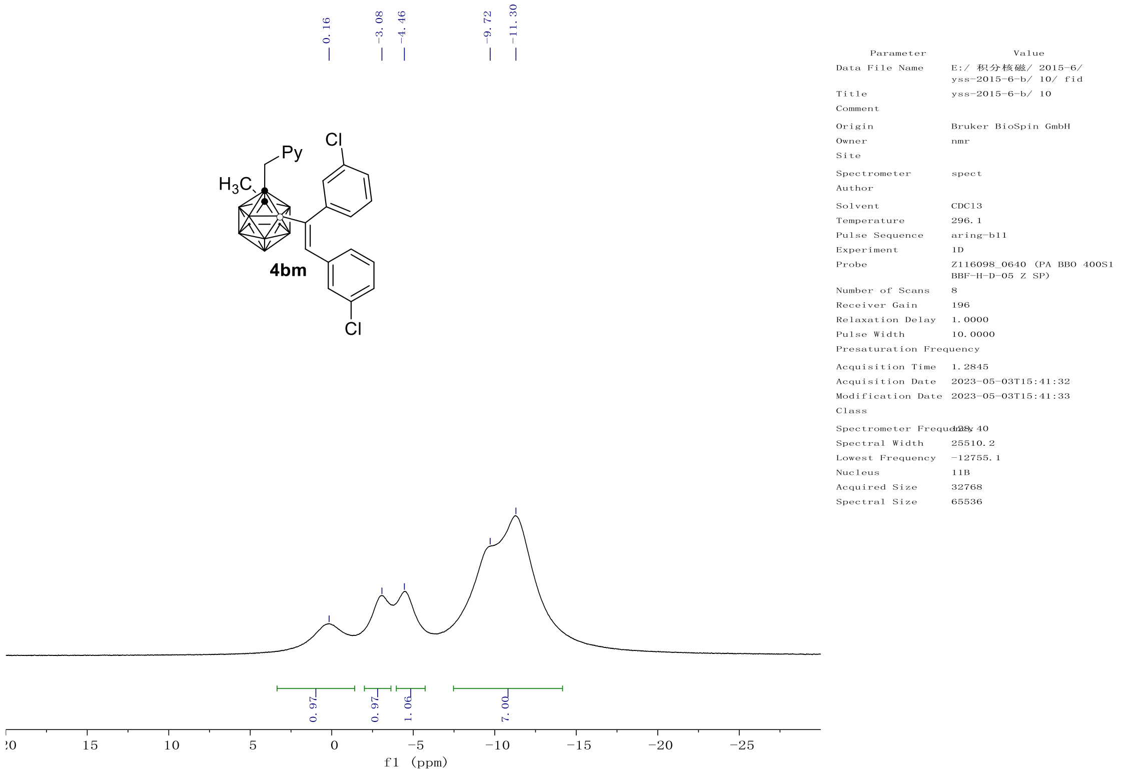

^{11}B NMR (128 MHz, $CDCl_3$) of **4bm**

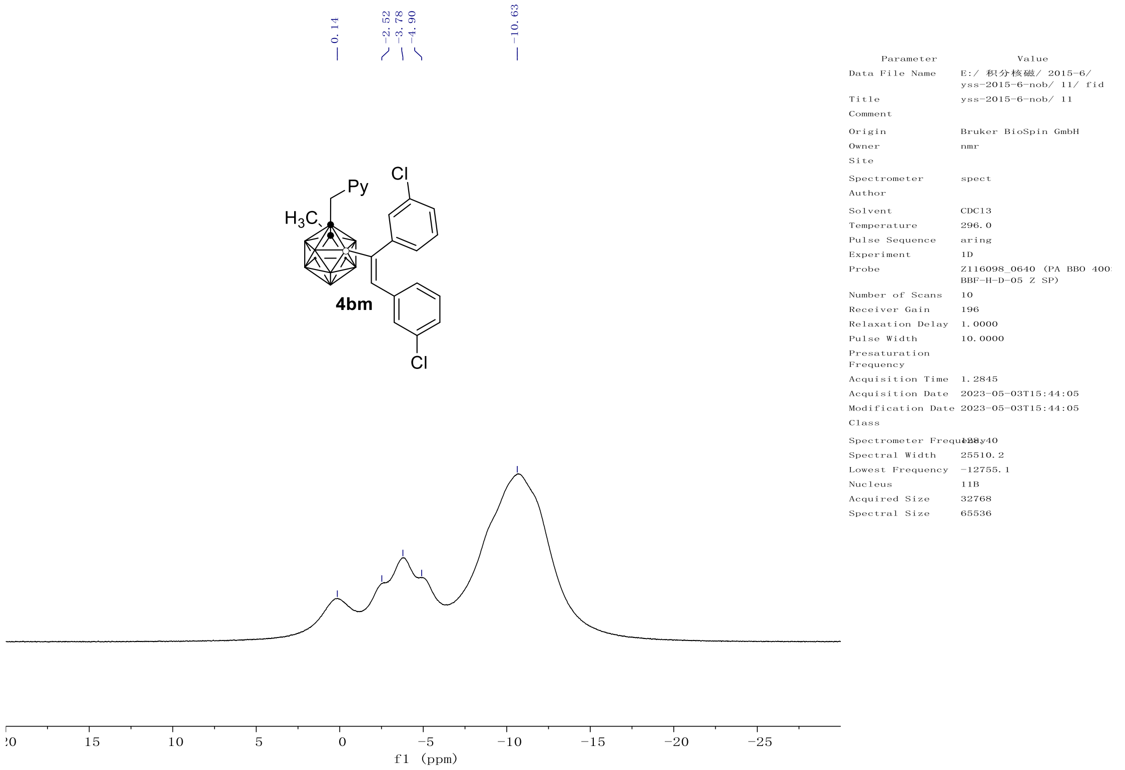

^1H NMR (400 MHz, $CDCl_3$) of **4bn**

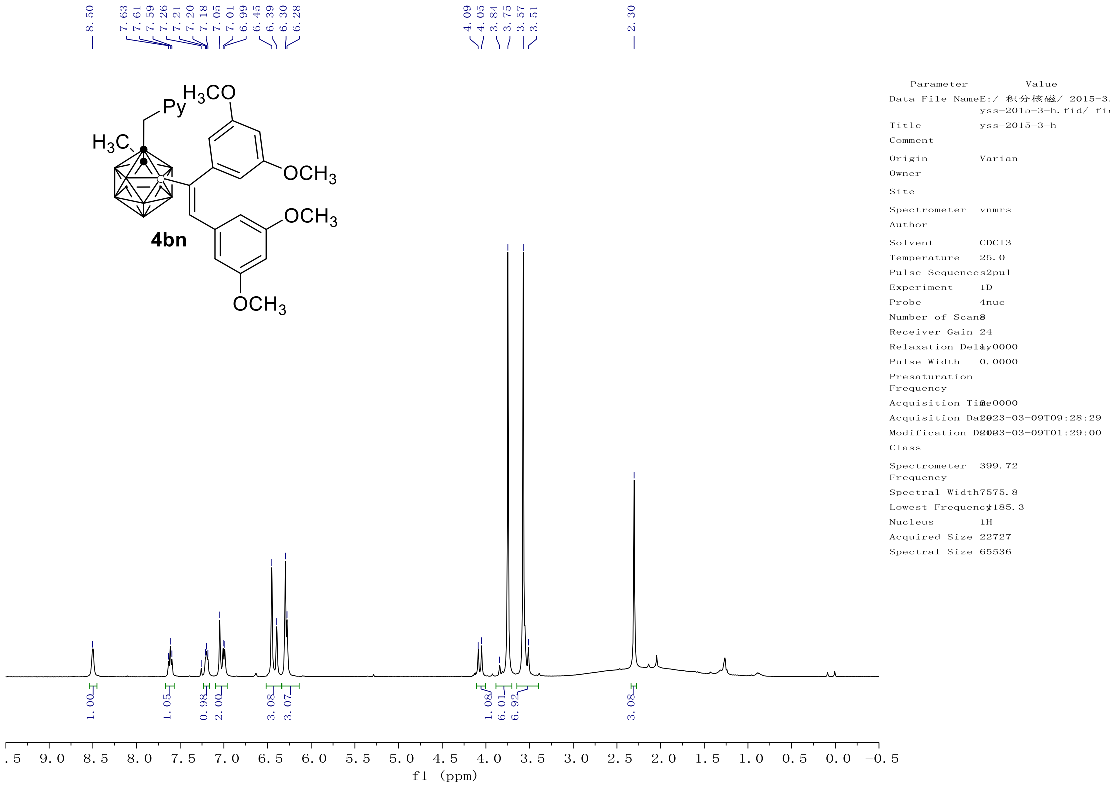

$^{13}C\{^1H\}$ NMR (101 MHz, $CDCl_3$) of **4bn**

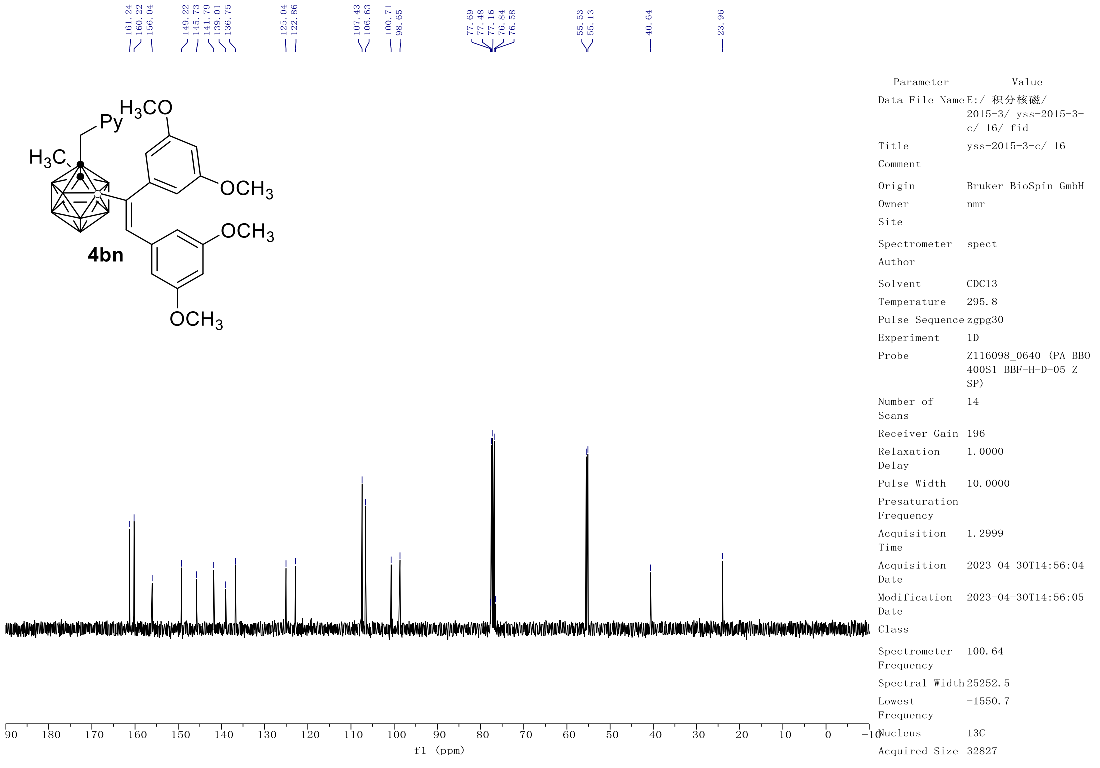

$^{11}B\{^{1}H\}$ NMR (128 MHz, $CDCl_3$) of **4bn**

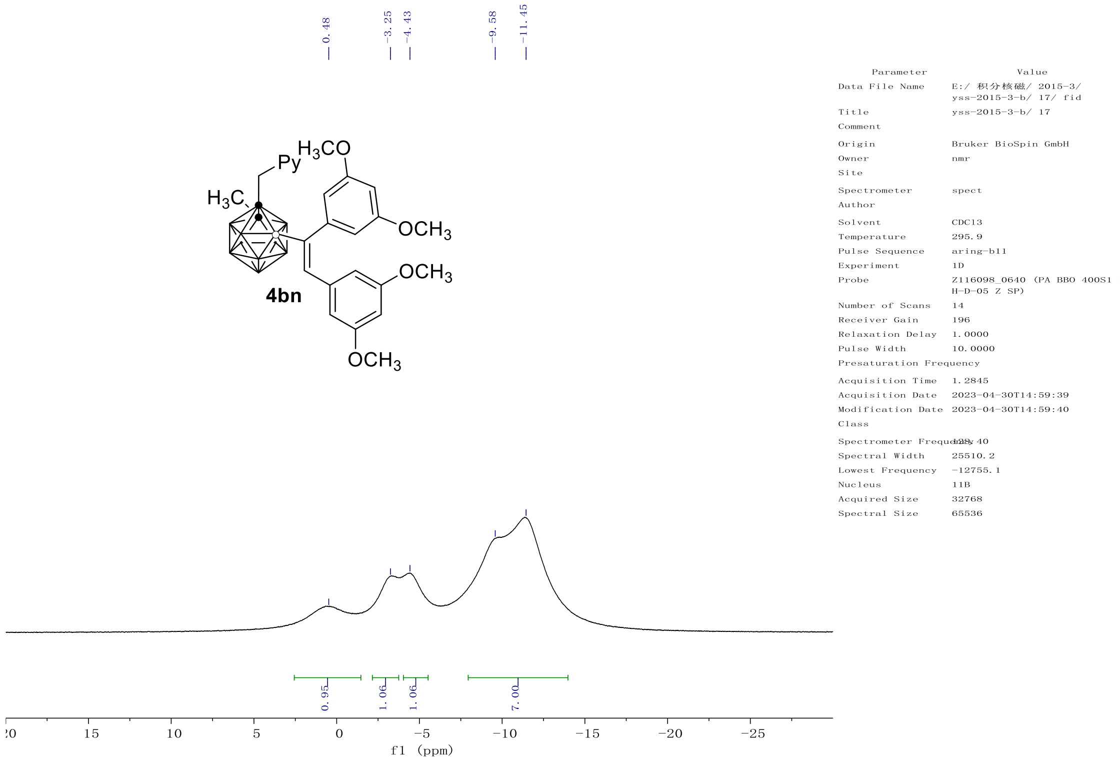

^{11}B NMR (128 MHz, $CDCl_3$) of **4bn**

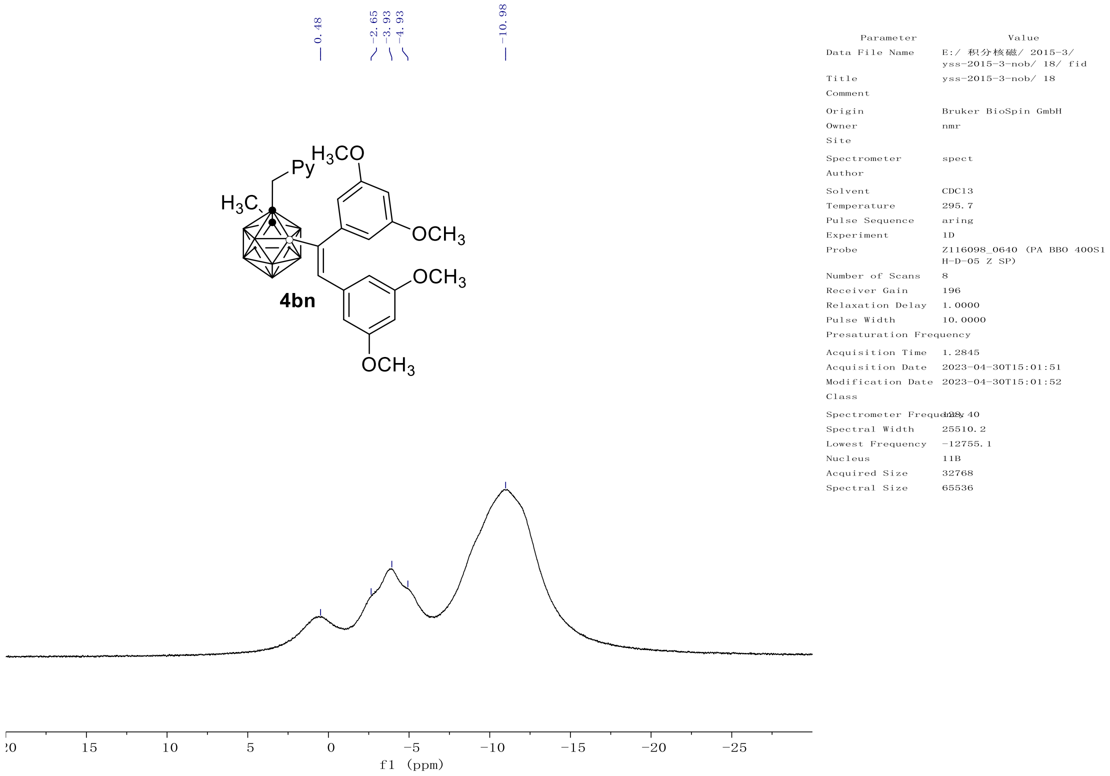

^{1}H NMR (400 MHz, $CDCl_3$) of **4bo**

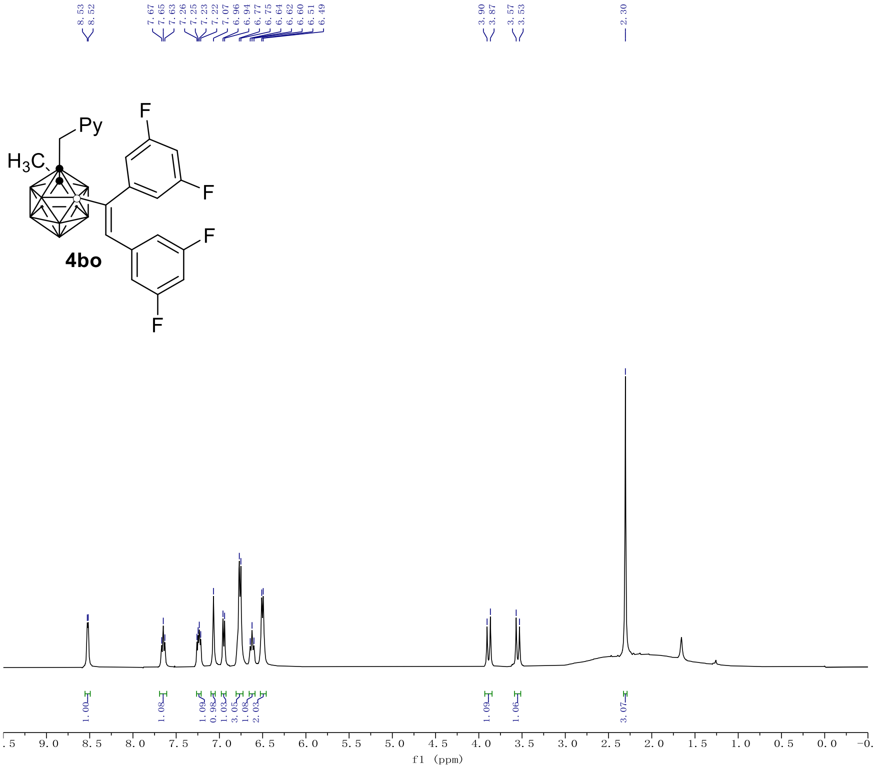

Parameter	Value
Data File Name	E:/ 积分核磁/ 2052-2/ 6-yss-2052-2-h.fid/ fid
Title	6-yss-2052-2-h
Comment	Std proton
Origin	Varian
Owner	
Site	
Spectrometer	vnmrs
Author	omc1
Solvent	CDCl3
Temperature	25.0
Pulse Sequence	s2pul
Experiment	1D
Probe	ATB
Number of Scans	8
Receiver Gain	30
Relaxation Delay	1.0000
Pulse Width	0.0000
Presaturation Frequency	
Acquisition Time	3.0000
Acquisition Date	2023-06-19T21:25:32
Modification Date	2023-06-19T13:26:00
Class	
Spectrometer Frequency	400.03
Spectral Width	10080.6
Lowest Frequency	-2032.6
Nucleus	1H
Acquired Size	30242
Spectral Size	65536

$^{13}C\{^{1}H\}$ NMR (101 MHz, $CDCl_3$) of **4bo**

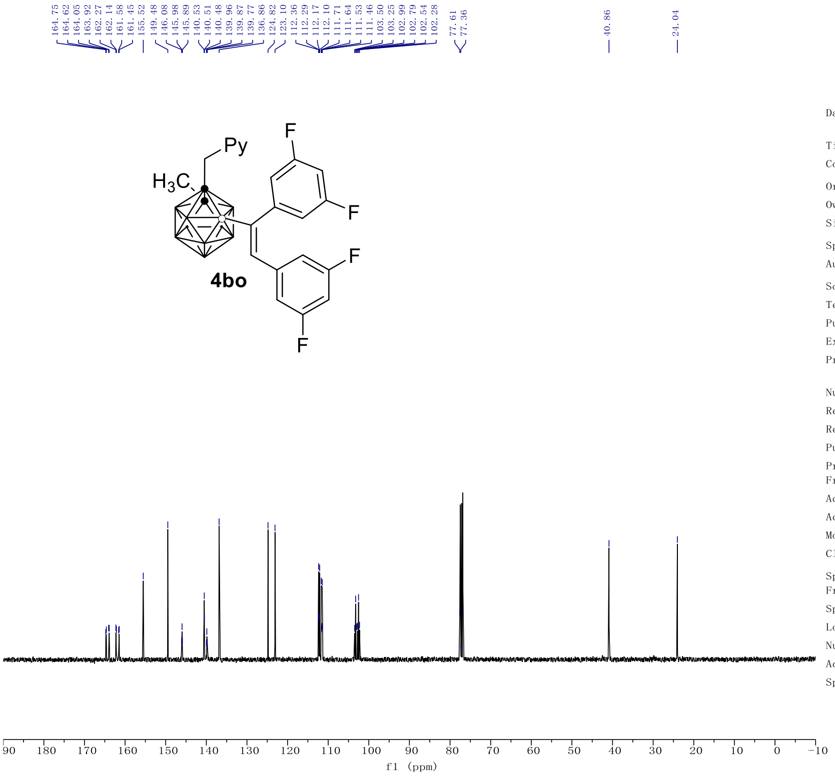

Parameter	Value
Data File Name	E:/ 积分核磁/ 2052-2/ 66-YSS-2052-2-C/ 1/ fid
Title	66-YSS-2052-2-C.1.fid
Comment	
Origin	Bruker BioSpin GmbH
Owner	nmr
Site	
Spectrometer	spect
Author	
Solvent	CDCl3
Temperature	298.1
Pulse Sequence	zgpg30
Experiment	1D
Probe	Z116098_0643 (PA BBO 400S1 BBF-H-D-05 Z SP)
Number of Scans	205
Receiver Gain	197
Relaxation Delay	1.0000
Pulse Width	10.0000
Presaturation Frequency	
Acquisition Time	1.2999
Acquisition Date	2023-06-20T22:03:48
Modification Date	2023-06-20T22:03:49
Class	
Spectrometer Frequency	100.62
Spectral Width	25252.5
Lowest Frequency	-1548.0
Nucleus	13C
Acquired Size	32827
Spectral Size	131072

$^{11}B\{^{1}H\}$ NMR (128 MHz, $CDCl_3$) of **4bo**

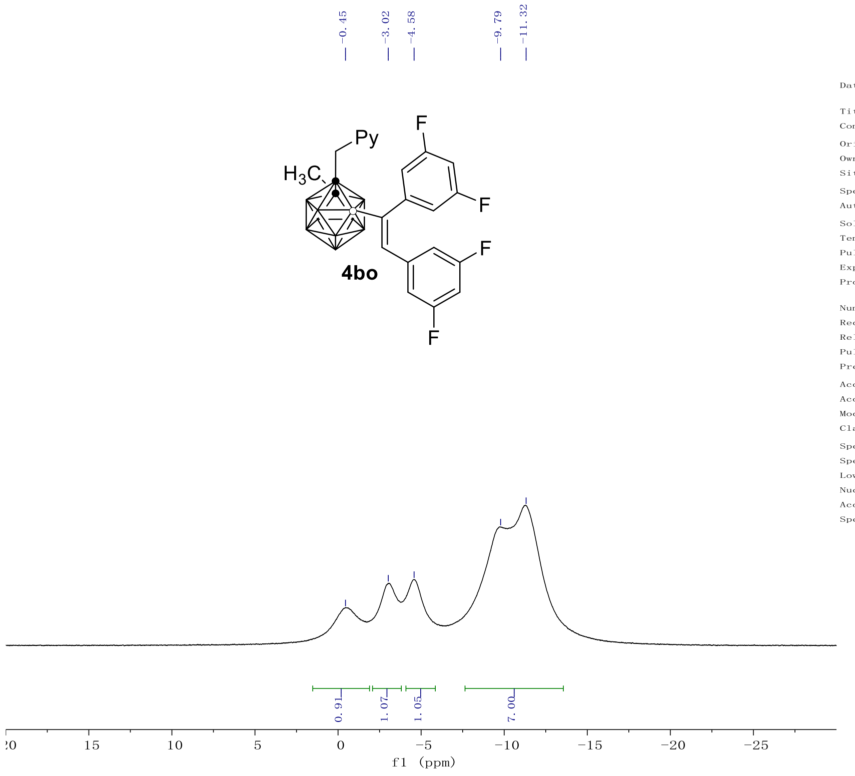

Parameter	Value
Data File Name	E:/ 积分核磁/ 2052-2/ yss-2052-2-b/ 9/ fid
Title	yss-2052-2-b/ 9
Comment	
Origin	Bruker BioSpin GmbH
Owner	nmr
Site	
Spectrometer	spect
Author	
Solvent	CDCl3
Temperature	296.1
Pulse Sequence	aring-b11
Experiment	1D
Probe	Z116098_0640 (PA BBO 400S BBF-H-D-05 Z SP)
Number of Scans	8
Receiver Gain	196
Relaxation Delay	1.0000
Pulse Width	10.0000
Presaturation Frequency	
Acquisition Time	1.2845
Acquisition Date	2023-06-17T16:23:57
Modification Date	2023-06-17T16:23:58
Class	
Spectrometer Frequency	128.40
Spectral Width	25510.2
Lowest Frequency	-12755.1
Nucleus	11B
Acquired Size	32768
Spectral Size	65536

^{11}B NMR (128 MHz, $CDCl_3$) of **4bo**

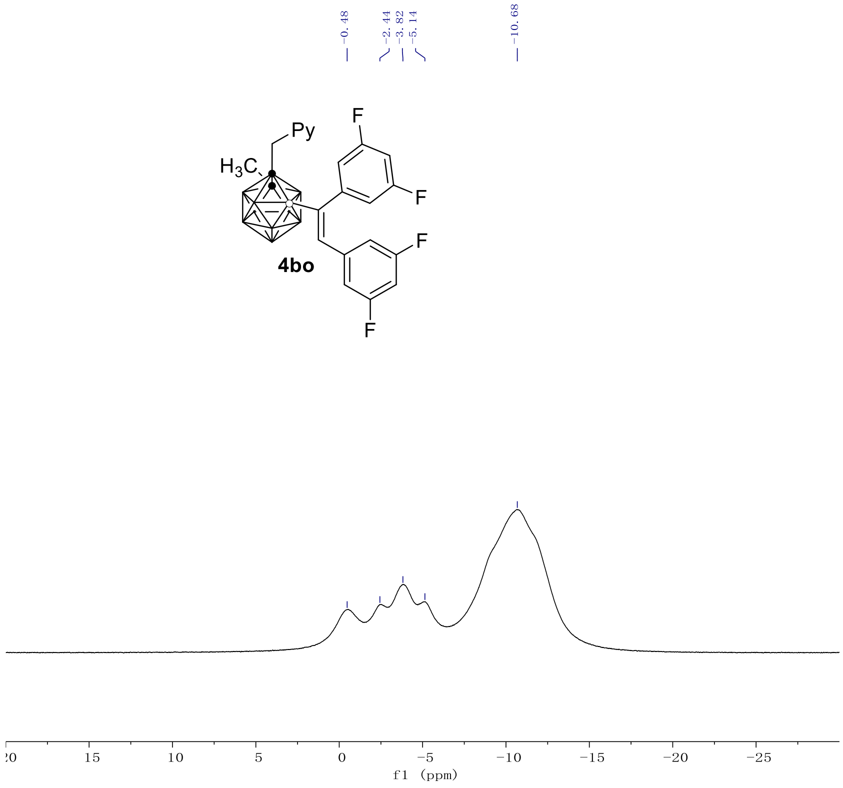

Parameter	Value
Data File Name	E:/ 积分核磁/ 2052-2/ yss-2052-2-nob/ 8/ fid
Title	yss-2052-2-nob/ 8
Comment	
Origin	Bruker BioSpin GmbH
Owner	nmr
Site	
Spectrometer	spect
Author	
Solvent	CDCl3
Temperature	296.1
Pulse Sequence	aring
Experiment	1D
Probe	Z116098_0640 (PA BBO 400 BBF-H-D-05 Z SP)
Number of Scans	15
Receiver Gain	196
Relaxation Delay	1.0000
Pulse Width	10.0000
Presaturation Frequency	
Acquisition Time	1.2845
Acquisition Date	2023-06-17T16:23:16
Modification Date	2023-06-17T16:23:17
Class	
Spectrometer Frequency	128.40
Spectral Width	25510.2
Lowest Frequency	-12755.1
Nucleus	11B
Acquired Size	32768
Spectral Size	65536

^{19}F NMR (376 MHz, $CDCl_3$) of **4bo**

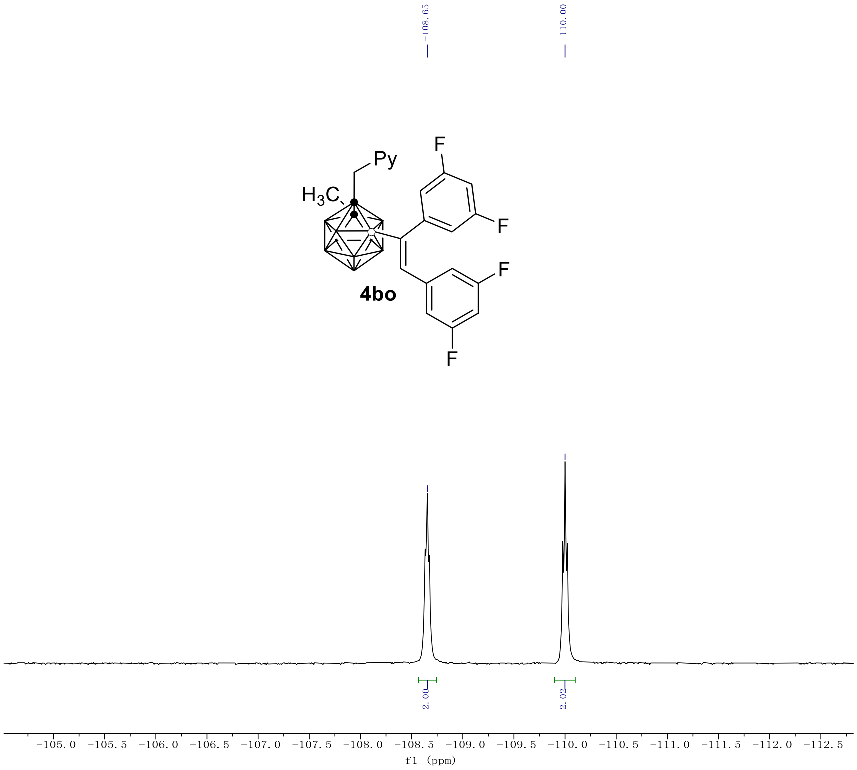

Parameter	Value
Data File Name	E:/ 积分核磁/ 2052-2/ yss-2052-2-f.fid/ fid
Title	yss-2052-2-f
Comment	Std Fluorine
Origin	Varian
Owner	
Site	
Spectrometer	vnmrs
Author	
Solvent	CDCl3
Temperature	25.0
Pulse Sequence	s2pul
Experiment	1D
Probe	ATB
Number of Scans	16
Receiver Gain	60
Relaxation Delay	1.0000
Pulse Width	0.0000
Presaturation Frequency	
Acquisition Time	1.0000
Acquisition Date	2023-06-19T21:36:52
Modification Date	2023-06-19T13:37:00
Class	
Spectrometer Frequency	376.37
Spectral Width	156250.0
Lowest Frequency	-115765.3
Nucleus	19F
Acquired Size	156250
Spectral Size	524288

^{1}H NMR (400 MHz, $CDCl_3$) of **4bq**

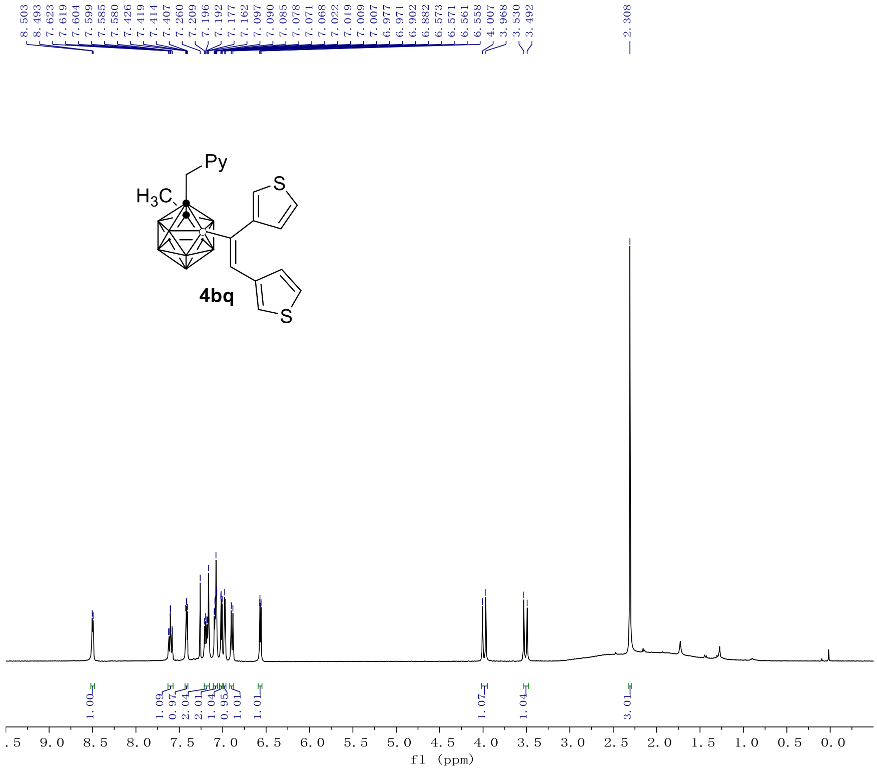

Parameter	Value
Data File Name	E:/ 积分核磁/ 2017-3/ 6yss-2017-3-h.fid/ fi
Title	6yss-2017-3-h
Comment	Std proton
Origin	Varian
Owner	
Site	
Spectrometer	vnmrs
Author	omc1
Solvent	CDCl3
Temperature	25.0
Pulse Sequence	s2pul
Experiment	1D
Probe	ATB
Number of Scans	4
Receiver Gain	24
Relaxation Delay	1.0000
Pulse Width	0.0000
Presaturation Frequency	
Acquisition Time	3.0000
Acquisition Date	2023-05-17T21:36:28
Modification Date	2023-05-17T13:37:00
Class	
Spectrometer Frequency	400.03
Spectral Width	10080.6
Lowest Frequency	2033.3
Nucleus	1H
Acquired Size	30242
Spectral Size	65536

^{13}C{^{1}H} NMR (101 MHz, $CDCl_3$) of **4bq**

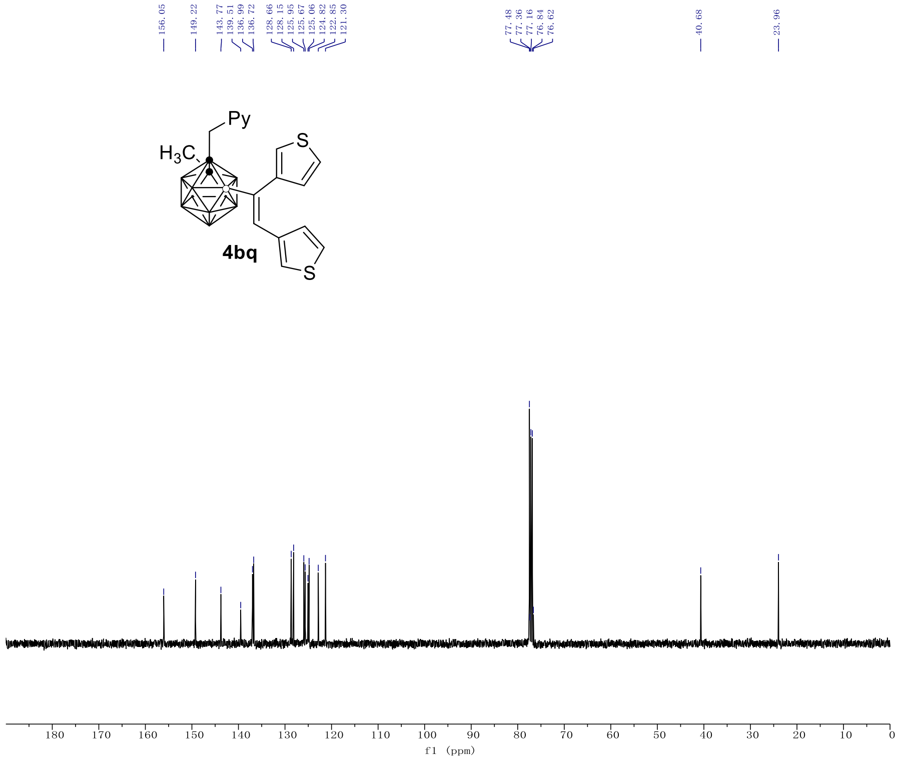

Parameter	Value
Data File Name	E:/ 积分核磁/ 2017-3/ yss-2017-3-c.fid/ fid
Title	yss-2017-3-c
Comment	Std carbon
Origin	Varian
Owner	
Site	
Spectrometer	vnmrs
Author	omc1
Solvent	cdcl3
Temperature	25.0
Pulse Sequence	s2pul
Experiment	1D
Probe	ATB
Number of Scans	120
Receiver Gain	60
Relaxation Delay	1.0000
Pulse Width	0.0000
Presaturation Frequency	
Acquisition Time	1.3000
Acquisition Date	2023-05-17T21:37:45
Modification Date	2023-05-17T13:42:00
Class	
Spectrometer Frequency	100.60
Spectral Width	28409.1
Lowest Frequency	-2122.3
Nucleus	13C
Acquired Size	36932
Spectral Size	131072

$^{11}B\{^1H\}$ NMR (128 MHz, $CDCl_3$) of **4bq**

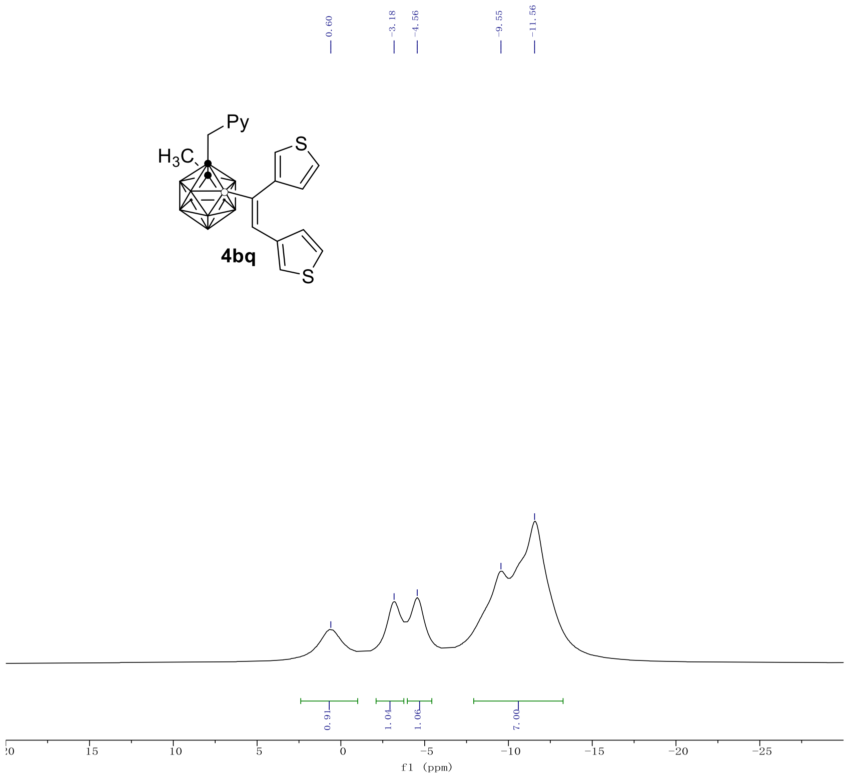

Parameter	Value
Data File Name	E:/ 积分核磁/ 2017-3/ yss-2017-3-b/ 6/ fid
Title	yss-2017-3-b/ 6
Comment	
Origin	Bruker BioSpin GmbH
Owner	nmr
Site	
Spectrometer	spect
Author	
Solvent	CDCl3
Temperature	295.9
Pulse Sequence	aring-b11
Experiment	1D
Probe	Z116098_0640 (PA BBO 400S1 BBF-H-D-05 Z SP)
Number of Scans	23
Receiver Gain	196
Relaxation Delay	1.0000
Pulse Width	10.0000
Presaturation Frequency	
Acquisition Time	1.2845
Acquisition Date	2023-05-17T21:56:57
Modification Date	2023-05-17T21:56:58
Class	
Spectrometer Frequency	128.40
Spectral Width	25510.2
Lowest Frequency	-12755.1
Nucleus	11B
Acquired Size	32768
Spectral Size	65536

^{11}B NMR (128 MHz, $CDCl_3$) of **4bq**

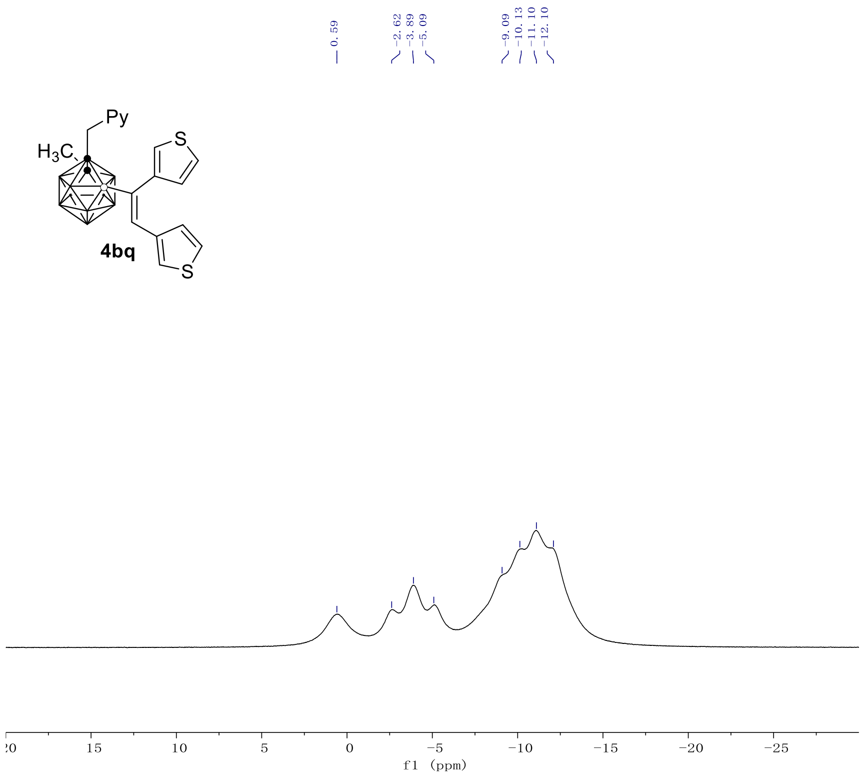

Parameter	Value
Data File Name	E:/ 积分核磁/ 2017-3/ yss-2017-3-nob/ 7/ fid
Title	yss-2017-3-nob/ 7
Comment	
Origin	Bruker BioSpin GmbH
Owner	nmr
Site	
Spectrometer	spect
Author	
Solvent	CDCl3
Temperature	295.9
Pulse Sequence	aring
Experiment	1D
Probe	Z116098_0640 (PA BBO 4(BBF-H-D-05 Z SP)
Number of Scans	24
Receiver Gain	196
Relaxation Delay	1.0000
Pulse Width	10.0000
Presaturation Frequency	
Acquisition Time	1.2845
Acquisition Date	2023-05-17T21:58:16
Modification Dat(	2023-05-17T21:58:17
Class	
Spectrometer Frequency	128.40
Spectral Width	25510.2
Lowest Frequency	-12755.1
Nucleus	11B
Acquired Size	32768
Spectral Size	65536

^{1}H NMR (400 MHz, $CDCl_3$) of **4br**

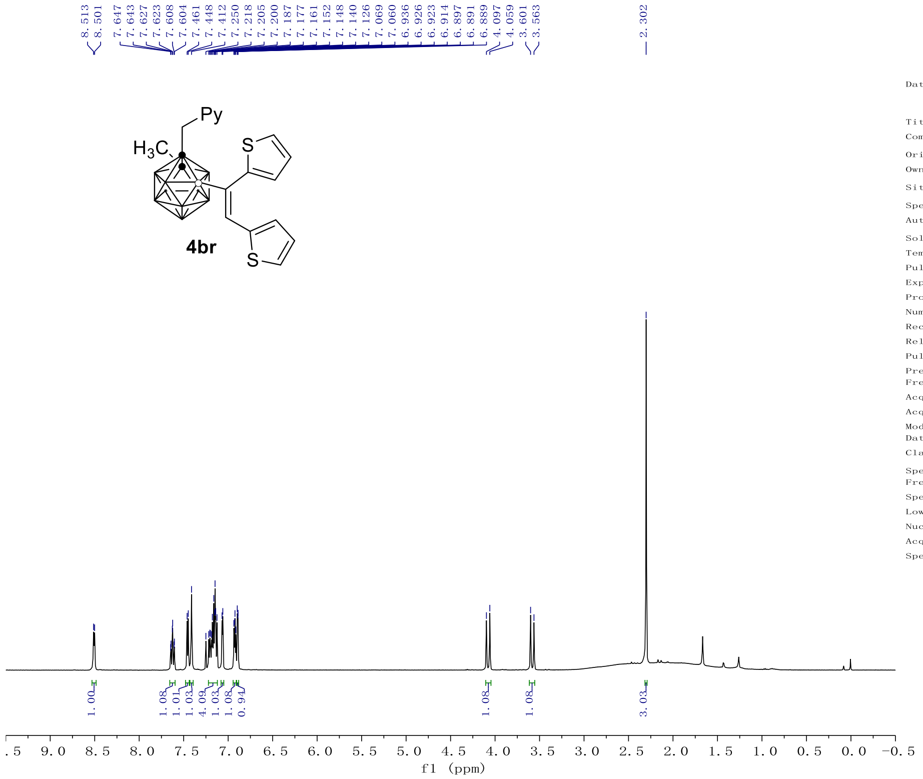

Parameter	Value
Data File Name	E:/ 积分核磁/ 2017-4 6-yss-2017-4-h.fid/ fid
Title	6-yss-2017-4-h
Comment	
Origin	Varian
Owner	
Site	
Spectrometer	vnmrs
Author	
Solvent	CDCl3
Temperature	25.0
Pulse Sequence	s2pul
Experiment	1D
Probe	4nuc
Number of Scans	8
Receiver Gain	36
Relaxation Delay	1.0000
Pulse Width	0.0000
Presaturation Frequency	
Acquisition Time	[illegible]9999
Acquisition Date	2023-05-19T20:52:57
Modification Date	2023-05-19T12:53:00
Class	
Spectrometer Frequency	399.72
Spectral Width	8012.8
Lowest Frequency	-808.7
Nucleus	1H
Acquired Size	24038
Spectral Size	65536

$^{13}C\{^1H\}$ NMR (101 MHz, $CDCl_3$) of **4br**

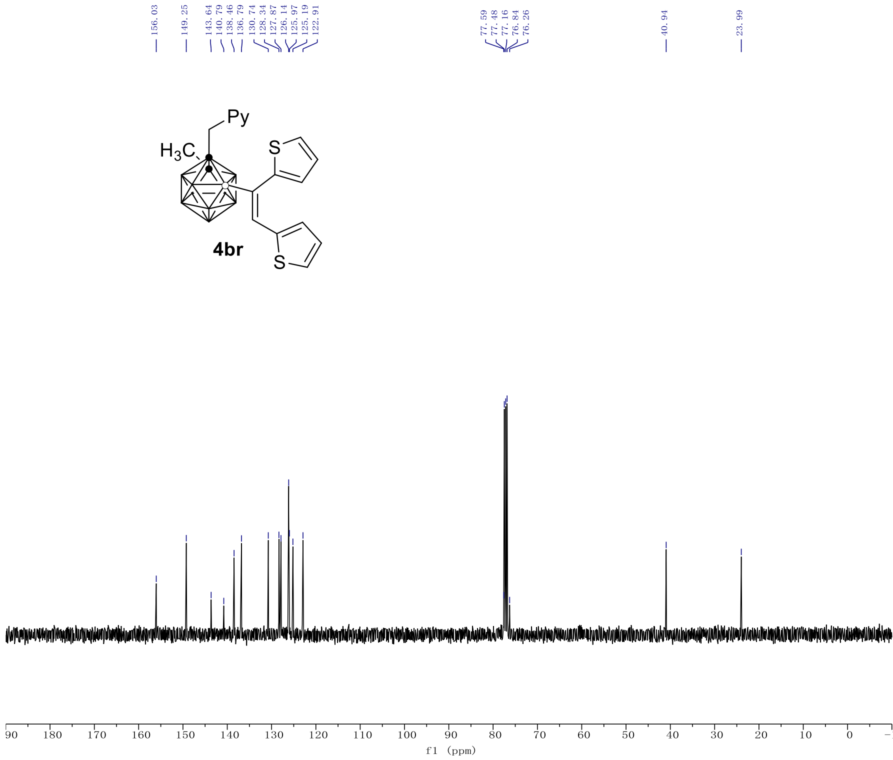

Parameter	Value
Data File Name	E:/ 积分核磁/ 2017-4/ yss-2017-4-c/ 16/ fid
Title	yss-2017-4-c/ 16
Comment	
Origin	Bruker BioSpin GmbH
Owner	nmr
Site	
Spectrometer	spect
Author	
Solvent	CDCl3
Temperature	296.3
Pulse Sequence	zgpg30
Experiment	1D
Probe	Z116098_0640 (PA BBO 400S1 BBF-H-D-05 Z SP)
Number of Scans	26
Receiver Gain	196
Relaxation Delay	1.0000
Pulse Width	10.0000
Presaturation Frequency	
Acquisition Time	1.2999
Acquisition Date	2023-05-03T15:59:21
Modification Date	2023-05-03T15:59:22
Class	
Spectrometer Frequency	100.64
Spectral Width	25252.5
Lowest Frequency	-1549.5
Nucleus	13C
Acquired Size	32827

$^{11}B\{^{1}H\}$ NMR (128 MHz, $CDCl_3$) of **4br**

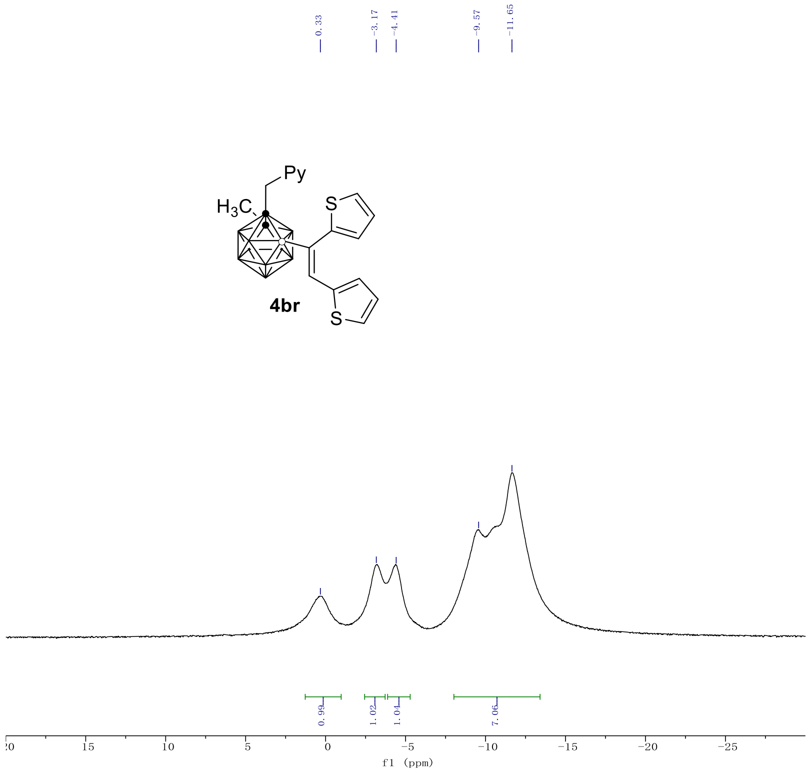

Parameter	Value
Data File Name	E:/ 积分核磁/ 2017-4/ yss-2017-4-b/ 17/ fid
Title	yss-2017-4-b/ 17
Comment	
Origin	Bruker BioSpin GmbH
Owner	nmr
Site	
Spectrometer	spect
Author	
Solvent	CDCl3
Temperature	296.1
Pulse Sequence	aring-b11
Experiment	1D
Probe	Z116098_0640 (PA BBO 400S1 BBF-H-D-05 Z SP)
Number of Scans	8
Receiver Gain	196
Relaxation Delay	1.0000
Pulse Width	10.0000
Presaturation Frequency	
Acquisition Time	1.2845
Acquisition Date	2023-05-03T16:01:59
Modification Date	2023-05-03T16:02:00
Class	
Spectrometer Frequency	128.40
Spectral Width	25510.2
Lowest Frequency	-12755.1
Nucleus	11B
Acquired Size	32768
Spectral Size	65536

^{11}B NMR (128 MHz, $CDCl_3$) of **4br**

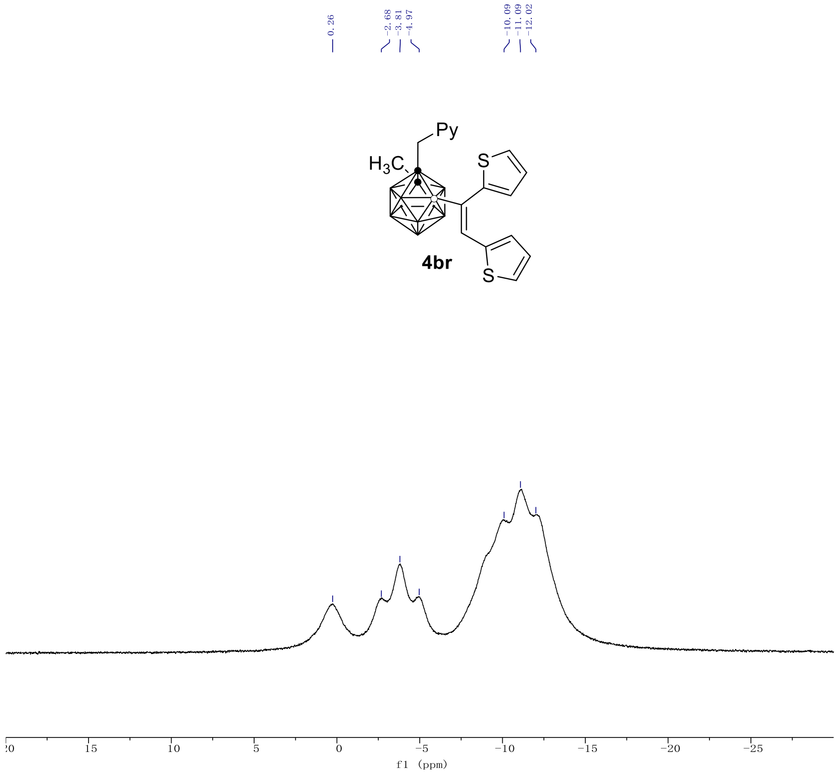

Parameter	Value
Data File Name	E:/ 积分核磁/ 2017-4/ yss-2017-4-nob/ 18/ fid
Title	yss-2017-4-nob/ 18
Comment	
Origin	Bruker BioSpin GmbH
Owner	nmr
Site	
Spectrometer	spect
Author	
Solvent	CDCl3
Temperature	296.0
Pulse Sequence	aring
Experiment	1D
Probe	Z116098_0640 (PA BBO 400S1 BBF-H-D-05 Z SP)
Number of Scans	5
Receiver Gain	196
Relaxation Delay	1.0000
Pulse Width	10.0000
Presaturation Frequency	
Acquisition Time	1.2845
Acquisition Date	2023-05-03T16:03:45
Modification Date	2023-05-03T16:03:46
Class	
Spectrometer Frequency	128.40
Spectral Width	25510.2
Lowest Frequency	-12755.1
Nucleus	11B
Acquired Size	32768
Spectral Size	65536

^{1}H NMR (400 MHz, $CDCl_3$) of **4bs**

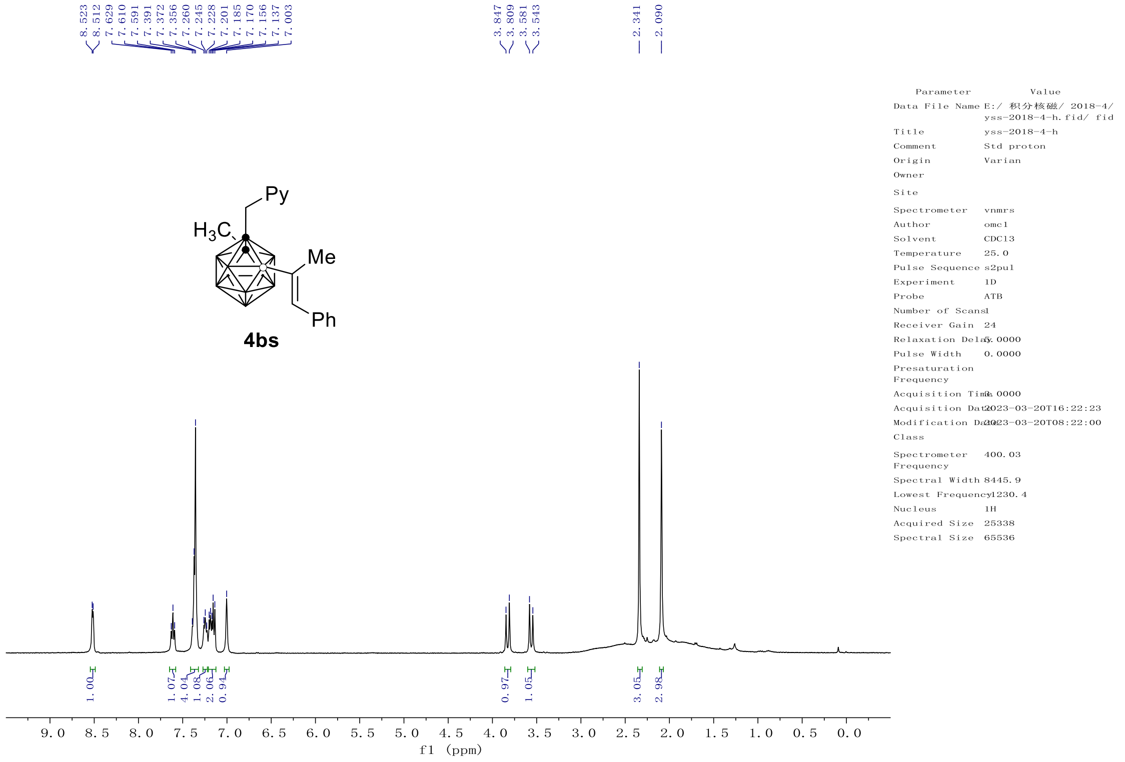

$^{13}C\{^{1}H\}$ NMR (101 MHz, $CDCl_3$) of **4bs**

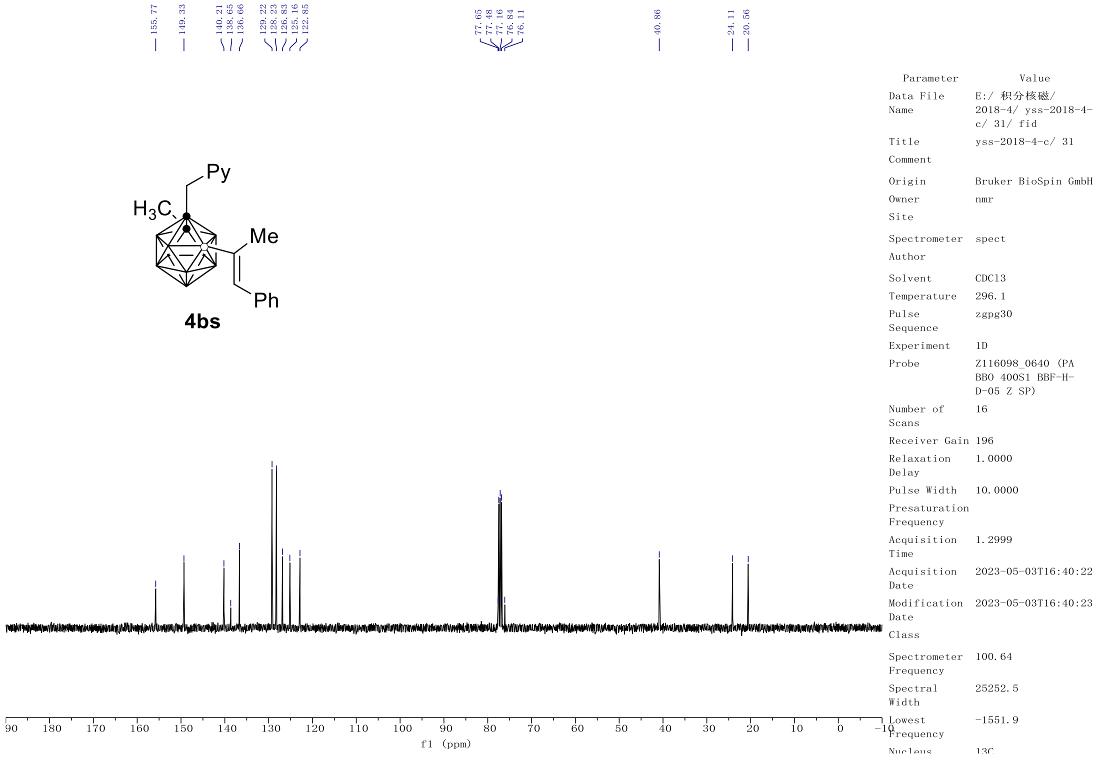

$^{11}B\{^{1}H\}$ NMR (128 MHz, $CDCl_3$) of **4bs**

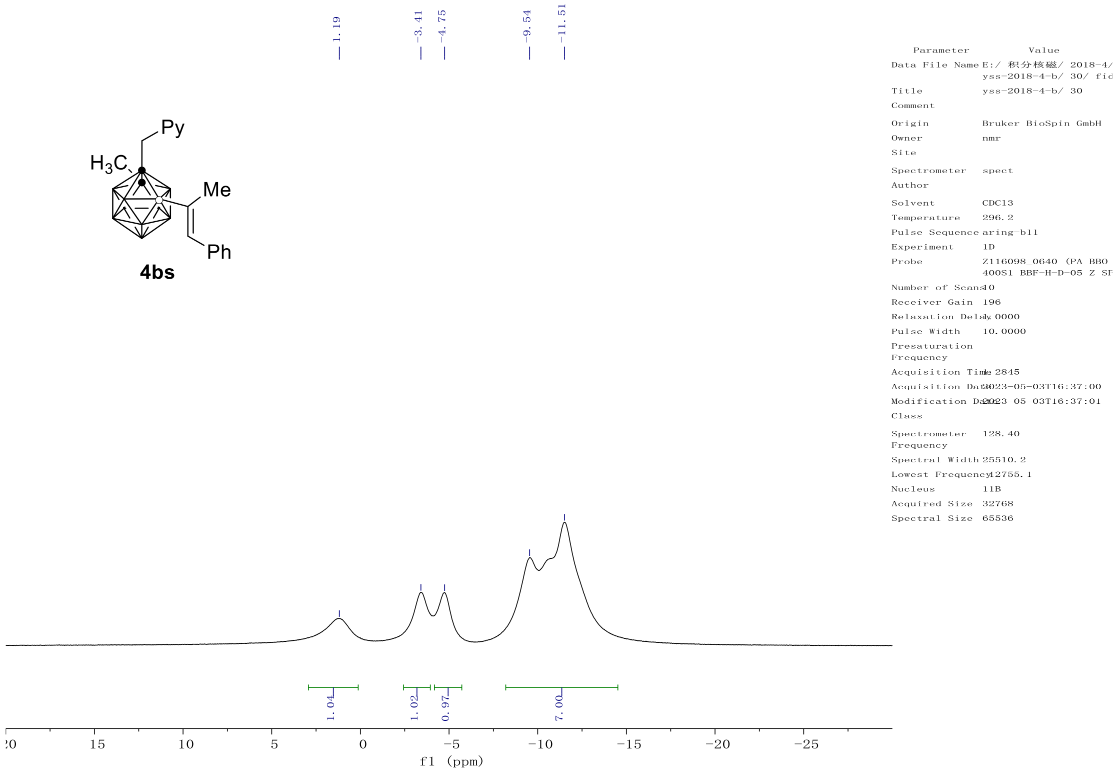

^{11}B NMR (128 MHz, $CDCl_3$) of **4bs**

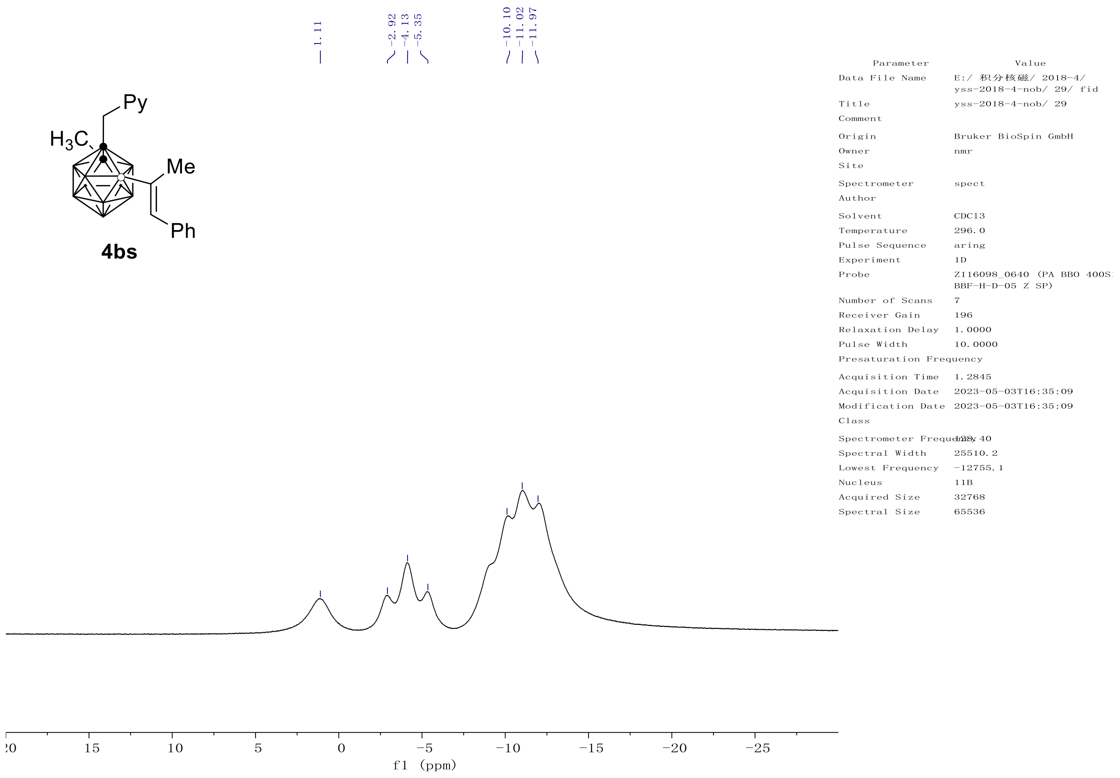

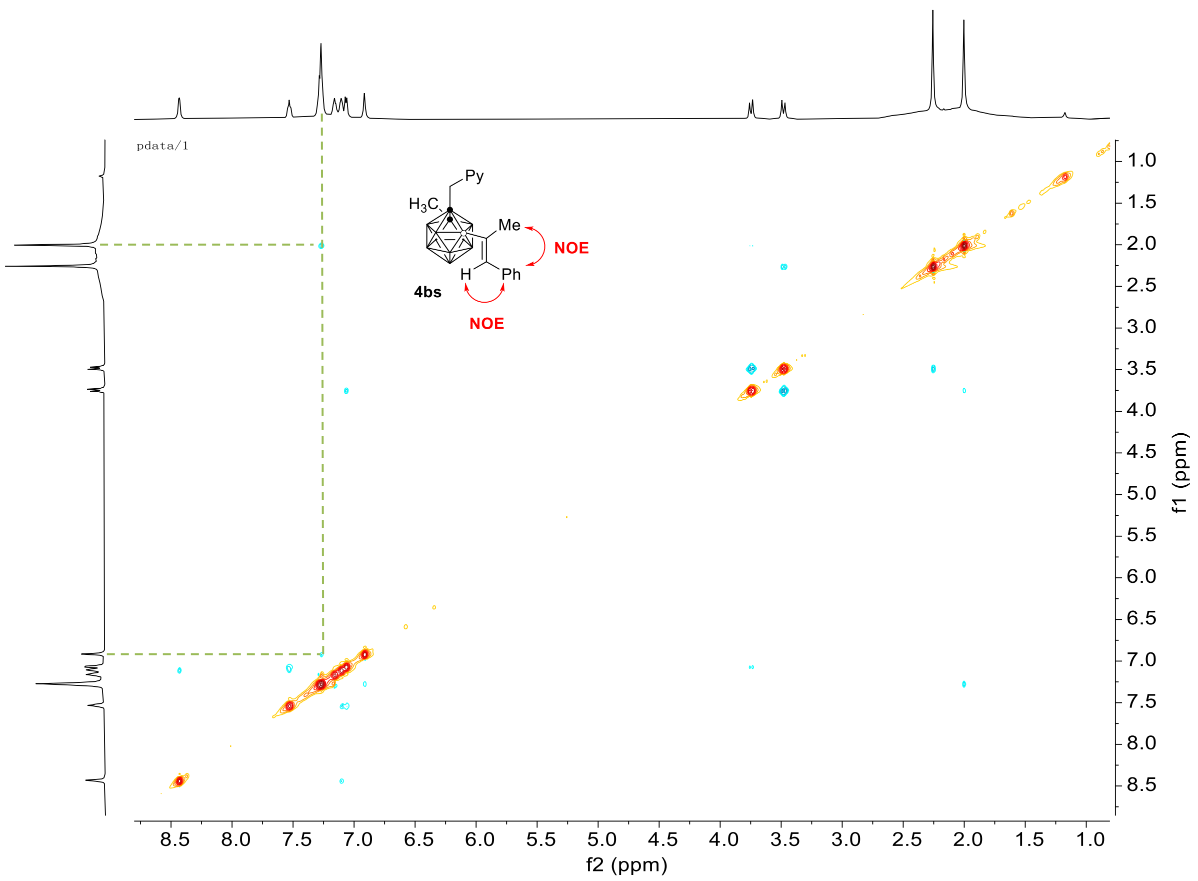
pdata/1
Py
H3C
Me
NOE
H
Ph
4bs
NOE
f2 (ppm)
f1 (ppm)

^{1}H NMR (400 MHz, $CDCl_3$) of **4bt**

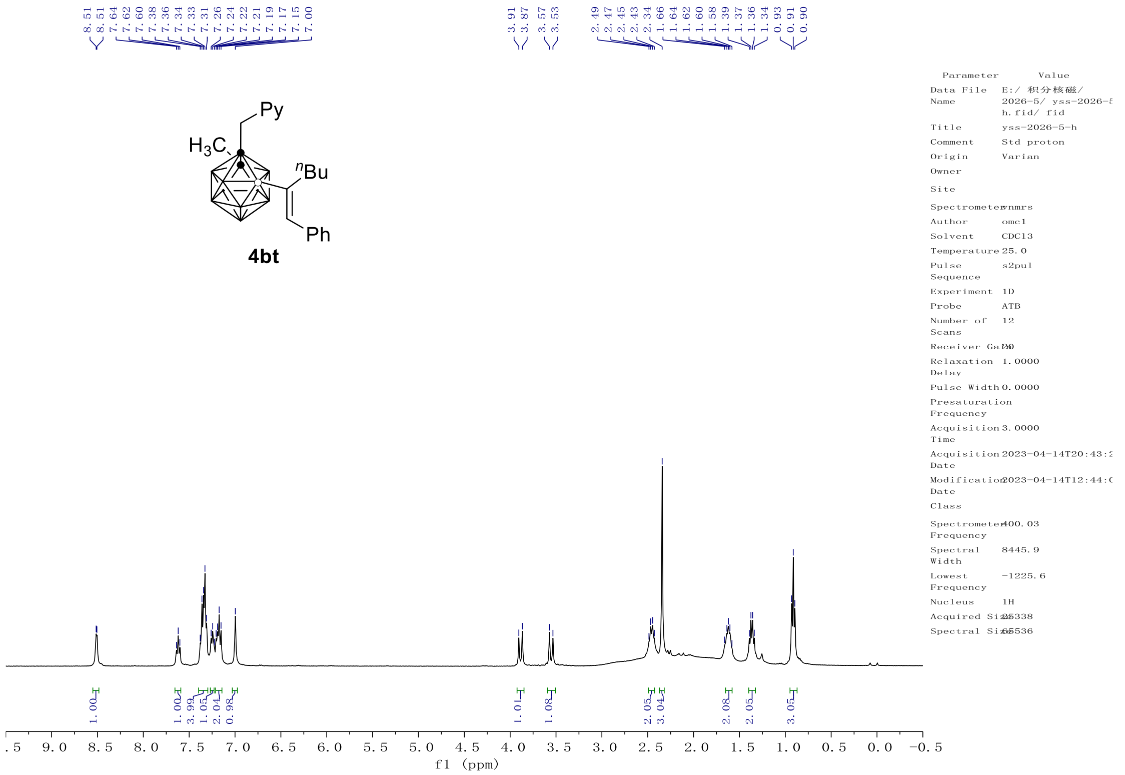

$^{13}C\{^{1}H\}$ NMR (101 MHz, $CDCl_3$) of **4bt**

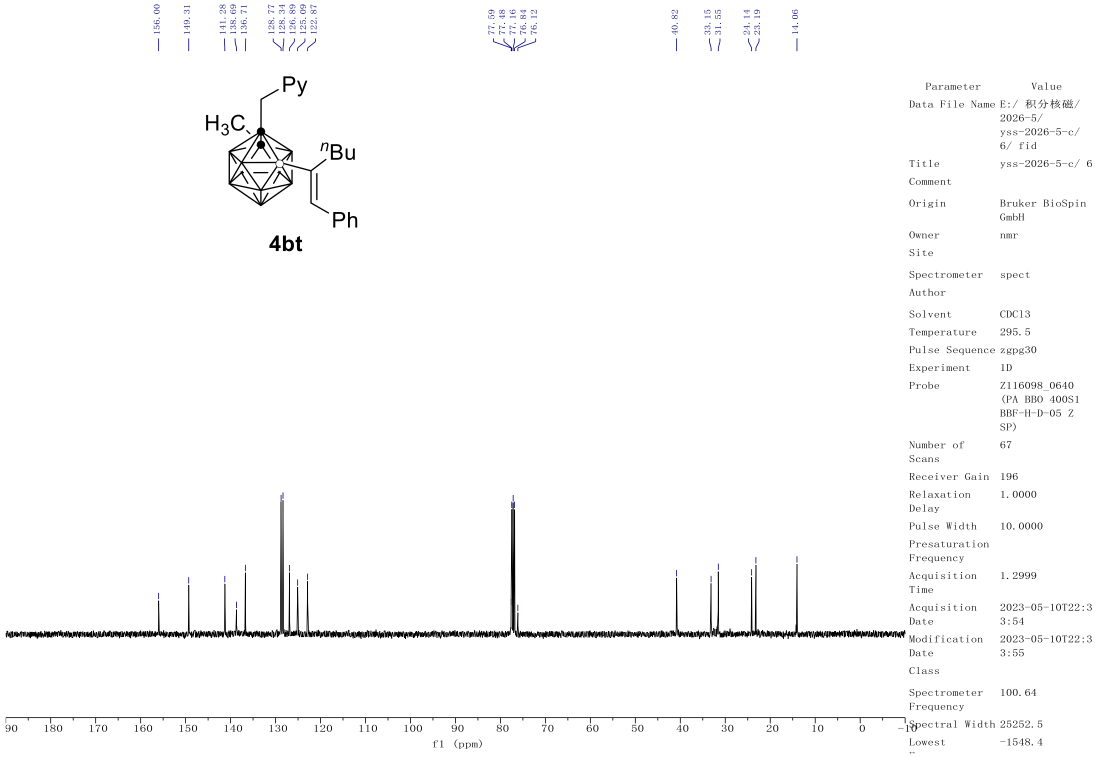

$^{11}B\{^{1}H\}$ NMR (128 MHz, $CDCl_3$) of **4bt**

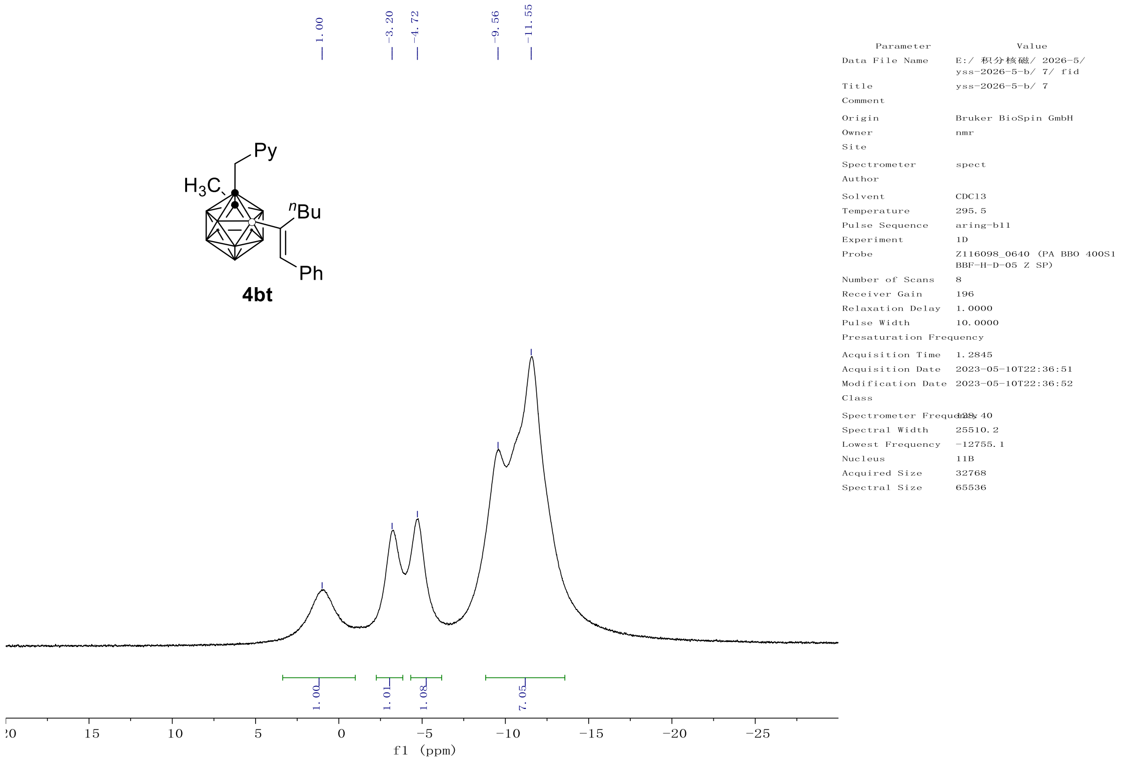

^{11}B NMR (128 MHz, $CDCl_3$) of **4bt**

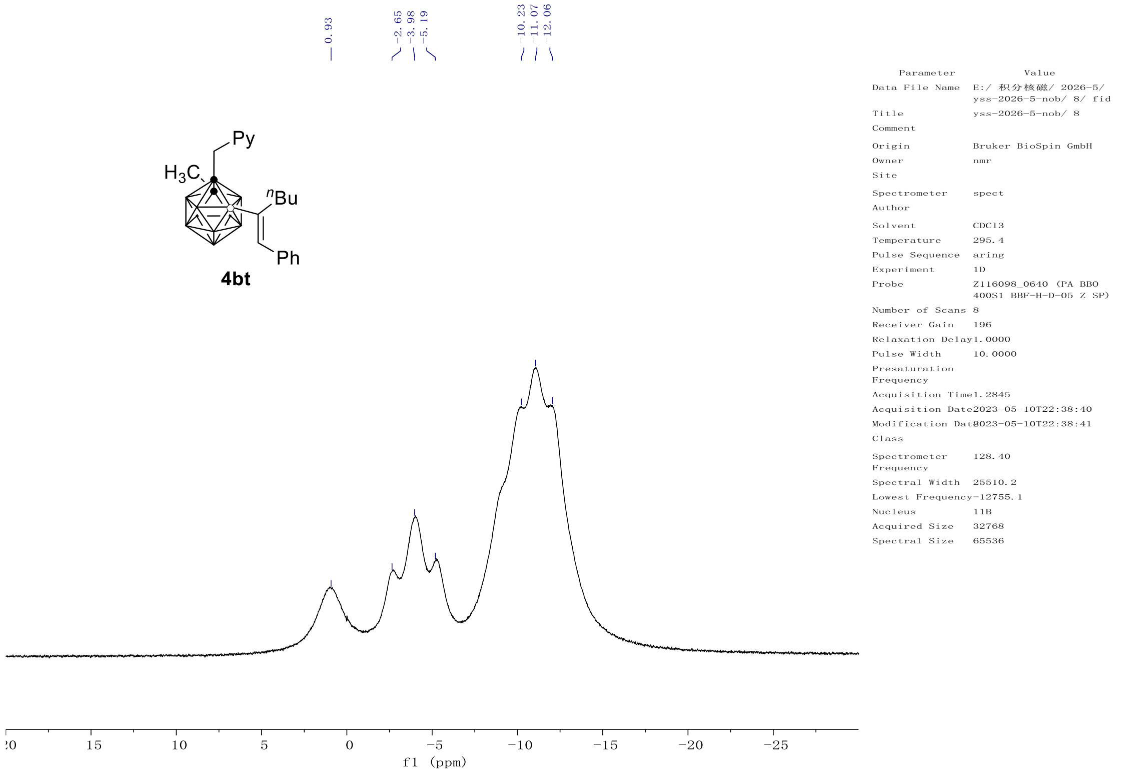

^{1}H NMR (400 MHz, $CDCl_3$) of **4bu**

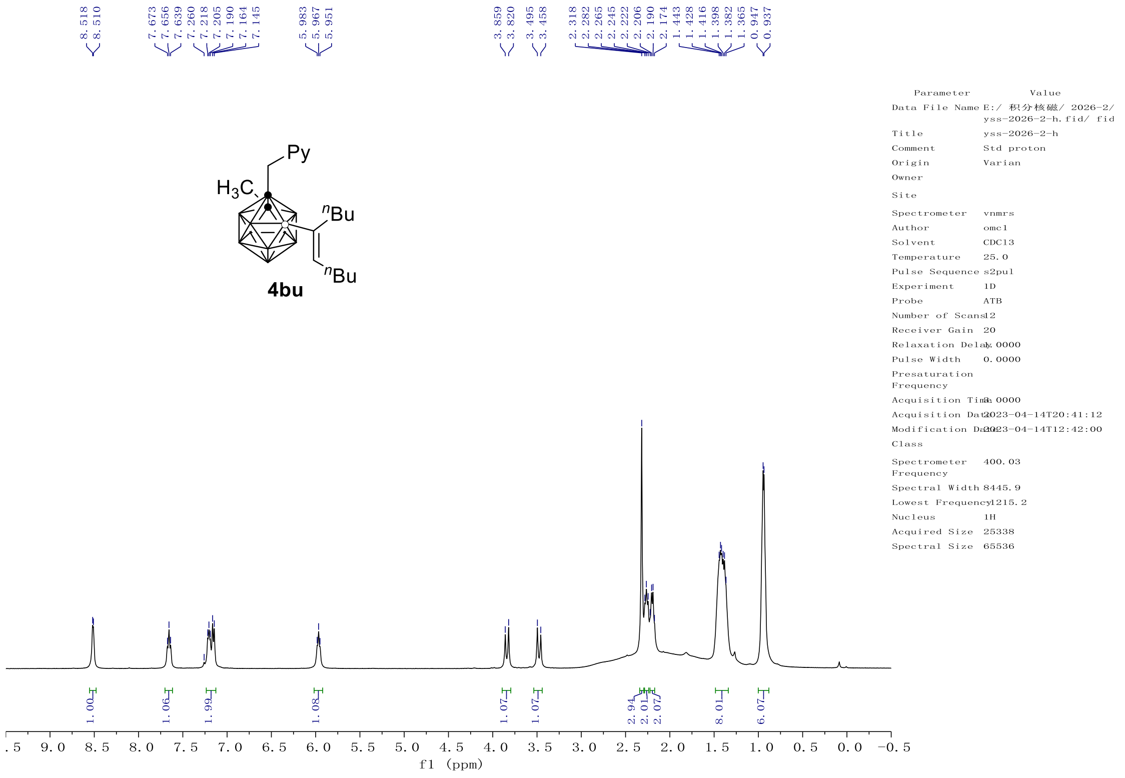

$^{13}C\{^{1}H\}$ NMR (101 MHz, $CDCl_3$) of **4bu**

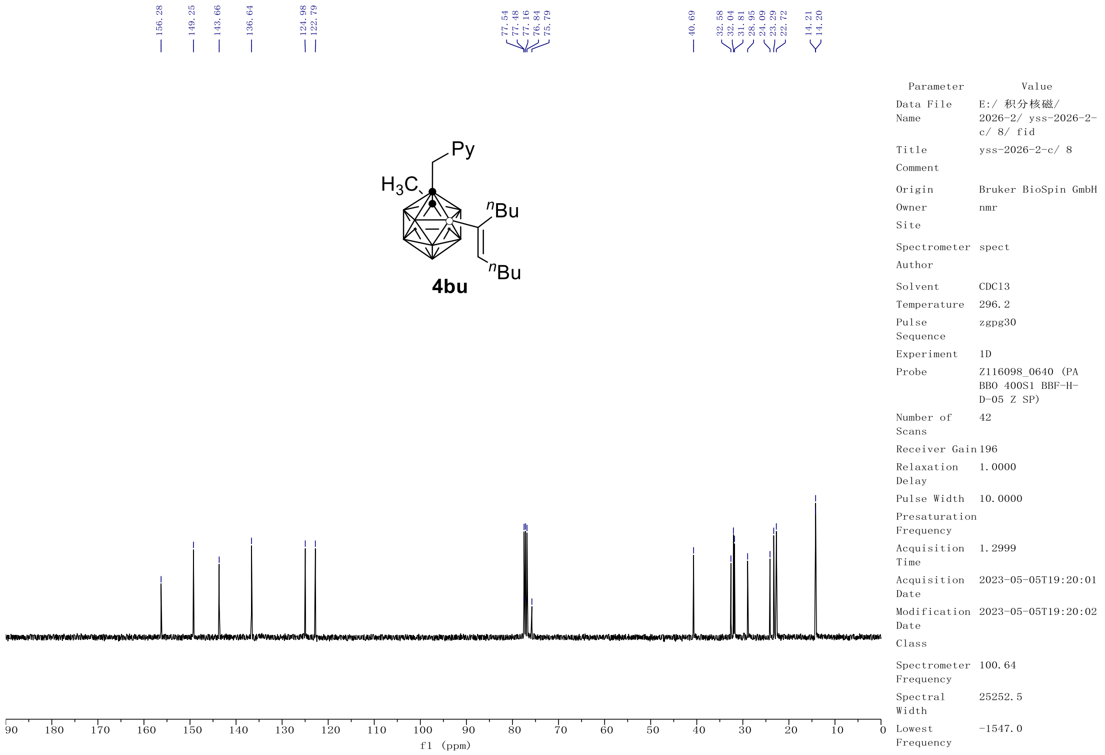

$^{11}B\{^{1}H\}$ NMR (128 MHz, $CDCl_3$) of **4bu**

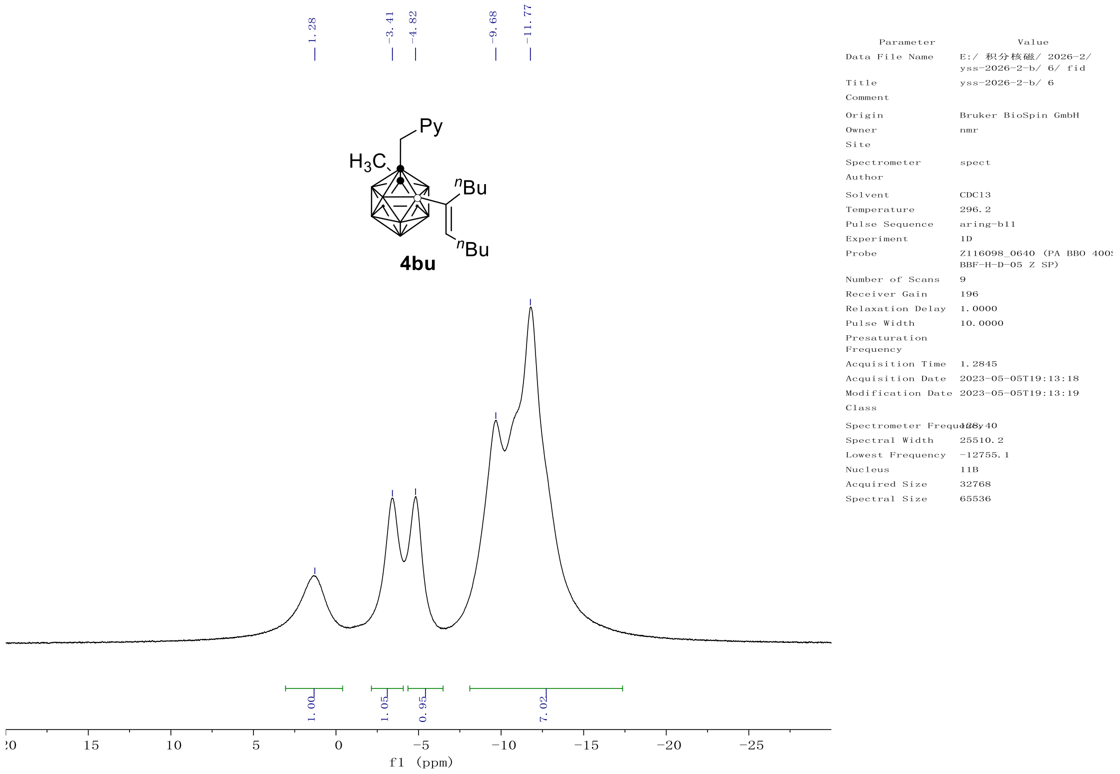

^{11}B NMR (128 MHz, $CDCl_3$) of **4bu**

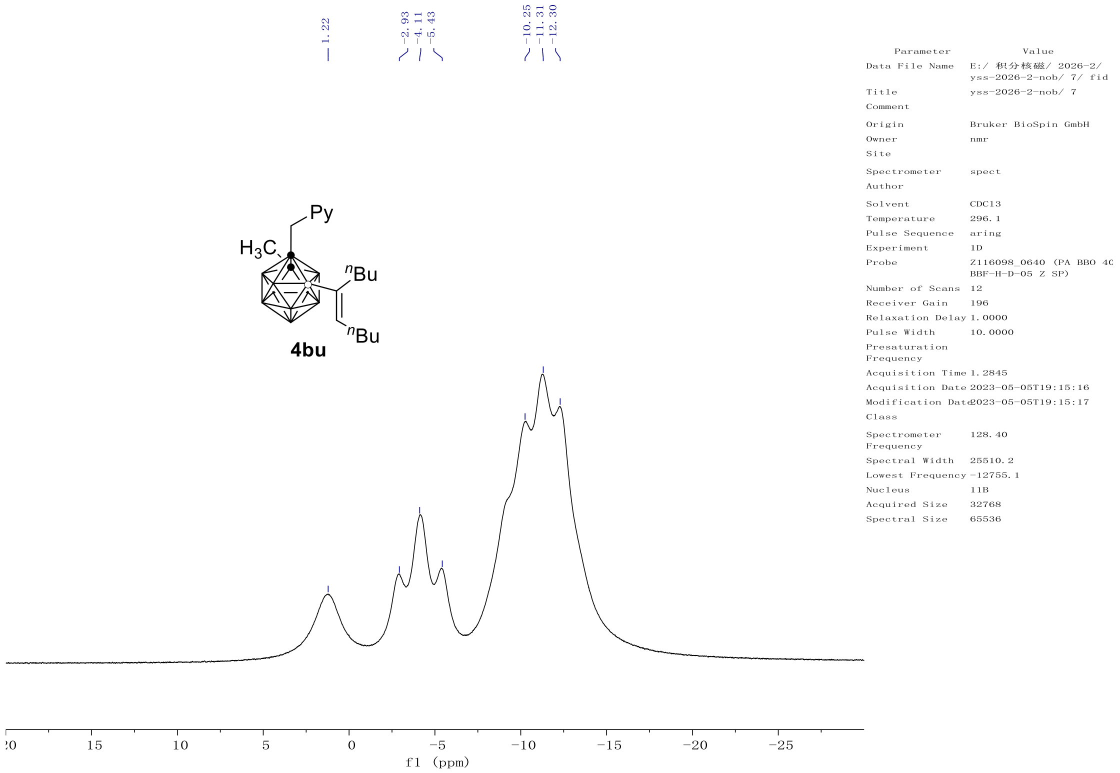

^1H NMR (400 MHz, $CDCl_3$) of **4ca**

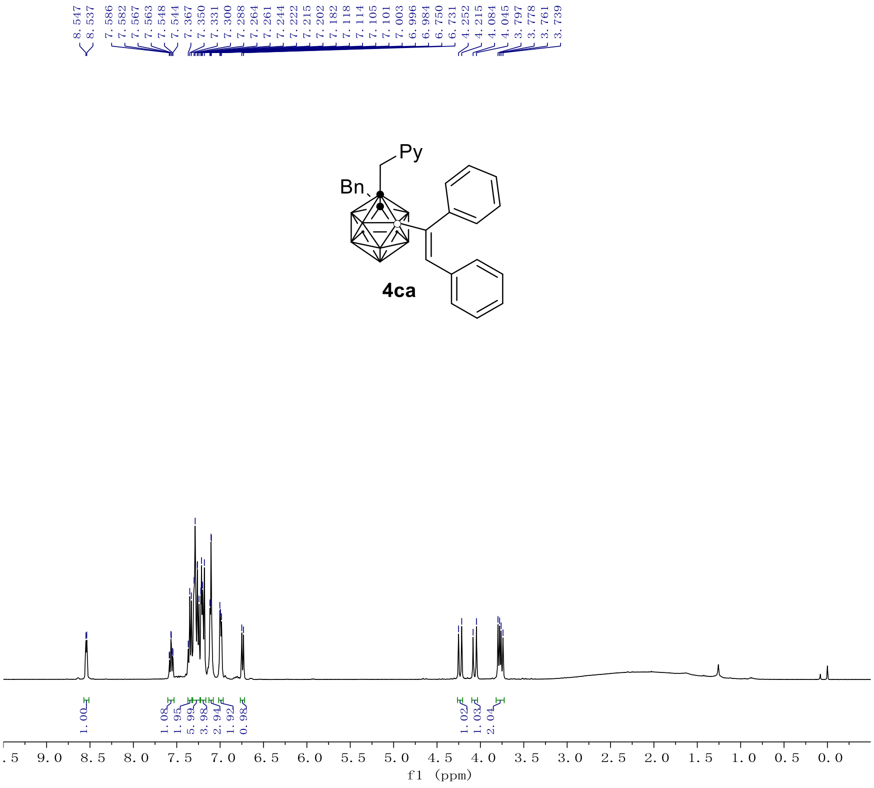

Parameter	Value
Data File Name	E:/ 核磁/ yss-230912-1.fid/ fid
Title	yss-230912-1
Comment	
Origin	Varian
Owner	
Site	
Spectrometer	vnmrs
Author	
Solvent	CDCl3
Temperature	25.0
Pulse Sequence	s2pul
Experiment	1D
Probe	4nuc
Number of Scans	4
Receiver Gain	34
Relaxation Delay	1.0000
Pulse Width	0.0000
Presaturation Frequency	
Acquisition Time	3.0000
Acquisition Date	2023-09-12T18:32:17
Modification Date	2023-09-12T10:32:00
Class	
Spectrometer Frequency	399.72
Spectral Width	8389.3
Lowest Frequency	-1210.4
Nucleus	1H
Acquired Size	25168
Spectral Size	65536

$^{13}C\{^1H\}$ NMR (101 MHz, $CDCl_3$) of **4ca**

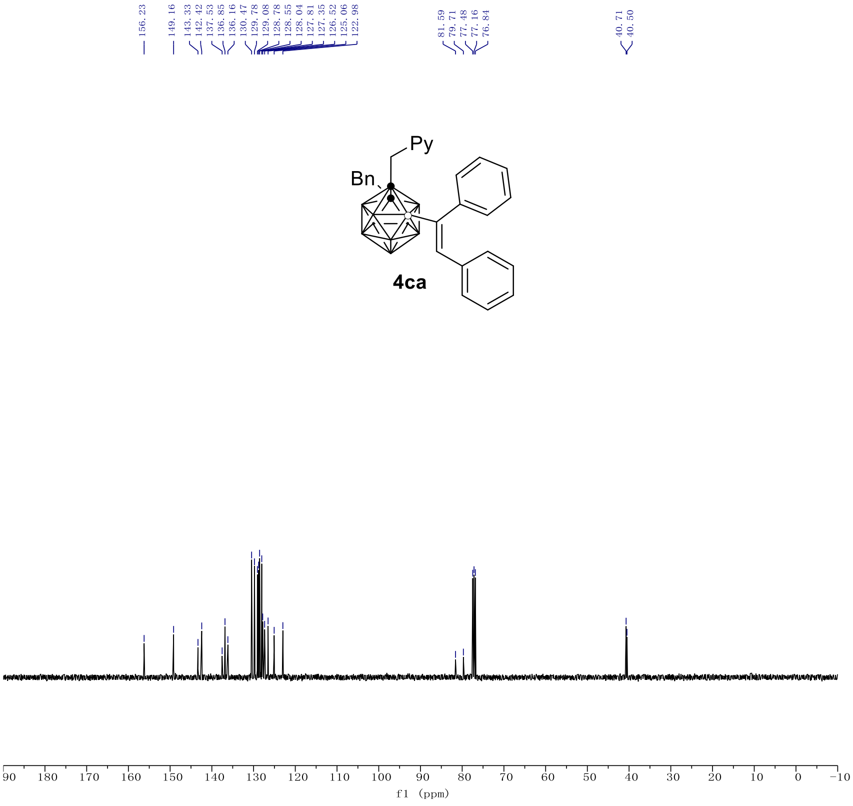

Parameter	Value
Data File Name	E:/ 积分核磁/ 1054/ yss-1054-c/ 1/ fid
Title	yss-1054-c.1.fid
Comment	
Origin	Bruker BioSpin GmbH
Owner	nmr
Site	
Spectrometer	spect
Author	
Solvent	CDCl3
Temperature	298.2
Pulse Sequence	zgpg30
Experiment	1D
Probe	Z116098_0643 (PA BBO 400S1 BBF-H-D-05 Z SP)
Number of Scans	33
Receiver Gain	197
Relaxation Delay	1.0000
Pulse Width	10.0000
Presaturation Frequency	
Acquisition Time	1.2999
Acquisition Date	2023-09-12T18:33:36
Modification Date	2023-09-12T18:33:37
Class	
Spectrometer Frequency	100.62
Spectral Width	25252.5
Lowest Frequency	-1552.9
Nucleus	13C
Acquired Size	32827
Spectral Size	131072

$^{11}B\{^{1}H\}$ NMR (128 MHz, $CDCl_3$) of **4ca**

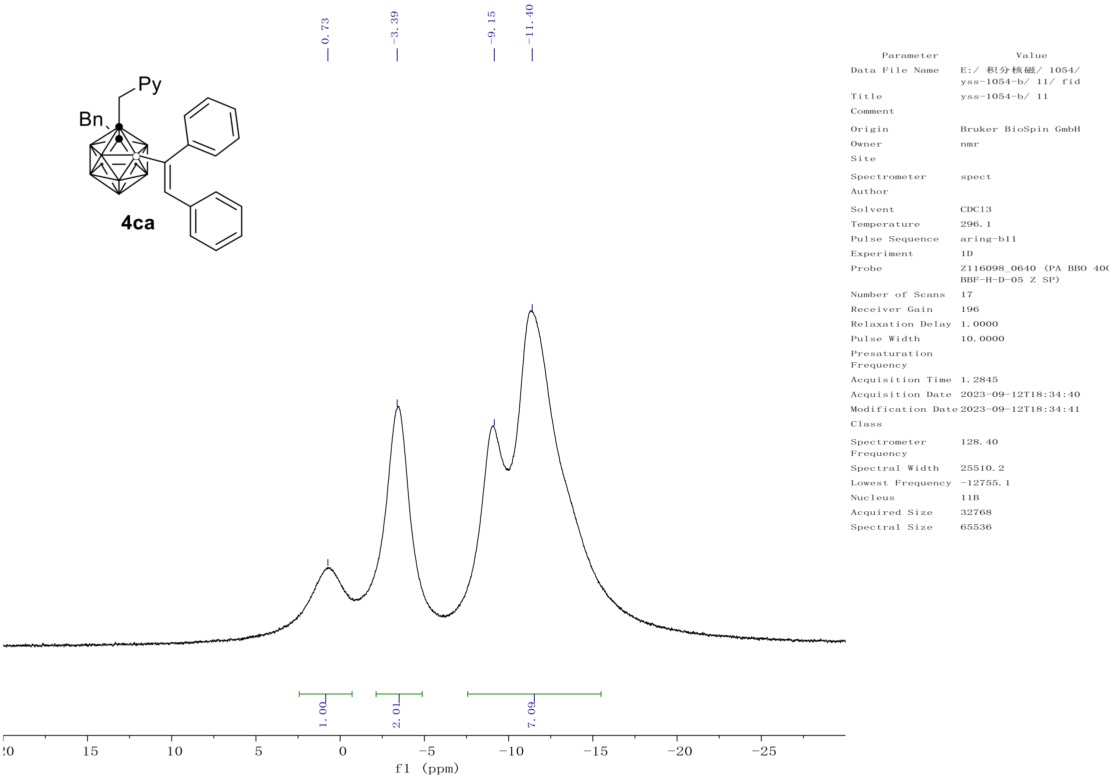

^{11}B NMR (128 MHz, $CDCl_3$) of **4ca**

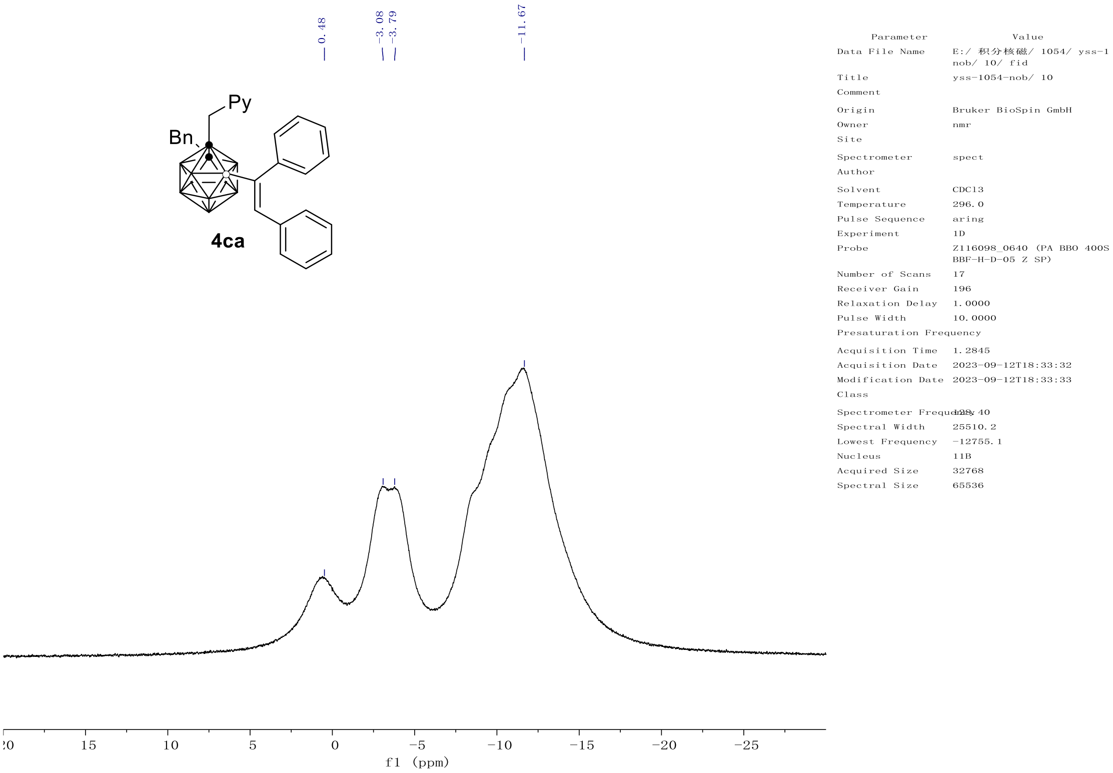

^{1}H NMR (400 MHz, $CDCl_3$) of **4da**

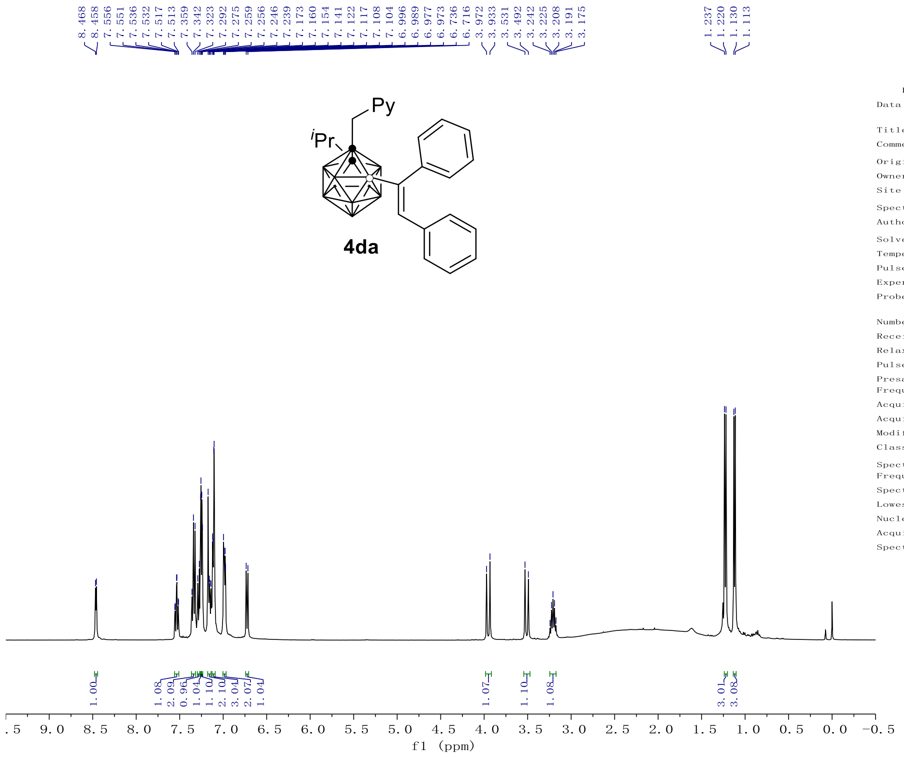

Parameter	Value
Data File Name	E:/ 核磁/ yss-1051-h/ fid
Title	yss-1051-h/ 6
Comment	
Origin	Bruker BioSpin GmbH
Owner	nmr
Site	
Spectrometer	spect
Author	
Solvent	CDCl3
Temperature	295.9
Pulse Sequence	zg30
Experiment	1D
Probe	Z116098_0640 (PA BBO 400S1 BBF-H-D-05 Z SP)
Number of Scans	9
Receiver Gain	51
Relaxation Delay	1.0000
Pulse Width	10.0000
Presaturation Frequency	
Acquisition Time	2.9998
Acquisition Date	2022-11-09T19:07:57
Modification Date	2022-11-09T19:07:57
Class	
Spectrometer Frequency	400.20
Spectral Width	7598.8
Lowest Frequency	-1213.3
Nucleus	1H
Acquired Size	22795
Spectral Size	65536

$^{13}C\{^{1}H\}$ NMR (101 MHz, $CDCl_3$) of **4da**

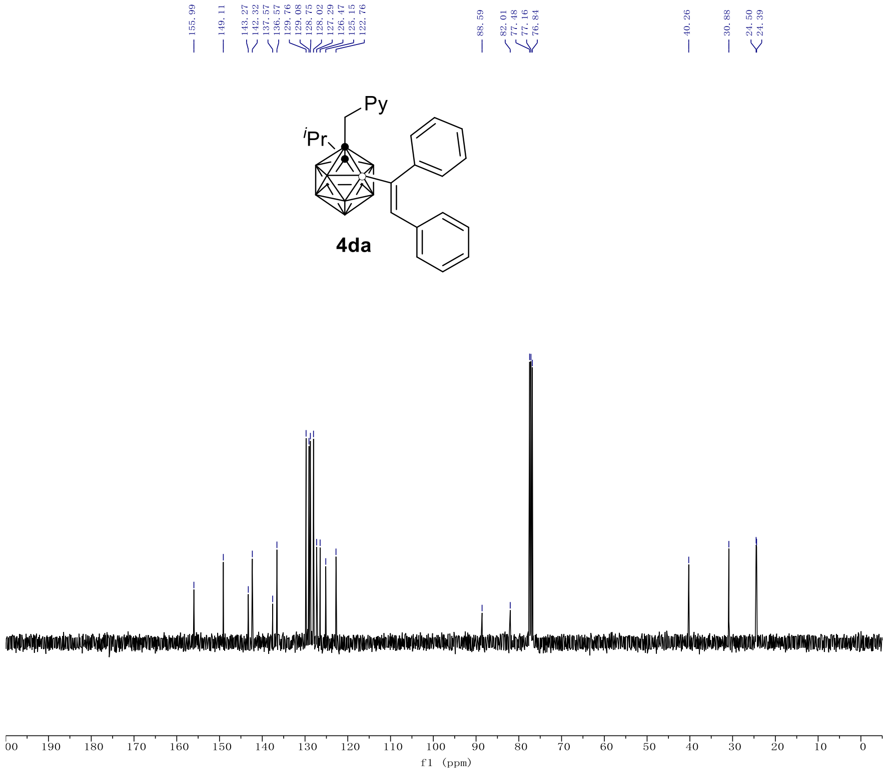

Parameter	Value
Data File Name	E:/ 积分核磁/ 1051/ 6-yss-1051-c/ 2/ fid
Title	6-yss-1051-c/ 2
Comment	
Origin	Bruker BioSpin GmbH
Owner	nmr
Site	
Spectrometer	spect
Author	
Solvent	CDCl3
Temperature	296.1
Pulse Sequence	zgpg30
Experiment	1D
Probe	Z116098_0640 (PA BBO 400S1 BBF-H-D-05 Z SP)
Number of Scans	18
Receiver Gain	196
Relaxation Delay	1.0000
Pulse Width	10.0000
Presaturation Frequency	
Acquisition Time	1.2999
Acquisition Date	2023-09-16T19:48:34
Modification Date	2023-09-16T19:48:34
Class	
Spectrometer Frequency	100.64
Spectral Width	25252.5
Lowest Frequency	-1549.4
Nucleus	13C
Acquired Size	32827
Spectral Size	131072

$^{11}B\{^{1}H\}$ NMR (128 MHz, $CDCl_3$) of **4da**

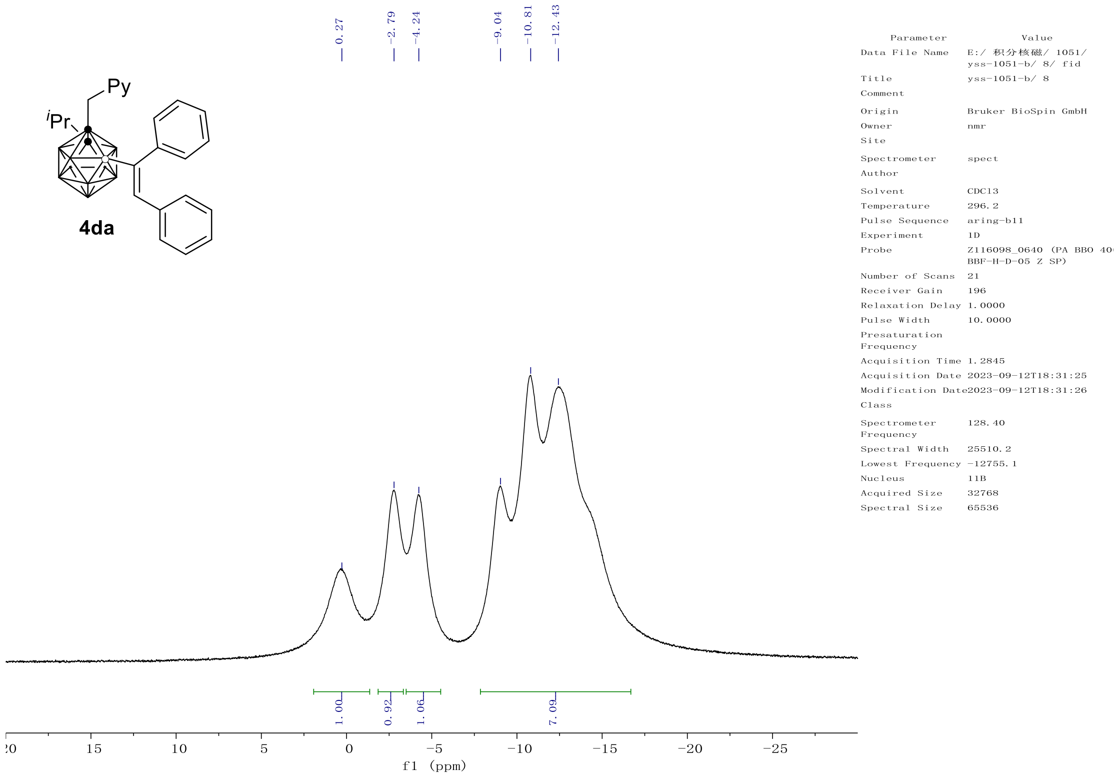

^{11}B NMR (128 MHz, $CDCl_3$) of **4da**

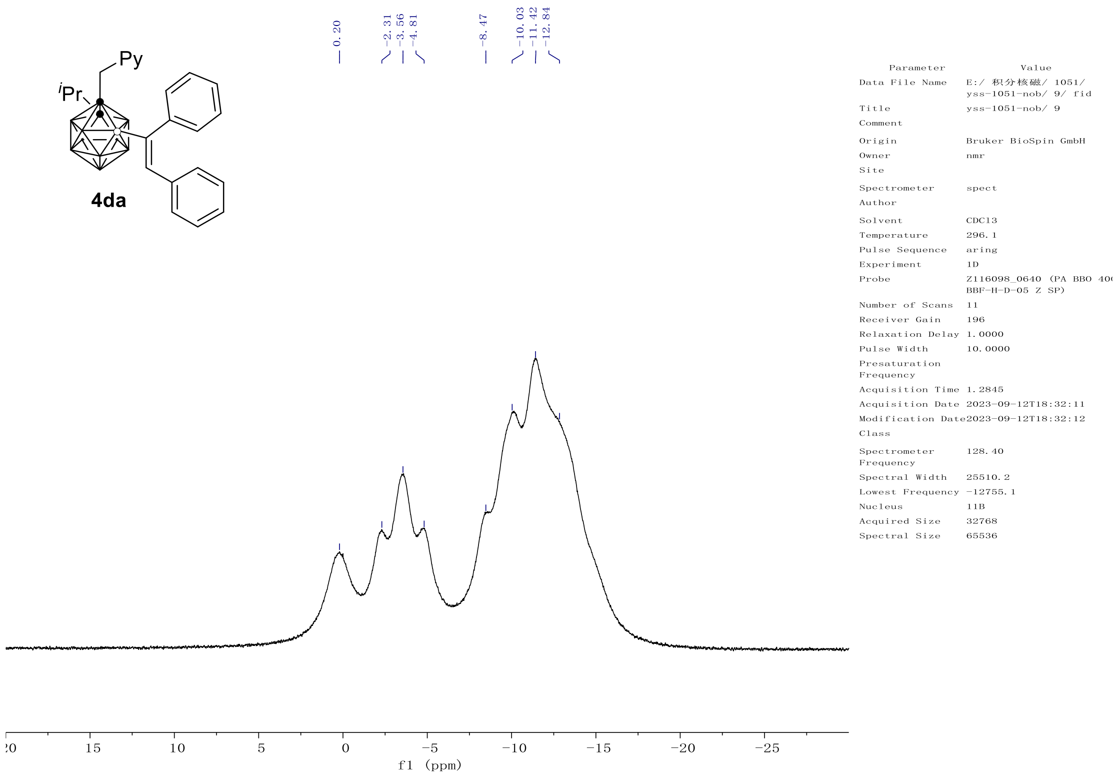

^{1}H NMR (400 MHz, $CDCl_3$) of **4ea**

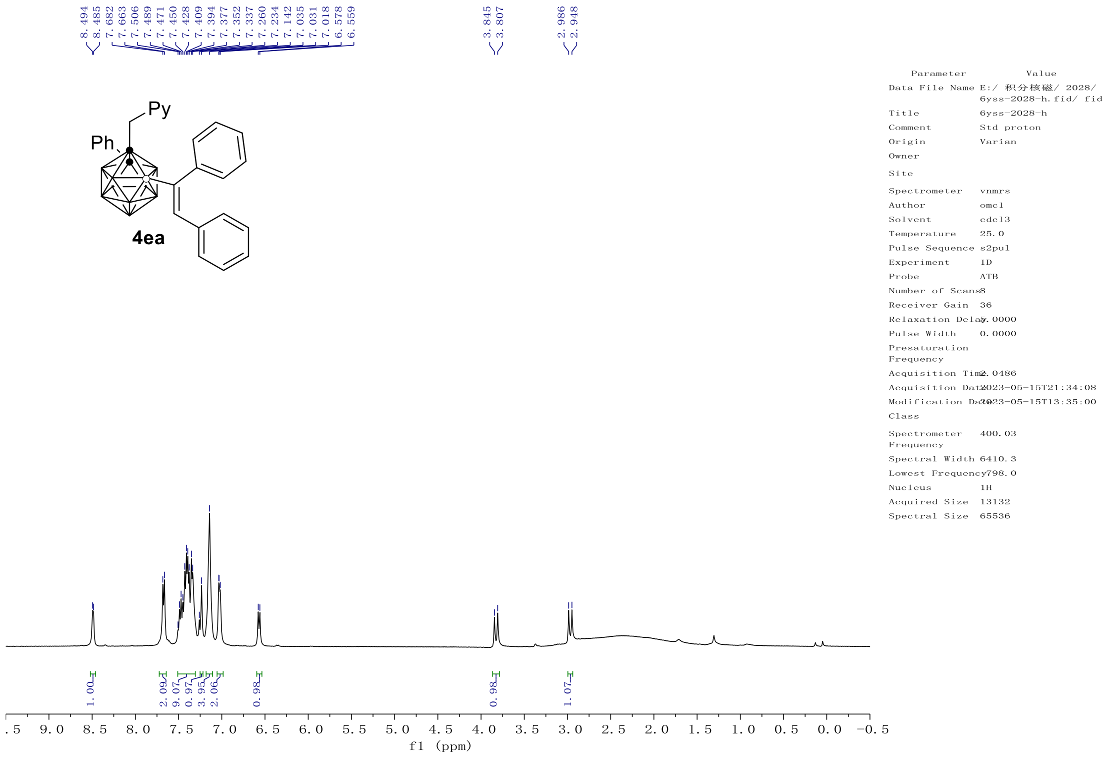

$^{13}C\{^{1}H\}$ NMR (101 MHz, $CDCl_3$) of **4ea**

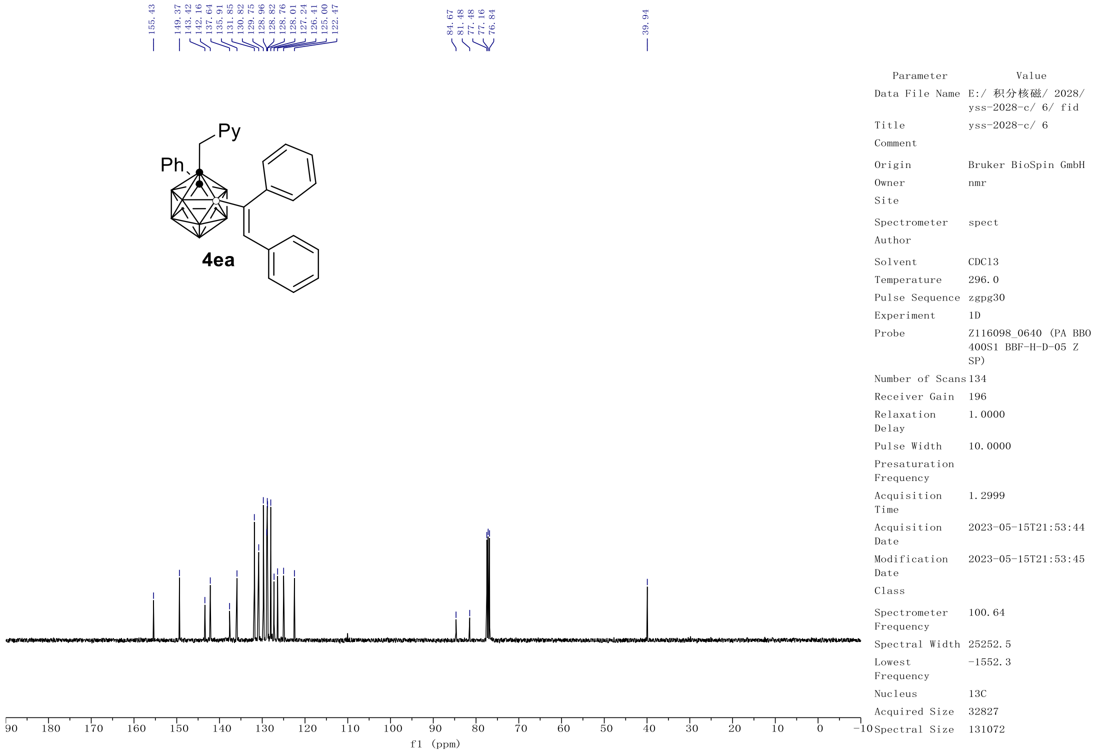

$^{11}B\{^{1}H\}$ NMR (128 MHz, $CDCl_3$) of **4ea**

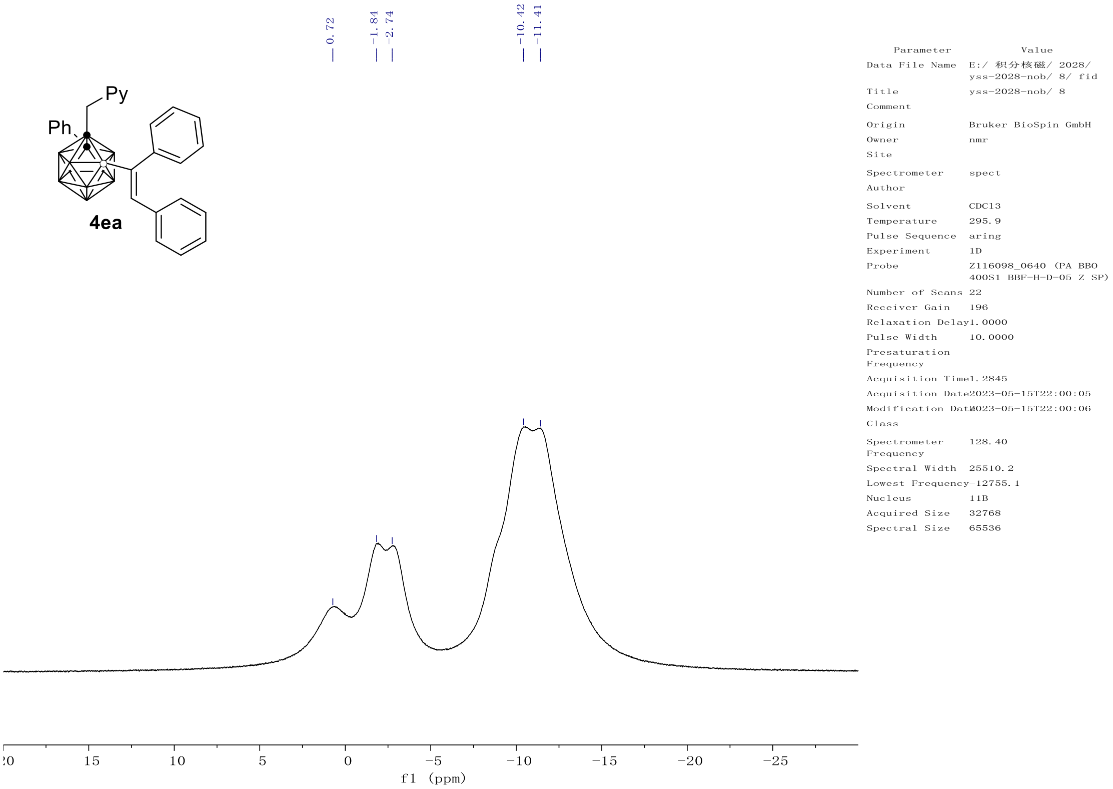

^{11}B NMR (128 MHz, $CDCl_3$) of **4ea**

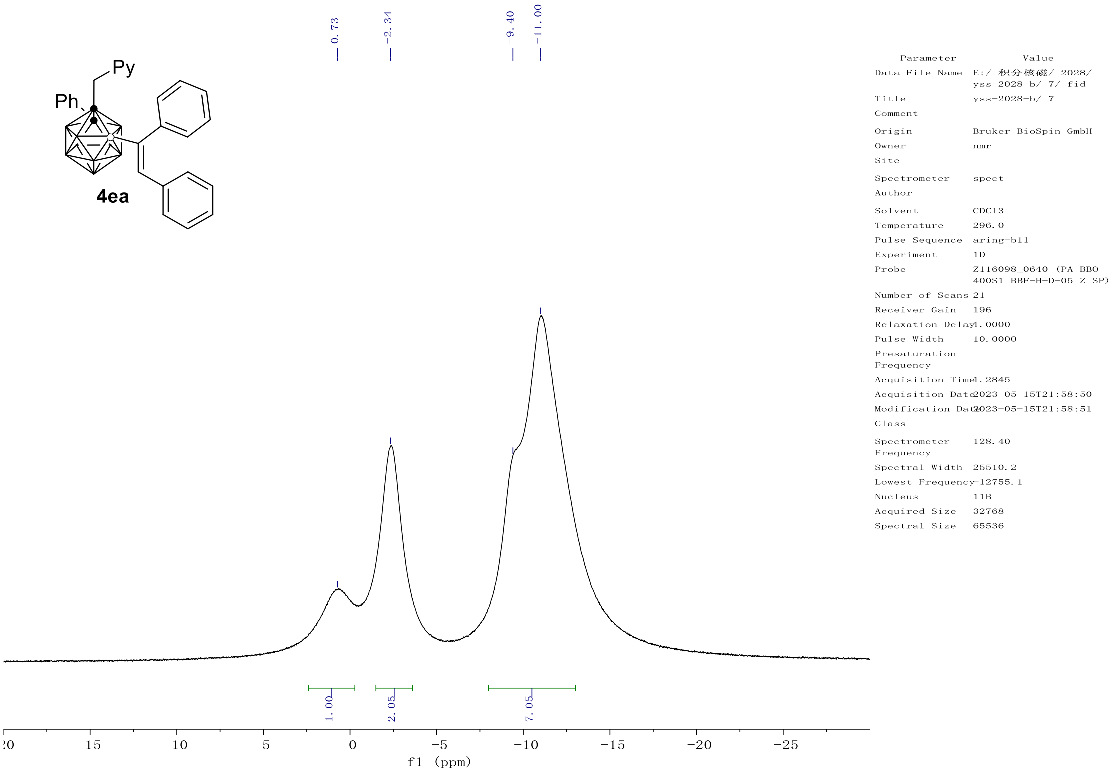

^{1}H NMR (400 MHz, $CDCl_3$) of **4fa**

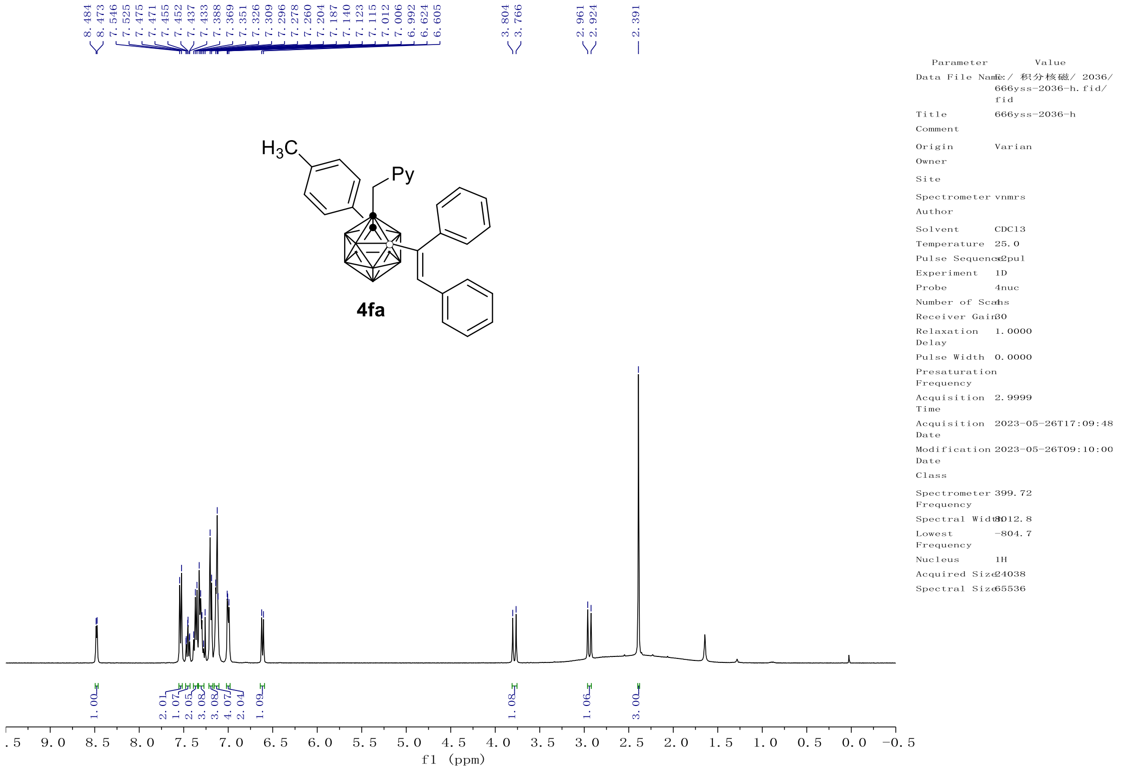

^{13}C{^{1}H} NMR (101 MHz, $CDCl_3$) of **4fa**

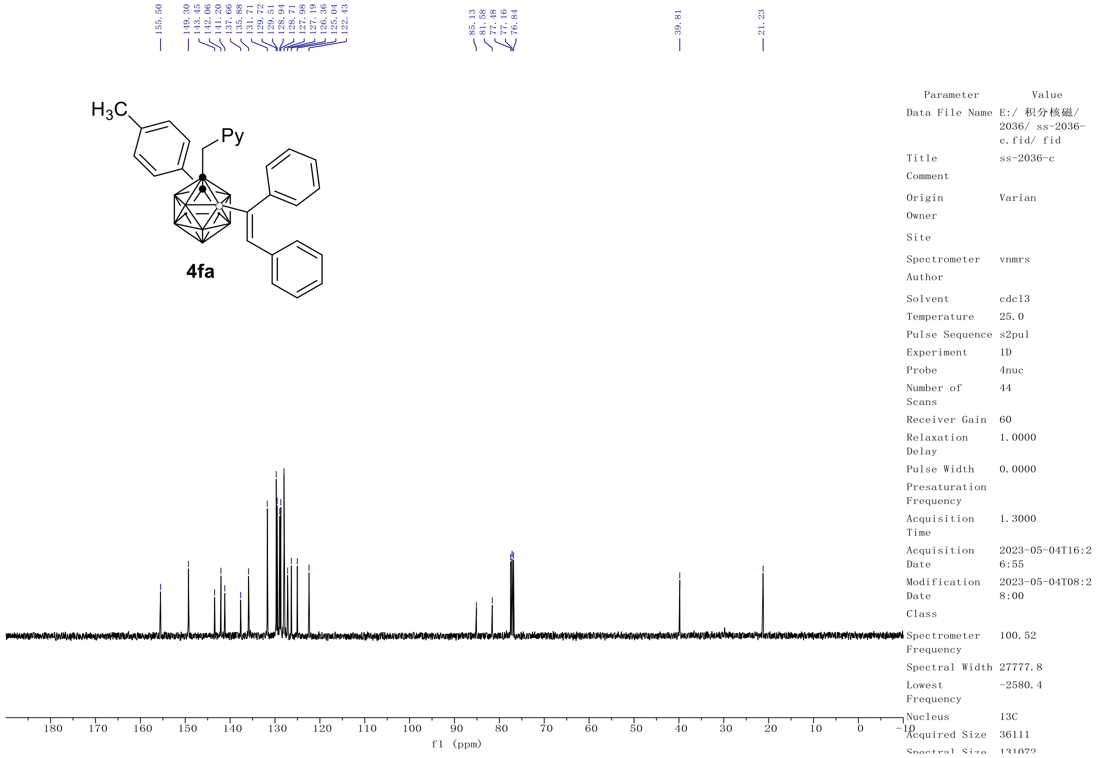

$^{11}B\{^{1}H\}$ NMR (128 MHz, $CDCl_3$) of **4fa**

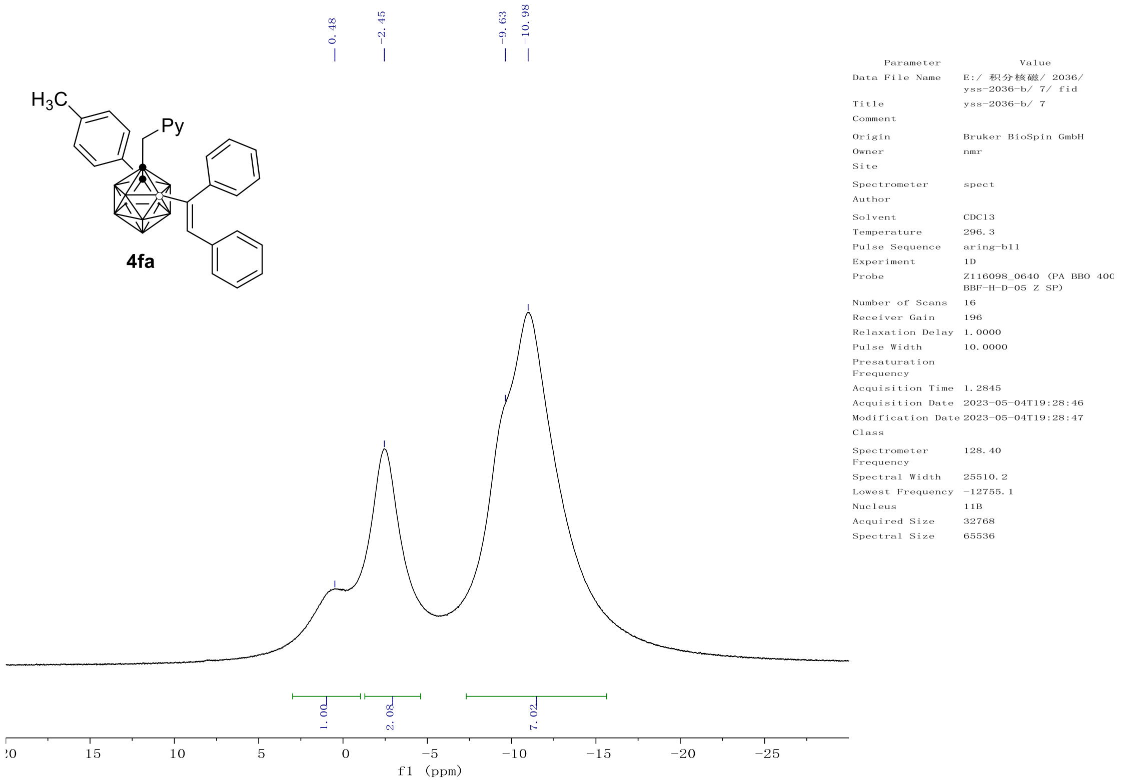

^{11}B NMR (128 MHz, $CDCl_3$) of **4fa**

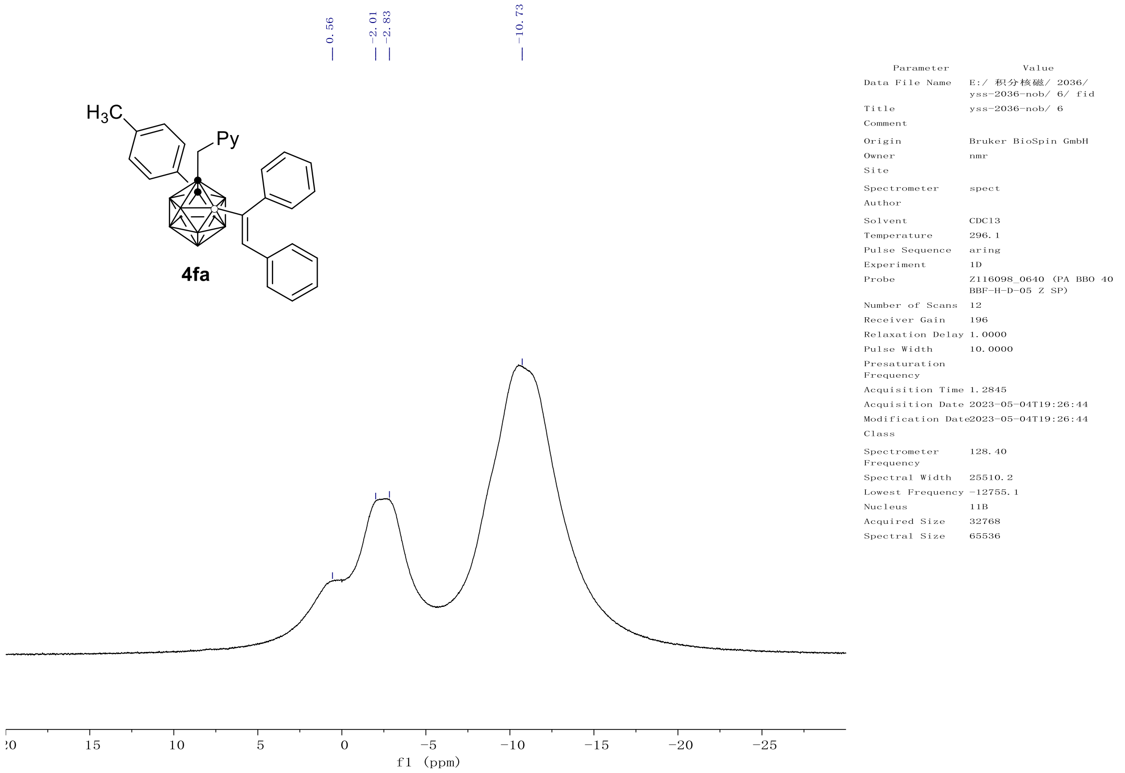

^{1}H NMR (400 MHz, $CDCl_3$) of **4ga**

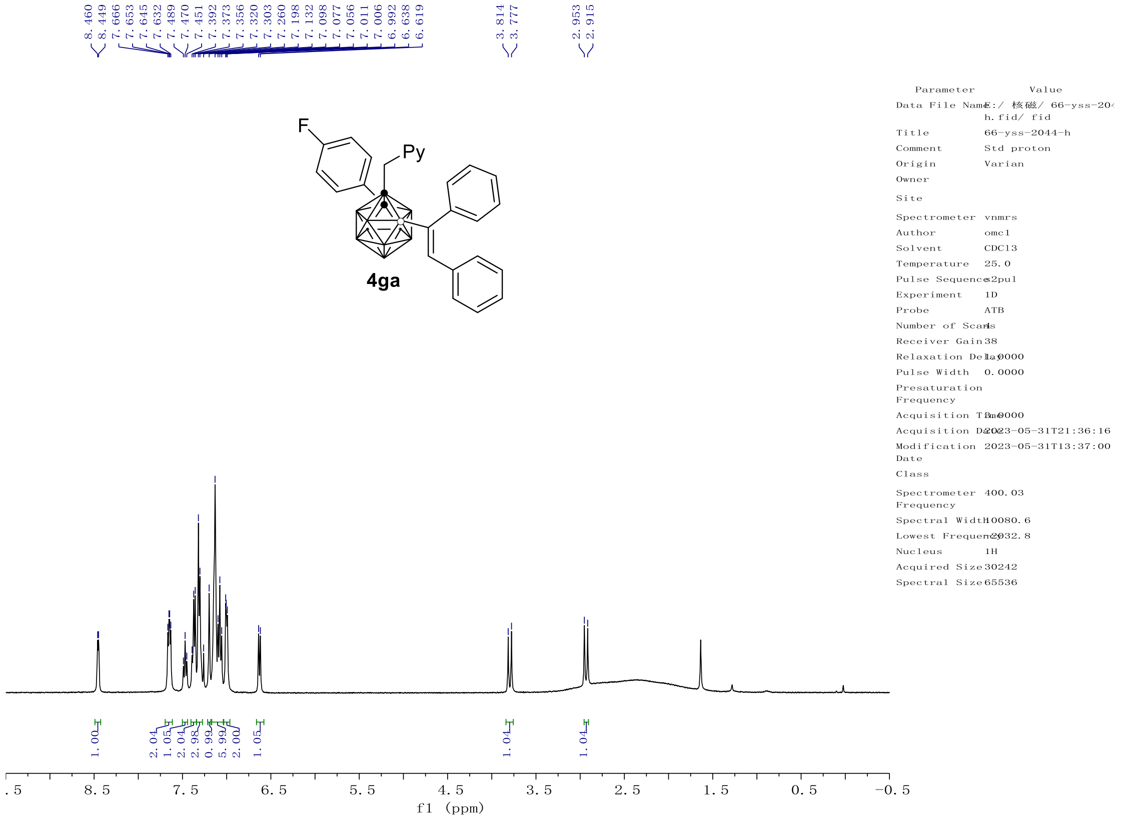

^{13}C{^{1}H} NMR (101 MHz, $CDCl_3$) of **4ga**

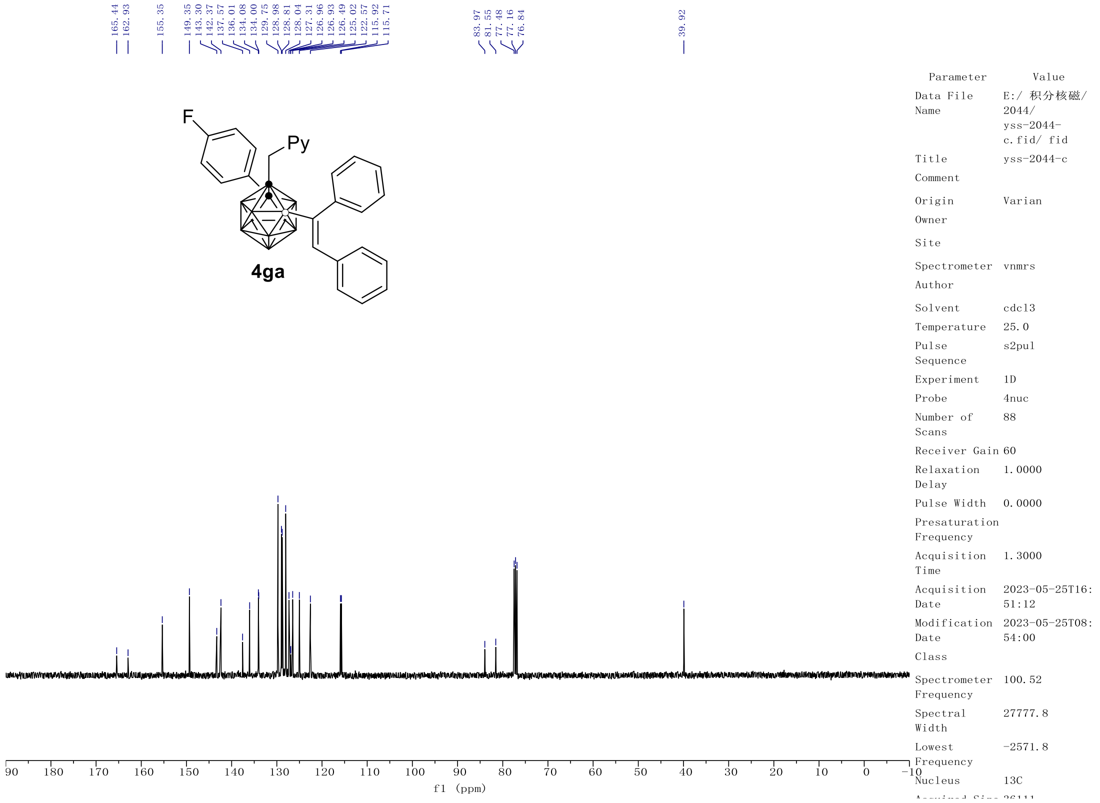

$^{11}B\{^1H\}$ NMR (128 MHz, $CDCl_3$) of **4ga**

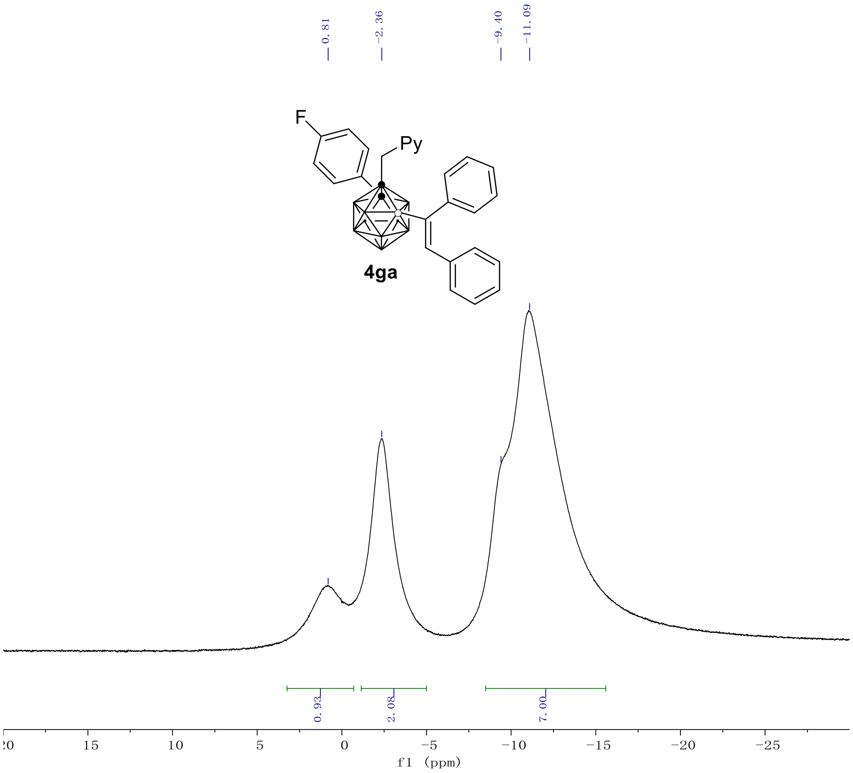

Parameter	Value
Data File Name	E:/ 积分核磁/ 2044/ yss-2044-b/ 17/ fid
Title	yss-2044-b/ 17
Comment	
Origin	Bruker BioSpin GmbH
Owner	nmr
Site	
Spectrometer	spect
Author	
Solvent	CDCl3
Temperature	296.1
Pulse Sequence	aring-b11
Experiment	1D
Probe	Z116098_0640 (PA BBO 400 BBF-H-D-05 Z SP)
Number of Scans	17
Receiver Gain	196
Relaxation Delay	1.0000
Pulse Width	10.0000
Presaturation Frequency	
Acquisition Time	1.2845
Acquisition Date	2023-05-25T16:44:52
Modification Date	2023-05-25T16:44:53
Class	
Spectrometer Frequency	128.40
Spectral Width	25510.2
Lowest Frequency	-12755.1
Nucleus	11B
Acquired Size	32768
Spectral Size	65536

^{11}B NMR (128 MHz, $CDCl_3$) of **4ga**

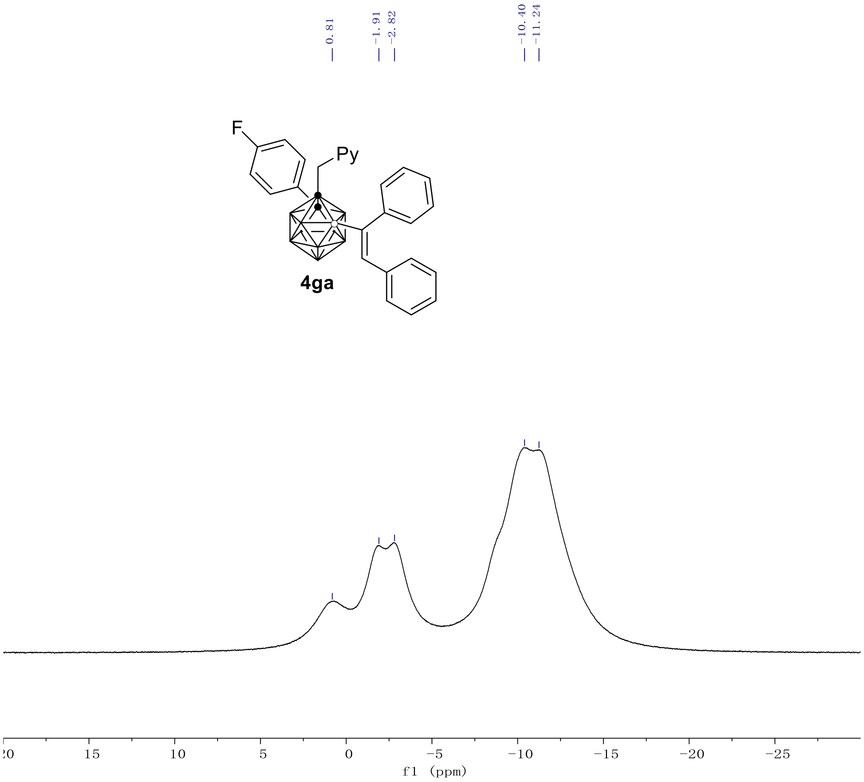

Parameter	Value
Data File Name	E:/ 积分核磁/ 2044/ yss-2044-nob/ 16/ fid
Title	yss-2044-nob/ 16
Comment	
Origin	Bruker BioSpin GmbH
Owner	nmr
Site	
Spectrometer	spect
Author	
Solvent	CDCl3
Temperature	296.0
Pulse Sequence	aring
Experiment	1D
Probe	Z116098_0640 (PA BBO 400S1 BBF-H-D-05 Z SP)
Number of Scans	20
Receiver Gain	196
Relaxation Delay	1.0000
Pulse Width	10.0000
Presaturation Frequency	
Acquisition Time	1.2845
Acquisition Date	2023-05-25T16:43:49
Modification Date	2023-05-25T16:43:50
Class	
Spectrometer Frequency	128.40
Spectral Width	25510.2
Lowest Frequency	-12755.1
Nucleus	11B
Acquired Size	32768
Spectral Size	65536

^{19}F NMR (376 MHz, $CDCl_3$) of **4ga**

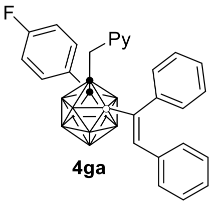

4ga

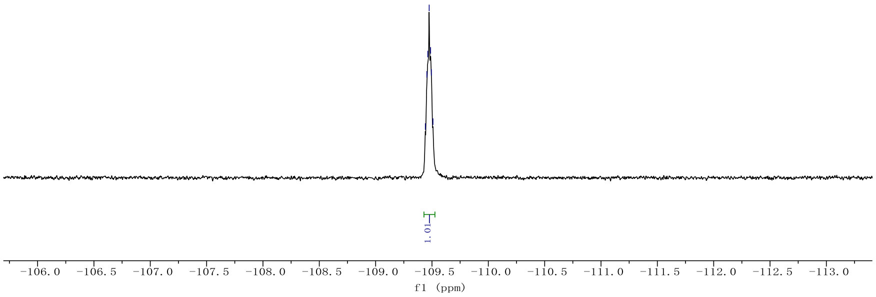

Parameter	Value
Data File Name	E:/ 积分核磁/ 2044/ yss-2044-f.fid/ fid
Title	yss-2044-f
Comment	Std Fluorine
Origin	Varian
Owner	
Site	
Spectrometer	vnmrs
Author	
Solvent	CDCl3
Temperature	25.0
Pulse Sequence	s2pul
Experiment	1D
Probe	ATB
Number of Scans	8
Receiver Gain	60
Relaxation Delay	1.0000
Pulse Width	0.0000
Presaturation Frequency	
Acquisition Time	1.0000
Acquisition Date	2023-05-31T21:37:17
Modification Date	2023-05-31T13:37:00
Class	
Spectrometer Frequency	376.37
Spectral Width	156250.0
Lowest Frequency	-115765.3
Nucleus	19F
Acquired Size	156250
Spectral Size	524288

^{1}H NMR (400 MHz, $CDCl_3$) of **4ha**

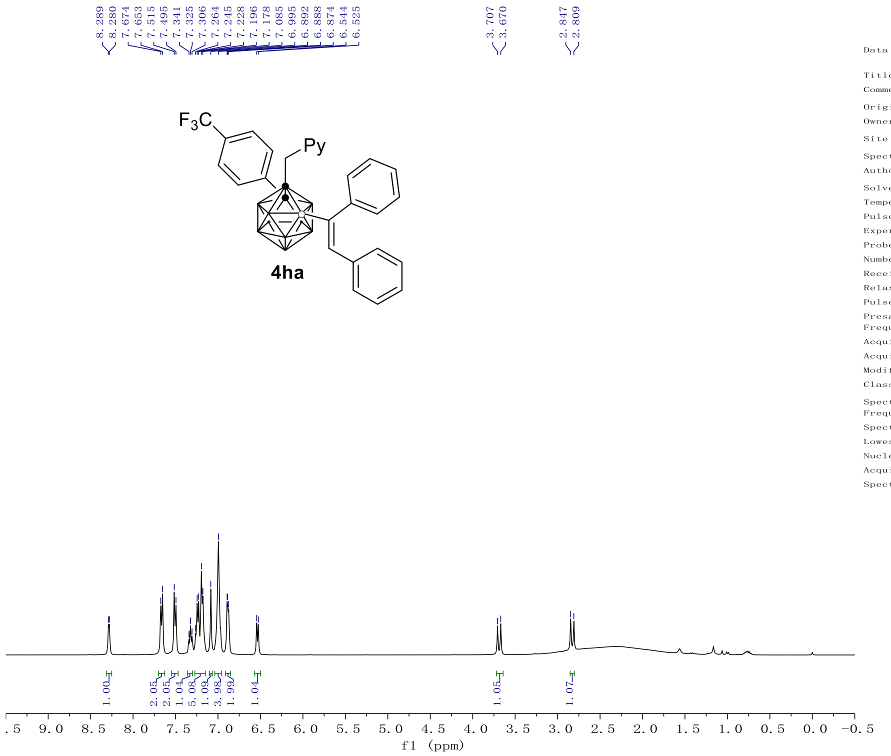

Parameter	Value
Data File Name	E:/ 积分核磁/ 2059/ yss-2059-h.fid/ fid
Title	yss-2059-h
Comment	
Origin	Varian
Owner	
Site	
Spectrometer	vnmrs
Author	
Solvent	CDCl3
Temperature	25.0
Pulse Sequence	s2pul
Experiment	1D
Probe	4nuc
Number of Scans	12
Receiver Gain	24
Relaxation Delay	1.0000
Pulse Width	0.0000
Presaturation Frequency	
Acquisition Time	3.0000
Acquisition Date	2023-07-07T18:52:01
Modification Date	2023-07-07T10:53:00
Class	
Spectrometer Frequency	399.72
Spectral Width	8389.3
Lowest Frequency	-1261.9
Nucleus	1H
Acquired Size	25168
Spectral Size	65536

^{13}C{^{1}H} NMR (101 MHz, $CDCl_3$) of **4ha**

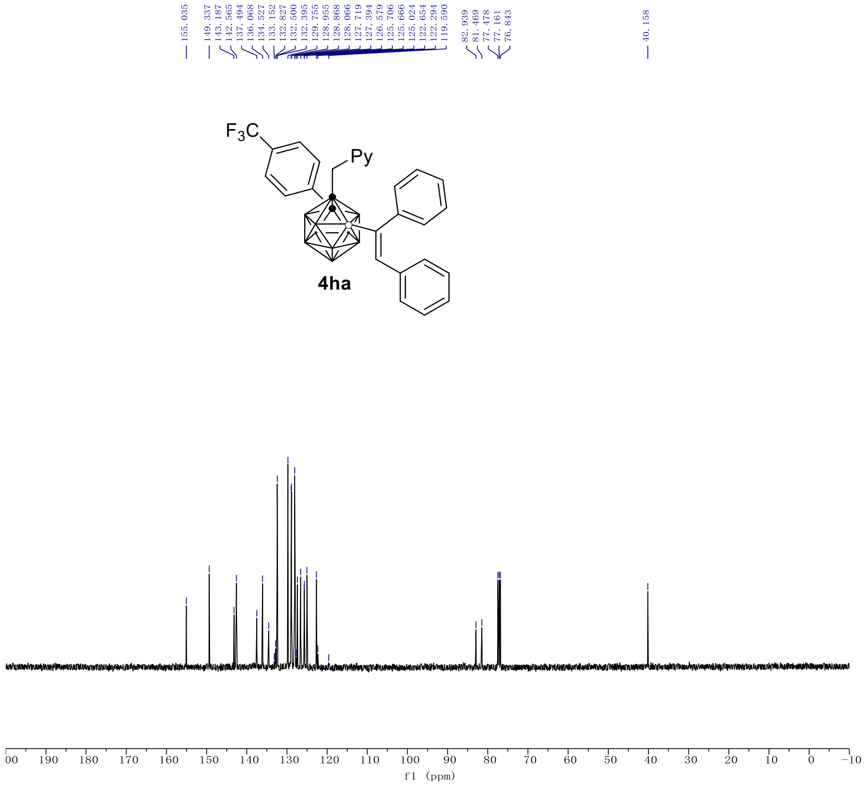

Parameter	Value
Data File Name	E:/ 积分核磁/ 2059/ yss-2059-c.fid/ fid
Title	yss-2059-c
Comment	
Origin	Varian
Owner	
Site	
Spectrometer	vnmrs
Author	
Solvent	cdcl3
Temperature	25.0
Pulse Sequence	s2pul
Experiment	1D
Probe	4nuc
Number of Scans	72
Receiver Gain	60
Relaxation Delay	1.0000
Pulse Width	0.0000
Presaturation Frequency	
Acquisition Time	1.3000
Acquisition Date	2023-07-07T18:53:48
Modification Date	2023-07-07T10:56:00
Class	
Spectrometer Frequency	100.52
Spectral Width	27777.8
Lowest Frequency	-2574.6
Nucleus	13C
Acquired Size	36111
[illegible]	[illegible]

$^{11}B\{^{1}H\}$ NMR (128 MHz, $CDCl_3$) of **4ha**

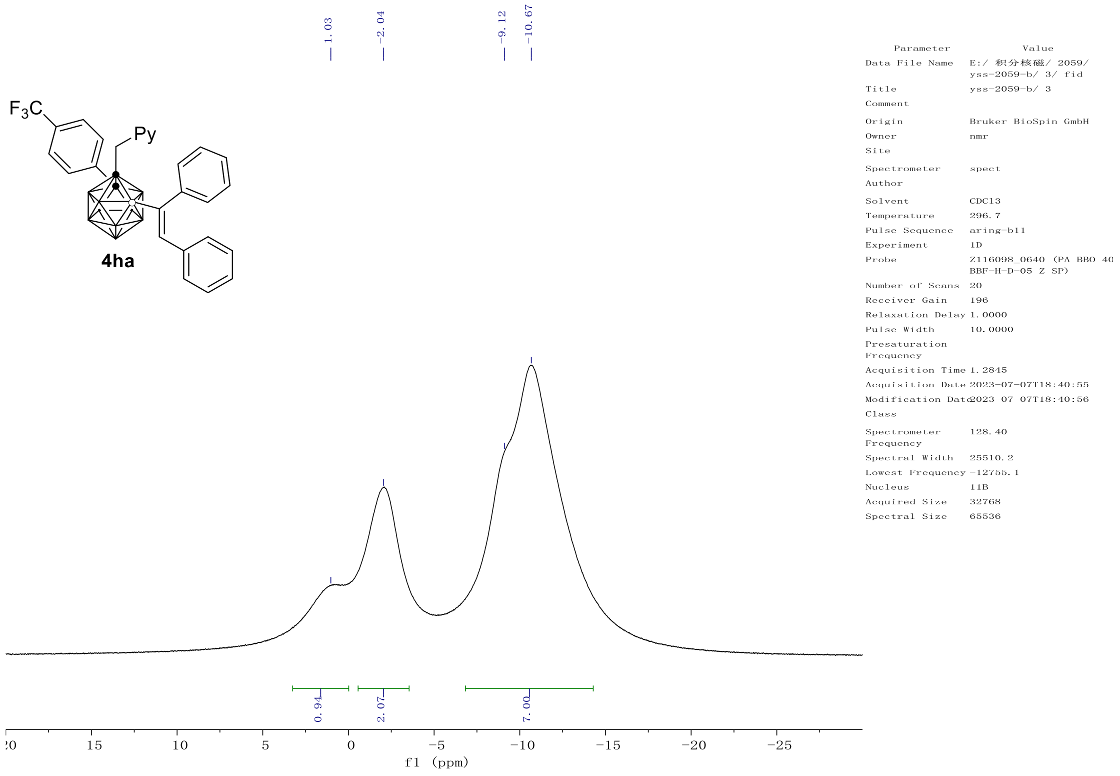

^{11}B NMR (128 MHz, $CDCl_3$) of **4ha**

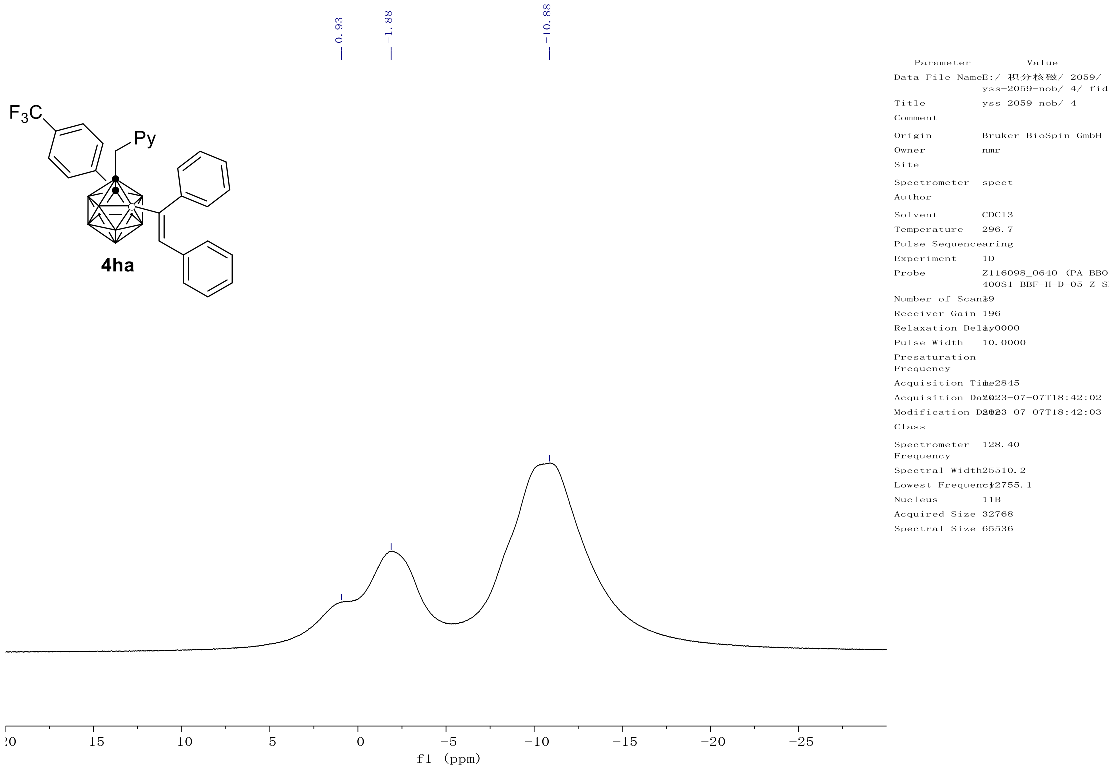

^{19}F NMR (376 MHz, $CDCl_3$) of **4ha**

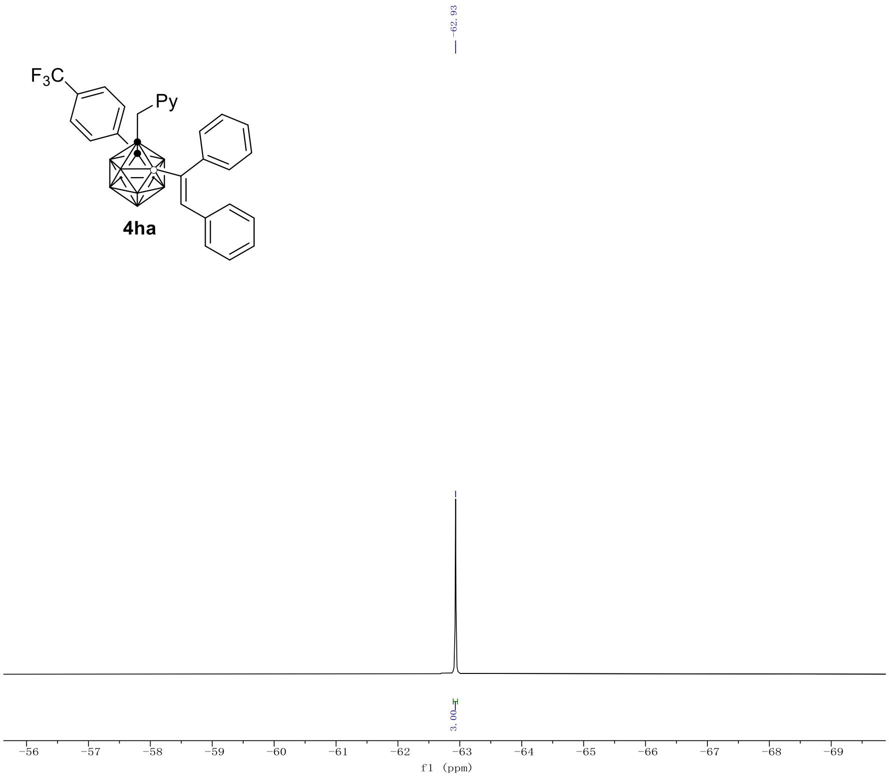

Parameter	Value
Data File Name	E:/ 积分核磁/ 2059/ yss-2059-f.fid/ fid
Title	yss-2059-f
Comment	
Origin	Varian
Owner	
Site	
Spectrometer	vnmrs
Author	
Solvent	CDCl3
Temperature	25.0
Pulse Sequence	s2pul
Experiment	1D
Probe	4nuc
Number of Scans	28
Receiver Gain	48
Relaxation Delay	1.0000
Pulse Width	0.0000
Presaturation Frequency	
Acquisition Time	1.0000
Acquisition Date	2023-07-07T18:56:57
Modification Date	2023-07-07T10:58:00
Class	
Spectrometer Frequency	376.07
Spectral Width	147058.8
Lowest Frequency	-111478.7
Nucleus	19F
Acquired Size	147059
Spectral Size	524288

^{1}H NMR (400 MHz, $CDCl_3$) of **4aa**

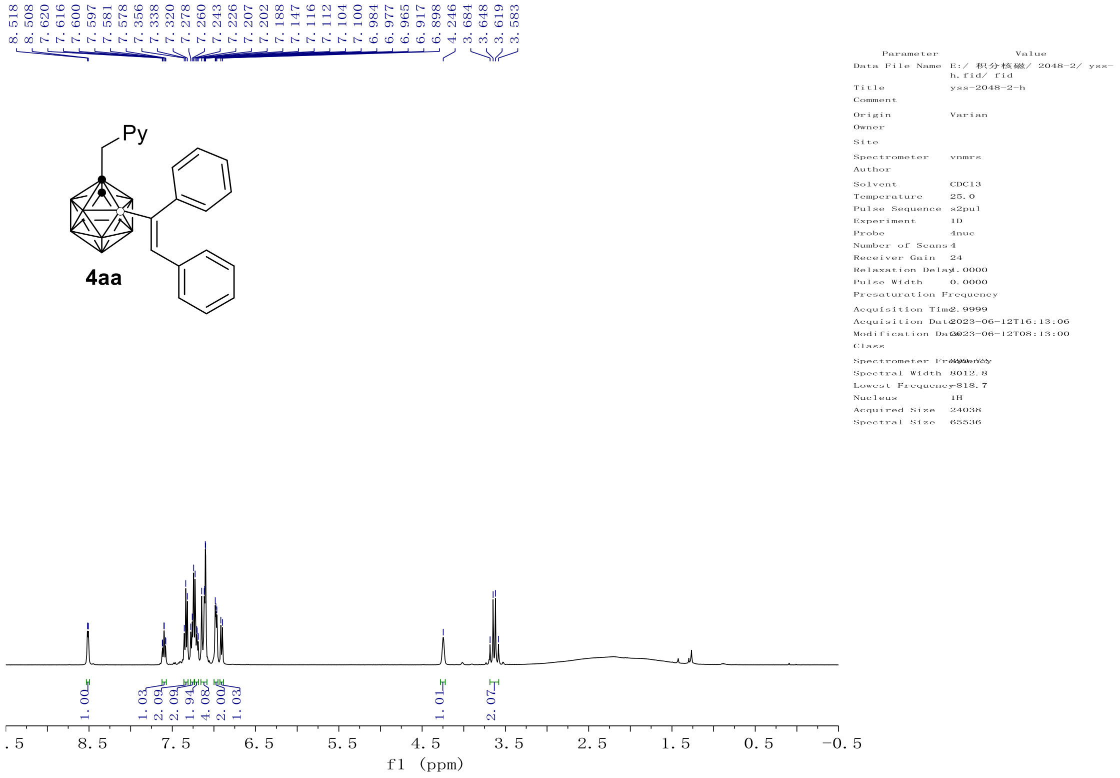

^{13}C{^{1}H} NMR (101 MHz, $CDCl_3$) of **4aa**

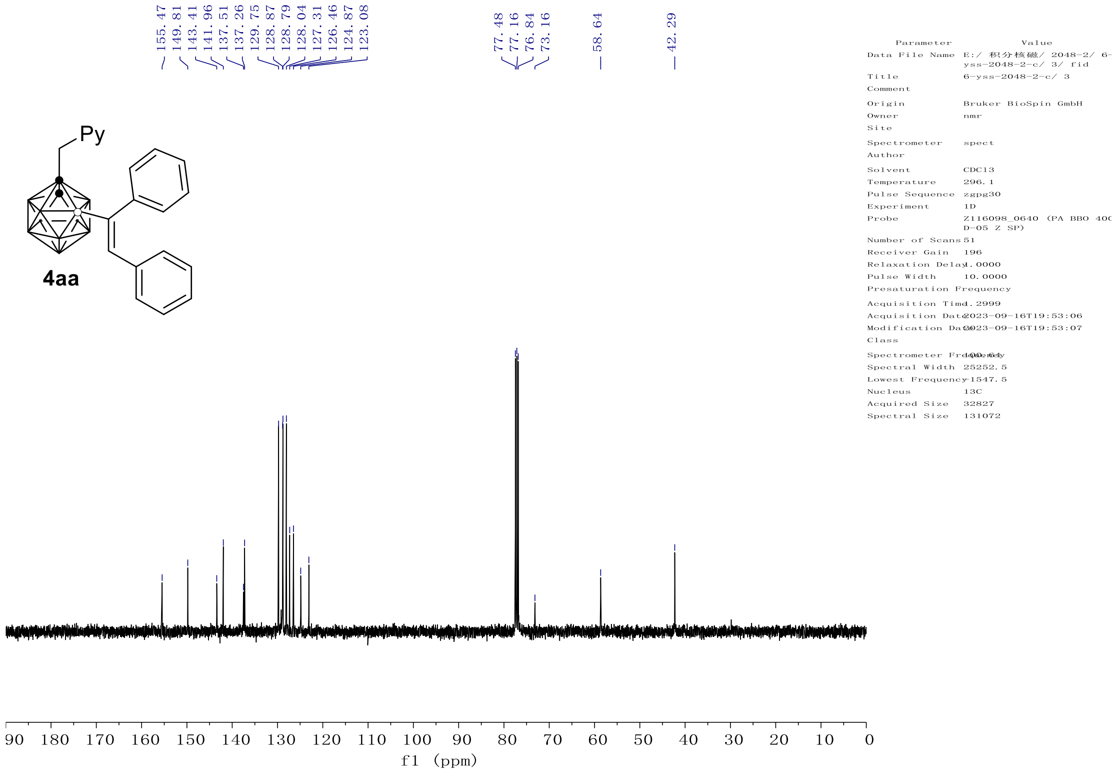

$^{11}B\{^{1}H\}$ NMR (128 MHz, $CDCl_3$) of **4aa**

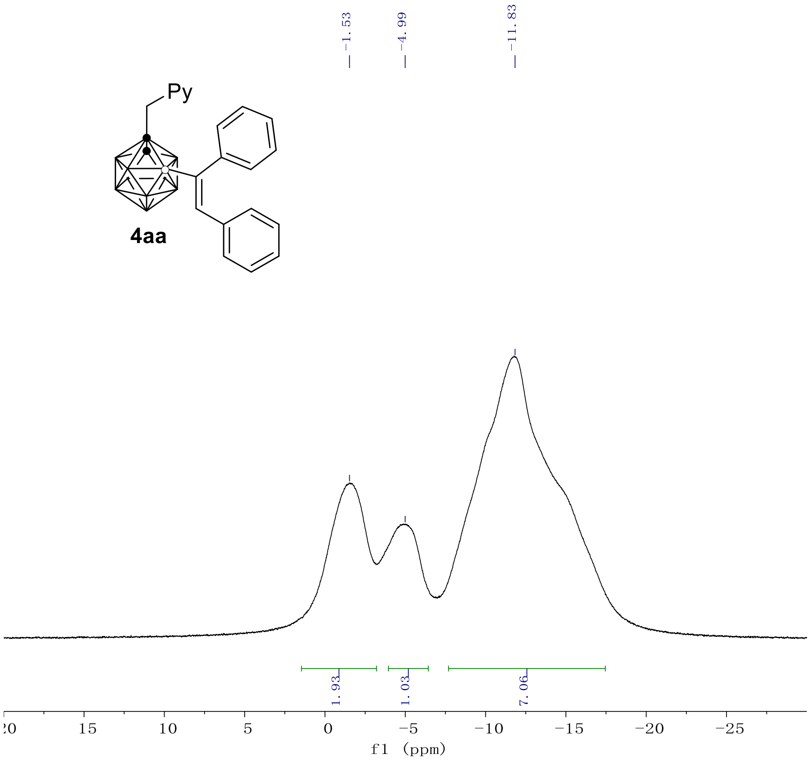

Parameter	Value
Data File Name	E:/ 积分核磁/ 2048-2/ yss-2048-fid
Title	yss-2048-2-b-2/ 14
Comment	
Origin	Bruker BioSpin GmbH
Owner	nmr
Site	
Spectrometer	spect
Author	
Solvent	CDCl3
Temperature	296.6
Pulse Sequence	aring-b11
Experiment	1D
Probe	Z116098_0640 (PA BBO 400S1 BBF-
Number of Scans	13
Receiver Gain	196
Relaxation Delay	1.0000
Pulse Width	10.0000
Presaturation Frequency	
Acquisition Time	1.2845
Acquisition Date	2023-06-12T16:10:35
Modification Date	2023-06-12T16:10:36
Class	
Spectrometer Frequency	128.40
Spectral Width	25510.2
Lowest Frequency	-12755.1
Nucleus	11B
Acquired Size	32768
Spectral Size	65536

^{11}B NMR (128 MHz, $CDCl_3$) of **4aa**

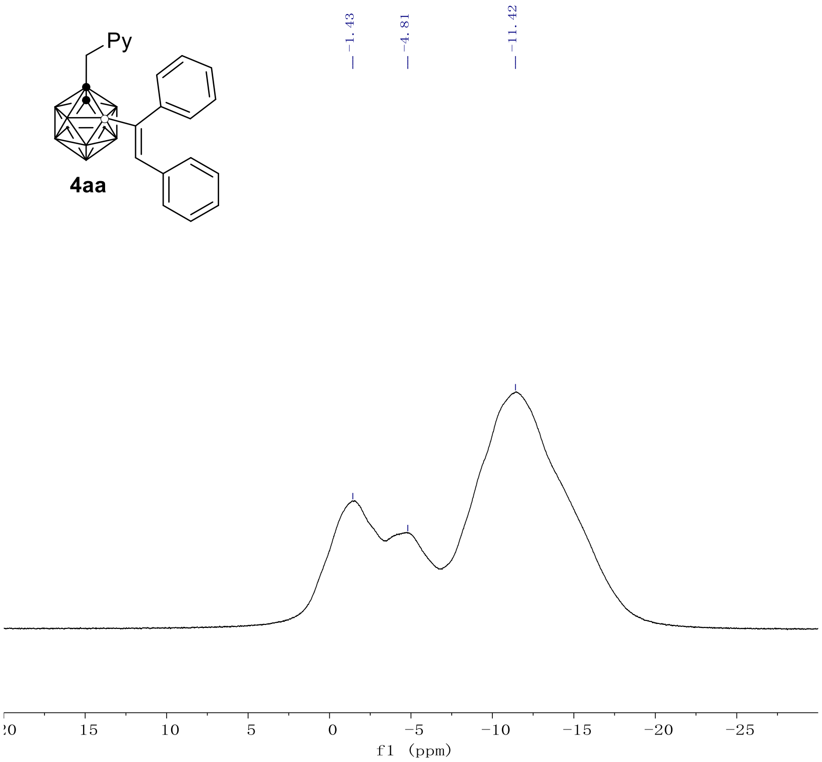

Parameter	Value
Data File Name	E:/ 积分核磁/ 2048-2/ yss-2048-fid
Title	yss-2048-2-nob/ 12
Comment	
Origin	Bruker BioSpin GmbH
Owner	nmr
Site	
Spectrometer	spect
Author	
Solvent	CDCl3
Temperature	296.5
Pulse Sequence	aring
Experiment	1D
Probe	Z116098_0640 (PA BBO 400S1 BBF-
Number of Scans	20
Receiver Gain	196
Relaxation Delay	1.0000
Pulse Width	10.0000
Presaturation Frequency	
Acquisition Time	1.2845
Acquisition Date	2023-06-12T16:07:29
Modification Date	2023-06-12T16:07:30
Class	
Spectrometer Frequency	128.40
Spectral Width	25510.2
Lowest Frequency	-12755.1
Nucleus	11B
Acquired Size	32768
Spectral Size	65536

^{1}H NMR (400 MHz, $CDCl_3$) of **3ba**

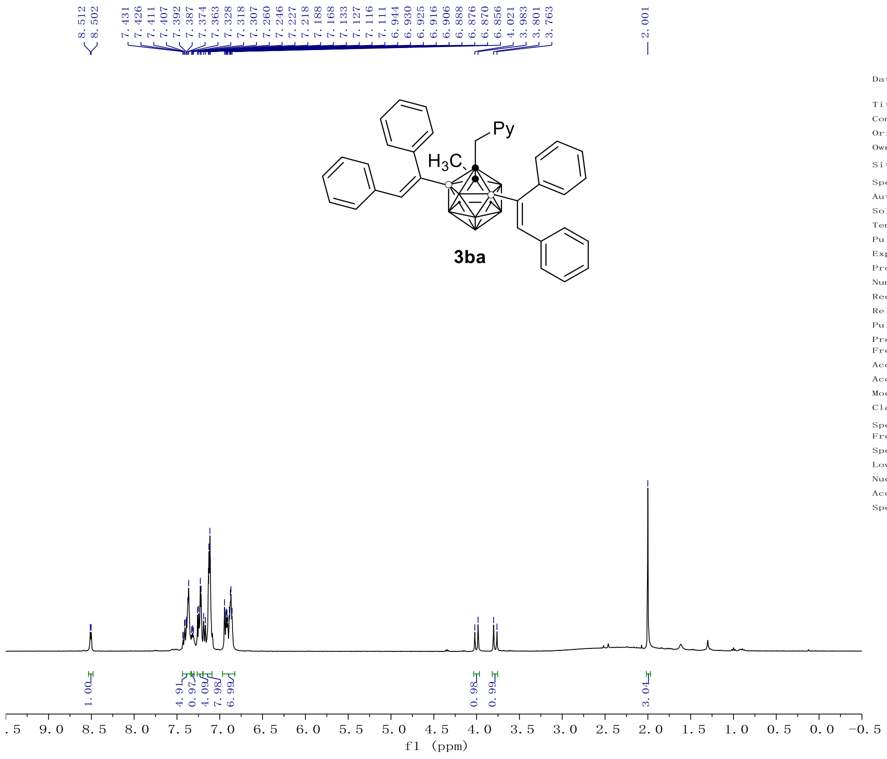

Parameter	Value
Data File Name	E:/ 积分核磁/ 2055/ yss-2055-h.fid/ fid
Title	yss-2055-h
Comment	Std proton
Origin	Varian
Owner	
Site	
Spectrometer	vnmrs
Author	omc1
Solvent	CDC13
Temperature	25.0
Pulse Sequence	s2pul
Experiment	1D
Probe	ATB
Number of Scans	4
Receiver Gain	28
Relaxation Delay	1.0000
Pulse Width	0.0000
Presaturation Frequency	
Acquisition Time	3.0000
Acquisition Date	2023-06-28T21:36:22
Modification Date	2023-06-28T13:37:00
Class	
Spectrometer Frequency	400.03
Spectral Width	10080.6
Lowest Frequency	-2032.7
Nucleus	1H
Acquired Size	30242
Spectral Size	65536

^{13}C{^{1}H} NMR (101 MHz, $CDCl_3$) of **3ba**

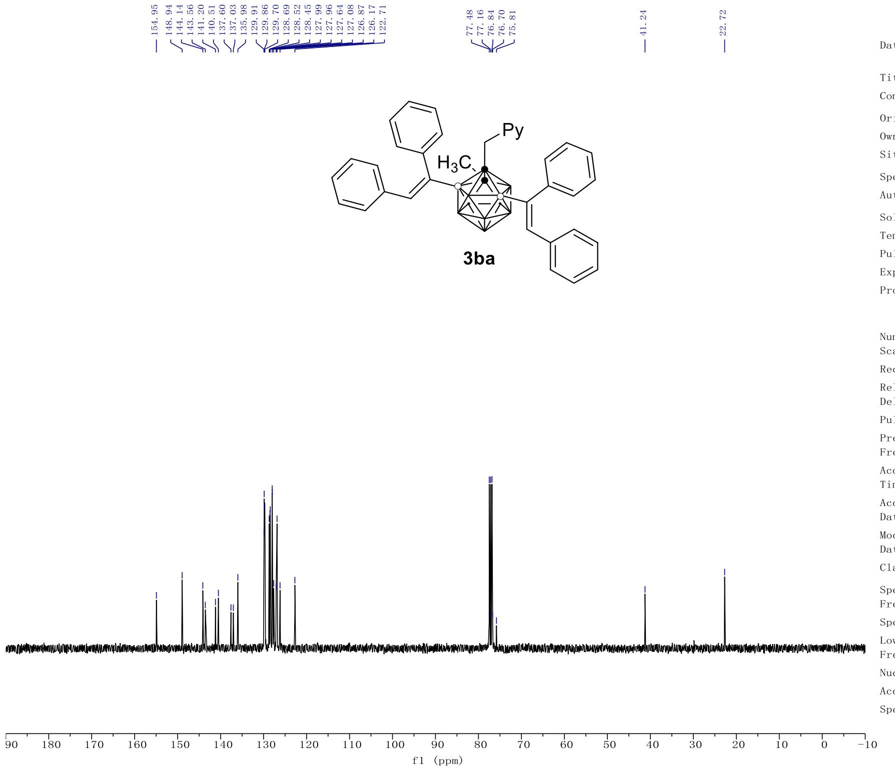

Parameter	Value
Data File Name	E:/ 积分核磁/ 2055/ yss-2055-c/ 5/ fid
Title	yss-2055-c/ 5
Comment	
Origin	Bruker BioSpin GmbH
Owner	nmr
Site	
Spectrometer	spect
Author	
Solvent	CDCl3
Temperature	296.5
Pulse Sequence	zgpg30
Experiment	1D
Probe	Z116098_0640 (PA BBO 400S1 BBF-H-D-05 Z SP)
Number of Scans	49
Receiver Gain	196
Relaxation Delay	1.0000
Pulse Width	10.0000
Presaturation Frequency	
Acquisition Time	1.2999
Acquisition Date	2023-06-28T22:04:32
Modification Date	2023-06-28T22:04:33
Class	
Spectrometer Frequency	100.64
Spectral Width	25252.5
Lowest Frequency	-1550.5
Nucleus	13C
Acquired Size	32827
Spectral Size	131072

$^{11}B\{^{1}H\}$ NMR (128 MHz, $CDCl_3$) of **3ba**

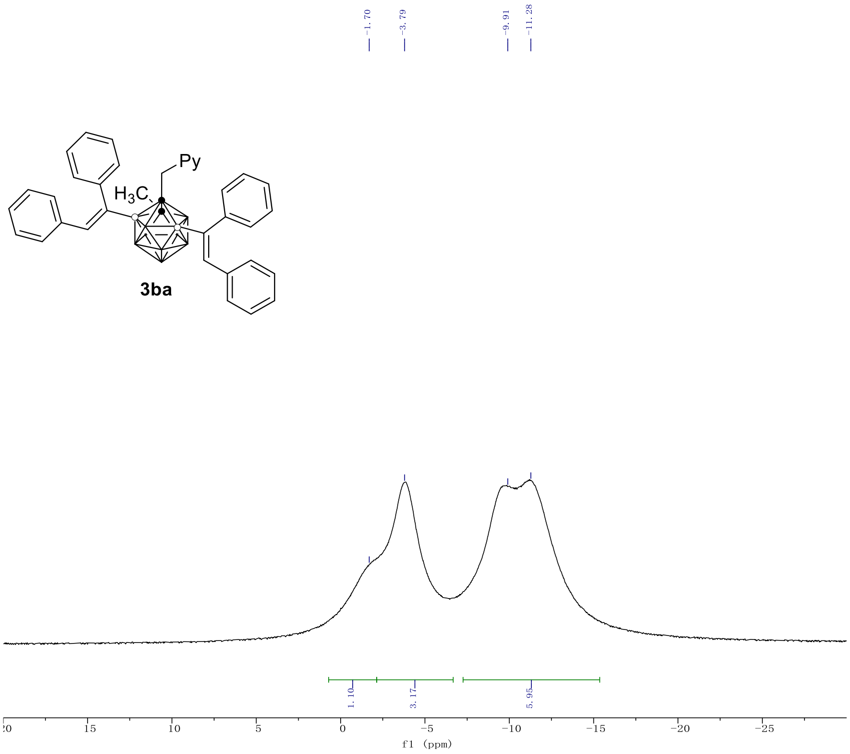

Parameter	Value
Data File Name	E:/ 积分核磁/ 2055/ yss-2055-b/ 3/ fid
Title	yss-2055-b/ 3
Comment	
Origin	Bruker BioSpin GmbH
Owner	nmr
Site	
Spectrometer	spect
Author	
Solvent	CDCl3
Temperature	296.6
Pulse Sequence	aring-b11
Experiment	1D
Probe	Z116098_0640 (PA BBO 400S1 BBF-H-D-05 Z SP)
Number of Scans	26
Receiver Gain	196
Relaxation Delay	1.0000
Pulse Width	10.0000
Presaturation Frequency	
Acquisition Time	1.2845
Acquisition Date	2023-06-28T21:58:56
Modification Date	2023-06-28T21:58:56
Class	
Spectrometer Frequency	128.40
Spectral Width	25510.2
Lowest Frequency	-12755.1
Nucleus	11B
Acquired Size	32768
Spectral Size	65536

^{11}B NMR (128 MHz, $CDCl_3$) of **3ba**

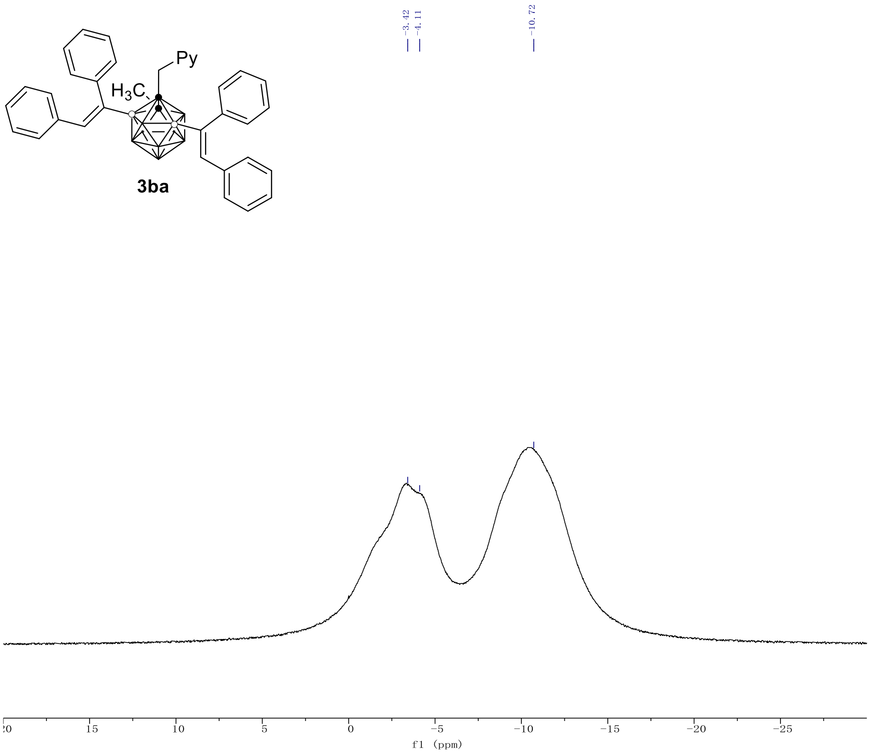

Parameter	Value
Data File Name	E:/ 积分核磁/ 2055/ yss-2055-nob/ 4/ fid
Title	yss-2055-nob/ 4
Comment	
Origin	Bruker BioSpin GmbH
Owner	nmr
Site	
Spectrometer	spect
Author	
Solvent	CDCl3
Temperature	296.6
Pulse Sequence	aring
Experiment	1D
Probe	Z116098_0640 (PA BBO 400S1 BBF-H-D-05 Z SP)
Number of Scans	27
Receiver Gain	196
Relaxation Delay	1.0000
Pulse Width	10.0000
Presaturation Frequency	
Acquisition Time	1.2845
Acquisition Date	2023-06-28T22:00:19
Modification Date	2023-06-28T22:00:20
Class	
Spectrometer Frequency	128.40
Spectral Width	25510.2
Lowest Frequency	-12755.1
Nucleus	11B
Acquired Size	32768
Spectral Size	65536

^{1}H NMR (400 MHz, $CDCl_3$) of **5a**

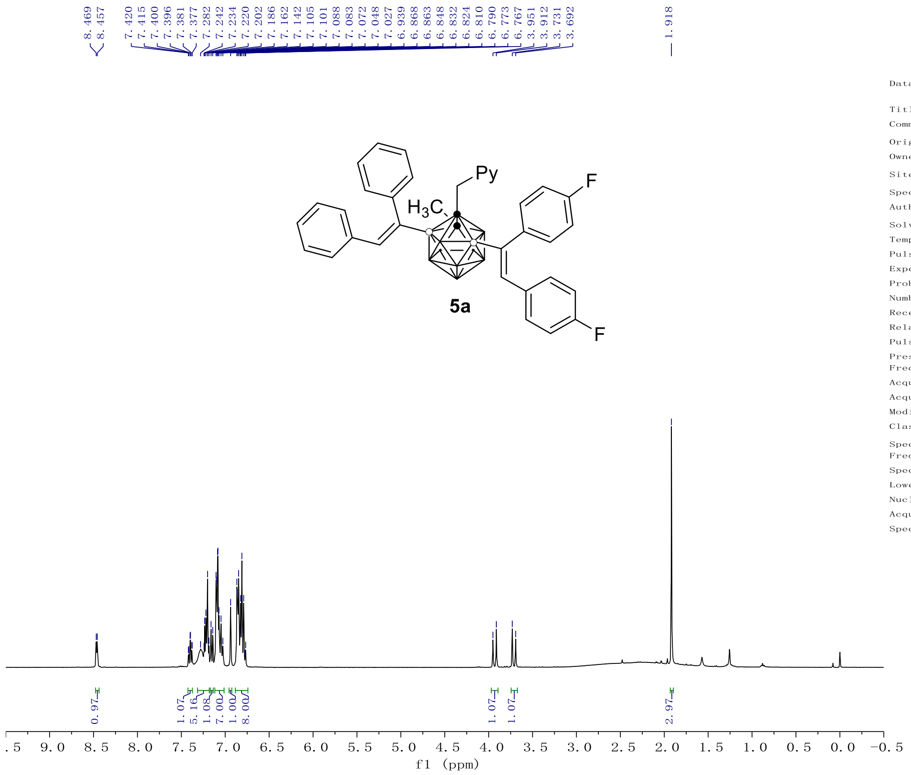

Parameter	Value
Data File Name	E:/ 积分核磁/ 3004/ 6 yss-3004-h.fid/ fid
Title	6-yss-3004-h
Comment	
Origin	Varian
Owner	
Site	
Spectrometer	vnmrs
Author	
Solvent	CDCl3
Temperature	25.0
Pulse Sequence	s2pul
Experiment	1D
Probe	4nuc
Number of Scans	4
Receiver Gain	30
Relaxation Delay	1.0000
Pulse Width	0.0000
Presaturation Frequency	
Acquisition Time	3.0000
Acquisition Date	2023-09-21T18:29:52
Modification Date	2023-09-21T10:30:00
Class	
Spectrometer Frequency	399.72
Spectral Width	8389.3
Lowest Frequency	-1202.7
Nucleus	1H
Acquired Size	25168
Spectral Size	65536

$^{13}C\{^{1}H\}$ NMR (101 MHz, $CDCl_3$) of **5a**

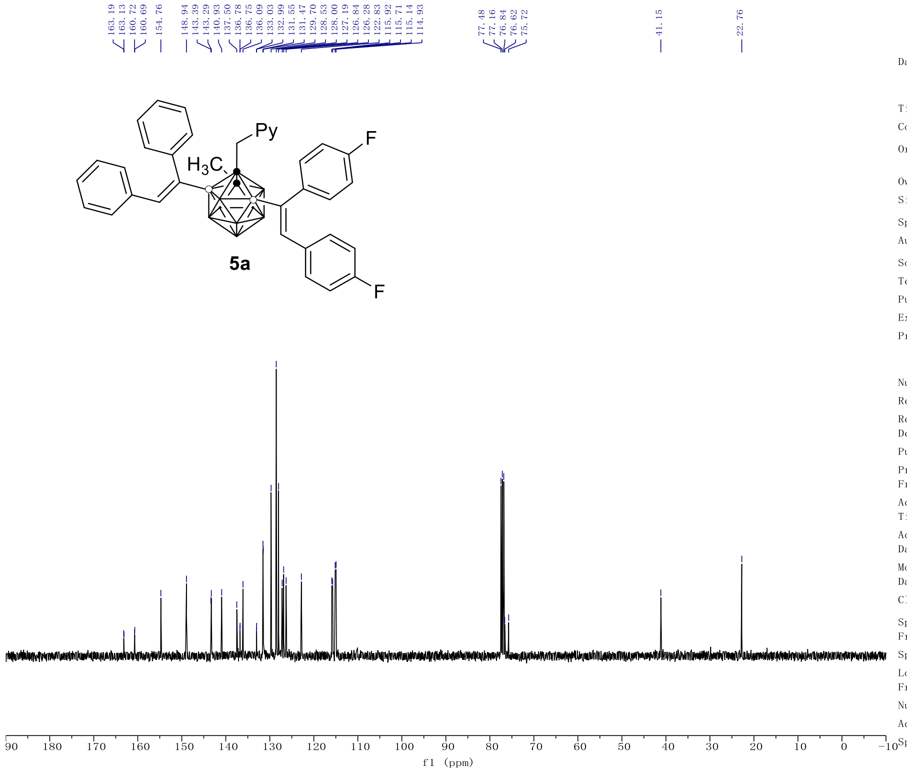

Parameter	Value
Data File Name	E:/ 核磁/ yss-3004-c/ 17/ fid
Title	yss-3004-c/ 17
Comment	
Origin	Bruker BioSpin GmbH
Owner	nmr
Site	
Spectrometer	spect
Author	
Solvent	CDCl3
Temperature	295.9
Pulse Sequence	zgpg30
Experiment	1D
Probe	Z116098_0640 (PA BBO 400S1 BBF-H-D-05 Z SP)
Number of Scans	42
Receiver Gain	196
Relaxation Delay	1.0000
Pulse Width	10.0000
Presaturation Frequency	
Acquisition Time	1.2999
Acquisition Date	2023-09-19T18:15:11
Modification Date	2023-09-19T18:15:12
Class	
Spectrometer Frequency	100.64
Spectral Width	25252.5
Lowest Frequency	-1551.4
Nucleus	13C
Acquired Size	32827
Spectral Size	131072

$^{11}B\{^1H\}$ NMR (128 MHz, $CDCl_3$) of **5a**

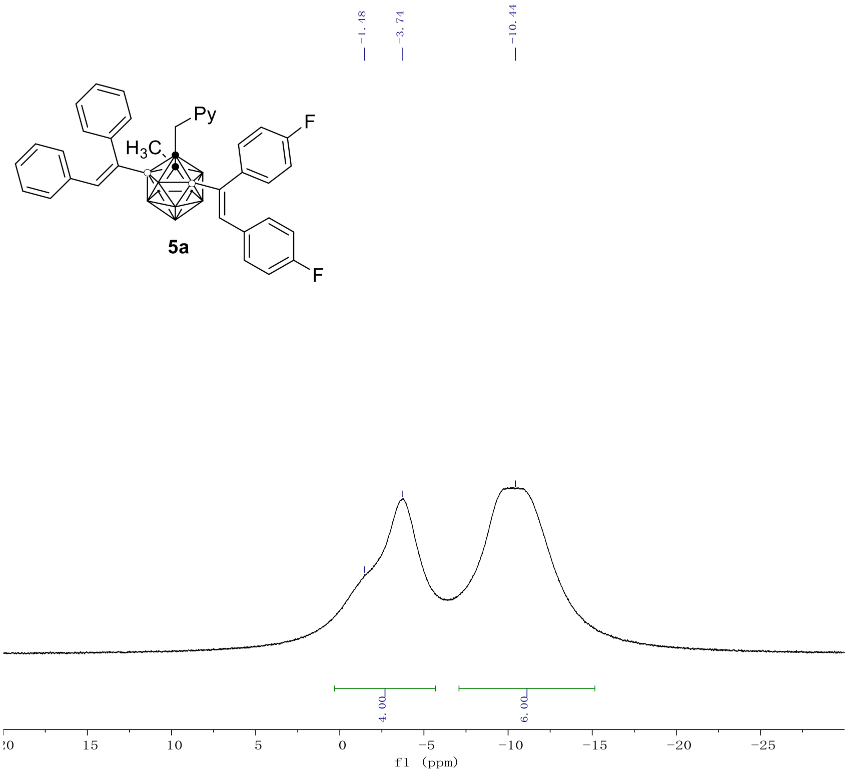

Parameter	Value
Data File Name	E:/ 核磁/ yss-3004-b/ 18 fid
Title	yss-3004-b/ 18
Comment	
Origin	Bruker BioSpin GmbH
Owner	nmr
Site	
Spectrometer	spect
Author	
Solvent	CDCl3
Temperature	295.9
Pulse Sequence	aring-b11
Experiment	1D
Probe	Z116098_0640 (PA BBO 400 BBF-H-D-05 Z SP)
Number of Scans	11
Receiver Gain	196
Relaxation Delay	1.0000
Pulse Width	10.0000
Presaturation Frequency	
Acquisition Time	1.2845
Acquisition Date	2023-09-19T18:17:40
Modification Date	2023-09-19T18:17:41
Class	
Spectrometer Frequency	128.40
Spectral Width	25510.2
Lowest Frequency	-12755.1
Nucleus	11B
Acquired Size	32768
Spectral Size	65536

^{11}B NMR (128 MHz, $CDCl_3$) of **5a**

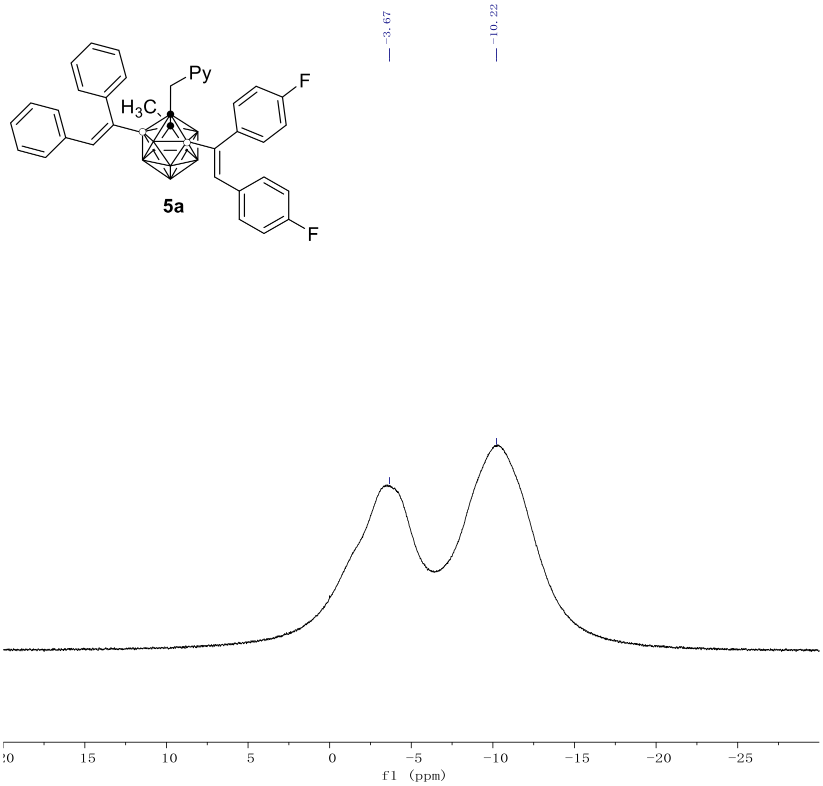

Parameter	Value
Data File Name	E:/ 核磁/ yss-3004-nob/ 19 fid
Title	yss-3004-nob/ 19
Comment	
Origin	Bruker BioSpin GmbH
Owner	nmr
Site	
Spectrometer	spect
Author	
Solvent	CDCl3
Temperature	296.0
Pulse Sequence	aring
Experiment	1D
Probe	Z116098_0640 (PA BBO 400S1 BBF-H-D-05 Z SP)
Number of Scans	11
Receiver Gain	196
Relaxation Delay	1.0000
Pulse Width	10.0000
Presaturation Frequency	
Acquisition Time	1.2845
Acquisition Date	2023-09-19T18:18:24
Modification Date	2023-09-19T18:18:25
Class	
Spectrometer Frequency	128.40
Spectral Width	25510.2
Lowest Frequency	-12755.1
Nucleus	11B
Acquired Size	32768
Spectral Size	65536

^{19}F NMR (376 MHz, $CDCl_3$) of **5a**

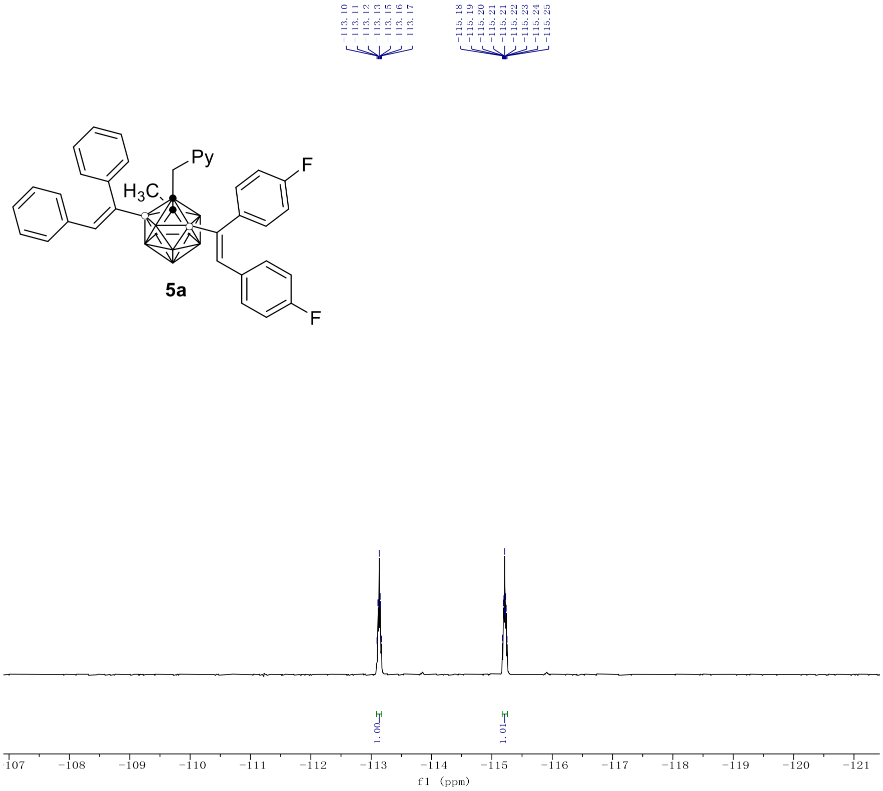

Parameter	Value
Data File Name	E:/ 核磁/ yss-3004-f.fid/ fid
Title	yss-3004-f
Comment	
Origin	Varian
Owner	
Site	
Spectrometer	vnmrs
Author	
Solvent	CDCl3
Temperature	25.0
Pulse Sequence	s2pul
Experiment	1D
Probe	4nuc
Number of Scans	4
Receiver Gain	60
Relaxation Delay	1.0000
Pulse Width	0.0000
Presaturation Frequency	
Acquisition Time	1.0000
Acquisition Date	2023-09-19T18:36:16
Modification Date	2023-09-19T10:36:00
Class	
Spectrometer Frequency	376.07
Spectral Width	147058.8
Lowest Frequency	-111478.7
Nucleus	19F
Acquired Size	147059
Spectral Size	524288

^{1}H NMR (400 MHz, $CDCl_3$) of **5b**

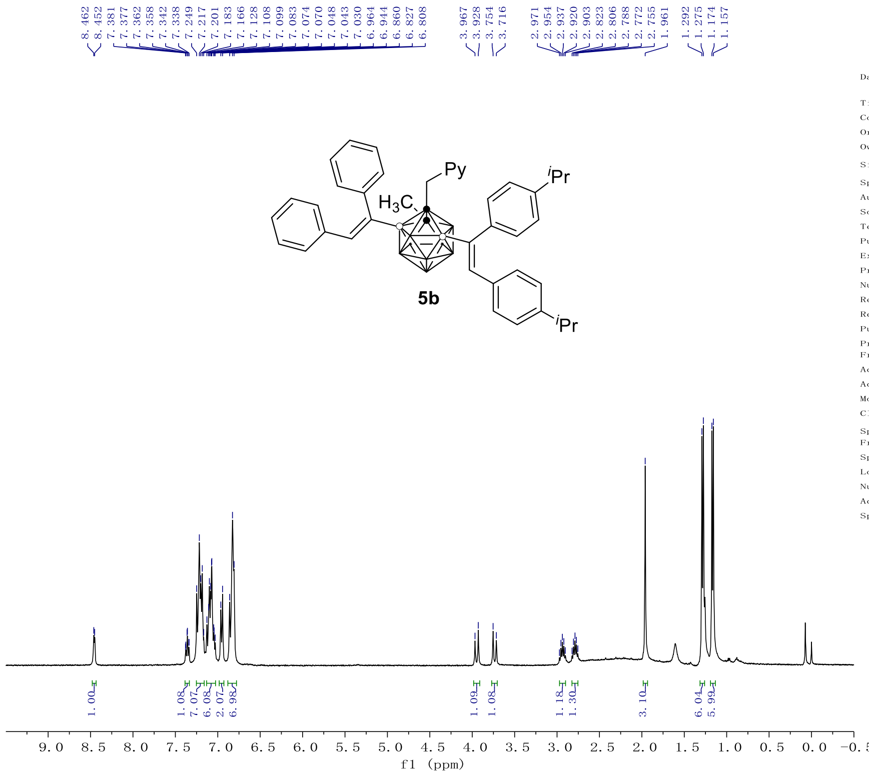

Parameter	Value
Data File Name	E:/ 积分核磁/ 3009/ yss-3009-h.fid/ fid
Title	yss-3009-h
Comment	Std proton
Origin	Varian
Owner	
Site	
Spectrometer	vnmrs
Author	omc1
Solvent	CDCl3
Temperature	25.0
Pulse Sequence	s2pul
Experiment	1D
Probe	ATB
Number of Scans	4
Receiver Gain	24
Relaxation Delay	1.0000
Pulse Width	0.0000
Presaturation Frequency	
Acquisition Time	3.0000
Acquisition Date	2023-09-30T16:40:20
Modification Date	2023-09-30T08:41:00
Class	
Spectrometer Frequency	400.03
Spectral Width	8445.9
Lowest Frequency	-1220.0
Nucleus	1H
Acquired Size	25338
Spectral Size	65536

^{13}C{^{1}H} NMR (101 MHz, $CDCl_3$) of **5b**

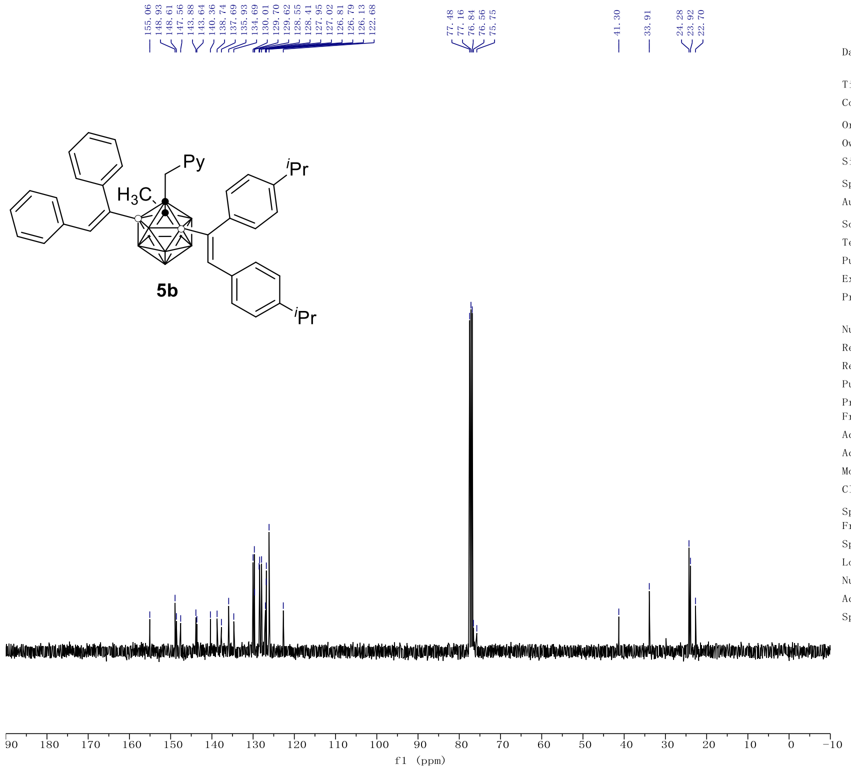

Parameter	Value
Data File Name	E:/ 积分核磁/ 3009/ yss-6-3009-c/ 11/ fid
Title	yss-6-3009-c/ 11
Comment	
Origin	Bruker BioSpin GmbH
Owner	nmr
Site	
Spectrometer	spect
Author	
Solvent	CDCl3
Temperature	296.0
Pulse Sequence	zgpg30
Experiment	1D
Probe	Z116098_0640 (PA BBO 400S1 BBF-H-D-05 Z SP)
Number of Scans	104
Receiver Gain	196
Relaxation Delay	1.0000
Pulse Width	10.0000
Presaturation Frequency	
Acquisition Time	1.2999
Acquisition Date	2023-09-30T17:25:41
Modification Date	2023-09-30T17:25:42
Class	
Spectrometer Frequency	100.64
Spectral Width	25252.5
Lowest Frequency	-1544.8
Nucleus	13C
Acquired Size	32827
Spectral Size	131072

$^{11}B\{^{1}H\}$ NMR (128 MHz, $CDCl_3$) of **5b**

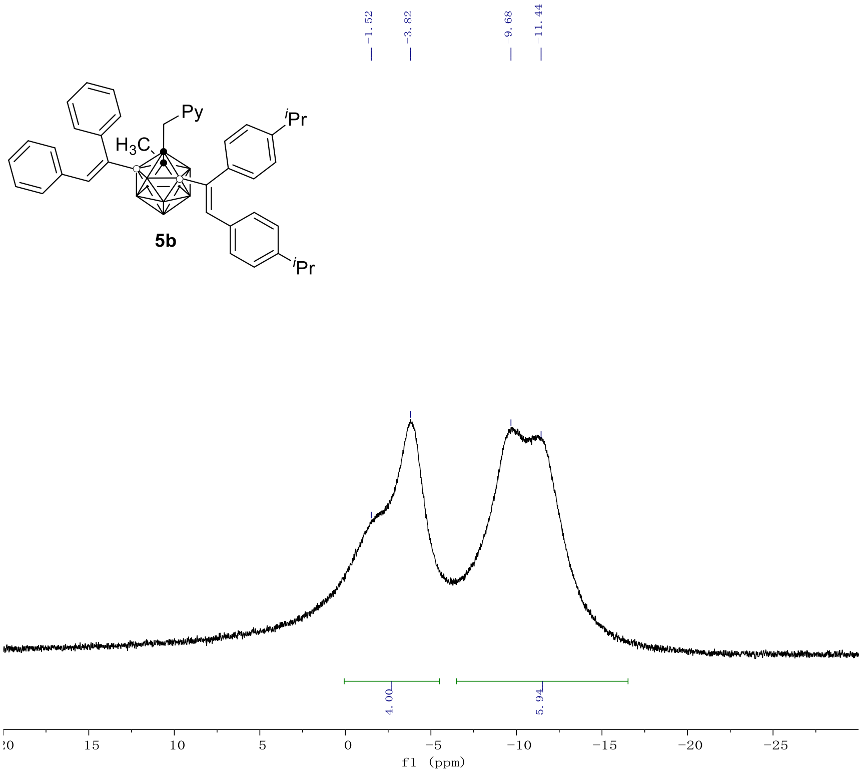

Parameter	Value
Data File Name	E:/ 积分核磁/ 3009/ yss--3009-b/ 12/ fid
Title	yss--3009-b/ 12
Comment	
Origin	Bruker BioSpin GmbH
Owner	nmr
Site	
Spectrometer	spect
Author	
Solvent	CDCl3
Temperature	296.0
Pulse Sequence	aring-b11
Experiment	1D
Probe	Z116098_0640 (PA BBO 40 BBF-H-D-05 Z SP)
Number of Scans	42
Receiver Gain	196
Relaxation Delay	1.0000
Pulse Width	10.0000
Presaturation Frequency	
Acquisition Time	1.2845
Acquisition Date	2023-09-30T17:29:45
Modification Date	2023-09-30T17:29:46
Class	
Spectrometer Frequency	128.40
Spectral Width	25510.2
Lowest Frequency	-12755.1
Nucleus	11B
Acquired Size	32768
Spectral Size	65536

^{11}B NMR (128 MHz, $CDCl_3$) of **5b**

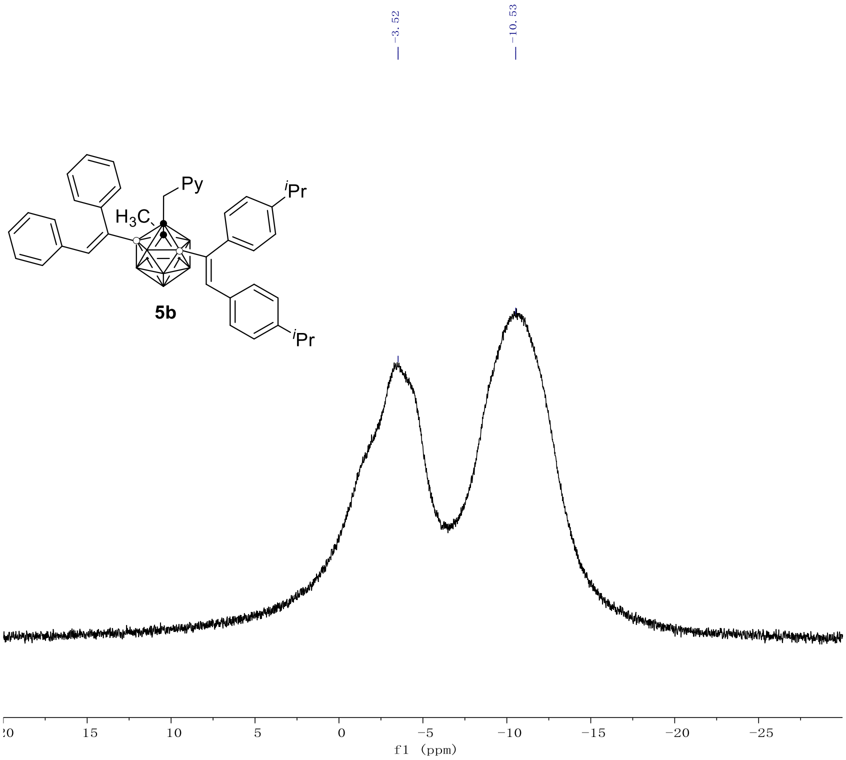

Parameter	Value
Data File Name	E:/ 积分核磁/ 3009/ yss--3009-nob/ 13/ fid
Title	yss--3009-nob/ 13
Comment	
Origin	Bruker BioSpin GmbH
Owner	nmr
Site	
Spectrometer	spect
Author	
Solvent	CDCl3
Temperature	296.0
Pulse Sequence	aring
Experiment	1D
Probe	Z116098_0640 (PA BBO 400 BBF-H-D-05 Z SP)
Number of Scans	41
Receiver Gain	196
Relaxation Delay	1.0000
Pulse Width	10.0000
Presaturation Frequency	
Acquisition Time	1.2845
Acquisition Date	2023-09-30T17:31:41
Modification Date	2023-09-30T17:31:42
Class	
Spectrometer Frequency	128.40
Spectral Width	25510.2
Lowest Frequency	-12755.1
Nucleus	11B
Acquired Size	32768
Spectral Size	65536
